# Supplementary material for: Decarboxylative oxidation-enabled consecutive C-C bond cleavage
Source: Nat Commun. 2022 Nov 18;13:7061. doi: 10.1038/s41467-022-34829-x (PMC9674625; doi:10.1038/s41467-022-34829-x)
Supplement: Supplementary file 1 — Supplementary Information [file 41467_2022_34829_MOESM1_ESM.pdf]

Supplementary information for decarboxylative oxidation-enabled consecutive C-C bond cleavage

Ruining Li[1#], Ya Dong[1#], Shah Nawaz Khan[1], Muhammad Kashif Zaman[1], Junliang Zhou[1], Pannan Miao[1], Lifu Hu[1] and Zhankui Sun[123*]

[1]Shanghai Key Laboratory for Molecular Engineering of Chiral Drugs, School of Pharmacy, Shanghai Jiao Tong University, No. 800 Dongchuan Rd., Shanghai, China

[2]Pharm-X Center, School of Pharmacy, Shanghai, Jiao Tong University, Shanghai, China

[3]AI Pharma Center, Zhangjiang Institute for Advanced Study, Shanghai Jiao Tong University, Shanghai, China

🖂email: zksun@sjtu.edu.cn

Table of Contents

1. Supplementary Methods 3

2. Supplementary Discussion 26

3. Supplementary Notes 36

4. Supplementary References 138

1. Supplementary Methods

1.1 Supplementary General Information

Commercial chemicals were used as supplied without further purification. The solvents were used directly from commercial reagents. The iridium photocatalysts [(dF(CF$_3$)ppy)$_2$-Ir- -Cl]$_2$ (**Ir-1**), [Ir(dF(CF$_3$)ppy)$_2$(dOMebpy)]PF$_6$ (**Ir-2**), [Ir(dF(CF$_3$)ppy)$_2$(dtbpy)]PF$_6$ (**Ir-3**) and [Ir(dF(CF$_3$)ppy)$_2$(bpy)]PF$_6$ (**Ir-4**) were synthesized following the published route[1]. Organic solutions were concentrated under reduced pressure on an IKA rotary evaporator using a water bath. Chromatographic purification of products was accomplished using force-flow chromatography on silica gel (200-300 mesh) according to the method of Still[2]. Thin-layer chromatography (TLC) was performed on 250 μm silica gel plates. TLC visualization was performed by UV lamp and KMnO$_4$ stain (1g KMnO$_4$, 1g NaHCO$_3$ and 100 mL H$_2$O). All key compounds were characterized by ^{1}H NMR, ^{13}C NMR and HRMS. ^{1}H NMR spectra were recorded at room temperature on an Agilent NMR Systems 400 MHz Spectrometer (^{1}H NMR at 400 MHz) and are internally referenced to residual protic CDCl$_3$ (*δ* 7.26 ppm) or protic DMSO-d_6 (2.50 ppm) unless otherwise noted. Data for ^{1}H NMR are reported as follows: chemical shift (*δ* ppm), multiplicity (s = singlet, d = doublet, t = triplet, q = quartet, m = multiplet, dd = doublet of doublets, dt = doublet of triplets, brs = broad singlet), and coupling constant (Hz). ^{13}C NMR spectra were recorded at room temperature on an Agilent NMR Systems 400 MHz Spectrometer (^{13}C NMR at 101 MHz) and data are reported in terms of chemical shift relative to CDCl$_3$ (77.16 ppm) or DMSO-d_6 (39.52 ppm). For ^{1}H NMR analysis of crude reaction mixtures, mesitylene was used as internal standard, and added as a pure liquid. HRMS: electrospray ionization mass (ESI-MS) was performed on Agilent 6210 Series TOF MS. The blue LEDs were purchased from supermarket and directly used without any filters. The brand name of LEDs is Jin Dian Yuan and the model number is JDY-TG01.

1.2 Supplementary Synthesis and Characterization of Substrates

General procedure A for the synthesis of acids

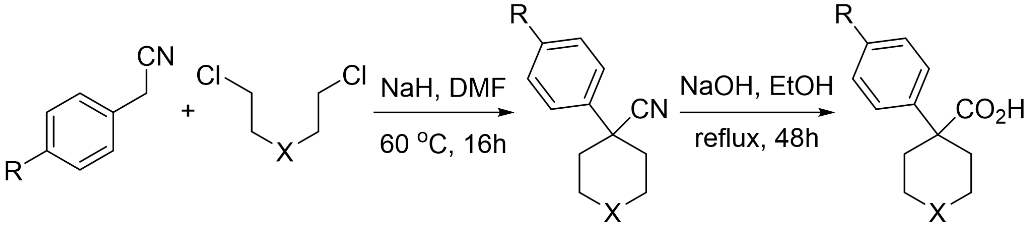

Supplementary Fig. 1. Preparation of acids from commercial compounds in two steps[2]

General procedure A for preparation of acids:

To a solution of substituted benzeneacetonitrile (2.00 g) and tert-butyl bis(2-chloroethyl)carbamate (1.0 equiv.) or 1-chloro-2-(2-chloroethoxy)ethane (1.0 equiv.) in DMF (25 mL) was added NaH (60% dispersion in mineral oil, 3.5 equiv.) portion wise at 0 °C over 15 min and the reaction was stirred at room temperature for 1 hour. Then the mixture was heated at 60 °C for 16 hours. The reaction mixture was poured into ice-water and extracted with ethyl acetate (3*50 mL). The combined organic layers were washed with brine (50 mL), dried over Na_2SO_4, filtered and concentrated. The crude product was purified by silica gel chromatography eluting with hexane/ethyl acetate (15 : 1) to provide the desired carbonitriles.

The mixture of carbonitrile in EtOH (20 mL) and 10 N NaOH (20 mL) was refluxed for 48 hours, cooled to room temperature, and then added 1 N HCl until pH to 5~6. The mixture was extracted by ethyl acetate (3*50 mL). The combined organic layers were washed with brine, dried over Na_2SO_4, filtered and concentrated. The crude product was recrystallized by ethyl acetate to give the desired acids.

1-(tert-butoxycarbonyl)-4-phenylpiperidine-4-carboxylic acid (1a)

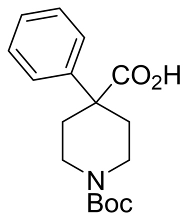

Prepared from phenylacetonitrile (2.00 g, 17.1 mmol) and tert-butyl bis(2-chloroethyl)carbamate (4.14 g, 17.1 mmol) according to the general procedures as a white solid (4.00 g, 77% yield for two steps). Data are consistent with those reported in the literature.[2]

^{1}H NMR (400 MHz, $CDCl_3$) δ 10.91 (brs, 1H), 7.40 (d, J = 7.7 Hz, 2H), 7.34 (t, J = 7.6 Hz, 2H), 7.27 (t, J = 7.2 Hz, 1H), 3.91 (brs, 2H), 3.09 (brs, 2H), 2.50 (d, J = 13.5 Hz, 2H), 1.86 (t, J = 10.5 Hz, 2H), 1.44 (s, 9H); ^{13}C NMR (101 MHz, $CDCl_3$) δ 179.6, 155.1, 141.6, 128.8, 127.6, 126.1, 79.9, 49.2, 41.6, 33.4, 28.5; HRMS (ESI) calcd for $C_{17}H_{23}NNaO_4$ [M + Na]$^+$ m/z = 328.1519, found: 328.1527.

1-benzoyl-4-phenylpiperidine-4-carboxylic acid (2a)

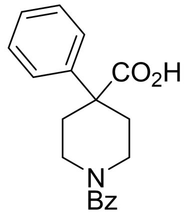

To a solution of **1a** (2.00 g, 6.56 mmol, 1.0 equiv.) in DCM (30 mL) was added TFA (3.00 mL, 40.4 mmol, 6.0 equiv.) portion wise at 0 ºC and the reaction was stirred at room temperature for overnight and then concentrated. The crude product was used directly for the next step.

To the solution of the crude product in DCM (20 mL) was added DIPEA (2.16 mL, 13.1 mmol, 2.0 equiv.) and BzCl (0.91 mL, 7.87 mmol, 1.2 equiv.) at 0 ºC and the reaction was stirred at room temperature for overnight. The mixture was quenched by H_2O (10 mL) and extracted by ethyl acetate (3*50 mL). The combined organic layer was washed with brine, dried over Na_2SO_4, filtered and concentrated. The crude product was purified by silica gel chromatography eluting with hexane/ethyl acetate (4 : 1) to provide **2a** (910 mg, 45% for two steps) as a white solid.

^{1}H NMR (400 MHz, DMSO-*d$_6$*) δ 12.82 (brs, 1H), 7.48 - 7.32 (m, 9H), 7.27 (t, J = 7.1 Hz, 1H), 4.30 (brs, 1H), 3.54 (brs, 1H), 3.32 - 2.98 (m, 2H), 2.55 - 2.47 (m, 2H), 1.84 (brs, 2H); ^{13}C NMR (101 MHz, DMSO-*d$_6$*) δ 175.2, 169.1, 142.5, 136.2, 129.5, 128.7, 128.5, 127.2, 126.9, 125.9, 49.1, 45.2, 40.0, 33.8, 33.1; HRMS (ESI) calcd for $C_{19}H_{19}NNaO_3$ [M + Na]$^+$ m/z = 332.1257, found: 332.1252; IR ν_{max}/cm^{-1} (film) : 2929, 1718, 1571, 1447, 1228, 1200, 1007, 721, 696.

1-(tert-butoxycarbonyl)-4-(4-fluorophenyl)piperidine-4-carboxylic acid (3a)

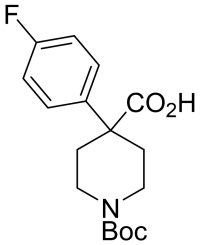

Prepared from 2-(4-fluorophenyl)acetonitrile (2.00 g, 14.8 mmol) and tert-butyl bis(2-chloroethyl)carbamate (3.59 g, 14.8 mmol) according to the general procedures as a white solid (2.30 g, 48% yield for two steps). Data are consistent with those reported in the literature.[3]

^{1}H NMR (400 MHz, $CDCl_3$) δ 11.12 (brs, 1H), 7.36 (dd, J = 8.4, 5.3 Hz, 2H), 7.02 (t, J = 8.5 Hz, 2H), 3.91 (brs, 2H), 3.06 (brs, 2H), 2.47 (d, J = 13.3 Hz, 2H), 1.79 (t, J = 10.5 Hz, 2H), 1.43 (s, 9H); ^{13}C NMR (101 MHz, $CDCl_3$) δ 179.3, 162.1 (d, J = 248 Hz), 155.1, 137.4 (d, J = 3.2 Hz), 127.8 (d, J = 8.1 Hz), 115.6 (d, J = 21.3 Hz), 80.1, 48.7, 41.8, 33.5, 28.5; HRMS (ESI) calcd for $C_{17}H_{22}FNNaO_4$ [M + Na]$^+$ m/z = 346.1425, found: 346.1530.

1-(tert-butoxycarbonyl)-4-(4-chlorophenyl)piperidine-4-carboxylic acid (4a)

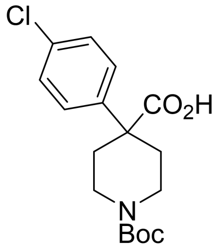

Prepared from 2-(4-chlorophenyl)acetonitrile (2.00 g, 10.8 mmol) and tert-butyl bis(2-chloroethyl)carbamate (2.62 g, 10.8 mmol) according to the general procedures as a white solid (1.80 g, 49% yield for two steps). Data are consistent with those reported in the literature.[3]

^{1}H NMR (400 MHz, CDCl$_3$) δ 11.10 (brs, 1H), 7.36 - 7.27 (m, 4H), 3.90 (brs, 2H), 3.06 (brs, 2H), 2.46 (d, J = 13.5 Hz, 2H), 1.87 - 1.72 (m, 2H), 1.43 (s, 9H); ^{13}C NMR (101 MHz, CDCl$_3$) δ 179.0, 155.0, 140.1, 133.6, 129.0, 127.5, 80.2, 48.8, 41.5, 33.3, 28.5; HRMS (ESI) calcd for $C_{17}H_{22}ClNNaO_4$ [M + Na]$^+$ m/z = 362.1130, found: 362.1131.

1-(tert-butoxycarbonyl)-4-(4-(trifluoromethyl)phenyl)piperidine-4-carboxylic acid (5a)

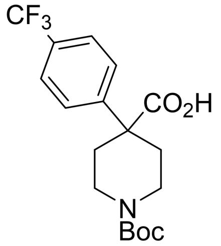

Prepared from 2-(4-(trifluoromethyl)phenyl)acetonitrile (2.00 g, 10.8 mmol) and tert-butyl bis(2-chloroethyl)carbamate (2.62 g, 10.8 mmol) according to the general procedures as a white solid (750 mg, 19% yield for two steps).

^{1}H NMR (400 MHz, CDCl$_3$) δ 11.41 (brs, 1H), 7.59 (d, J = 8.4 Hz, 2H), 7.52 (d, J = 8.3 Hz, 2H), 3.95 (d, J = 10.0 Hz, 2H), 3.08 (brs, 2H), 2.51 (d, J = 13.4 Hz, 2H), 1.91 - 1.77 (m, 2H), 1.43 (s, 9H); ^{13}C NMR (101 MHz, CDCl$_3$) δ 178.3, 155.1, 145.7, 130.0 (q, J = 32.7 Hz), 126.6, 125.7 (q, J = 3.7 Hz), 124.0 (d, J = 271 Hz), 80.4, 49.4, 41.2, 33.4, 28.5; HRMS (ESI) calcd for $C_{17}H_{22}F_3NNaO_4$ [M + Na]$^+$ m/z = 396.1393, found: 396.1395; IR ν_{max}/cm^{-1} (film) : 2978, 1731, 1699, 1654, 1439, 1328, 1168, 1128, 1073, 1018, 930, 839.

1-(tert-butoxycarbonyl)-4-(4-methoxyphenyl)piperidine-4-carboxylic acid (6a)

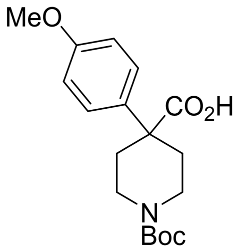

Prepared from 2-(4-methoxyphenyl)acetonitrile (2.00 g, 13.6 mmol) and tert-butyl bis(2-chloroethyl)carbamate (3.30 g, 13.6 mmol) according to the general procedures as a white solid (2.50 g, 55% yield for two steps). Data are consistent with those reported in the literature.[2]

^{1}H NMR (400 MHz, CDCl$_3$) δ 11.14 (brs, 1H), 7.31 (d, J = 8.8 Hz, 2H), 6.87 (d, J = 8.8 Hz, 2H), 3.89 (brs, 2H), 3.78 (s, 3H), 3.08 (brs, 2H), 2.47 (d, J = 13.5 Hz, 2H), 1.82 (t, J = 10.4 Hz, 2H), 1.44 (s, 9H); ^{13}C NMR (101 MHz, CDCl$_3$) δ 179.9, 158.8, 154.9, 133.4, 127.1, 114.0, 79.7, 55.2, 48.3, 41.5, 33.2, 28.4; HRMS (ESI) calcd for $C_{18}H_{25}NNaO_5$ [M + Na]$^+$ m/z = 358.1625, found: 358.1629.

1-(tert-butoxycarbonyl)-4-(4-(tert-butyl)phenyl)piperidine-4-carboxylic acid (7a)

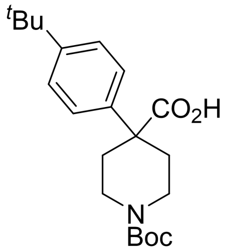

Prepared from 2-(4-(tert-butyl)phenyl)acetonitrile (2.00 g, 11.6 mmol) and tert-butyl bis(2-chloroethyl)carbamate (2.80 g, 11.6 mmol) according to the general procedures as a white solid (2.76 g, 66% yield for two steps). Data are consistent with those reported in the literature.[2]

^{1}H NMR (400 MHz, DMSO-*d6*) *δ* 12.75 (brs, 1H), 7.36 (d, *J* = 8.5 Hz, 2H), 7.30 (d, *J* = 8.5 Hz, 2H), 3.80 (d, *J* = 13.4 Hz, 2H), 2.94 (brs, 2H), 2.35 (d, *J* = 13.5 Hz, 2H), 1.76 - 1.61 (m, 2H), 1.39 (s, 9H), 1.24 (d, *J* = 11.7 Hz, 9H); ^{13}C NMR (101 MHz, DMSO-*d6*) *δ* 175.2, 154.1, 149.3, 139.7, 125.5, 125.3, 78.8, 48.3, 41.7, 34.1, 33.2, 31.1, 28.1; HRMS (ESI) calcd for $C_{21}H_{31}NNaO_4$ $[M + Na]^+$ m/z = 384.2145, found: 384.2152.

4-([1,1'-biphenyl]-4-yl)-1-(tert-butoxycarbonyl)piperidine-4-carboxylic acid (8a)

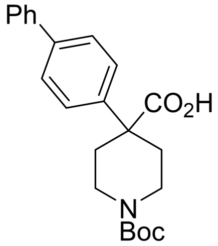

Prepared from 2-([1,1'-biphenyl]-4-yl)acetonitrile (2.00 g, 10.4 mmol) and tert-butyl bis(2-chloroethyl)carbamate (2.51 g, 10.4 mmol) according to the general procedures as a white solid (1.88 g, 47% yield for two steps).

^{1}H NMR (400 MHz, DMSO-*d6*) *δ* 7.64 (t, *J* = 7.7 Hz, 4H), 7.52 - 7.42 (m, 4H), 7.37 (d, *J* = 7.2 Hz, 1H), 3.82 (d, *J* = 13.4 Hz, 2H), 2.99 (brs, 2H), 2.40 (d, *J* = 13.3 Hz, 2H), 1.88 -1.67 (m, 2H), 1.39 (s, 9H). ^{13}C NMR (101 MHz, DMSO-*d6*) *δ* 175.0, 154.0, 141.8 139.7, 138.9, 128.9, 127.5, 126.8, 126.6, 126.4, 78.7, 48.5, 41.4, 33.2, 28.1; HRMS (ESI) calcd for $C_{23}H_{27}NNaO_4$ $[M + Na]^+$ m/z = 404.1832, found: 404.1838; IR ν_{max}/cm^{-1} (film) : 2962, 2925, 1719, 1654, 1458, 1261, 1094, 1019, 799.

1-(tert-butoxycarbonyl)-4-(thiophen-3-yl)piperidine-4-carboxylic acid (9a)

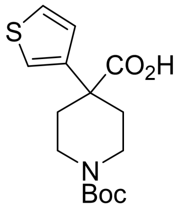

Prepared from 2-(thiophen-3-yl)acetonitrile (1.00 g, 8.13 mmol) and tert-butyl bis(2-chloroethyl)carbamate (1.97 g, 8.13 mmol) according to the general procedures as a white solid (1.32 g, 52% yield for two steps).

^{1}H NMR (400 MHz, $CDCl_3$) *δ* 10.10 (s, 1H), 7.27 (dd, *J* = 4.7, 3.3 Hz, 1H), 7.15 - 7.12 (m, 1H), 7.09 (d, *J* = 5.2 Hz, 1H), 3.78 (d, *J* = 12.1 Hz, 2H), 3.13 (t, *J* = 11.4 Hz, 2H), 2.42 (d, *J* = 13.7 Hz,

2H), 1.88 (t, J = 10.2 Hz, 2H), 1.42 (s, 9H); ^{13}C NMR (101 MHz, $CDCl_3$) δ 179.2, 155.0, 142.7, 126.1, 126.1, 121.5, 80.0, 47.4, 41.3, 33.7, 28.5; HRMS (ESI) calcd for $C_{15}H_{21}NNaO_4S$ [M + Na]$^+$ m/z = 334.1083, found: 334.1084; IR ν_{max}/cm^{-1} (film) : 3102, 2972, 1718, 1636, 1438, 1365, 1223, 1188, 1135, 852, 775, 695.

Procedure for the synthesis of 1-(*tert*-butoxycarbonyl)-4-(1-methyl-1*H*-indol-3-y--l)piperidine-4-carboxylic acid (10a)

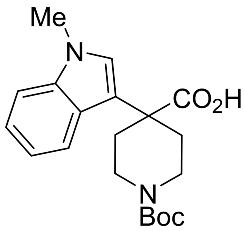

Prepared from 2-(1-methyl-1H-indol-3-yl) acetonitrile (1.30 g) and tert-butyl bis(2-chloroethyl)carbamate (1.0 equiv.) according to the general procedures, white solid (890 mg, 84% yield).

^{1}H NMR (400 MHz, $CDCl_3$) 7.79 (d, J = 8.1 Hz, 1H), 7.27 (dd, J = 8.3, 1.0 Hz, 1H), 7.23-7.17 (m, 1H), 7.06-7.00(m, 1H), 6.91 (s, 1H), 3.86 (d, J = 23.0 Hz, 2H), 3.72 (s, 3H), 3.20 (d, J = 13.8 Hz, 2H), 2.59 (d, J = 13.5 Hz, 2H), 1.99 (dd, J = 24.0, 3.9 Hz, 2H), 1.44 (s, 9H); ^{13}C NMR (101 MHz, $CDCl_3$) δ 179.9, 155.0, 137.5, 126.4, 125.8, 121.7, 120.9, 119.3, 115.4, 109.5, 79.6, 45.4, 42.2, 33.1, 32.8, 28.4; HRMS (ESI) calcd for $C_{20}H_{26}N_2NaO_4$ [M + H]$^+$ m/z = 381.1785, found:381.1863; IR ν_{max}/cm^{-1} (film) :.2973, 1724, 1425, 1366, 1168, 1078, 765, 548.

4-phenyltetrahydro-2H-pyran-4-carboxylic acid (49a)

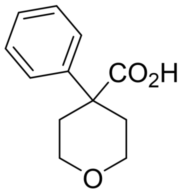

Prepared from phenylacetonitrile (2.00 g, 17.1 mmol) and 1-chloro-2-(2-chloroethoxy)ethane (2.00 mL, 17.1 mmol) according to the general procedures as a white solid (2.31 g, 66% yield for two steps).

^{1}H NMR (400 MHz, $CDCl_3$) δ 10.81 (s, 1H), 7.43 (d, J = 7.4 Hz, 2H), 7.37 (t, J = 7.6 Hz, 2H), 7.30 (t, J = 7.1 Hz, 1H), 3.95 (dt, J = 11.9, 3.6 Hz, 2H), 3.65 (t, J = 11.2 Hz, 2H), 2.53 (d, J = 13.2 Hz, 2H), 2.11 - 1.93 (m, 2H); ^{13}C NMR (101 MHz, $CDCl_3$) δ 180.0, 141.8, 128.9, 127.6, 126.0, 65.6, 48.4, 34.1; IR ν_{max}/cm^{-1} (film) : 2952, 1883, 1720, 1438, 1301, 1224, 1205, 1128, 1023, 932, 826, 700.

4-(4-fluorophenyl)tetrahydro-2H-pyran-4-carboxylic acid (50a)

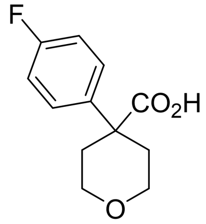

Prepared from 2-(4-fluorophenyl)acetonitrile (2.00 g, 14.8 mmol) and 1-chloro-2-(2-chloroethoxy)ethane (1.73 mL, 14.8 mmol) according to the general procedures as a white solid (2.50 g, 75% yield for two steps).

^{1}H NMR (400 MHz, DMSO-*d$_6$*) δ 7.42 (dd, *J* = 8.2, 5.8 Hz, 2H), 7.17 (t, *J* = 8.8 Hz, 2H), 3.80 (d, *J* = 11.8 Hz, 2H), 3.45 (t, *J* = 11.2 Hz, 2H), 2.37 (d, *J* = 13.2 Hz, 2H), 1.88 - 1.72 (m, 2H).; ^{13}C NMR (101 MHz, DMSO-*d$_6$*) δ 175.3, 161.2 (d, *J* = 245 Hz), 139.3 (d, *J* = 2.9 Hz), 127.9 (d, *J* = 8.2 Hz), 115.2 (d, *J* = 21.2 Hz), 64.9, 47.5, 34.2; IR ν_{max}/cm^{-1} (film) : 2970, 2881, 1718, 1512, 1301, 1235, 1221, 1131, 1099, 1029, 895, 828, 737.

4-(4-chlorophenyl)tetrahydro-2H-pyran-4-carboxylic acid (51a)

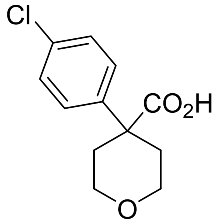

Prepared from 2-(4-chlorophenyl)acetonitrile (2.00 g, 13.2 mmol) and 1-chloro-2-(2-chloroethoxy)ethane (1.55 mL, 13.2 mmol) according to the general procedures as a white solid (2.50 g, 79% yield for two steps).

^{1}H NMR (400 MHz, DMSO-*d$_6$*) δ 12.71 (brs, 1H), 7.41 (s, 4H), 3.80 (dt, *J* = 11.7, 3.5 Hz, 2H), 3.44 (t, *J* = 11.2 Hz, 2H), 2.35 (d, *J* = 13.1 Hz, 2H), 1.85 - 1.75 (m, 2H); ^{13}C NMR (101 MHz, DMSO-*d$_6$*) δ 175.0, 142.1, 131.8, 128.5, 127.8, 64.8, 47.6, 33.9; IR ν_{max}/cm^{-1} (film) : 2967, 2861, 1730, 1685, 1491, 1249, 1110, 1092, 1011, 819, 669.

4-(4-methoxyphenyl)tetrahydro-2H-pyran-4-carboxylic acid (52a)

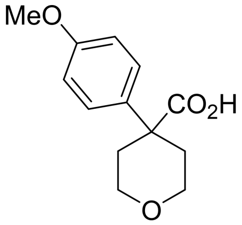

Prepared from 2-(4-methoxyphenyl)acetonitrile (2.00 g, 13.6 mmol) and 1-chloro-2-(2-chloroethoxy)ethane (1.59 mL, 13.6 mmol) according to the general procedures as a white solid (2.03 g, 63% yield for two steps). Data are consistent with those reported in the literature.[4]

^{1}H NMR (400 MHz, DMSO-*d$_6$*) δ 12.45 (brs, 1H), 7.35 - 7.28 (m, 2H), 6.95 - 6.89 (m, 2H), 3.84 - 3.76 (m, 2H), 3.73 (s, 3H), 3.45 (t, *J* = 10.8 Hz, 2H), 2.36 (d, *J* = 13.2 Hz, 2H), 1.85 - 1.72 (m, 2H).; ^{13}C NMR (101 MHz, DMSO-*d$_6$*) δ 175.7, 158.2, 135.1, 126.9, 113.9, 65.0, 55.1, 47.2, 34.3.

4-([1,1'-biphenyl]-4-yl)tetrahydro-2H-pyran-4-carboxylic acid (53a)

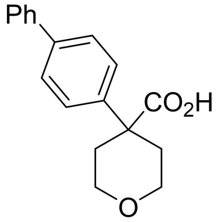

Prepared from 2-([1,1'-biphenyl]-4-yl)acetonitrile (2.00 g, 10.4 mmol) and 1-chloro-2-(2-chloroethoxy)ethane (1.21 mL, 10.4 mmol) according to the general procedures as a white solid (1.14 g, 39% yield for two steps).

^{1}H NMR (400 MHz, DMSO-d_6) δ 12.71 (s, 1H), 7.68 - 7.63 (m, 4H), 7.51 - 7.43 (m, 4H), 7.36 (t, J = 7.3 Hz, 1H), 3.84 (d, J = 11.7 Hz, 2H), 3.48 (t, J = 10.7 Hz, 2H), 2.41 (d, J = 13.2 Hz, 2H), 1.93 - 1.82 (m, 2H).; ^{13}C NMR (101 MHz, DMSO-d_6) δ 175.2, 142.2, 139.7, 138.8, 128.9, 127.5, 126.8, 126.6, 126.3, 64.9, 47.7, 34.0; IR ν_{max}/cm^{-1} (film) : 2968, 1701, 1684, 1654, 1508, 1457, 1244, 1026, 823, 698.

General procedure B for the synthesis of acids

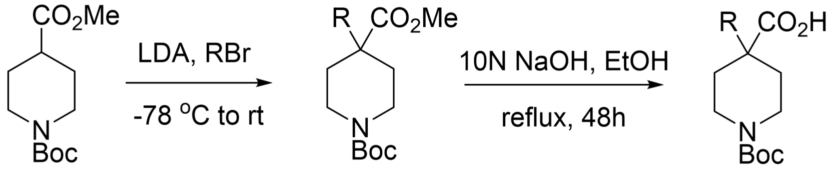

Supplementary Fig. 2. Preparation of acids from commercial compounds in two steps.

General procedure B for the preparation of acids:

To a stirred solution of LDA (2.0 M in solution in hexane, 6.20 mL, 12.3 mmol, 1.5 equiv.) in THF (20 mL) was added dropwise a solution of 1-(tert-butyl) 4-methyl piperidine-1,4-dicarboxylate (2.00 g, 8.23 mmol, 1.0 equiv.) in THF (10 mL) at -78 °C under N_2. The mixture was stirred at this temperature for 1 h before the solution of RBr (1.5 equiv.) in THF (10 mL) was added. Then the reaction was warmed to room temperature and stirred overnight. The mixture was quenched with sat. NH_4Cl (20 mL) and extracted with ethyl acetate (3*50 mL). The combined organic layer was washed with brine, dried over Na_2SO_4, filtered and concentrated. The crude product was purified by silica gel chromatography eluting with hexane/ethyl acetate (15 : 1) to provide the desired esters.

To the ester in EtOH (20 mL) was added 10 N NaOH (20 mL). The mixture was refluxed for 48 hours before it was cooled to room temperature and then neutralized with 1 N HCl until pH to 5~6. The mixture was extracted by ethyl acetate (3*50 mL). The combined organic layer was washed with brine, dried over Na_2SO_4, filtered and concentrated. The crude product was recrystallized by ethyl acetate to give the desired acids.

4-allyl-1-(tert-butoxycarbonyl)piperidine-4-carboxylic acid (17a)

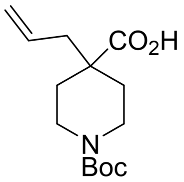

Prepared from 1-(tert-butyl) 4-methyl piperidine-1,4-dicarboxylate (2.00 g, 8.23 mmol) and 3-bromoprop-1-ene (1.06 mL, 12.3 mmol) according to the general procedures as a white solid (1.56 g, 70% yield for two steps). Data are consistent with those reported in the literature.[5]

^{1}H NMR (400 MHz, $CDCl_3$) δ 9.16 (brs, 1H), 5.74 - 5.63 (m, 1H), 5.07 (s, 1H), 5.04 - 5.02 (m, 1H), 3.83 (brs, 2H), 2.96 (t, J = 11.2 Hz, 2H), 2.28 (d, J = 7.4 Hz, 2H), 2.06 (d, J = 13.6 Hz, 2H), 1.41 (s, 9H), 1.39 - 1.31 (m, 2H); ^{13}C NMR (101 MHz, $CDCl_3$) δ 181.0, 155.1, 132.5, 118.9, 79.8, 45.9, 44.2, 41.2, 32.8, 28.6; HRMS (ESI) calcd for $C_{14}H_{23}NNaO_4$ $[M + Na]^+$ m/z = 292.1519, found: 292.1521.

4-benzyl-1-(tert-butoxycarbonyl)piperidine-4-carboxylic acid (18a)

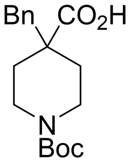

Prepared from 1-(tert-butyl) 4-methyl piperidine-1,4-dicarboxylate (2.00 g, 8.23 mmol) and BnBr (1.47 mL, 12.3 mmol) according to the general procedures as a white solid (1.20 g, 46% yield for two steps). Data are consistent with those reported in the literature.[6]

^{1}H NMR (400 MHz, $CDCl_3$) δ 7.27-7.22 (m, 3H), 7.10 - 7.08 (m, 2H), 3.94 (brs, 2H), 2.94-2.80 (m, 2H), 2.86 (s, 2H), 2.05 (d, J = 13.5 Hz, 2H), 1.47-1.43 (m, 2H), 1.43 (s, 9H); ^{13}C NMR (101 MHz, $CDCl_3$) δ 180.7, 155.1, 136.1, 130.0, 128.2, 127.0, 79.9, 47.3, 46.4, 41.4, 33.0, 28.5; HRMS (ESI) calcd for $C_{18}H_{25}NNaO_4$ $[M + Na]^+$ m/z = 342.1676, found: 342.1679.

1-(tert-butoxycarbonyl)-4-isopropylpiperidine-4-carboxylic acid (19a)

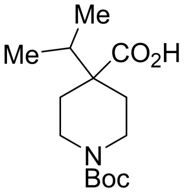

Prepared from 1-(tert-butyl) 4-methyl piperidine-1,4-dicarboxylate (2.00 g, 8.23 mmol) and 2-bromopropane (2.32 mL, 12.3 mmol) according to the general procedures as a white solid (0.50 g, 22% yield for two steps).

^{1}H NMR (400 MHz, $CDCl_3$) δ 10.77 (brs, 1H), 4.00 (brs, 2H), 2.78 (s, 2H), 2.04 (d, J = 13.1 Hz, 2H), 1.79 - 1.72 (m, 1H), 1.36 (s, 9H), 1.38- 1.31 (m, 2H), 0.90 (d, J = 6.9 Hz, 6H); ^{13}C NMR (101 MHz, $CDCl_3$) δ 180.9, 155.1, 79.7, 49.4, 41.8, 36.2, 30.6, 28.6, 17.6; HRMS (ESI) calcd for $C_{14}H_{25}NNaO_4$ $[M + Na]^+$ m/z = 294.1676, found: 294.1678.

2-hydroxy-1,2-dimethylcyclopentane-1-carboxylic acid (38a)

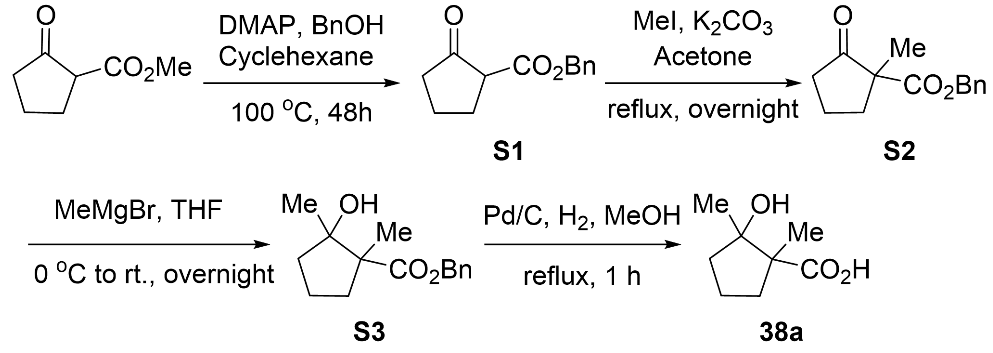

Supplementary Fig. 3. Preparation of **38a** from commercial compounds in four steps

A mixture of methyl 2-cyclopentanonecarboxylate (1.42 g, 10.0 mmol), phenylmethanol (1.19 g, 11.0 mmol, 1.1 equiv.) and DMAP (61 mg, 0.50 mmol, 0.05 equiv.) in cyclohexane (20 mL) was heated overnight in a Dean-Stark trap. The reaction mixture was cooled to room temperature, quenched with H_2O (20 mL) and then extracted with ethyl acetate (3*50 mL). The combined organic layers were washed with brine (50 mL), dried over Na_2SO_4, filtered and concentrated. The crude product was purified by silica gel chromatography eluting with hexane/ethyl acetate (10: 1) to provide the desired intermediate **S1** as colorless oil (1.16 g, 53% yield).

To the mixture of **S1** (1.16 g, 5.30 mmol) and K_2CO_3 (2.93 g, 21.2 mmol, 4.0 equiv.) in acetone (20 mL) was added MeI (1.32 mL, 21.2 mmol, 4.0 equiv.) by dropwise under N_2 and the resulting mixture was stirred and refluxed overnight. The reaction was cooled to room temperature, quenched with H_2O (20 mL) and extracted with ethyl acetate (3*50 mL). The combined organic layers were washed with brine (50 mL), dried over Na_2SO_4, filtered and concentrated. The crude product was purified by silica gel chromatography eluting with hexane/ethyl acetate (5: 1) to provide the desired intermediate **S2** as colorless oil (719 mg, 58% yield).

To a solution of **S2** (719 mg, 3.10 mmol) in dry THF (25 mL) was added MeMgBr (4.65 mL, 4.65 mmol, 1.0 M in THF, 1.5 equiv.) by dropwise at 0 °C over 5 min and the reaction was stirred at 0 °C for 1 hour. Then the mixture was allowed to stir at room temperature overnight. The reaction mixture was quenched with sat. NH_4Cl and extracted with ethyl acetate (3*50 mL). The combined organic layers were washed with brine (50 mL), dried over Na_2SO_4, filtered and concentrated. The crude product was purified by silica gel chromatography eluting with hexane/ethyl acetate (5: 1) to provide **S3** as colorless oil (347 mg, 45% yield).

To a stirred solution of **S3** (347 mg, 1.40 mmol) in degassed methanol (5 mL) was added 10% palladium on carbon (12 mg). The reaction mixture was then stirred under a hydrogen atmosphere for 1 hour then filtered through celite. The filtrate was concentrated in vacuo and recrystallized by ethyl acetate to give **38a** as a white solid (205 mg, 93% yield).

1H NMR (400 MHz, $CDCl_3$) δ 2.25 - 2.13 (m, 1H), 1.97 - 1.87 (m, 1H), 1.87 - 1.78 (m, 1H), 1.78 - 1.60 (m, 3H), 1.29 (d, J = 2.4 Hz, 6H); ^{13}C NMR (101 MHz, $CDCl_3$) δ 182.5, 80.7, 55.3, 37.3, 32.9, 25.1, 19.7, 18.0; HRMS (ESI) calcd for $C_8H_{14}NaO_3$ $[M + Na]^+$ m/z = 181.0835, found: 181.0836; IR ν_{max}/cm^{-1} (film) : 3434, 2972, 2882, 1698, 1378, 1298, 1129, 923, 818.

2-hydroxy-2-methyl-1-phenylcyclopentane-1-carboxylic acid (39a)

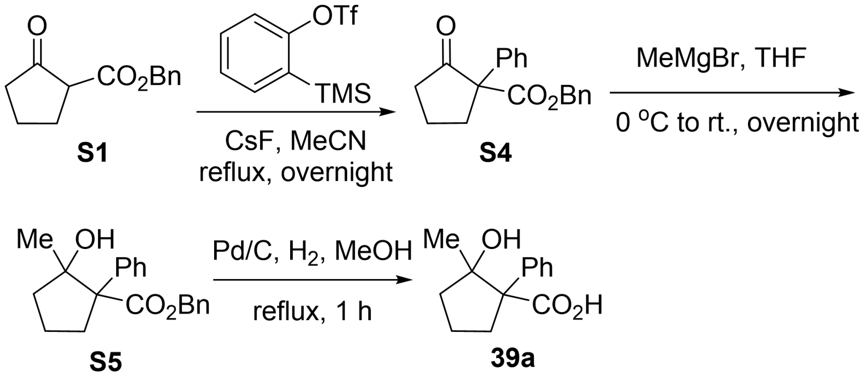

Supplementary Fig. 4. Preparation of **39a** from **S1** in three steps

The mixture of **S1** (2.18 g, 10 mmol), 2-(trimethylsilyl) phenyl trifluoromethanesulfonate (3.58 g, 12 mmol, 1.2 equiv.) and CsF (2.28 g, 15 mmol, 1.5 equiv.) in MeCN (30 mL) under N_2 was refluxed overnight. The solvent was removed in vacuum. The residue was diluted with H_2O (20 mL) and extracted by ethyl acetate (3*50 mL). The combined organic layers were washed with brine, dried over Na_2SO_4, filtered and concentrated. The residue was purified by silica gel chromatography eluting with hexane/ethyl acetate (5: 1) to provide **S4** (white solid, 677 mg, 23% yield).

To a solution of **S4** (677 mg, 2.30 mmol) in dry THF (25 mL) was added MeMgBr (3.45 mL, 3.45 mmol, 1.0 M in THF, 1.5 equiv.) by dropwise at 0 °C over 5 min and the reaction was stirred at 0 °C for 1 hour. Then the mixture was allowed to stir at room temperature overnight. The reaction mixture was quenched with sat. NH_4Cl, and then extracted with ethyl acetate (3*50 mL). The combined organic layers were washed with brine (50 mL), dried over Na_2SO_4, filtered and concentrated. The crude product was purified by silica gel chromatography eluting with hexane/ethyl acetate (5: 1) to provide **S5** as colorless oil (341 mg, 48% yield).

To a stirred solution of **S5** (341 mmol, 1.10 mmol) in degassed methanol (10 mL) was added 10% palladium on carbon (6 mg). The reaction mixture was then stirred under a hydrogen atmosphere for 1 hour then filtered through celite. The filtrate was reduced in vacuo, then the crude product was recrystallized by ethyl acetate to give **39a** as a white solid (220 mg, 95% yield).

1H NMR (400 MHz, DMSO-d_6) δ 9.30 (s, 1H), 7.51 (d, J = 7.4 Hz, 2H), 7.17 (t, J = 7.4 Hz, 2H), 7.07 (t, J = 7.8 Hz, 1H), 2.66 (t, J = 10.6 Hz, 1H), 1.91 - 1.72 (m, 2H), 1.57 (m, J = 10.8 Hz, 2H), 1.46 (m, J = 15.1, 10.7 Hz, 1H), 0.77 (s, 3H); ^{13}C NMR (101 MHz, DMSO-d_6) δ 178.9, 144.0, 127.5, 127.0, 125.1, 80.5, 60.7, 31.9, 24.9, 17.9; HRMS (ESI) calcd for $C_{13}H_{16}NaO_3$ $[M + Na]^+$ m/z = 243.0992, found: 243.0991; IR ν_{max}/cm^{-1} (film) : 3434, 3056, 2967, 2878, 1685, 1497, 1447, 1375, 1262, 722.

1-hydroxy-1,2-dimethyl-1,2,3,4-tetrahydronaphthalene-2-carboxylic acid (40a)

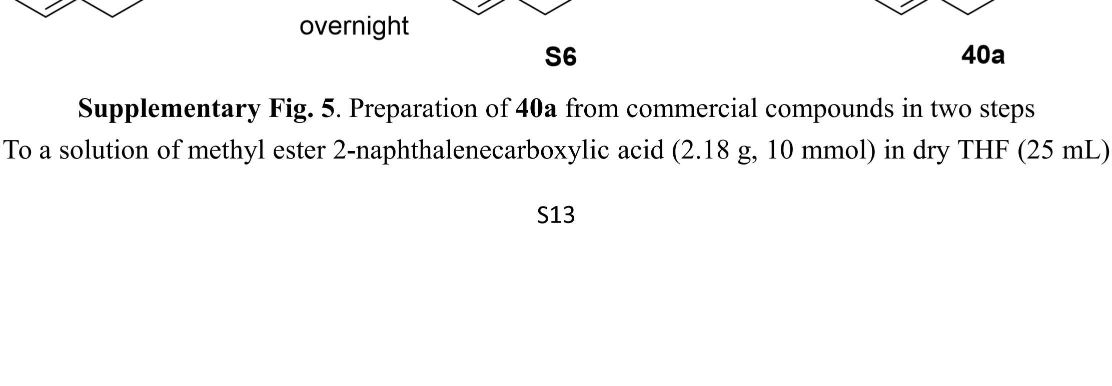

Supplementary Fig. 5. Preparation of **40a** from commercial compounds in two steps

To a solution of methyl ester 2-naphthalenecarboxylic acid (2.18 g, 10 mmol) in dry THF (25 mL)

was added MeMgBr (15 mL, 15 mmol, 1.0 M in THF, 1.5 equiv.) by dropwise at 0 °C and the reaction was stirred at 0 °C for 1 hour. Then the mixture was allowed to stir at room temperature overnight. The reaction mixture was quenched with sat. NH_4Cl and then extracted with ethyl acetate (3*50 mL). The combined organic layers were washed with brine (50 mL), dried over Na_2SO_4, filtered and concentrated. The crude product was purified by silica gel chromatography eluting with hexane/ethyl acetate (5: 1) to provide **S6** as colorless oil (1.17 g, 50% yield).

The mixture of **S6** (1.17 g, 5.0 mmol) in EtOH (20 mL) and 1 N NaOH (20 mL) was refluxed for 24 hours, cooled to room temperature, and then added 1 N HCl until pH to 5~6. The mixture was extracted by ethyl acetate (3*50 mL). The combined organic layers were washed with brine, dried over Na_2SO_4, filtered and concentrated. The crude product was recrystallized by ethyl acetate to give **40a** as a white solid (990 mg, 90% yield).

1H NMR (400 MHz, DMSO-d_6) δ 7.47 (d, J = 7.5 Hz, 1H), 7.14 - 7.00 (m, 2H), 6.97 (d, J = 7.3 Hz, 1H), 2.82 - 2.65 (m, 2H), 1.90 (dddd, J = 14.3, 10.6, 9.5, 5.8 Hz, 2H), 1.24 (s, 3H), 0.94 (s, 3H); ^{13}C NMR (101 MHz, DMSO-d_6) δ 181.8, 144.4, 133.6, 127.7, 125.5, 125.4, 125.3, 73.1, 46.4, 31.7, 28.3, 25.0, 19.3; HRMS (ESI) calcd for $C_{13}H_{16}NaO_3$ [M + Na]$^+$ m/z = 243.0992, found: 243.0996; IR ν_{max}/cm^{-1} (film) : 3400, 3021, 2932, 1702, 1455, 1212, 1037, 949, 772, 739.

1-hydroxy-2-methyl-1,2,3,4-tetrahydronaphthalene-2-carboxylic acid (41a)

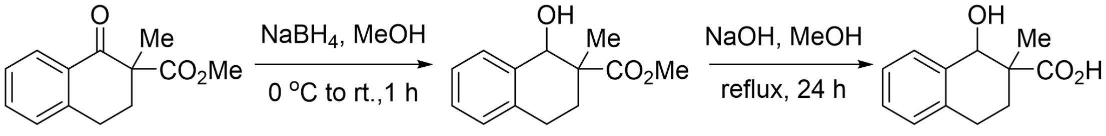

S7 **41a**

Supplementary Fig. 6. Preparation of acid **41a** from commercial compounds in two steps

To a solution of methyl ester 2-naphthalenecarboxylic acid (2.18 g, 10 mmol) in MeOH (25 mL) was added $NaBH_4$ (570 mg, 15 mmol, 1.5 equiv.) at 0 °C under N_2. The reaction was stirred at 0 °C under N_2 for 1 hour. The reaction was quenched with Na_2SO_4 and water, and then extracted with ethyl acetate (3*50 mL). The combined organic layers were washed with brine (50 mL), dried over Na_2SO_4, filtered and concentrated. The crude product was purified by silica gel chromatography eluting with hexane/ethyl acetate (5: 1) to provide **S7** as colorless oil (1.46 g, 67% yield).

The mixture of **S7** (1.46 g, 6.7 mmol) in EtOH (20 mL) and 1 N NaOH (20 mL) was refluxed for 24 hours, cooled to room temperature, and then added 1 N HCl until pH to 5~6. The mixture was extracted by ethyl acetate (3*50 mL). The combined organic layers were washed with brine, dried over Na_2SO_4, filtered and concentrated. The crude product was recrystallized by ethyl acetate to give **41a** as a white solid (1.20 g, 90% yield).

^{1}H NMR (400 MHz, $CDCl_3$) δ 7.51 - 7.45 (m, 1H), 7.25 - 7.19 (m, 2H), 7.14 - 7.08 (m, 1H), 4.63 (s, 1H), 2.94 (dt, J = 17.8, 6.4 Hz, 1H), 2.86 - 2.76 (m, 1H), 2.35 (dt, J = 13.9, 7.0 Hz, 1H), 1.92 - 1.80 (m, 1H), 1.34 (s, 3H); ^{13}C NMR (101 MHz, DMSO-d_6) δ 177.5, 137.7, 135.0, 130.4, 128.4, 127.2, 125.8, 72.3, 45.3, 24.9, 24.0,19.4; HRMS (ESI) calcd for $C_{12}H_{14}NaO_3$ [M + Na]$^+$ m/z = 229.0835, found: 229.0838; IR ν_{max}/cm^{-1} (film) : 2928, 1716, 1455, 1237, 1127, 1009, 826, 738.

1-hydroxy-2-phenyl-1,2,3,4-tetrahydronaphthalene-2-carboxylic acid (42a)

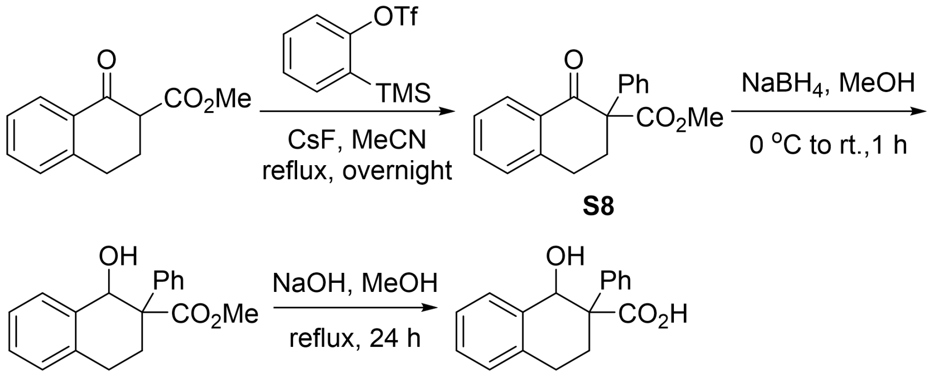

S8

S9

42a

Supplementary Fig. 7. Preparation of **42a** from commercial compounds in three steps

The mixture of methyl 1-oxo-1,2,3,4-tetrahydronaphthalene-2-carboxylate (2.04 g, 10 mmol), 2-(trimethylsilyl) phenyl trifluoromethanesulfonate (3.58 g, 12 mmol, 1.2 equiv) and CsF (2.28 g, 15 mmol, 1.5 equiv.) in MeCN (30 mL) under N_2 was refluxed overnight. The solvent was removed in vacuum. The residue was diluted with H_2O (20 mL) and extracted by ethyl acetate (3*50 mL). The combined organic layers were washed with brine, dried over Na_2SO_4, filtered and concentrated. The residue was purified by silica gel chromatography eluting with hexane/ethyl acetate (5: 1) to provide the desired intermediate **S8** as a white solid (616 mg, 22% yield).

The mixture of **S8** (616 mg, 2.2 mmol) in MeOH (25 mL) was added $NaBH_4$ (125 mg, 3.3 mmol, 1.5 equiv.) at 0 °C under N_2 and the reaction was stirred at 0 °C under N_2 for 1 hour. The reaction was quenched with Na_2SO_4 and water, and then extracted with ethyl acetate (3*50 mL). The combined organic layers were washed with brine (50 mL), dried over Na_2SO_4, filtered and concentrated. The crude product was purified by silica gel chromatography eluting with hexane/ethyl acetate (5: 1) to provide the desired intermediate **S9** as colorless oil (508 mg, 82% yield).

The mixture of **S9** (508 mg, 1.8 mmol) in EtOH (20 mL) and 1 N NaOH (20 mL) was refluxed for 24 hours, cooled to room temperature, and then added 1 N HCl until pH to 5~6. The mixture was extracted by ethyl acetate (3*50 mL). The combined organic layers were washed with brine, dried over Na_2SO_4, filtered and concentrated. The crude product was recrystallized by ethyl acetate to give **42a** as a white solid (402 mg, 83% yield).

^{1}H NMR (400 MHz, $CDCl_3$) δ 7.52 (dd, J = 5.4, 3.4 Hz, 2H), 7.40 (ddd, J = 13.5, 6.3, 4.3 Hz, 3H), 7.34 - 7.29 (m, 1H), 7.25 - 7.19 (m, 2H), 7.13 - 7.09 (m, 1H), 5.31 (s, 1H), 2.92 (t, J = 7.2 Hz, 2H), 2.54 (t, J = 6.9 Hz, 2H); ^{13}C NMR (176 MHz, $CDCl_3$) δ 178.3, 139.0, 136.3, 135.1, 129.8, 129.0, 128.8, 128.1, 127.8, 126.9, 126.4, 71.7, 54.1, 26.2, 24.3; HRMS (ESI) calcd for $C_{17}H_{16}NaO_3$ [M + Na]$^+$ m/z = 291.0992, found: 291.0992; IR ν_{max}/cm^{-1} (film) : 3399, 3060, 2925, 2854, 1705, 1493, 1264, 1186, 1080, 1002, 775, 738.

1-(tert-butoxycarbonyl)-4-hydroxy-3,4-dimethylpiperidine-3-carboxylic acid (43a)

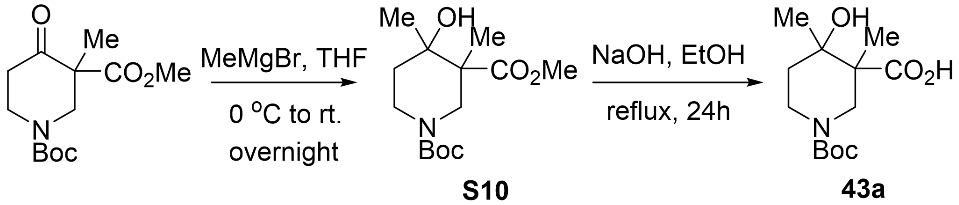

S10

43a

Supplementary Fig. 8. Preparation of **43a** from commercial compounds in two steps

To a solution of 1-(tert-butyl) 3-methyl 3-methyl-4-oxopiperidine-1,3-dicarboxylate (1.00 g, 3.69 mmol) in dry THF (25 mL) was added MeMgBr (5.54 mL, 5.54 mmol, 1.0 M in THF, 1.5 equiv.) by dropwise at 0 °C over 5 min and the reaction was stirred at 0 °C for 1 hour. Then the mixture was allowed to stir at room temperature overnight. The reaction was quenched with sat. NH_4Cl, and then extracted with ethyl acetate (3*50 mL). The combined organic layers were washed with brine (50 mL), dried over Na_2SO_4, filtered and concentrated. The crude product was purified by silica gel chromatography eluting with hexane/ethyl acetate (5: 1) to provide the desired product **S10**.

The mixture of compound **S10** in EtOH (20 mL) and 1 N NaOH (20 mL) was refluxed for 24 hours, cooled to room temperature, and then added 1 N HCl until pH to 5~6. The mixture was extracted by ethyl acetate (3*50 mL). The combined organic layers were washed with brine, dried over Na_2SO_4, filtered and concentrated. The crude product was recrystallized by ethyl acetate to give the desired product **43a** as a white solid (800 mg, 79% yield for two steps).

^{1}H NMR (400 MHz, $CDCl_3$) δ 3.95 (m, J = 21.6, 12.4 Hz, 2H), 3.13 (d, J = 14.1 Hz, 1H), 2.90 (t, J = 11.8 Hz, 1H), 1.81 (td, J = 12.8, 4.8 Hz, 1H), 1.57 (dt, J = 13.5, 3.4 Hz, 1H), 1.43 (s, 9H), 1.35 (s, 3H), 1.29 (s, 3H); ^{13}C NMR (176 MHz, $CDCl_3$) δ 179.2, 155.1, 80.3, 71.3, 49.6, 34.0, 31.4, 30.1, 28.3, 23.8, 16.8; HRMS (ESI) calcd for $C_{13}H_{23}NNaO_5$ [M + Na]$^+$ m/z = 296.1468, found: 296.1465; IR ν_{max}/cm^{-1} (film) : 2928, 2315, 1697, 1669, 1433, 1366, 1259, 1156, 972, 746.

4-(3,4-dichlorophenyl)-1-hydroxy-1,2-dimethyl-1,2,3,4-tetrahydronaphthalene-2-carboxylic acid (54a)

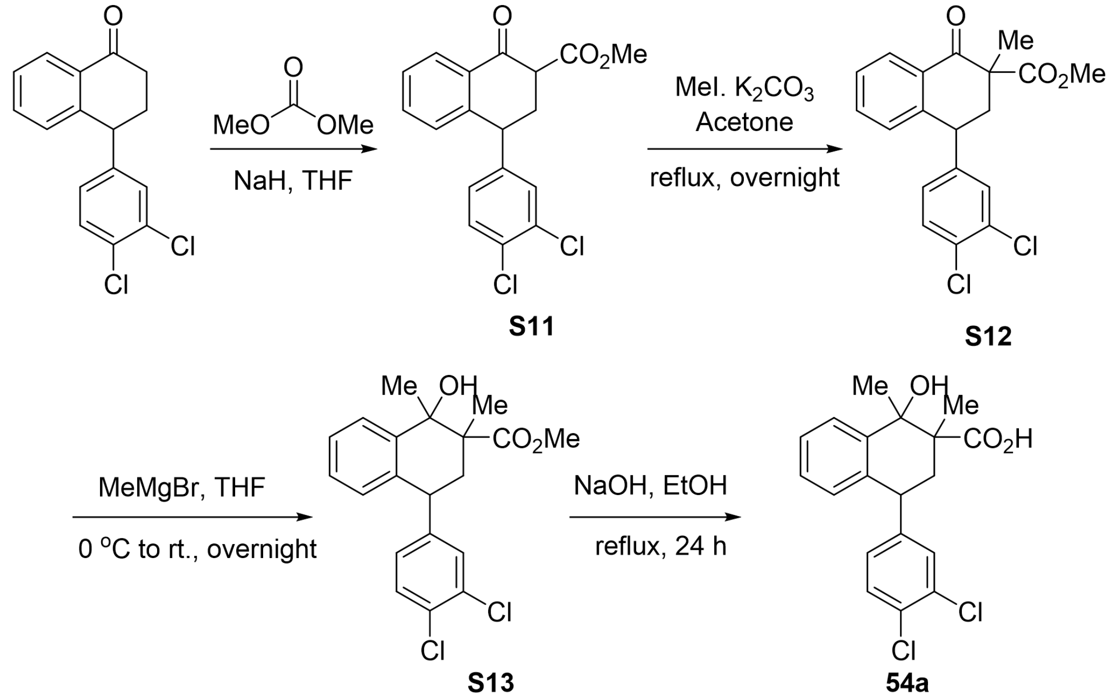

S11 S12 S13 54a

Supplementary Fig. 9. Preparation of **54a** from commercial compounds in four steps

To the solution of 4-(3,4-Dichlorophenyl)-1-tetralone (2.91 g, 10 mmol) in dry THF (20 mL) was added NaH (60% dispersion in mineral oil, 1.40 g, 35 mmol, 3.5 equiv.) at 0 °C over 5 min and the reaction was stirred under N_2 at room temperature for 1 hour. Then dimethyl carbonate (9.0 g, 100 mmol, 10 equiv.) was added into the flask by dropwise, the resulting mixture was stirred under N_2

at 70 ºC for 16 hours. Then reaction mixture was poured into ice-water and extracted with ethyl acetate (3*50 mL). The combined organic layers were washed with brine (50 mL), dried over Na_2SO_4, filtered and concentrated. The crude product was purified by silica gel chromatography eluting with hexane/ethyl acetate (5: 1) to provide the desired intermediate **S11** as colorless oil (2.27 g, 65% yield).

To the mixture of **S11** (2.27 g, 6.5 mmol) and K_2CO_3 (3.59 g, 26 mmol, 4 equiv.) in acetone (20 mL) was added MeI (1.62 mL, 26 mmol, 4 equiv.) by dropwise under N_2. The resulting mixture was stirred and refluxed overnight under N_2. The reaction was cooled to room temperature, quenched with H_2O (20 mL) and extracted with ethyl acetate (3*50 mL). The combined organic layers were washed with brine (50 mL), dried over Na_2SO_4, filtered and concentrated. The crude product was purified by silica gel chromatography eluting with hexane/ethyl acetate (5: 1) to provide the desired intermediate **S12** as a white solid (1.23 g, 52% yield).

To a solution of **S12** (1.23 g, 3.4 mmol) in dry THF (25 mL) was added MeMgBr (5.1 mL, 5.1 mmol, 1.0 M in THF, 1.5 equiv.) by dropwise at 0 ºC over 5 min and the reaction was stirred at 0 ºC for 1 hour. Then the mixture was allowed to stir at room temperature overnight. The reaction mixture was quenched with sat. NH_4Cl and then extracted with ethyl acetate (3*50 mL). The combined organic layers were washed with brine (50 mL), dried over Na_2SO_4, filtered and concentrated. The crude product was purified by silica gel chromatography eluting with hexane/ethyl acetate (5: 1) to provide **S13** as a white solid (794 mg, 62%).

The mixture of **S13** (794 mg, 2.1 mmol) in EtOH (20 mL) and 1 N NaOH (20 mL) was refluxed for 24 hours, cooled to room temperature, and then added 1 N HCl until pH to 5~6. The mixture was extracted by ethyl acetate (3*50 mL). The combined organic layers were washed with brine, dried over Na_2SO_4, filtered and concentrated. The crude product was recrystallized by ethyl acetate to give **54a** as a white solid (837 mg, 23% yield).

^{1}H NMR (400 MHz, $CDCl_3$)) δ 7.72 (d, J = 8.0 Hz, 1H), 7.38 (d, J = 8.2 Hz, 1H), 7.30 (t, J = 7.6 Hz, 1H), 7.23 (d, J = 2.1 Hz, 1H), 7.17 - 7.11 (m, 1H), 6.96 (dd, J = 8.3, 2.1 Hz, 1H), 6.76 (d, J = 7.8 Hz, 1H), 4.18 - 4.07 (m, 1H), 2.41 (dd, J = 14.6, 6.8 Hz, 1H), 2.19 (dd, J = 14.5, 12.3 Hz, 1H), 1.68 (s, 3H), 1.44 (s, 3H); ^{13}C NMR (101 MHz, $CDCl_3$) δ 181.6, 146. 6, 141.6, 134.8, 132.6, 130.7, 130.6, 129.1, 128.1, 127.5, 127.4, 126.0, 74.3, 53.4, 49.6, 41.9, 38.5, 31.1, 18.7; HRMS (ESI) calcd for $C_{19}H_{18}Cl_2NaO_3$ [M + Na]$^+$ m/z = 387.0525, found: 387.0527; IR ν_{max}/cm^{-1} (film) : 3399, 2925, 2854, 1704, 1493, 1447, 1384, 1260, 1186, 1080, 775, 699.

10,13-dimethyl-3-phenylhexadecahydro-1H-cyclopenta[a]phenanthrene-3-carboxylic acid (55a)

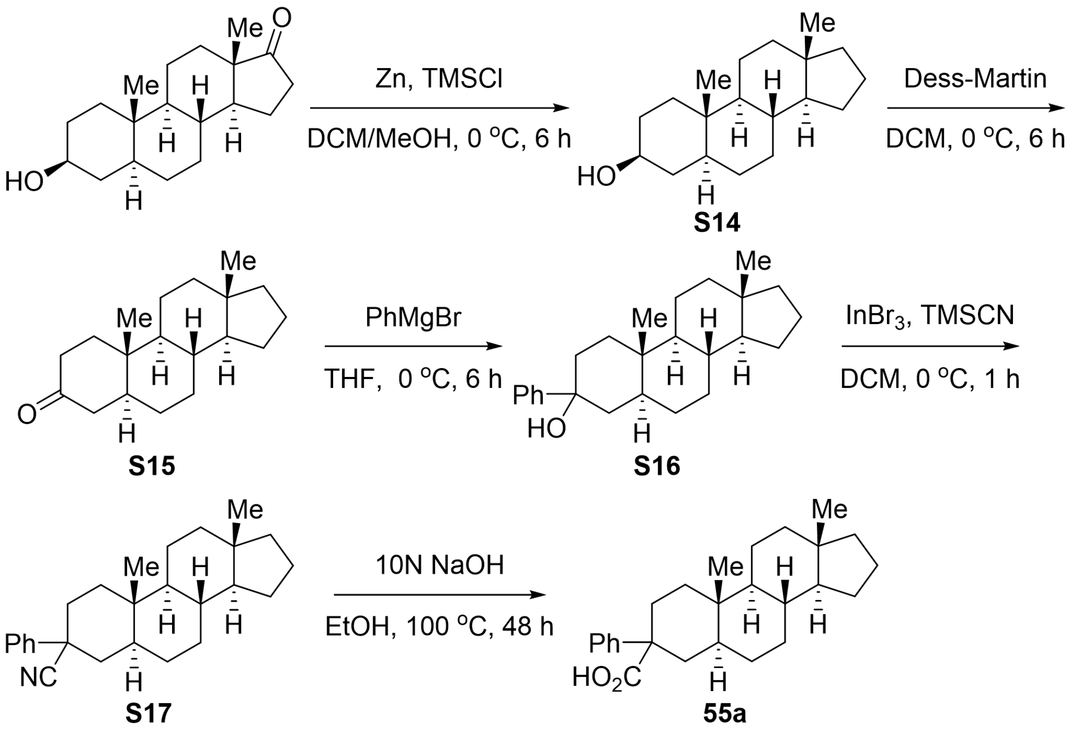

Supplementary Fig. 10. Preparation of **55a** from commercial compounds in five steps

To the mixture of 3-hydroxy-10,13-dimethylhexadecahydro-17H-cyclopenta[a]phenanthren-17-one (2.0 g, 6.9 mmol) and Zn powder (14,0 g, 131.1mmol, 19 equiv.) in 100 mL DCM/MeOH (V/V=1:1) was added TMSCl (28 mL, 224.3 mmol, 32.5 equiv.) by dropwise with ice bath. The resulting mixture was stirred at room temperature overnight. The mixture was filtered the solid, and then removed the solvent in vacuum. The residue was extracted with EA (3*50 mL), and then concentrated. The crude product (**S14,** 1.7 g, 6.1 mmol, 93% yield) was obtained.

To the solution of the **S14** (1.7 g, 6.1 mmol,) in DCM was added Dess-Martin periodinane (2.8 g, 6.7 mmol, 1.1 equiv.), the resulting mixture was stirred at room temperature for 6 hours. The mixture was filtered the solid, and then removed the solvent in vacuum. The residue was extracted with EA (3*50 mL), and then concentrated. The crude product (**S15**, 1.5 g, 5.5 mmol, 90% yield) was obtained.

To a solution of **S15** (1.5 g, 5.5 mmol) in dry THF (25 mL) was added PhMgBr (3.2 mL, 8.3 mmol, 2.6 M in THF, 1.5 equiv.) by dropwise at 0 °C over 5 min and the reaction was stirred at 0 °C for 1 hour. Then the mixture was allowed to stir at room temperature overnight. The reaction mixture was quenched with sat. NH_4Cl and then extracted with ethyl acetate (3*50 mL). The combined organic layers were washed with brine (50 mL), dried over Na_2SO_4, filtered and concentrated. The crude product was purified by silica gel chromatography eluting with hexane/ethyl acetate (5: 1) to provide **S16** as a white solid (1.06 g, 3.0 mmol, 55% yield).

The solution of **S16** (1.06 g, 3.0 mmol) in DCM was added dropwise into the mixture of TMSCN (600 mg, 6.0 mmol, 2 equiv) and $InBr_3$ (105 mg, 0.3 mmol, 0.1 equiv) in DCM under N_2 at 0 °C. The resulting mixture was stirred for 1 hour. Removed the solvent, after the dryness and concentration, the residue was purified by chromatography with hexane/ethyl acetate (10: 1) to provide **S17** as a white solid (723 mg, 2.0 mmol, 67% yield).

The mixture of **S17** (723 mg, 2.0 mmol) in EtOH (20 mL) and 10 N NaOH (20 mL) was refluxed for 48 hours, cooled to room temperature, and then added 1 N HCl until pH to 5~6. The mixture

was extracted by ethyl acetate (3*50 mL). The combined organic layers were washed with brine, dried over Na_2SO_4, filtered and concentrated. The crude product was purified by chromatography with hexane/ethyl acetate (10: 1) to provide **55a** as a white solid (200 mg, 0.53 mmol, 27% yield). ^{1}H NMR (400 MHz, $CDCl_3$) δ 7.49 - 7.42 (m, 2H), 7.38 - 7.32 (m, 2H), 7.27 - 7.23 (m, 1H), 2.51 (dd, J = 14.6, 3.2 Hz, 1H), 2.18 (dt, J = 14.3, 2.8 Hz, 1H), 2.00 (td, J = 14.5, 3.7 Hz, 1H), 1.87 (dd, J = 14.3, 12.8 Hz, 1H), 1.66 - 1.54 (m, 5H), 1.52 - 1.33 (m, 3H), 1.30 - 1.15 (m, 5H), 1.14-0.96 (m, 4H), 0.84 (s, 3H), 0.82 - 0.71 (m, 2H), 0.65 (s, 3H), 0.49 (ddd, J = 14.9, 10.9, 4.1 Hz, 1H); ^{13}C NMR (101 MHz, $CDCl_3$) δ 181.5, 139.4, 128.6, 127.6, 126.8, 54.4, 54.3, 49.6, 41.3, 40.8, 40.4, 38.8, 36.0, 35.7, 34.8, 34.4, 32.1, 28.6, 27.7, 25.4, 20.9, 20.4, 17.5, 12.4; HRMS (ESI) calcd for $C_{26}H_{36}NaO_2$ $[M + Na]^+$ m/z = 403.2608, found: 403.2624; IR ν_{max}/cm^{-1} (film) : 3445, 2934, 2856, 2026, 1699, 1631, 1615, 1355, 1276, 722, 697, 667.

10,13-dimethyl-17-(6-methylheptan-2-yl)-3-phenylhexadecahydro-1H-cyclopenta[a]phenanthrene-3-carboxylic acid (56a)

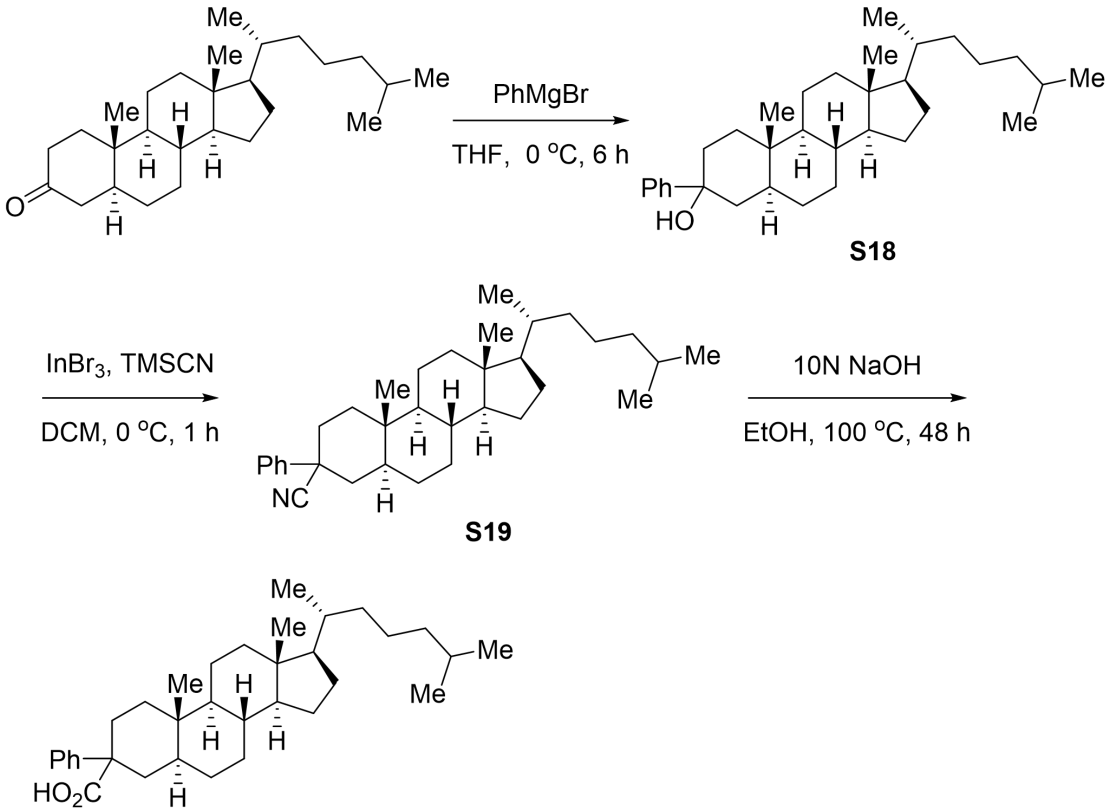

56a

Supplementary Fig. 11. Preparation of **56a** from commercial compounds in three steps

To a solution of 5α-cholestan-3-one (3.9 g, 10.0 mmol) in dry THF (50 mL) was added PhMgBr (5.8 mL, 15 mmol, 2.6 M in THF, 1.5 equiv.) by dropwise at 0 °C over 5 min and the reaction was stirred at 0 °C for 1 hour. Then the mixture was allowed to stir at room temperature overnight. The reaction mixture was quenched with sat. NH_4Cl and then extracted with ethyl acetate (3*100 mL). The combined organic layers were washed with brine (50 mL), dried over Na_2SO_4, filtered and concentrated. The crude product was purified by silica gel chromatography eluting with hexane/ethyl acetate (5: 1) to provide **S18** as a white solid (2.46 g, 5.3 mmol, 53% yield).

The solution of **S18** (2.40 g, 5.2 mmol) in DCM was added by dropwise into the flask with the

mixture of TMSCN (1.0 g, 10.4 mmol, 2 equiv) and $InBr_3$ (185 mg, 0.52 mmol, 0.1 equiv) in DCM under N_2 at 0 °C. The resulting mixture was stirred for 1 hour. Removed the solvent, after the dryness and concentration, the residue was purified by chromatography with hexane/ethyl acetate (10: 1) to provide **S18** as a white solid (1.48 g, 3.1 mmol, 60% yield).

The mixture of **S18** (1.4 g, 3.0 mmol) in EtOH (20 mL) and 10 N NaOH (20 mL) was refluxed for 48 hours, cooled to room temperature, and then added 1 N HCl until pH to 5~6. The mixture was extracted by ethyl acetate (3*50 mL). The combined organic layers were washed with brine, dried over Na_2SO_4, filtered and concentrated. The crude product was purified by chromatography with hexane/ethyl acetate (5: 1) to provide **56a** as a white solid (369 mg, 0.75 mmol, 25% yield).

^1H NMR (400 MHz, $CDCl_3$) δ 7.45 (d, J = 7.8 Hz, 2H), 7.33 (t, J = 7.6 Hz, 2H), 7.28 - 7.23 (m, 1H), 2.49 (d, J = 14.5 Hz, 1H), 2.18 - 2.13 (m, 1H), 1.98 (d, J = 3.3 Hz, 1H), 1.86 (t, J = 13.6 Hz, 2H), 1.73 (dt, J = 15.4, 7.8 Hz, 2H), 1.61 - 1.41 (m, 5H), 1.35 - 1.19 (m, 9H), 1.07 (d, J = 6.8 Hz, 4H), 1.00 - 0.92 (m, 4H), 0.83 (m, 12H), 0.78 - 0.71 (m, 1H), 0.58 (s, 3H), 0.47 - 0.40 (m, 1H); ^{13}C NMR (176 MHz, $CDCl_3$) δ 180.8, 139.5, 128.6, 127.6, 126.8, 56.4, 56.3, 54.0, 49.6, 42.5, 41.3, 39.9, 39.5, 36.2, 35.9, 35.8, 35.3, 34.9, 34.3, 31.8, 28.5, 28.2, 28.0, 27.7, 24.1, 23.9, 22.8, 22.6, 20.9 18.6, 12.4, 12.0; HRMS (ESI) calcd for $C_{34}H_{52}NaO_2$ [M + Na] $^+$ m/z = 515.3860, found: 515.3977; IR ν_{max}/cm^{-1} (film) : 3465, 2929, 2026, 1637, 1356, 1260, 1077, 773, 696, 667, 606.

1.3 Supplementary Reaction Optimization and Control Experiment

General procedure: To a 25 mL round bottomed flask equipped with a magnetic stirrer bar were added acid (0.5 mmol, 1.0 equiv.), photocatalyst (0.015 mmol, 0.03 equiv.), copper (0.1 mmol, 0.2 equiv.), ligand (0.125 mmol, 0.25 equiv.), base (0.75 mmol, 1.5 equiv.) and DCM (10 mL). The flask was quickly degassed three times and flushed with oxygen through balloon, and then the mixture was irradiated with two 45 W blue LEDs (5 cm away) at 15 ºC for 40 hours. The reaction mixture was filtered and concentrated. The residue was purified by column chromatography on silica with petroleum ether/ethyl acetate as eluent.

(a) Optimization tables of reaction conditions

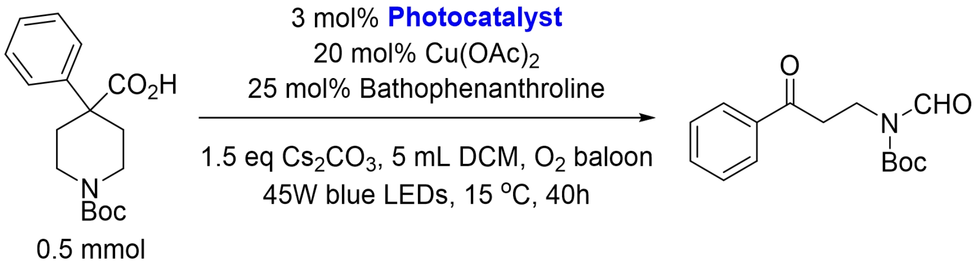

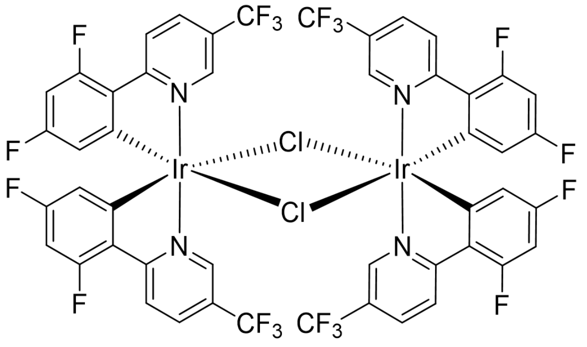

[(dF(CF$_3$)ppy)$_2$-Ir-μ-Cl]$_2$
(**Ir-1**) **48%**

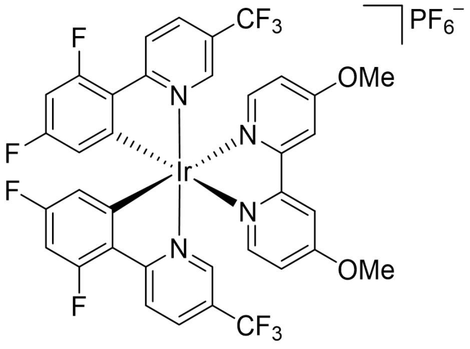

[Ir(dF(CF$_3$)ppy)$_2$(dOMebpy)]PF$_6$
(**Ir-2**) 47%

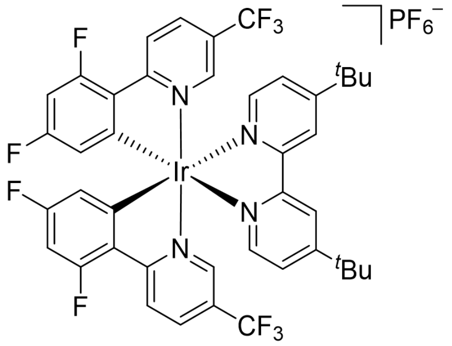

[Ir(dF(CF$_3$)ppy)$_2$(dtbpy)]PF$_6$
(**Ir-3**) 40%

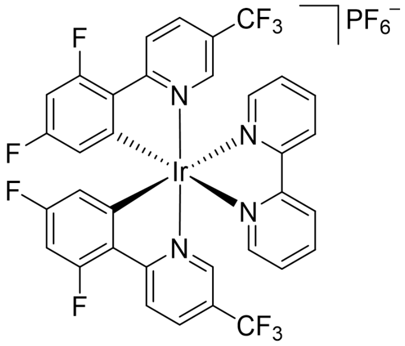

[Ir(dF(CF$_3$)ppy)$_2$(bpy)]PF$_6$
(**Ir-4**) 43%

Supplementary Fig. 12. Evaluation of different photocatalysts.

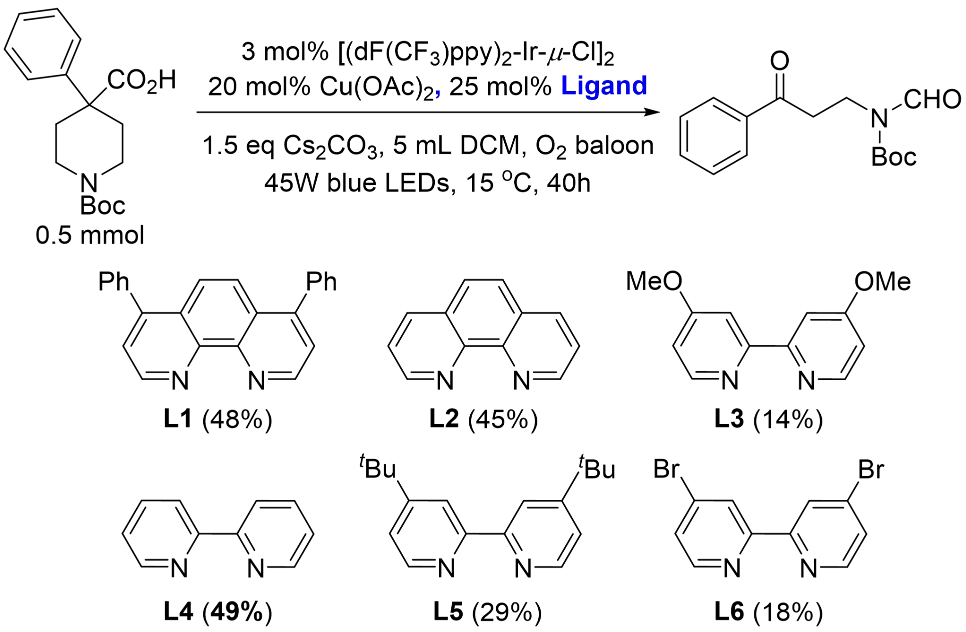

Supplementary Fig. 13. Evaluation of different ligands.

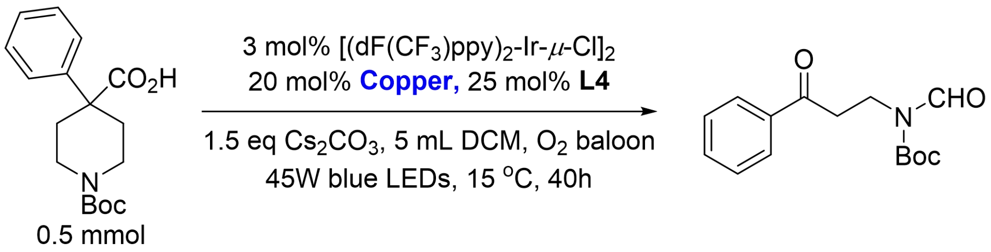

Copper	Yield
CuO	30%
$CuBr_2$	19%
CuCN	6%
**$Cu(OAc)_2$**	**49%**

Supplementary Fig. 14. Evaluation of different coppers.

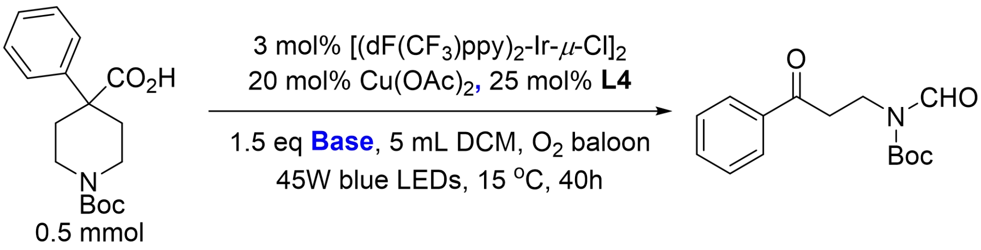

Base (equiv.)	Yield
K_2CO_3 (1.5)	42%
TEA (1.5)	12%
**Cs_2CO_3 (1.5)**	**49%**
Cs_2CO_3 (1.0)	30%
Cs_2CO_3 (2.0)	50%
Cs_2CO_3 (3.0)	20%

Supplementary Fig. 15. Evaluation of different bases.

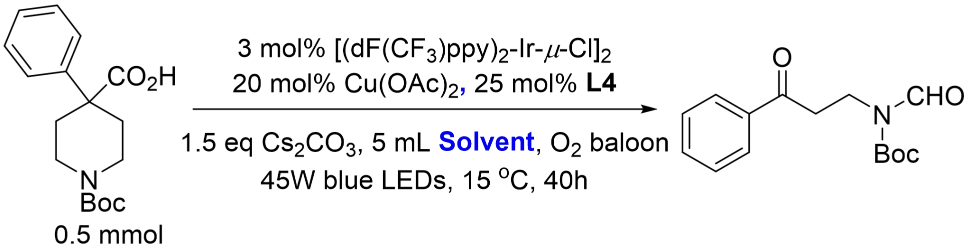

Solvent	Yield
MeCN	26%
1,4-dioxane	16%
DMF	0%
**DCM**	**49%**

Supplementary Fig. 16. Evaluation of different solvents.

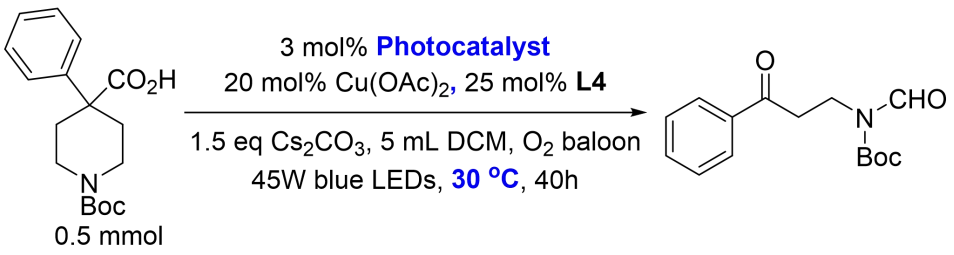

Photocatalyst	Yield
**Ir-1**	**81%**
**Ir-2**	48%
**Ir-3**	60%
**Ir-4**	50%

Supplementary Fig. 17. Evaluation of different photocatalysts at 30 °C.

(b) Control Experiments

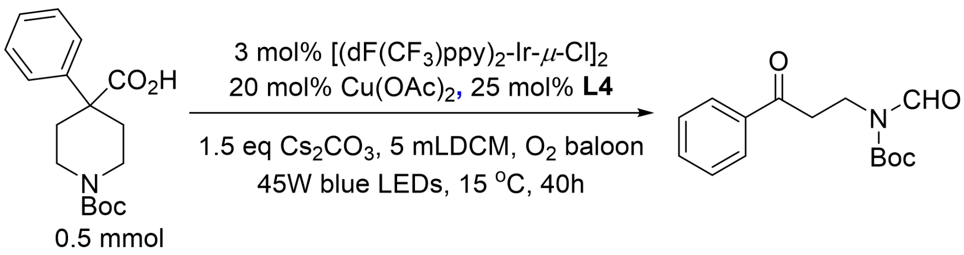

Condition	Yield
no photocatalyst	0%
no copper, no ligand	<5%
no ligand	26%
no light	0%
no base	0%
no copper	6%
no copper, 5 mol% **L4**	1%

Supplementary Fig. 18. Control reactions. Yields determined by ^{1}H NMR analysis.

(c) Evaluation of additive

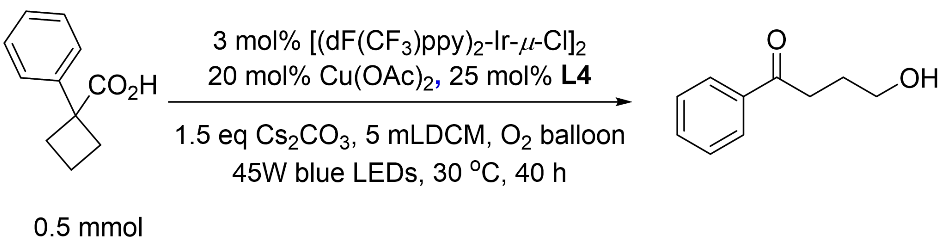

**Additive (1.5 eq.)**	Yield
Tetrabutylammonium iodide	30%
Tetrabutylammonium bromide	10%
NH_4Cl	35%
TEA	40%
Selectfluor	75%

Supplementary Fig. 19. Evaluation of additive.

2 Supplementary Discussion

(a)

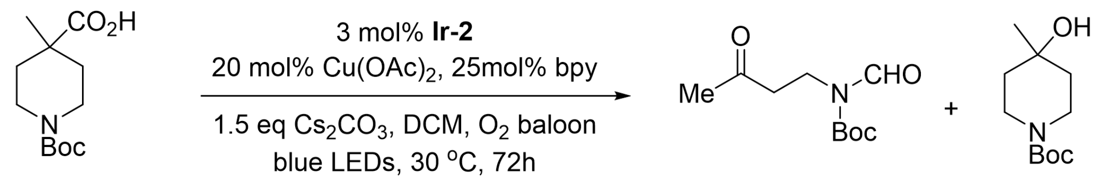

15a **15b**, 54% **15c**, 31%

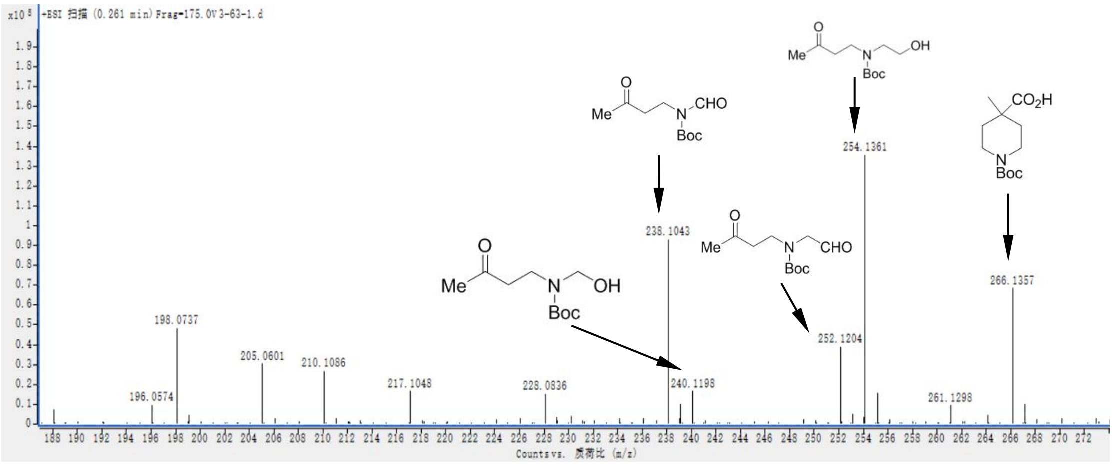

This experiment was executed as follows: to a 25 mL round-bottom flask equipped with a stir bar was added **15a** (123 mg, 0.50 mmol), [Ir(dF(CF_3)ppy)$_2$(dOMebpy)]PF_6 (16.7 mg, 0.015 mmol, 0.03 equiv.), $Cu(OAc)_2$ (18.1 mg, 0.1 mmol, 0.2 equiv.), 2,2'-bipyridine (19.5 mg, 0.125 mmol, 0.25 equiv.), Cs_2CO_3 (245 mg, 0.75 mmol, 1.5 equiv.) and DCM (10 mL). The flask was quickly degassed three times and flushed with oxygen through balloon, and then the mixture was heated to 30 °C in an oil bath and irradiated with two 45 W blue LEDs (5 cm away) for 24 hours. The reaction was monitor by LC-MS. Byproduct and reaction intermediates can be detected in mass spectrometry. After 72 hours, the reaction mixture was filtered, concentrated and purified by column chromatography on silica.

(b)

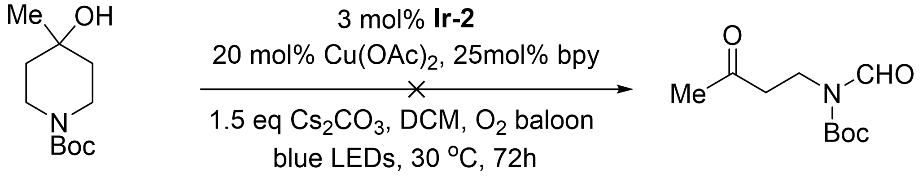

15c **15b**

This experiment was executed as follows: to a 25 mL round-bottom flask equipped with a stir bar was added **15c** (107 mg, 0.50 mmol), [Ir(dF(CF_3)ppy)$_2$(dOMebpy)]PF_6 (16.7 mg, 0.015 mmol, 0.03 equiv.), $Cu(OAc)_2$ (18.1 mg, 0.1 mmol, 0.2 equiv.), 2,2'-bipyridine (19.5 mg, 0.125 mmol, 0.25 equiv.), Cs_2CO_3 (245 mg, 0.75 mmol, 1.5 equiv.) and DCM (10 mL). The flask was quickly degassed three times and flushed with oxygen through balloon, and then the mixture was heated to

30 °C in an oil bath and irradiated with two 45 W blue LEDs (5 cm away) for 72 hours. The reaction was monitor by LC-MS and no reaction happened.

(c)

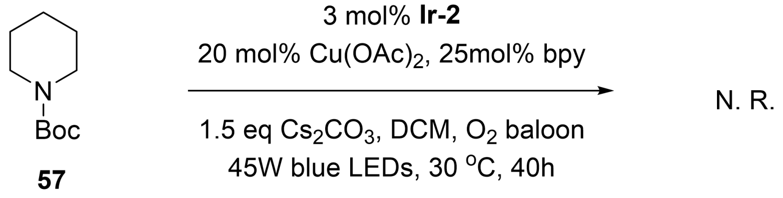

This experiment was executed as follows: to a 25 mL round-bottom flask equipped with a stir bar was added **57** (123 mg, 0.50 mmol), [Ir(dF(CF$_3$)ppy)$_2$(dOMebpy)]PF$_6$ (16.7 mg, 0.015 mmol, 0.03 equiv.), Cu(OAc)$_2$ (18.1 mg, 0.1 mmol, 0.2 equiv.), 2,2'-bipyridine (19.5 mg, 0.125 mmol, 0.25 equiv.), Cs$_2$CO$_3$ (245 mg, 0.75 mmol, 1.5 equiv.) and DCM (10 mL). The flask was quickly degassed three times and flushed with oxygen through balloon, and then the mixture was heated to 30 °C in an oil bath and irradiated with two 45 W blue LEDs (5 cm away) for 40 hours. The reaction was monitor by LC-MS and no reaction happened.

(d)

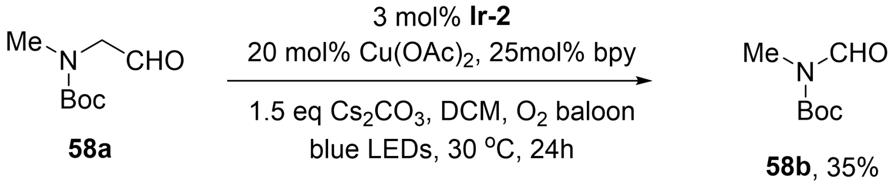

This experiment was executed as follows: to a 25 mL round-bottom flask equipped with a stir bar was added **58a** (123 mg, 0.50 mmol), [Ir(dF(CF$_3$)ppy)$_2$(dOMebpy)]PF$_6$ (16.7 mg, 0.015 mmol, 0.03 equiv.), Cu(OAc)$_2$ (18.1 mg, 0.1 mmol, 0.2 equiv.), 2,2'-bipyridine (19.5 mg, 0.125 mmol, 0.25 equiv.), Cs$_2$CO$_3$ (245 mg, 0.75 mmol, 1.5 equiv.) and DCM (10 mL). The flask was quickly degassed three times and flushed with oxygen through balloon, and then the mixture was heated to 30 °C in an oil bath and irradiated with two 45 W blue LEDs (5 cm away) for 24 hours. The reaction mixture was filtered, concentrated and purified by column chromatography on silica. **58b** was obtained in 35% yield.

(e)

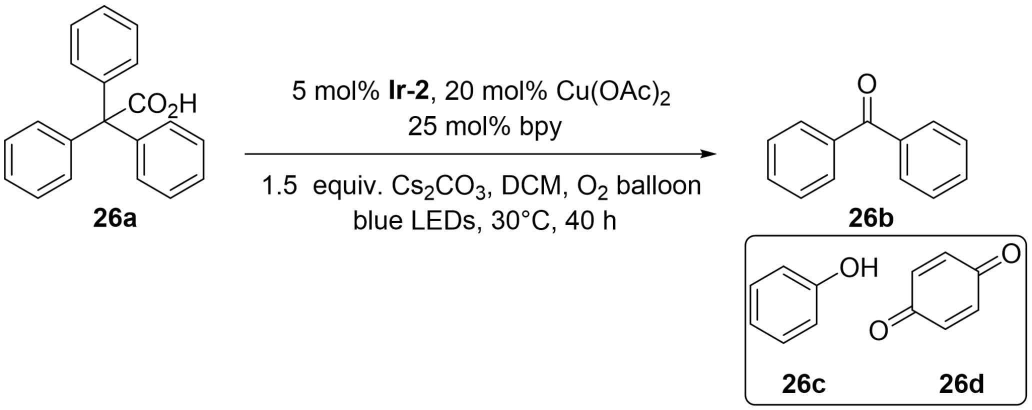

detected by GC-MS

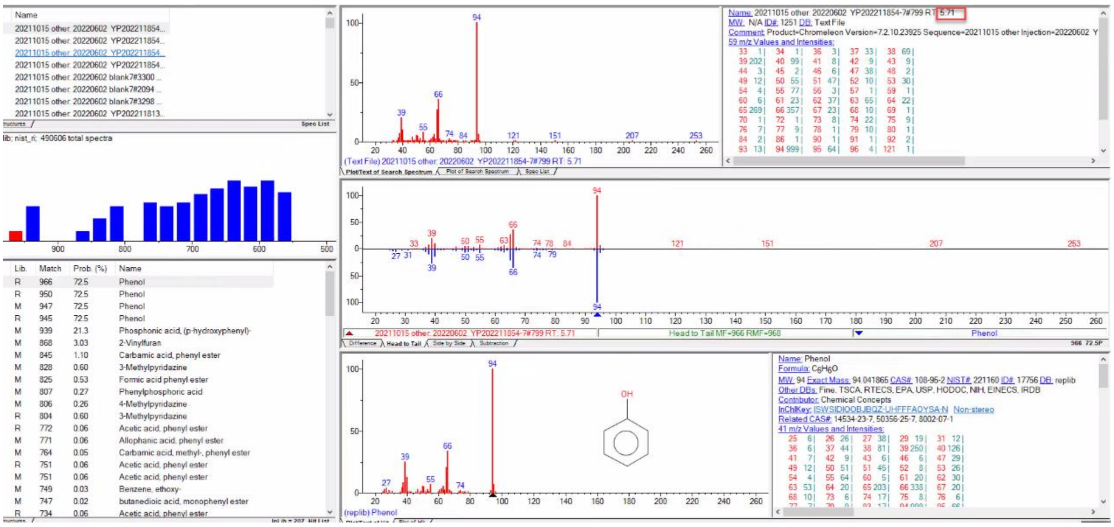

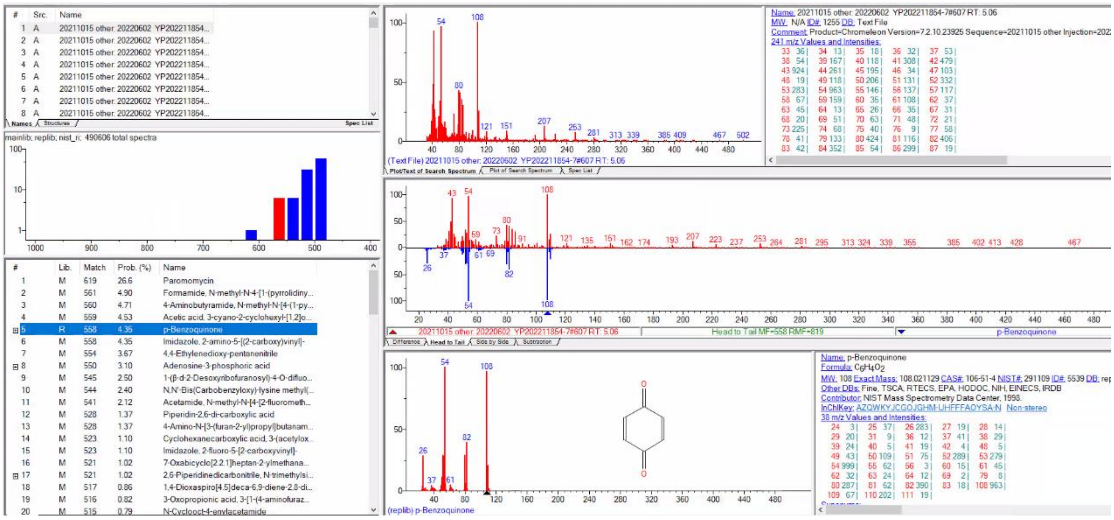

This experiment was executed as follows: to a 25 mL round-bottom flask equipped with a stir bar was added **26a** (49 mg, 0.50 mmol), [Ir(dF(CF$_3$)ppy)$_2$(dOMebpy)]PF$_6$ (16.7 mg, 0.015 mmol, 0.03 equiv.), Cu(OAc)$_2$ (18.1 mg, 0.1 mmol, 0.2 equiv.), 2,2'-bipyridine (19.5 mg, 0.125 mmol, 0.25 equiv.), Cs$_2$CO$_3$ (245 mg, 0.75 mmol, 1.5 equiv.) and DCM (10 mL). The flask was quickly degassed three times and flushed with oxygen through balloon, and then the mixture was heated to 30 °C in an oil bath and irradiated with two 45 W blue LEDs (5 cm away) for 24 hours. After 72 hours, the reaction was detected by GC-MS. **26c** and **26d** can be detected GC-MS. Then, the

reaction mixture was filtered, concentrated and purified by column chromatography on silica. **26b** were obtained.

(f)

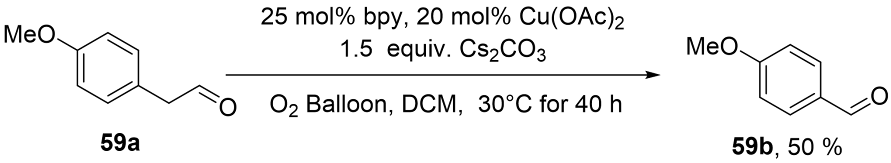

This experiment was executed as follows: to a 25 mL round-bottom flask equipped with a stir bar was added **59a** (0.20 mmol), $Cu(OAc)_2$ (18.1 mg, 0.1 mmol, 0.2 equiv.), 2,2'-bipyridine (19.5 mg, 0.125 mmol, 0.25 equiv.), Cs_2CO_3 (245 mg, 0.75 mmol, 1.5 equiv.) and DCM (10 mL). The flask was quickly degassed three times and flushed with oxygen through balloon, and then the mixture was heated to 30 °C in an oil bath for 40 hours. After 40 hours, the reaction mixture was filtered, concentrated and purified by column chromatography on silica. **59b** was obtained in 50% yield.

(g)

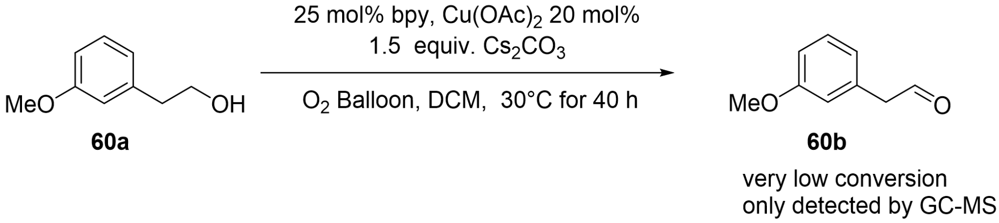

This experiment was executed as follows: to a 25 mL round-bottom flask equipped with a stir bar was added **60a** (0.20 mmol), $Cu(OAc)_2$ (18.1 mg, 0.1 mmol, 0.2 equiv.), 2,2'-bipyridine (19.5 mg, 0.125 mmol, 0.25 equiv.), Cs_2CO_3 (245 mg, 0.75 mmol, 1.5 equiv.) and DCM (10 mL). The flask was quickly degassed three times and flushed with oxygen through balloon, and then the mixture was heated to 30 °C in an oil bath for 40 hours. After 40 hours, the reaction was monitored by GC-MS. From GC-MS, **60b** was detected. However, the conversion was low and we could not isolate **60b.**

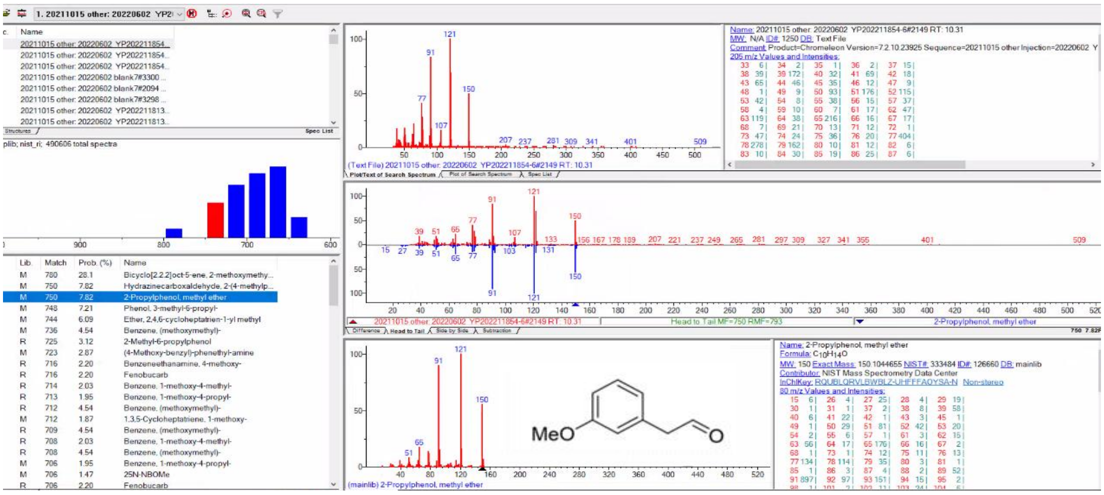

(h)

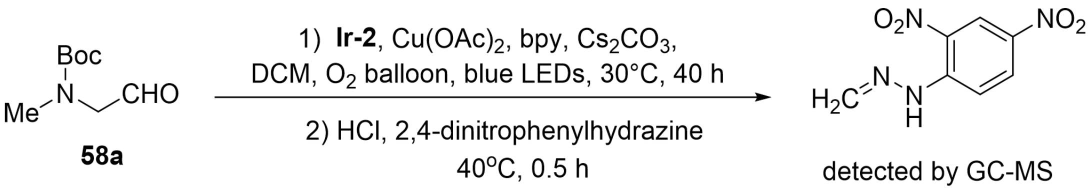

This experiment was executed as follows: to a 25 mL round-bottom flask equipped with a stir bar was added **58a** (0.50 mmol), [Ir(dF(CF$_3$)ppy)$_2$(dOMebpy)]PF$_6$ (16.7 mg, 0.015 mmol, 0.03 equiv.), Cu(OAc)$_2$ (18.1 mg, 0.1 mmol, 0.2 equiv.), 2,2'-bipyridine (19.5 mg, 0.125 mmol, 0.25 equiv.), Cs$_2$CO$_3$ (245 mg, 0.75 mmol, 1.5 equiv.) and DCM (10 mL). The flask was quickly degassed three times and flushed with oxygen through balloon, and then the mixture was heated to 30 °C in an oil bath for 40 hours. The reaction mixture was filtered, then HCl (2.5 mL) and 2,4-dinitrophenylhydrazine (0.2 mmol) were added into the organic residue. The resulting mixture was stirred at 40 °C for 0.5 h. The reaction was monitored by GC-MS, and 1-(2,4-dinitrophenyl)-2-methylenehydrazine was detected by GC-MS.

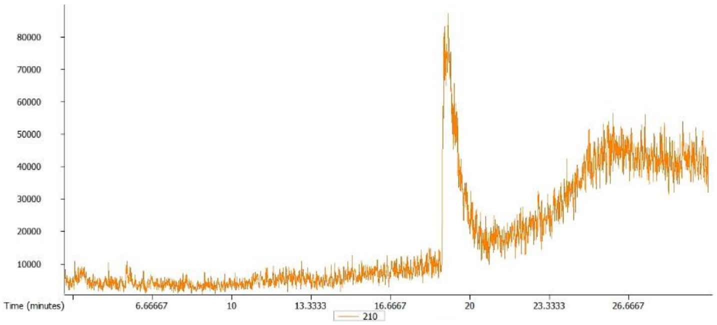

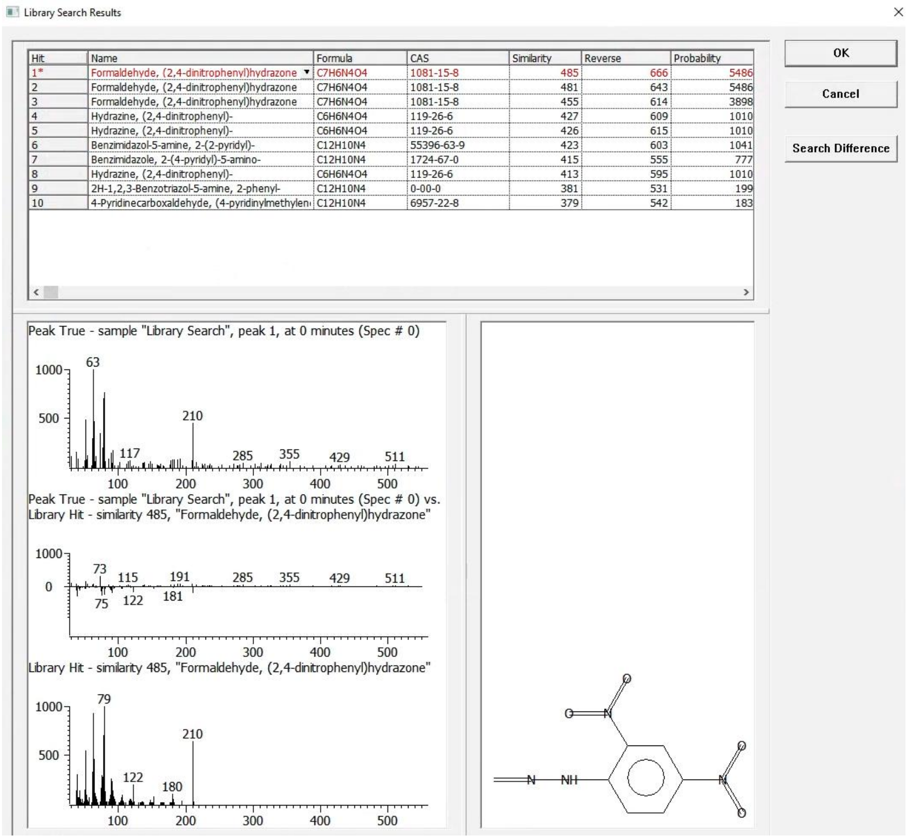

Hit	Name	Formula	CAS	Similarity	Reverse	Probability
1*	Formaldehyde, (2,4-dinitrophenyl)hydrazone	C7H6N4O4	1081-15-8	485	666	5486
2	Formaldehyde, (2,4-dinitrophenyl)hydrazone	C7H6N4O4	1081-15-8	481	643	5486
3	Formaldehyde, (2,4-dinitrophenyl)hydrazone	C7H6N4O4	1081-15-8	455	614	3898
4	Hydrazine, (2,4-dinitrophenyl)-	C6H6N4O4	119-26-6	427	609	1010
5	Hydrazine, (2,4-dinitrophenyl)-	C6H6N4O4	119-26-6	426	615	1010
6	Benzimidazol-5-amine, 2-(2-pyridyl)-	C12H10N4	55396-63-9	423	603	1041
7	Benzimidazole, 2-(4-pyridyl)-5-amino-	C12H10N4	1724-67-0	415	555	777
8	Hydrazine, (2,4-dinitrophenyl)-	C6H6N4O4	119-26-6	413	595	1010
9	2H-1,2,3-Benzotriazol-5-amine, 2-phenyl-	C12H10N4	0-00-0	381	531	199
10	4-Pyridinecarboxaldehyde, (4-pyridinylmethylen	C12H10N4	6957-22-8	379	542	183

From these control experiments, we could conclude:

11c is a by-product, not a reacting intermediate;

Photocatalytic decarboxylation was the initial step and the key to induce the consecutive C-C bond cleavage;

Copper, base and O_2 are responsible for the dehomologation reaction of aldehyde.

Formaldehyde was formed during the reaction.

(i) Stern-Volmer luminescence quenching experiments

General procedure: To a solution of **Ir-1** ([(dF(CF_3)ppy)$_2$-Ir-μ-Cl]$_2$) in DMSO was added appropriate amount of quencher in a quartz cuvette under O_2 atmosphere. Then the emission of the sample was collected. Luminescence spectra was obtained with the detection wavelength and the luminescence emission spectrum was excited at 425 nm by using FL6500. After the acquisition, the data were plotted according to the Stern - Volmer equation4 shown below.

$$I_0/I = 1 + KSV[Q]$$

Where I_0 is the luminescence intensity in the absence of the quencher, I is the intensity in the presence of the quencher, KSV is the Stern - Volmer constant, and [Q] is the concentration of the quencher.

All fluorescence measurements were performed at 24 °C in a quartz cuvette (d = 10 x 4 mm) with an excitation wavelength of 425 nm and an excitation and emission bandwidth of 20 nm. The scan speed was set at 1200 nm/min and the PMT voltage was set to 20 V. All samples were measured in DMSO with sat. Cs_2CO_3, and degassed by sparging with oxygen for 5 min, unless otherwise noted.

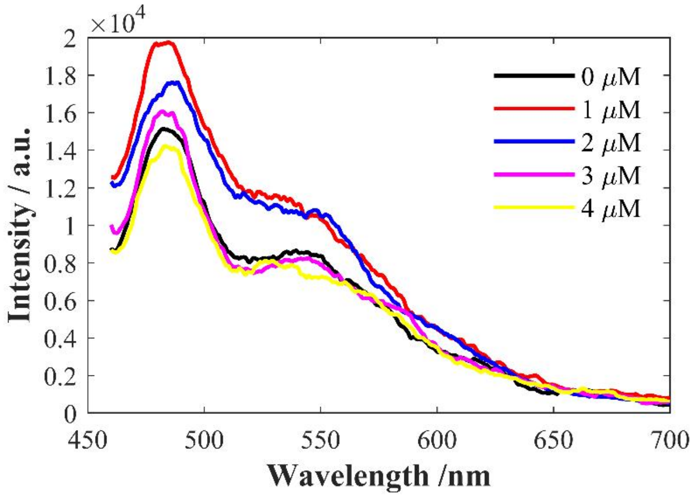

Supplementary Fig. 20. The luminescence spectra of **Ir-1** (1 μM) in DMSO without Cs_2CO_3 and O_2, with **1a** (0-4 μM).

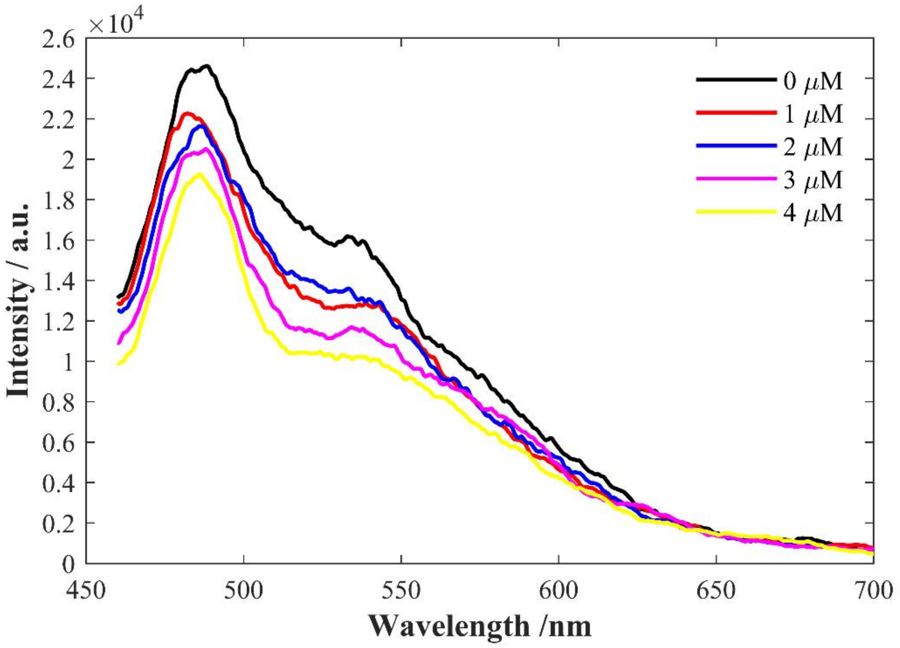

Supplementary Fig. 21. The luminescence spectra of **Ir-1** (1 μM) in DMSO with sat. Cs_2CO_3 but no O_2, with **1a** (0-4 μM).

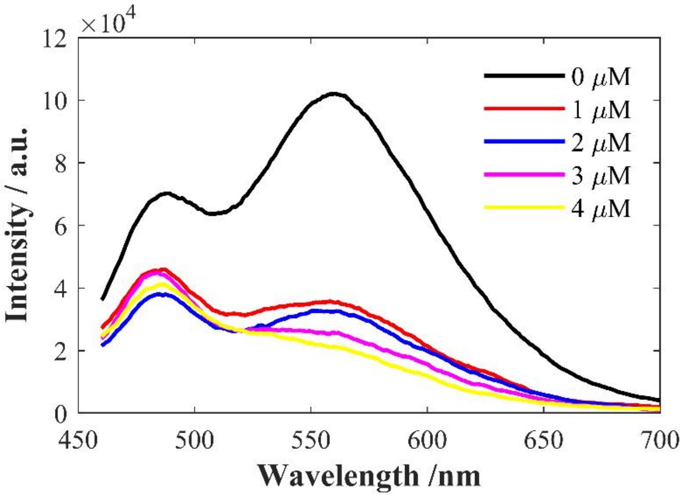

Supplementary Fig. 22. The luminescence spectra of **Ir-1** (1 μM) with **1a** (0-4 μM) in DMSO with sat. Cs_2CO_3 and O_2.

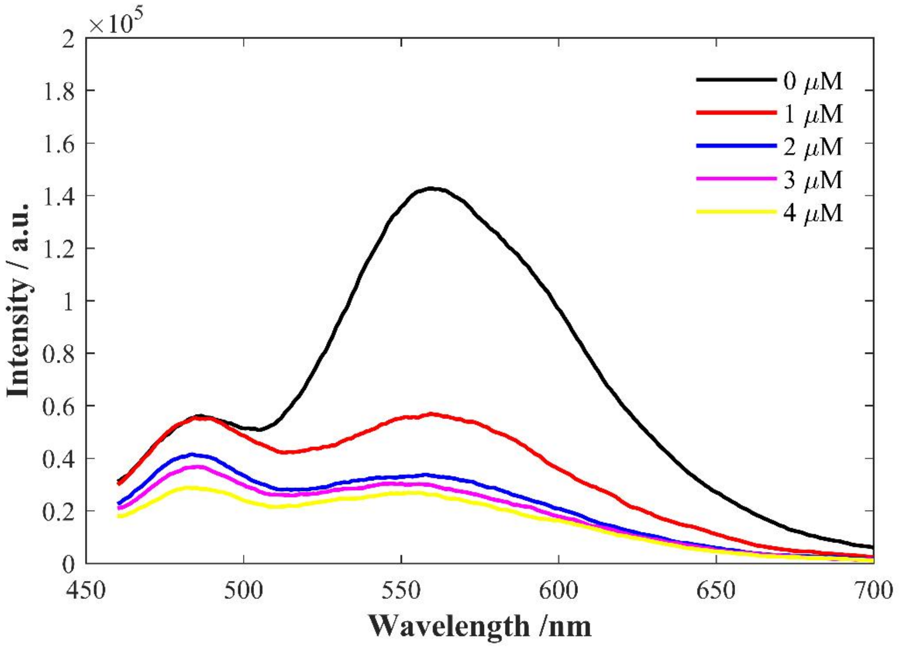

Supplementary Fig. 23. The luminescence spectra of **Ir-1** (1 μM) with Selectfluor (0-4 μM) in DMSO with sat. Cs_2CO_3 and O_2.

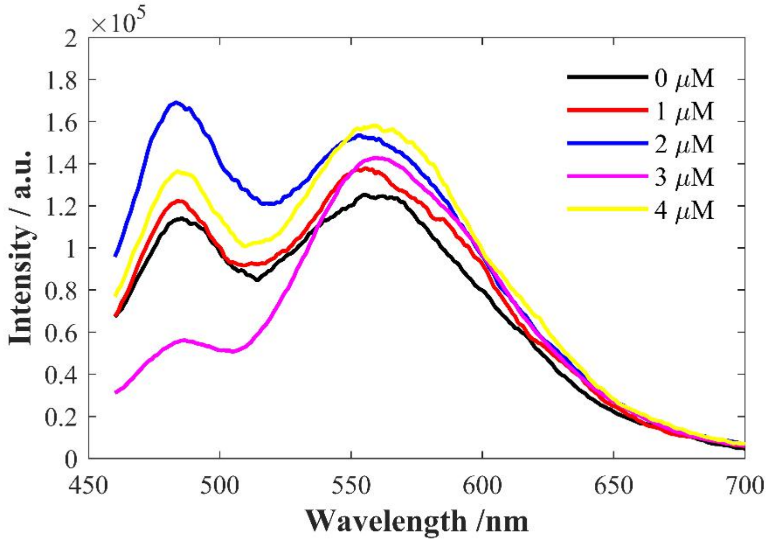

Supplementary Fig. 24. The luminescence spectra of **Ir-1** (1 μM) with **L4** (0-4 μM) in DMSO with sat. Cs_2CO_3 and O_2.

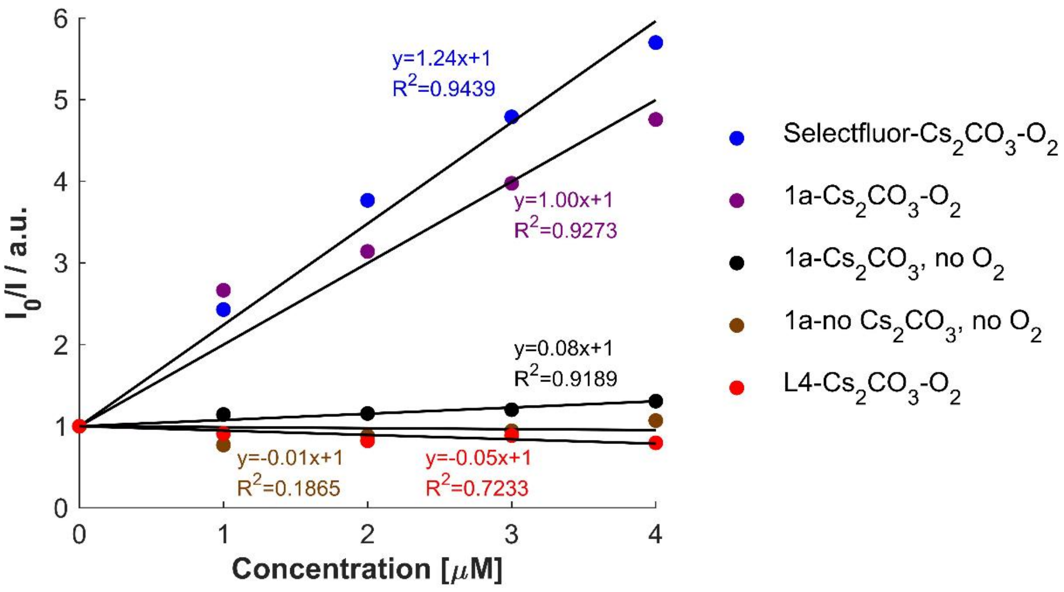

Supplementary Fig. 25. Stern-Volmer Plot for all quenching experiments

From Stern-Volmer, we could see under base condition and with O_2, substrate **1a** was the quencher of the photocatalyst. Without base or O_2, substrate **1a** will not quench the photocatalyst.
At the same time, Selectfluor is also the quencher of the photocatalyst.

Base on Green Chem. (2022, 24, 5553-5558), ChemSusChem (2016, 9, 241-245) and our observations, the following mechanism is proposed:

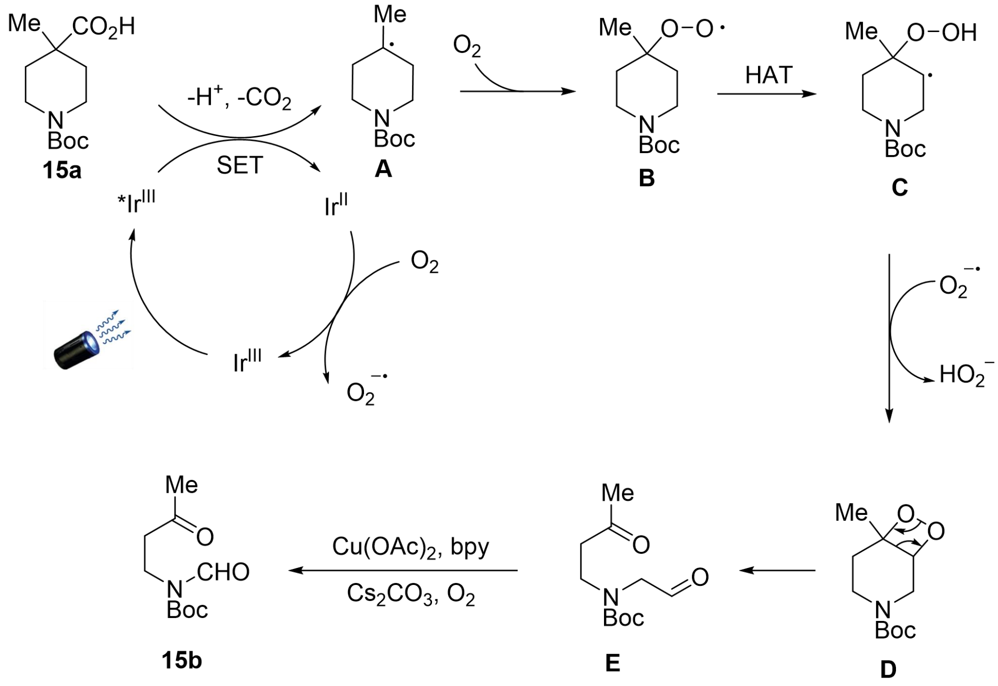

Supplementary Fig. 26. Proposed mechanism for photocatalytic reaction

However, the possibility of another mechanism could not be ruled out (Isr. J. Chem. 60, 410-415 (2020); J. Am. Chem. Soc. 141, 10556-10564 (2019).).

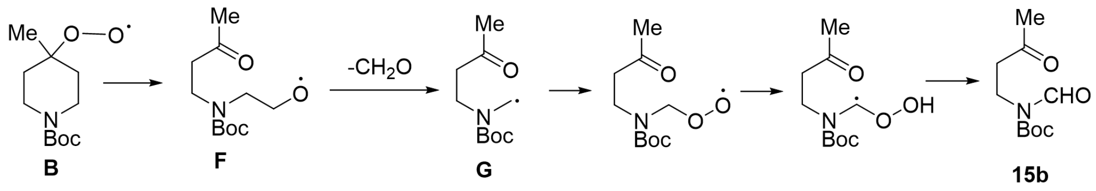

Supplementary Fig. 27. Proposed mechanism for photocatalytic reaction

3 Supplementary Notes

3.1 Supplementary Gram-scale Reaction

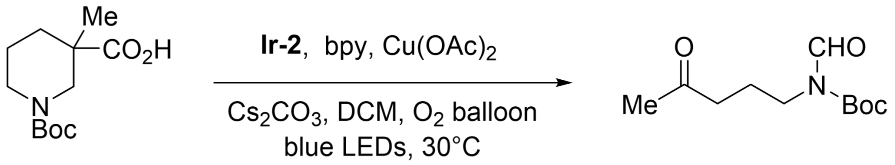

22a, 4 mmol

22b, 73%

This experiment was executed as follows: to a 250 mL round bottomed flask equipped with a magnetic stirrer bar were added the acid (1.0 mmol, 1.0 equiv.), $[Ir(dF(CF_3)ppy)_2(dOMebpy)]PF_6$ (33.4 mg, 0.03 mmol, 0.03 equiv.), $Cu(OAc)_2$ (36.2 mg, 0.2 mmol, 0.2 equiv.), 2,2'-bipyridine (39.0 mg, 0.25 mmol, 0.25 equiv.), Cs_2CO_3 (490.0 mg, 1.5 mmol, 1.5 equiv.) and DCM (20 mL). The flask was quickly degassed three times and flushed with oxygen through balloon, and then the mixture was irradiated with two 45 W blue LEDs (5 cm away) at 30 °C for 10 hours. Then another batch of the acid (1.0 mmol, 1.0 equiv.), $[Ir(dF(CF_3)ppy)_2(dOMebpy)]PF_6$ (33.4 mg, 0.03 mmol, 0.03 equiv.), $Cu(OAc)_2$ (36.2 mg, 0.2 mmol, 0.2 equiv.), 2,2'-bipyridine (39.0 mg, 0.25 mmol, 0.25 equiv.), Cs_2CO_3 (490.0 mg, 1.5 mmol, 1.5 equiv.) and DCM (20 mL) were added into the mixture. The flask was quickly degassed three times and flushed with oxygen through balloon. Then the mixture was irradiated with two 45 W blue LEDs (5 cm away) at 30 °C for 10 hours. Another batch of the acids and the catalysts were added. 10 hours later, the forth batch of the acids and the catalysts were added. The flask was quickly degassed three times and flushed with oxygen through balloon, and then the mixture was irradiated with two 45 W blue LEDs (5 cm away) at 30 °C for 48 hours. The reaction mixture was filtered and concentrated. The residue was purified by column chromatography on silica with petroleum ether/ethyl acetate as eluent. The desired product was obtained as colorless oil (669 mg, 73% yield).

3.2 Supplementary General Procedures and Experimental Data

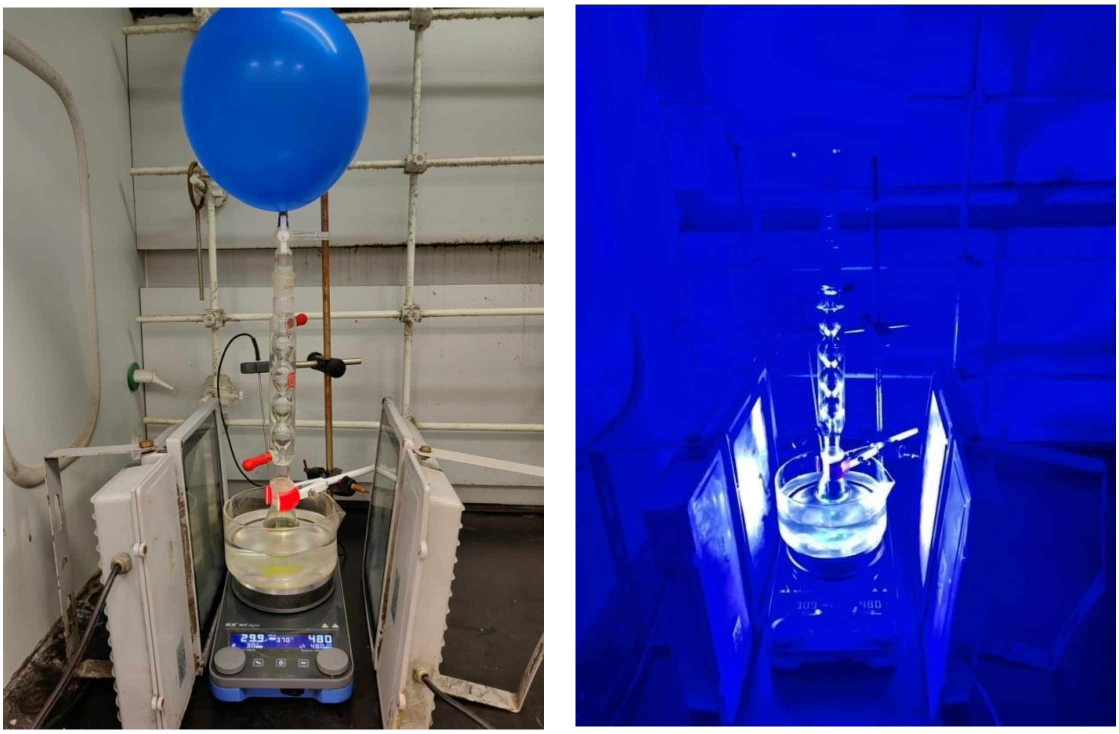

Supplementary Fig. 28. Pictures of experimental setup

(a) General procedure A for the reaction: To a 50 mL round bottomed flask equipped with a magnetic stirrer bar were added acid (0.5 mmol, 1.0 equiv.), photocatalyst (**Ir-1** or **Ir-2**) (0.015 mmol, 0.03 equiv.), $Cu(OAc)_2$ (18.1 mg, 0.1 mmol, 0.2 equiv.), 2,2'-bipyridine (19.5 mg, 0.125 mmol, 0.25 equiv.), Cs_2CO_3 (245 mg, 0.75 mmol, 1.5 equiv.) and DCM (10 mL). The flask was quickly degassed three times and flushed with oxygen through balloon, and then the mixture was heated to 30 ^oC in an oil bath and irradiated with two 45 W blue LEDs (5 cm away) for 40 hours or 72 hours. The reaction mixture was filtered and concentrated. The residue was purified by column chromatography on silica with petroleum ether/ethyl acetate mixture as the eluent.

(b) General procedure B for the reaction: To a 50 mL round bottomed flask equipped with a magnetic stirrer bar were added acid (0.5 mmol, 1.0 equiv.), $[(dF(CF_3)ppy)_2$-Ir- -Cl$]_2$ (**Ir-1**) (22.3 mg, 0.015 mmol, 0.03 equiv.), $Cu(OAc)_2$ (18.1 mg, 0.1 mmol, 0.2 equiv.), 2,2'-bipyridine (19.5 mg, 0.125 mmol, 0.25 equiv.), Cs_2CO_3 (245 mg, 0.75 mmol, 1.5 equiv.), Selectfluor (265 mg, 0.75 mmol, 1.5 equiv.) and DCM (10 mL). The flask was quickly degassed two times and flushed with oxygen through balloon, and then the mixture was heated to 30 ^oC in an oil bath and irradiated with two 45 W blue LEDs (5 cm away) for 40 hours. The reaction mixture was filtered and concentrated. The residue was purified by column chromatography on silica with petroleum ether/ethyl acetate mixture as the eluent.

tert-butyl formyl(3-oxo-3-phenylpropyl)carbamate (1b)

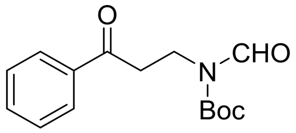

Prepared following the general procedure A employing **1a** (153 mg, 0.5 mmol, 1.0 equiv.), [(dF(CF$_3$)ppy)$_2$-Ir- -Cl]$_2$ (22.3 mg, 0.015 mmol, 0.03 equiv.), Cu(OAc)$_2$ (18.1 mg, 0.1 mmol, 0.2 equiv.), 2,2'-bipyridine (19.5 mg, 0.125 mmol, 0.25 equiv.), Cs_2CO_3 (245 mg, 0.75 mmol, 1.5 equiv.) and DCM (10 mL) for 40 hours. The desired product was obtained as white solid (112 mg, 81% yield) after purification by flash column chromatography on silica gel (petroleum ether/ethyl acetate = 10/1). Data are consistent with those reported in the literature.[7]

^{1}H NMR (400 MHz, $CDCl_3$) δ 9.16 (s, 1H), 7.91 (d, J = 7.4 Hz, 2H), 7.54 (t, J = 7.4 Hz, 1H), 7.43 (t, J = 7.7 Hz, 2H), 4.01 (t, J = 7.6 Hz, 2H), 3.19 (t, J = 7.6 Hz, 2H), 1.50 (s, 9H); ^{13}C NMR (101 MHz, $CDCl_3$) δ 197.7, 163.0, 152.2, 136.6, 133.4, 128.7, 128.1, 84.4, 37.0, 36.7, 28.1; HRMS (ESI) calcd for $C_{15}H_{19}NNaO_4$ $[M + Na]^+$ m/z = 300.1206, found: 300.1207.

N-formyl-N-(3-oxo-3-phenylpropyl)benzamide (2b)

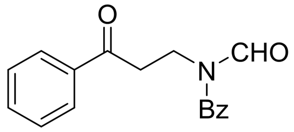

Prepared following the general procedure A employing **2a** (155 mg, 0.5 mmol, 1.0 equiv.), [(dF(CF$_3$)ppy)$_2$-Ir- -Cl]$_2$ (22.3 mg, 0.015 mmol, 0.03 equiv.), Cu(OAc)$_2$ (18.1 mg, 0.1 mmol, 0.2 equiv.), 2,2'-bipyridine (19.5 mg, 0.125 mmol, 0.25 equiv.), Cs_2CO_3 (245 mg, 0.75 mmol, 1.5 equiv.) and DCM (10 mL) for 40 hours. The desired product was obtained as white solid (80 mg, 57% yield) after purification by flash column chromatography on silica gel (petroleum ether/ethyl acetate = 10/1).

^{1}H NMR (400 MHz, $CDCl_3$) δ 8.94 (s, 1H), 7.96 (d, J = 7.9 Hz, 2H), 7.61 - 7.53 (m, 4H), 7.53 - 7.43 (m, 4H), 4.30 (t, J = 7.1 Hz, 2H), 3.39 (t, J = 7.1 Hz, 2H); ^{13}C NMR (101 MHz, $CDCl_3$) δ 197.9, 172.5, 164.2, 136.7, 133.5, 132.5, 129.1, 128.8, 128.2, 36.9, 36.5; HRMS (ESI) calcd for $C_{17}H_{15}NNaO_3$ $[M + Na]^+$ m/z = 304.0944, found: 304.0942.

tert-butyl (3-(4-fluorophenyl)-3-oxopropyl)(formyl)carbamate (3b)

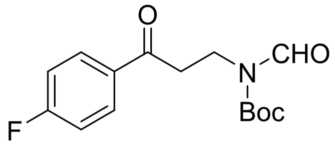

Prepared following the general procedure A employing **3a** (162 mg, 0.5 mmol, 1.0 equiv.), [(dF(CF$_3$)ppy)$_2$-Ir- -Cl]$_2$ (22.3 mg, 0.015 mmol, 0.03 equiv.), Cu(OAc)$_2$ (18.1 mg, 0.1 mmol, 0.2 equiv.), 2,2'-bipyridine (19.5 mg, 0.125 mmol, 0.25 equiv.), Cs_2CO_3 (245 mg, 0.75 mmol, 1.5 equiv.) and DCM (10 mL) for 40 hours. The desired product was obtained as white solid (128 mg, 87% yield) after purification by flash column chromatography on silica gel (petroleum ether/ethyl acetate = 10/1).

^{1}H NMR (400 MHz, $CDCl_3$) δ 9.14 (s, 1H), 8.02 - 7.85 (m, 2H), 7.17 - 7.03 (m, 2H), 3.99 (t, J = 7.6 Hz, 2H), 3.16 t, J = 7.6 Hz, 2H), 1.50 (s, 9H); ^{13}C NMR (101 MHz, $CDCl_3$) δ 196.2, 165.8 (d,

J = 256 Hz)., 163.0, 152.2, 133.0, 130.8 (d, J = 9.4 Hz), 115.8 (d, J = 22.0 Hz), 84.5, 36.9, 36.7, 28.1; HRMS (ESI) calcd for $C_{15}H_{18}FNNaO_4$ [M + Na] $^+$ m/z = 318.1112, found: 318.1109; IR ν_{max}/cm^{-1} (film) : 2979, 1740, 1686, 1344, 1150, 1014, 849, 811, 778.

tert-butyl (3-(4-chlorophenyl)-3-oxopropyl)(formyl)carbamate (4b)

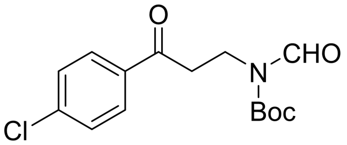

Prepared following the general procedure A employing **4a** (170 mg, 0.5 mmol, 1.0 equiv.), [(dF(CF$_3$)ppy)$_2$-Ir- -Cl]$_2$ (22.3 mg, 0.015 mmol, 0.03 equiv.), Cu(OAc)$_2$ (18.1 mg, 0.1 mmol, 0.2 equiv.), 2,2'-bipyridine (19.5 mg, 0.125 mmol, 0.25 equiv.), Cs_2CO_3 (245 mg, 0.75 mmol, 1.5 equiv.) and DCM (10 mL) for 40 hours. The desired product was obtained as white solid (120 mg, 77% yield) after purification by flash column chromatography on silica gel (petroleum ether/ethyl acetate = 10/1).

^{1}H NMR (400 MHz, CDCl$_3$) δ 9.14 (s, 1H), 7.85 (d, J = 8.6 Hz, 2H), 7.40 (d, J = 8.6 Hz, 2H), 3.99 (t, J = 7.6 Hz, 2H), 3.16 (t, J = 7.6 Hz, 2H),, 1.51 (s, 9H); ^{13}C NMR (101 MHz, CDCl$_3$) δ 196.5, 163.0, 152.1, 139.8, 134.9, 129.5, 129.1, 84.5, 37.0, 36.6, 28.1; HRMS (ESI) calcd for $C_{15}H_{18}ClNNaO_4$ [M + Na] $^+$ m/z = 334.0817, found: 334.0815; IR ν_{max}/cm^{-1} (film) : 2979, 1740, 1686, 1344, 1150, 1014, 987, 849, 811, 778.

tert-butyl formyl(3-oxo-3-(4-(trifluoromethyl)phenyl)propyl)carbamate (5b)

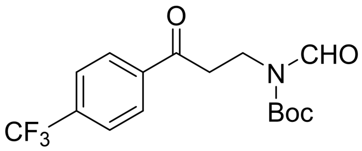

Prepared following the general procedure A employing **5a** (187 mg, 0.5 mmol, 1.0 equiv.), [(dF(CF$_3$)ppy)$_2$-Ir- -Cl]$_2$ (22.3 mg, 0.015 mmol, 0.03 equiv.), Cu(OAc)$_2$ (18.1 mg, 0.1 mmol, 0.2 equiv.), 2,2'-bipyridine (19.5 mg, 0.125 mmol, 0.25 equiv.), Cs_2CO_3 (245 mg, 0.75 mmol, 1.5 equiv.) and DCM (10 mL) for 40 hours. The desired product was obtained as white solid (90 mg, 52% yield) after purification by flash column chromatography on silica gel (petroleum ether/ethyl acetate = 10/1).

^{1}H NMR (400 MHz, CDCl$_3$) δ 9.16 (s, 1H), 8.03 (d, J = 8.1 Hz, 2H), 7.72 (d, J = 8.2 Hz, 2H), 4.03 (t, J = 7.6 Hz, 2H), 3.23 (t, J = 7.6 Hz, 2H), 1.53 (s, 9H); ^{13}C NMR (101 MHz, CDCl$_3$) δ 196.9, 163.1, 152.1, 139.2, 134.6 (d, J = 32.7 Hz), 128.5, 125.9 (q, J = 3.7 Hz), 123.7 (d, J = 274 Hz), 84.7, 37.4, 36.5, 28.1; HRMS (ESI) calcd for $C_{16}H_{18}F_3NNaO_4$ [M + Na] $^+$ m/z = 368.1080, found: 368.1086; IR ν_{max}/cm^{-1} (film) : 2981, 2936, 1741, 1691, 1326, 1150, 1067, 1016, 853, 805, 778.

tert-butyl formyl(3-(4-methoxyphenyl)-3-oxopropyl)carbamate (6b)

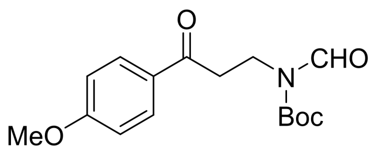

Prepared following the general procedure A employing **6a** (168 mg, 0.5 mmol, 1.0 equiv.), [(dF(CF$_3$)ppy)$_2$-Ir- -Cl]$_2$ (22.3 mg, 0.015 mmol, 0.03 equiv.), Cu(OAc)$_2$ (18.1 mg, 0.1 mmol, 0.2 equiv.), 2,2'-bipyridine (19.5 mg, 0.125 mmol, 0.25 equiv.), Cs$_2$CO$_3$ (245 mg, 0.75 mmol, 1.5 equiv.) and DCM (10 mL) for 40 hours. The desired product was obtained as white solid (101 mg, 66% yield) after purification by flash column chromatography on silica gel (petroleum ether/ethyl acetate = 10/1).

^{1}H NMR (400 MHz, CDCl$_3$) δ 9.15 (s, 1H), 7.89 (d, J = 9.0 Hz, 2H), 6.90 (d, J = 8.9 Hz, 2H), 3.99 (t, J = 7.6 Hz, 2H), 3.83 (s, 3H), 3.14 (t, J = 7.6 Hz, 2H), 1.50 (s, 9H); ^{13}C NMR (101 MHz, CDCl$_3$) δ 196.3, 163.7, 163.0, 152.3, 130.4, 129.8, 113.9, 84.4, 55.5, 36.9, 36.7, 28.1; HRMS (ESI) calcd for $C_{16}H_{21}NNaO_5$ [M + Na]$^+$ m/z = 330.1312, found: 330.1316; IR ν_{max}/cm^{-1} (film) : 2966, 2871, 1739, 1683, 1343, 1270, 1150, 1014, 987, 849, 794, 777.

tert-butyl (3-(4-(tert-butyl)phenyl)-3-oxopropyl)(formyl)carbamate (7b)

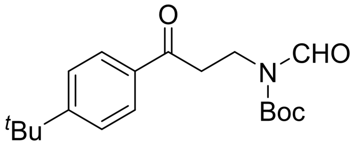

Prepared following the general procedure A employing **7a** (181 mg, 0.5 mmol, 1.0 equiv.), [(dF(CF$_3$)ppy)$_2$-Ir- -Cl]$_2$ (22.3 mg, 0.015 mmol, 0.03 equiv.), Cu(OAc)$_2$ (18.1 mg, 0.1 mmol, 0.2 equiv.), 2,2'-bipyridine (19.5 mg, 0.125 mmol, 0.25 equiv.), Cs$_2$CO$_3$ (245 mg, 0.75 mmol, 1.5 equiv.) and DCM (10 mL) for 40 hours. The desired product was obtained as white solid (133 mg, 80% yield) after purification by flash column chromatography on silica gel (petroleum ether/ethyl acetate = 10/1).

^{1}H NMR (400 MHz, CDCl$_3$) δ 9.16 (s, 1H), 7.86 (d, J = 8.4 Hz, 2H), 7.45 (d, J = 8.4 Hz, 2H), 4.01 (t, J = 7.6 Hz, 2H), 3.18 (t, J = 7.6 Hz, 2H), 1.50 (s, 9H), 1.31 (s, 9H); ^{13}C NMR (101 MHz, CDCl$_3$) δ 197.4, 163.0, 157.1, 152.3, 134.1, 128.1, 125.7, 84.3, 36.9, 36.8, 35.2, 31.1, 28.1; HRMS (ESI) calcd for $C_{19}H_{27}NNaO_4$ [M + Na]$^+$ m/z = 356.1832, found: 356.1836; IR ν_{max}/cm^{-1} (film) : 2978, 2842, 1739, 1685, 1344, 1259, 1171, 1149, 1029, 985, 845, 807, 777.

tert-butyl (3-([1,1'-biphenyl]-4-yl)-3-oxopropyl)(formyl)carbamate (8b)

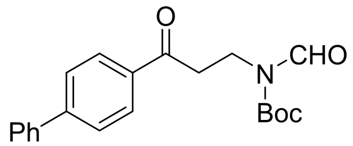

Prepared following the general procedure A employing **8a** (191 mg, 0.5 mmol, 1.0 equiv.), [(dF(CF$_3$)ppy)$_2$-Ir- -Cl]$_2$ (22.3 mg, 0.015 mmol, 0.03 equiv.), Cu(OAc)$_2$ (18.1 mg, 0.1 mmol, 0.2 equiv.), 2,2'-bipyridine (19.5 mg, 0.125 mmol, 0.25 equiv.), Cs$_2$CO$_3$ (245 mg, 0.75 mmol, 1.5 equiv.) and DCM (10 mL) for 40 hours. The desired product was obtained as white solid (141 mg, 80% yield) after purification by flash column chromatography on silica gel (petroleum ether/ethyl acetate = 10/1).

^{1}H NMR (400 MHz, $CDCl_3$) δ 9.20 (s, 1H), 8.01 (d, J = 8.3 Hz, 2H), 7.67 (d, J = 8.3 Hz, 2H), 7.61 (d, J = 7.2 Hz, 2H), 7.46 (t, J = 7.4 Hz, 2H), 7.40 (t, J = 7.2 Hz, 1H), 4.06 (t, J = 7.6 Hz, 2H), 3.25 (t, J = 7.6 Hz, 2H), 1.54 (s, 9H); ^{13}C NMR (101 MHz, $CDCl_3$) δ 197.4, 163.1, 152.3, 146.1, 139.9, 135.3, 129.0, 128.7, 128.4, 127.4, 127.3, 84.5, 37.1, 36.8, 28.1; HRMS (ESI) calcd for $C_{21}H_{23}NNaO_4$ $[M + Na]^+$ m/z = 376.1519, found: 376.1516; IR ν_{max}/cm^{-1} (film) : 2978, 1739, 1684, 1344, 1149, 1014, 851, 762, 698.

tert-butyl formyl(3-oxo-3-(thiophen-3-yl)propyl)carbamate (9b)

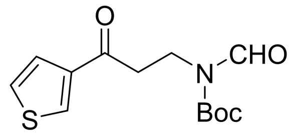

Prepared following the general procedure A employing **9a** (156 mg, 0.5 mmol, 1.0 equiv.), $[(dF(CF_3)ppy)_2$-Ir-   -$Cl]_2$ (22.3 mg, 0.015 mmol, 0.03 equiv.), $Cu(OAc)_2$ (18.1 mg, 0.1 mmol, 0.2 equiv.), 2,2'-bipyridine (19.5 mg, 0.125 mmol, 0.25 equiv.), Cs_2CO_3 (245 mg, 0.75 mmol, 1.5 equiv.) and DCM (10 mL) for 40 hours. The desired product was obtained as white solid (115 mg, 81% yield) after purification by flash column chromatography on silica gel (petroleum ether/ethyl acetate = 10/1).

^{1}H NMR (400 MHz, $CDCl_3$) δ 9.14 (s, 1H), 8.05 (dd, J = 2.7, 1.1 Hz, 1H), 7.50 (dd, J = 5.1, 1.0 Hz, 1H), 7.29 (dd, J = 5.1, 2.9 Hz, 1H), 3.98 (t, J = 7.6 Hz, 2H), 3.09 (t, J = 7.6 Hz, 2H), 1.50 (s, 9H); ^{13}C NMR (101 MHz, $CDCl_3$) δ 192.0, 163.0, 152.2, 142.0, 132.4, 126.9, 126.6, 84.5, 38.3, 36.7, 28.1; HRMS (ESI) calcd for $C_{13}H_{17}NNaO_4S$ $[M + Na]^+$ m/z = 306.0770, found: 306.0772; IR ν_{max}/cm^{-1} (film) : 2979, 1740, 1684, 1512, 1343, 1149, 1014, 798, 777.

1-methylindoline-2,3-dione (10b)

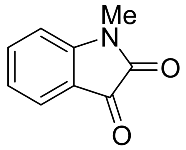

(a) Prepared following the general procedure A employing 1-(tert-butoxycarbonyl)-4-(1-methyl-1*H*-indol-3-yl)piperidine-4-carboxylic acid (179 mg, 0.5 mmol, 1.0 equiv.), $[Ir(dF(CF_3)ppy)_2(dOMebpy)]PF_6$ (16.7 mg, 0.015 mmol, 0.03 equiv.), $Cu(OAc)_2$ (18.1 mg, 0.1 mmol, 0.2 equiv.), 2,2'-bipyridine (19.5 mg, 0.125 mmol, 0.25 equiv.), Cs_2CO_3 (245 mg, 0.75 mmol, 1.5 equiv.) and DCM (10 mL) for 72 hours. The desired product was obtained as brown solid (64 mg, 80% yield) after purification by flash column chromatography on silica gel (petroleum ether/ethyl acetate = 5/1). Data are consistent with those reported in the literature.

^{1}H NMR (400 MHz, $CDCl_3$) δ 7.64-7.58 (m, 2H), 7.16-7.11 (m, 1H), 6.91-6.89 (m, 1H), 3.26 (s, 3H).^{13}C NMR (101 MHz, $CDCl_3$) δ 183.4, 158.3, 151.5, 138.4, 125.3, 123.9, 117.5, 109.9, 26.2.

4-oxo-4-phenylbutanal (11b)

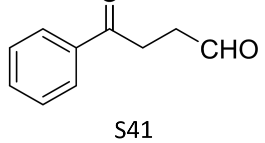

Prepared following the general procedure A employing 1-phenylcyclobutane-1-carboxylic acid (88 mg, 0.5 mmol, 1.0 equiv.), [(dF(CF_3)ppy)$_2$-Ir- -Cl]$_2$ (22.3 mg, 0.015 mmol, 0.03 equiv.), Cu(OAc)$_2$ (18.1 mg, 0.1 mmol, 0.2 equiv.), 2,2'-bipyridine (19.5 mg, 0.125 mmol, 0.25 equiv.), Cs_2CO_3 (245 mg, 0.75 mmol, 1.5 equiv.) and DCM (10 mL) for 40 hours. The desired product was obtained as white solid (71 mg, 88% yield) after purification by flash column chromatography on silica gel (petroleum ether/ethyl acetate = 10/1). Data are consistent with those reported in the literature.[9]

^{1}H NMR (400 MHz, $CDCl_3$) δ 9.92 (s, 1H), 7.99 (d, J = 8.1 Hz, 2H), 7.58 (t, J = 7.3 Hz, 1H), 7.48 (t, J = 7.5 Hz, 2H), 3.34 (t, J = 6.2 Hz, 2H), 2.94 (t, J = 6.1 Hz, 2H).; ^{13}C NMR (101 MHz, $CDCl_3$) δ 200.8, 198.0, 136.6, 133.5, 128.8, 128.2, 37.8, 31.2; HRMS (ESI) calcd for $C_{10}H_{10}NaO_2$ $[M+Na]^+$ m/z = 185.0573, found: 185.0573.

4-(4-fluorophenyl)-4-oxobutanal (12b)

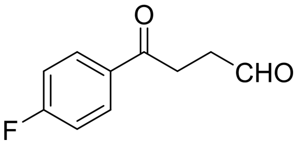

Prepared following the general procedure A employing 1-(4-fluorophenyl)cyclobutane-1-carboxylic acid (97 mg, 0.5 mmol, 1.0 equiv.), [(dF(CF_3)ppy)$_2$-Ir- -Cl]$_2$ (22.3 mg, 0.015 mmol, 0.03 equiv.), Cu(OAc)$_2$ (18.1 mg, 0.1 mmol, 0.2 equiv.), 2,2'-bipyridine (19.5 mg, 0.125 mmol, 0.25 equiv.), Cs_2CO_3 (245 mg, 0.75 mmol, 1.5 equiv.) and DCM (10 mL) for 40 hours. The desired product was obtained as white solid (58 mg, 65% yield) after purification by flash column chromatography on silica gel (petroleum ether/ethyl acetate = 10/1). Data are consistent with those reported in the literature.[10]

^{1}H NMR (400 MHz, $CDCl_3$) δ 9.91 (s, 1H), 8.02 (dd, J = 8.7, 5.5 Hz, 2H), 7.15 (t, J = 8.6 Hz, 2H), 3.30 (t, J = 6.3 Hz, 2H), 2.95 (t, J = 6.3 Hz, 2H); ^{13}C NMR (101 MHz, $CDCl_3$) δ 200.6, 196.4, 165.0 (d, J = 256 Hz), 133.0, 130.9 (d, J = 10.3 Hz), 115.9 (d, J = 20.0 Hz), 37.7, 31.0; HRMS (ESI) calcd for $C_{10}H_{12}FO_2$ $[M+H]^+$ m/z = 181.0659, found: 181.0652.

4-(4-bromophenyl)-4-oxobutanal (13b)

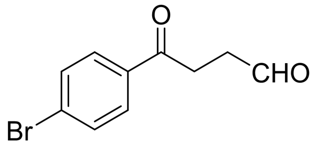

Prepared following the general procedure A employing 1-(4-bromophenyl)cyclobutane-1-carboxylic acid (127 mg, 0.5 mmol, 1.0 equiv.), [(dF(CF_3)ppy)$_2$-Ir- -Cl]$_2$ (22.3 mg, 0.015 mmol, 0.03 equiv.), Cu(OAc)$_2$ (18.1 mg, 0.1 mmol, 0.2 equiv.), 2,2'-bipyridine (19.5 mg, 0.125 mmol, 0.25 equiv.), Cs_2CO_3 (245 mg, 0.75 mmol, 1.5 equiv.) and DCM (10 mL) for 40 hours. The desired product was obtained as white solid (81 mg, 67% yield) after purification by flash column chromatography on silica gel (petroleum ether/ethyl acetate = 10/1). Data are consistent with those reported in the literature.[11]

^{1}H NMR (400 MHz, $CDCl_3$) δ 9.90 (s, 1H), 7.85 (d, J = 8.6 Hz, 2H), 7.62 (d, J = 8.5 Hz, 2H), 3.29 (t, J = 6.3 Hz, 2H), 2.95 (t, J = 6.3 Hz, 2H). ^{13}C NMR (101 MHz, $CDCl_3$) δ 200.5, 196.9,

135.3, 132.1, 129.7, 128.7, 37.7, 31.0; HRMS (ESI) calcd for $C_{10}H_{10}BrO_2$ $[M+H]^+$ m/z = 240.9859, found: 240.9855.

5-oxo-5-phenylpentanal (14b)

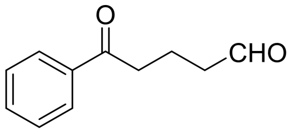

Prepared following the general procedure A employing 1-phenylcyclopentane-1-carboxylic acid (95 mg, 0.5 mmol, 1.0 equiv.), $[(dF(CF_3)ppy)_2$-Ir-   -$Cl]_2$ (22.3 mg, 0.015 mmol, 0.03 equiv.), $Cu(OAc)_2$ (18.1 mg, 0.1 mmol, 0.2 equiv.), 2,2'-bipyridine (19.5 mg, 0.125 mmol, 0.25 equiv.), Cs_2CO_3 (245 mg, 0.75 mmol, 1.5 equiv.) and DCM (10 mL) for 40 hours. The desired product was obtained as white solid (58 mg, 65% yield) after purification by flash column chromatography on silica gel (petroleum ether/ethyl acetate = 10/1). Data are consistent with those reported in the literature.[12]

^{1}H NMR (400 MHz, $CDCl_3$) δ 9.81 (s, 1H), 7.96 (d, J = 7.9 Hz, 2H), 7.57 (t, J = 7.4 Hz, 1H), 7.47 (t, J = 7.6 Hz, 2H), 3.06 (t, J = 7.0 Hz, 2H), 2.60 (t, J = 7.1 Hz, 2H), 2.09 (p, J = 7.0 Hz, 2H); ^{13}C NMR (101 MHz, $CDCl_3$) δ 202.1, 199.5, 136.9, 133.3, 128.8, 128.2, 43.2, 37.4, 16.7; HRMS (ESI) calcd for $C_{11}H_{12}NaO_2$ $[M+Na]^+$ m/z = 199.0730, found: 199.0733.

tert-butyl formyl(3-oxobutyl)carbamate (15b and 21b)

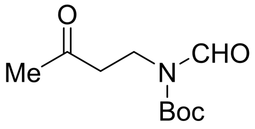

(a) Prepared following the general procedure A employing 1-(tert-butoxycarbonyl)-4-methylpiperidine-4-carboxylic acid (122 mg, 0.5 mmol, 1.0 equiv.), $[Ir(dF(CF_3)ppy)_2(dOMebpy)]PF_6$ (16.7 mg, 0.015 mmol, 0.03 equiv.), $Cu(OAc)_2$ (18.1 mg, 0.1 mmol, 0.2 equiv.), 2,2'-bipyridine (19.5 mg, 0.125 mmol, 0.25 equiv.), Cs_2CO_3 (245 mg, 0.75 mmol, 1.5 equiv.) and DCM (10 mL) for 72 hours. The desired product was obtained as colorless oil (58 mg, 54% yield) after purification by flash column chromatography on silica gel (petroleum ether/ethyl acetate = 10/1).

(b) Prepared following the general procedure A employing 1-(tert-butoxycarbonyl)-3-methylpyrrolidine-3-carboxylic acid (115 mg, 0.5 mmol, 1.0 equiv.), $[Ir(dF(CF_3)ppy)_2(dOMebpy)]PF_6$ (16.7 mg, 0.015 mmol, 0.03 equiv.), $Cu(OAc)_2$ (18.1 mg, 0.1 mmol, 0.2 equiv.), 2,2'-bipyridine (19.5 mg, 0.125 mmol, 0.25 equiv.), Cs_2CO_3 (245 mg, 0.75 mmol, 1.5 equiv.) and DCM (10 mL) for 72 hours. The desired product was obtained as colorless oil (69 mg, 64% yield) after purification by flash column chromatography on silica gel (petroleum ether/ethyl acetate = 10/1).

^{1}H NMR (400 MHz, $CDCl_3$) δ 9.10 (s, 1H), 3.83 (t, J = 7.6 Hz, 2H), 2.66 (t, J = 7.6 Hz, 2H), 2.14 (s, 3H), 1.52 (s, 9H); ^{13}C NMR (101 MHz, $CDCl_3$) δ 206.1, 162.8, 152.0, 84.2, 41.7, 35.7, 29.9, 27.9; HRMS (ESI) calcd for $C_{10}H_{17}NNaO_4$ $[M + Na]^+$ m/z = 238.1050, found: 238.1044; IR ν_{max}/cm^{-1} (film) : 2980, 1741, 1691, 1371, 1343, 1152, 1054, 852, 777.

tert-butyl formyl(3-oxopentyl)carbamate (16b)

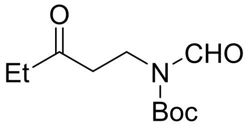

Prepared following the general procedure A employing 1-(tert-butoxycarbonyl)-4-ethylpiperidine-4-carboxylic acid (129 mg, 0.5 mmol, 1.0 equiv.), [Ir(dF(CF_3)ppy)$_2$(dOMebpy)]PF_6 (16.7 mg, 0.015 mmol, 0.03 equiv.), Cu(OAc)$_2$ (18.1 mg, 0.1 mmol, 0.2 equiv.), 2,2'-bipyridine (19.5 mg, 0.125 mmol, 0.25 equiv.), Cs_2CO_3 (245 mg, 0.75 mmol, 1.5 equiv.) and DCM (10 mL) for 72 hours. The desired product was obtained as colorless oil (50 mg, 44% yield) after purification by flash column chromatography on silica gel (petroleum ether/ethyl acetate = 10/1).

^{1}H NMR (400 MHz, $CDCl_3$) δ 9.06 (s, 1H), 3.80 (t, J = 7.6 Hz, 2H), 2.60 (t, J = 7.6 Hz, 2H), 2.38 (q, J = 7.3 Hz, 2H), 1.48 (s, 9H), 0.99 (t, J = 7.3 Hz, 3H); ^{13}C NMR (101 MHz, $CDCl_3$) δ 208.8, 162.9, 152.1, 84.3, 40.5, 36.0, 28.0, 7.6; HRMS (ESI) calcd for $C_{11}H_{19}NNaO_4$ [M + Na]$^+$ m/z = 252.1206, found: 252.1202; IR ν_{max}/cm^{-1} (film) : 2979, 2940, 1741, 1690, 1371, 1342, 1152, 1069, 852, 778.

tert-butyl 4-oxopiperidine-1-carboxylate (10c, 17b, 18b and 19b)

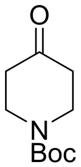

(a) Prepared following the general procedure A employing 1-(tert-butoxycarbonyl)-4-(1-methyl-1*H*-indol-3-yl)piperidine-4-carboxylic acid **10a** (179 mg, 0.5 mmol, 1.0 equiv.), [Ir(dF(CF_3)ppy)$_2$(dOMebpy)]PF_6 (16.7 mg, 0.015 mmol, 0.03 equiv.), Cu(OAc)$_2$ (18.1 mg, 0.1 mmol, 0.2 equiv.), 2,2'-bipyridine (19.5 mg, 0.125 mmol, 0.25 equiv.), Cs_2CO_3 (245 mg, 0.75 mmol, 1.5 equiv.) and DCM (10 mL) for 72 hours. The desired product was obtained as brown solid (79 mg, 80% yield) after purification by flash column chromatography on silica gel (petroleum ether/ethyl acetate = 10/1).

(b) Prepared following the general procedure A employing **17a** (135 mg, 0.5 mmol, 1.0 equiv.), [Ir(dF(CF_3)ppy)$_2$(dOMebpy)]PF_6 (16.7 mg, 0.015 mmol, 0.03 equiv.), Cu(OAc)$_2$ (18.1 mg, 0.1 mmol, 0.2 equiv.), 2,2'-bipyridine (19.5 mg, 0.125 mmol, 0.25 equiv.), Cs_2CO_3 (245 mg, 0.75 mmol, 1.5 equiv.) and DCM (10 mL) for 72 hours. The desired product was obtained as colorless oil (54 mg, 54% yield) after purification by flash column chromatography on silica gel (petroleum ether/ethyl acetate = 10/1).

(c) Prepared following the general procedure A employing **18a** (160 mg, 0.5 mmol, 1.0 equiv.), [Ir(dF(CF_3)ppy)$_2$(dOMebpy)]PF_6 (16.7 mg, 0.015 mmol, 0.03 equiv.), Cu(OAc)$_2$ (18.1 mg, 0.1 mmol, 0.2 equiv.), 2,2'-bipyridine (19.5 mg, 0.125 mmol, 0.25 equiv.), Cs_2CO_3 (245 mg, 0.75 mmol, 1.5 equiv.) and DCM (10 mL) for 72 hours. The desired product was obtained as colorless

oil (59 mg, 59% yield) after purification by flash column chromatography on silica gel (petroleum ether/ethyl acetate = 10/1).

(d) prepared following the general procedure A employing **19a** (136 mg, 0.5 mmol, 1.0 equiv.), [Ir(dF(CF$_3$)ppy)$_2$(dOMebpy)]PF$_6$ (16.7 mg, 0.015 mmol, 0.03 equiv.), Cu(OAc)$_2$ (18.1 mg, 0.1 mmol, 0.2 equiv.), 2,2'-bipyridine (19.5 mg, 0.125 mmol, 0.25 equiv.), Cs_2CO_3 (245 mg, 0.75 mmol, 1.5 equiv.) and DCM (10 mL) for 72 hours. The desired product was obtained as colorless oil (61 mg, 61% yield) after purification by flash column chromatography on silica gel (petroleum ether/ethyl acetate = 10/1).Data are consistent with those reported in the literature.[8]
^{1}H NMR (400 MHz, $CDCl_3$) δ 3.59 (t, J = 6.2 Hz, 4H), 2.31 (t, J = 6.2 Hz, 4H), 1.36 (s, 9H); ^{13}C NMR (101 MHz, $CDCl_3$) δ 207.5, 154.2, 80.1, 42.8, 40.8, 28.1.

tert-butyl formyl(2-oxopropyl)carbamate (20b)

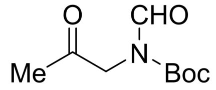

Prepared following the general procedure A employing 1-(tert-butoxycarbonyl)-3-methylazetidine-3-carboxylic acid (108 mg, 0.5 mmol, 1.0 equiv.), [(dF(CF$_3$)ppy)$_2$-Ir- -Cl]$_2$ (22.3 mg, 0.015 mmol, 0.03 equiv.), Cu(OAc)$_2$ (18.1 mg, 0.1 mmol, 0.2 equiv.), 2,2'-bipyridine (19.5 mg, 0.125 mmol, 0.25 equiv.), Cs_2CO_3 (245 mg, 0.75 mmol, 1.5 equiv.) and DCM (10 mL) for 72 hours. The desired product was obtained as white solid (58 mg, 58% yield) after purification by flash column chromatography on silica gel (petroleum ether/ethyl acetate = 10/1).
^{1}H NMR (400 MHz, $CDCl_3$) δ 9.18 (s, 1H), 4.38 (s, 2H), 2.16 (s, 3H), 1.49 (s, 9H); ^{13}C NMR (101 MHz, $CDCl_3$) δ 200.4, 162.4, 151.8, 84.8, 49.5, 28.0, 27.0; HRMS (ESI) calcd for $C_9H_{15}NNaO_4$ $[M + Na]^+$ m/z = 224.0893, found: 224.0898; IR ν_{max}/cm^{-1} (film) : 2979, 2940, 1741, 1371, 1342, 1152, 1069, 852, 811, 778.

tert-butyl formyl(4-oxopentyl)carbamate (22b)

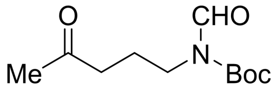

Prepared following the general procedure A employing 1-(tert-butoxycarbonyl)-3-methylpiperidine-3-carboxylic acid (122 mg, 0.5 mmol, 1.0 equiv.), [Ir(dF(CF$_3$)ppy)$_2$(dOMebpy)]PF$_6$ (16.7 mg, 0.015 mmol, 0.03 equiv.), Cu(OAc)$_2$ (18.1 mg, 0.1 mmol, 0.2 equiv.), 2,2'-bipyridine (19.5 mg, 0.125 mmol, 0.25 equiv.), Cs_2CO_3 (245 mg, 0.75 mmol, 1.5 equiv.) and DCM (10 mL) for 72 hours. The desired product was obtained as colorless oil (101 mg, 88% yield) after purification by flash column chromatography on silica gel (petroleum ether/ethyl acetate = 10/1).
^{1}H NMR (400 MHz, $CDCl_3$) δ 9.11 (s, 1H), 3.54 (t, J = 7.2 Hz, 2H), 2.39 (t, J = 7.2 Hz, 2H), 2.08 (s, 3H), 1.81 - 1.71 (m, 2H), 1.50 (s, 9H); ^{13}C NMR (101 MHz, $CDCl_3$) δ 207.6, 163.2, 152.5, 84.1, 40.5, 39.8, 29.9, 28.0, 22.3; HRMS (ESI) calcd for $C_{11}H_{19}NNaO_4$ $[M + Na]^+$ m/z = 252.1206, found: 252.1201; IR ν_{max}/cm^{-1} (film) : 2979, 1737, 1689, 1371, 1344, 1151, 1067, 891, 853, 778.

tert-butyl formyl(4-oxohexyl)carbamate (23b)

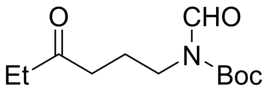

Prepared following the general procedure A employing 1-(tert-butoxycarbonyl)-3-ethylpiperidine-3-carboxylic acid (128 mg, 0.5 mmol, 1.0 equiv.), [Ir(dF(CF_3)ppy)$_2$(dOMebpy)]PF_6 (16.7 mg, 0.015 mmol, 0.03 equiv.), Cu(OAc)$_2$ (18.1 mg, 0.1 mmol, 0.2 equiv.), 2,2'-bipyridine (19.5 mg, 0.125 mmol, 0.25 equiv.), Cs_2CO_3 (245 mg, 0.75 mmol, 1.5 equiv.) and DCM (10 mL) for 72 hours. The desired product was obtained as colorless oil (95 mg, 78% yield) after purification by flash column chromatography on silica gel (petroleum ether/ethyl acetate = 10/1).

^{1}H NMR (400 MHz, $CDCl_3$) δ 9.09 (s, 1H), 3.52 (t, J = 7.0 Hz, 2H), 2.40 - 2.29 (m, 4H), 1.78-1.71 (m, 2H), 1.48 (s, 9H), 0.97 (t, J = 7.3 Hz, 3H); ^{13}C NMR (101 MHz, $CDCl_3$) δ 210.3, 163.1, 152.4, 84.0, 39.8, 39.2, 35.8, 28.0, 22.3, 7.8; HRMS (ESI) calcd for $C_{12}H_{21}NNaO_4$ [M + Na] $^+$ m/z = 266.1363, found: 266.1369; IR ν_{max}/cm^{-1} (film) : 2978, 1736, 1689, 1371, 1341, 1152, 854, 777.

tert-butyl formyl(4-oxo-5-phenylpentyl)carbamate (24b)

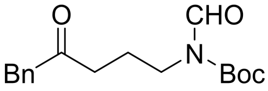

Prepared following the general procedure A employing 3-benzyl-1-(tert-butoxycarbonyl)piperidine-3-carboxylic acid (160 mg, 0.5 mmol, 1.0 equiv.), [Ir(dF(CF_3)ppy)$_2$(dOMebpy)]PF_6 (16.7 mg, 0.015 mmol, 0.03 equiv.), Cu(OAc)$_2$ (18.1 mg, 0.1 mmol, 0.2 equiv.), 2,2'-bipyridine (19.5 mg, 0.125 mmol, 0.25 equiv.), Cs_2CO_3 (245 mg, 0.75 mmol, 1.5 equiv.) and DCM (10 mL) for 72 hours. The desired product was obtained as colorless oil (47 mg, 31% yield) after purification by flash column chromatography on silica gel (petroleum ether/ethyl acetate = 10/1).

^{1}H NMR (400 MHz, $CDCl_3$) δ 9.13 (s, 1H), 7.31 (t, J = 7.2 Hz, 2H), 7.28 - 7.22 (m, 1H), 7.19 (d, J = 7.2 Hz, 2H), 3.67 (s, 2H), 3.55 (t, J = 7.0 Hz, 2H), 2.44 (t, J = 7.2 Hz, 2H), 1.82-1.74 (m, 2H), 1.52 (s, 9H); ^{13}C NMR (101 MHz, $CDCl_3$) δ 207.2, 163.2, 152.5, 134.3, 129.5, 128.8, 127.1, 84.1, 50.2, 39.8, 38.9, 28.1, 22.3; HRMS (ESI) calcd for $C_{17}H_{23}NNaO_4$ [M + Na] $^+$ m/z = 328.1519, found: 328.1519; IR ν_{max}/cm^{-1} (film) : 2978, 1736, 1689, 1370, 1340, 1149, 852, 777, 700.

tert-butyl formyl(5-methyl-4-oxohexyl)carbamate (25b)

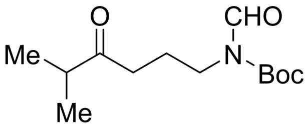

Prepared following the general procedure A employing 1-(tert-butoxycarbonyl)-3-isopropylpiperidine-3-carboxylic acid (136 mg, 0.5 mmol, 1.0 equiv.), [Ir(dF(CF_3)ppy)$_2$(dOMebpy)]PF_6 (16.7 mg, 0.015 mmol, 0.03 equiv.), Cu(OAc)$_2$ (18.1 mg, 0.1 mmol, 0.2 equiv.), 2,2'-bipyridine (19.5 mg, 0.125 mmol, 0.25 equiv.), Cs_2CO_3 (245 mg, 0.75 mmol, 1.5 equiv.) and DCM (10 mL) for 72 hours. The desired product was obtained as colorless

oil (103 mg, 80% yield) after purification by flash column chromatography on silica gel (petroleum ether/ethyl acetate = 10/1).

^{1}H NMR (400 MHz, $CDCl_3$) δ 9.07 (s, 1H), 3.51 (t, J = 7.1 Hz, 2H), 2.53-2.46 (m, 1H), 2.38 (t, J = 7.3 Hz, 2H), 1.75-1.68 (m, 2H), 1.46 (s, 9H), 1.00 (d, J = 6.9 Hz, 6H); ^{13}C NMR (101 MHz, $CDCl_3$) δ 213.4, 163.1, 152.4, 84.0, 40.7, 39.8, 37.2, 28.0, 22.3, 18.2. HRMS (ESI) calcd for $C_{13}H_{23}NNaO_4$ [M + Na]$^+$ m/z = 280.1519, found: 280.1512; IR ν_{max}/cm^{-1} (film) : 2927, 1735, 1685, 1508, 1458, 1157, 1019, 797.

benzophenone (26b and 27b)

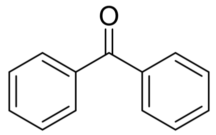

(a) Prepared following the general procedure A employing 2,2,2-triphenylacetic acid (144 mg, 0.5 mmol, 1.0 equiv.), [Ir(dF(CF_3)ppy)$_2$(dOMebpy)]PF_6 (16.7 mg, 0.015 mmol, 0.03 equiv.), Cu(OAc)$_2$ (18.1 mg, 0.1 mmol, 0.2 equiv.), 2,2'-bipyridine (19.5 mg, 0.125 mmol, 0.25 equiv.), Cs_2CO_3 (245 mg, 0.75 mmol, 1.5 equiv.) and DCM (10 mL) for 72 hours. The desired product was obtained as colorless oil (49 mg, 54% yield) after purification by flash column chromatography on silica gel (petroleum ether/ethyl acetate = 10/1).

(b) Prepared following the general procedure A employing 2,2-diphenylpentanoic acid (127 mg, 0.5 mmol, 1.0 equiv.), [Ir(dF(CF_3)ppy)$_2$(dOMebpy)]PF_6 (16.7 mg, 0.015 mmol, 0.03 equiv.), Cu(OAc)$_2$ (18.1 mg, 0.1 mmol, 0.2 equiv.), 2,2'-bipyridine (19.5 mg, 0.125 mmol, 0.25 equiv.), Cs_2CO_3 (245 mg, 0.75 mmol, 1.5 equiv.) and DCM (10 mL) for 72 hours. The desired product was obtained as colorless oil (47 mg, 52% yield) after purification by flash column chromatography on silica gel (petroleum ether/ethyl acetate = 10/1).Data are consistent with those reported in the literature.[13]

^{1}H NMR (400 MHz, $CDCl_3$) δ 7.81 (d, J = 7.7 Hz, 4H), 7.59 (t, J = 7.4 Hz, 2H), 7.48 (t, J = 7.6 Hz, 4H); HRMS (ESI) calcd for $C_{13}H_{10}NaO$ [M + Na]$^+$ m/z = 205.0624, found: 205.0623.

acetophenone (28b, 29b and 37b)

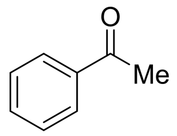

(a) Prepared following the general procedure A employing 2,2-diphenylpropanoic acid (113 mg, 0.5 mmol, 1.0 equiv.), [Ir(dF(CF_3)ppy)$_2$(dOMebpy)]PF_6 (16.7 mg, 0.015 mmol, 0.03 equiv.), Cu(OAc)$_2$ (18.1 mg, 0.1 mmol, 0.2 equiv.), 2,2'-bipyridine (19.5 mg, 0.125 mmol, 0.25 equiv.), Cs_2CO_3 (245 mg, 0.75 mmol, 1.5 equiv.) and DCM (10 mL) for 72 hours. The desired product was obtained as colorless oil (28 mg, 47% yield) after purification by flash column chromatography on silica gel (petroleum ether/ethyl acetate = 10/1).

(b) Prepared following the general procedure A employing 2-methyl-2-phenylpropanoic acid (132 mg, 0.5 mmol, 1.0 equiv.), [Ir(dF(CF_3)ppy)$_2$(dOMebpy)]PF_6 (16.7 mg, 0.015 mmol, 0.03 equiv.), Cu(OAc)$_2$ (18.1 mg, 0.1 mmol, 0.2 equiv.), 2,2'-bipyridine (19.5 mg, 0.125 mmol, 0.25 equiv.), Cs_2CO_3 (245 mg, 0.75 mmol, 1.5 equiv.) and DCM (10 mL) for 72 hours. The desired product was obtained as colorless oil (26 mg, 43% yield) after purification by flash column chromatography on silica gel (petroleum ether/ethyl acetate = 10/1).

(c) prepared following the general procedure A employing 3-phenylbutanoic acid (132 mg, 0.5 mmol, 1.0 equiv.), [Ir(dF(CF_3)ppy)$_2$(dOMebpy)]PF_6 (16.7 mg, 0.015 mmol, 0.03 equiv.), Cu(OAc)$_2$ (18.1 mg, 0.1 mmol, 0.2 equiv.), 2,2'-bipyridine (19.5 mg, 0.125 mmol, 0.25 equiv.), Cs_2CO_3 (245 mg, 0.75 mmol, 1.5 equiv.) and DCM (10 mL) for 72 hours. The desired product was obtained as colorless oil (40 mg, 66% yield) after purification by flash column chromatography on silica gel (petroleum ether/ethyl acetate = 10/1).Data are consistent with those reported in the literature.[14]

^{1}H NMR (400 MHz, $CDCl_3$) δ 7.96 (d, J = 7.6 Hz, 2H), 7.57 (t, J = 7.4 Hz, 1H), 7.47 (t, J = 7.6 Hz, 2H), 2.61 (s, 3H).

1-(p-tolyl)ethan-1-one (30b)

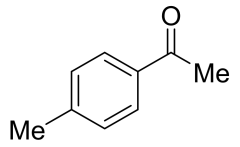

Prepared following the general procedure A employing 2-methyl-2-(p-tolyl)propanoic acid (89 mg, 0.5 mmol, 1.0 equiv.), [Ir(dF(CF_3)ppy)$_2$(dOMebpy)]PF_6 (16.7 mg, 0.015 mmol, 0.03 equiv.), Cu(OAc)$_2$ (18.1 mg, 0.1 mmol, 0.2 equiv.), 2,2'-bipyridine (19.5 mg, 0.125 mmol, 0.25 equiv.), Cs_2CO_3 (245 mg, 0.75 mmol, 1.5 equiv.) and DCM (10 mL) for 72 hours. The desired product was obtained as colorless oil (35 mg, 52% yield) after purification by flash column chromatography on silica gel (petroleum ether/ethyl acetate = 10/1). Data are consistent with those reported in the literature.[15]

^{1}H NMR (400 MHz, $CDCl_3$) δ 7.85 (d, J = 8.4 Hz, 2H), 7.25 (d, J = 8.4 Hz, 2H), 2.57 (s, 3H), 2.41 (s, 3H).

1-(4-chlorophenyl)ethan-1-one (31b)

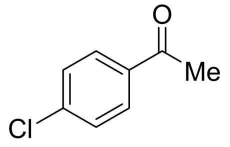

Prepared following the general procedure A employing 2-(4-chlorophenyl)-2-methylpropanoic acid (99 mg, 0.5 mmol, 1.0 equiv.), [Ir(dF(CF_3)ppy)$_2$(dOMebpy)]PF_6 (16.7 mg, 0.015 mmol, 0.03 equiv.), Cu(OAc)$_2$ (18.1 mg, 0.1 mmol, 0.2 equiv.), 2,2'-bipyridine (19.5 mg, 0.125 mmol, 0.25 equiv.), Cs_2CO_3 (245 mg, 0.75 mmol, 1.5 equiv.) and DCM (10 mL) for 72 hours. The desired product was obtained as colorless oil (31 mg, 41% yield) after purification by flash column chromatography on silica gel (petroleum ether/ethyl acetate = 10/1). Data are consistent with those reported in the literature.[16]

^{1}H NMR (400 MHz, $CDCl_3$) δ 7.89 (d, J = 8.4 Hz, 2H), 7.43 (d, J = 8.4 Hz, 2H), 2.59 (s, 3H).

1-(4-bromophenyl)ethan-1-one (32b)

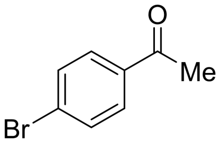

Prepared following the general procedure A employing 2-(4-bromophenyl)-2-methylpropanoic acid (121 mg, 0.5 mmol, 1.0 equiv.), [Ir(dF(CF_3)ppy)$_2$(dOMebpy)]PF_6 (16.7 mg, 0.015 mmol, 0.03 equiv.), Cu(OAc)$_2$ (18.1 mg, 0.1 mmol, 0.2 equiv.), 2,2'-bipyridine (19.5 mg, 0.125 mmol, 0.25 equiv.), Cs_2CO_3 (245 mg, 0.75 mmol, 1.5 equiv.) and DCM (10 mL) for 72 hours. The desired product was obtained as colorless oil (38 mg, 38% yield) after purification by flash column chromatography on silica gel (petroleum ether/ethyl acetate = 10/1). Data are consistent with those reported in the literature.[17]
^{1}H NMR (400 MHz, $CDCl_3$) δ 7.80 (d, J = 8.4 Hz, 2H), 7.58 (d, J = 8.4 Hz, 2H), 2.57 (s, 3H).

propiophenone (33b)

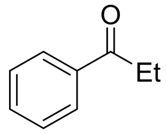

Prepared following the general procedure A employing 2-ethyl-2-phenylbutanoic acid (96 mg, 0.5 mmol, 1.0 equiv.), [Ir(dF(CF_3)ppy)$_2$(dOMebpy)]PF_6 (16.7 mg, 0.015 mmol, 0.03 equiv.), Cu(OAc)$_2$ (18.1 mg, 0.1 mmol, 0.2 equiv.), 2,2'-bipyridine (19.5 mg, 0.125 mmol, 0.25 equiv.), Cs_2CO_3 (245 mg, 0.75 mmol, 1.5 equiv.) and DCM (10 mL) for 72 hours. The desired product was obtained as colorless oil (58 mg, 86% yield) after purification by flash column chromatography on silica gel (petroleum ether/ethyl acetate = 10/1). Data are consistent with those reported in the literature.[17]
^{1}H NMR (400 MHz, $CDCl_3$) δ 7.96 (d, J = 7.2 Hz, 2H), 7.54 (t, J = 7.2 Hz, 1H), 7.44 (t, J = 7.2 Hz, 2H), 3.00 (q, J = 7.2 Hz, 2H), 1.22 (t, J = 7.2 Hz, 3H).

4-methoxybenzaldehyde (34b)

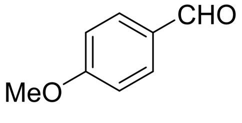

Prepared following the general procedure A employing 3-(4-methoxyphenyl)propanoic acid (96 mg, 0.5 mmol, 1.0 equiv.), [Ir(dF(CF_3)ppy)$_2$(dOMebpy)]PF_6 (16.7 mg, 0.015 mmol, 0.03 equiv.), Cu(OAc)$_2$ (18.1 mg, 0.1 mmol, 0.2 equiv.), 2,2'-bipyridine (19.5 mg, 0.125 mmol, 0.25 equiv.), Cs_2CO_3 (245 mg, 0.75 mmol, 1.5 equiv.) and DCM (10 mL) for 24h. The desired product was obtained as colorless oil (17 mg, 25% yield) after purification by flash column chromatography on silica gel (petroleum ether/ethyl acetate = 10/1). Data are consistent with those reported in the literature.[18]
^{1}H NMR (400 MHz, $CDCl_3$) δ 9.88 (s, 1H), 7.84 (d, J = 8.9 Hz, 2H), 7.00 (d, J = 8.7 Hz, 2H), 3.89 (s, 3H).

3,4-dimethoxybenzaldehyde (35b)

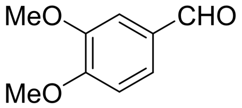

Prepared following the general procedure A employing 3-(3,4-dimethoxyphenyl)propanoic acid (105 mg, 0.5 mmol, 1.0 equiv.), [Ir(dF(CF$_3$)ppy)$_2$(dOMebpy)]PF$_6$ (16.7 mg, 0.015 mmol, 0.03 equiv.), Cu(OAc)$_2$ (18.1 mg, 0.1 mmol, 0.2 equiv.), 2,2'-bipyridine (19.5 mg, 0.125 mmol, 0.25 equiv.), Cs$_2$CO$_3$ (245 mg, 0.75 mmol, 1.5 equiv.) and DCM (10 mL) for 24h. The desired product was obtained as colorless oil (44 mg, 53% yield) after purification by flash column chromatography on silica gel (petroleum ether/ethyl acetate = 10/1). Data are consistent with those reported in the literature.[19]

^{1}H NMR (400 MHz, CDCl$_3$) δ 9.86 (s, 1H), 7.46 (d, J = 8.4 Hz, 1H), 7.41 (s, 1H), 6.98 (d, J = 8.4 Hz, 1H), 3.97 (s, 3H), 3.95 (s, 3H); HRMS (ESI) calcd for C$_{13}$H$_{11}$O [M + H]$^+$ m/z = 167.0703, found: 167.0708.

2,5-dimethoxybenzaldehyde (36b)

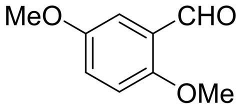

Prepared following the general procedure A employing 3-(2,5-dimethoxyphenyl)propanoic acid (105 mg, 0.5 mmol, 1.0 equiv.), [Ir(dF(CF$_3$)ppy)$_2$(dOMebpy)]PF$_6$ (16.7 mg, 0.015 mmol, 0.03 equiv.), Cu(OAc)$_2$ (18.1 mg, 0.1 mmol, 0.2 equiv.), 2,2'-bipyridine (19.5 mg, 0.125 mmol, 0.25 equiv.), Cs$_2$CO$_3$ (245 mg, 0.75 mmol, 1.5 equiv.) and DCM (10 mL) for 24h. The desired product was obtained as colorless oil (47 mg, 57% yield) after purification by flash column chromatography on silica gel (petroleum ether/ethyl acetate = 10/1). Data are consistent with those reported in the literature.[20]

^{1}H NMR (400 MHz, CDCl$_3$) δ 10.43 (s, 1H), 7.31 (d, J = 3.2 Hz, 1H), 7.12 (dd, J = 9.2, 3.2 Hz, 1H), 6.93 (d, J = 9.2 Hz, 1H), 3.88 (s, 3H), 3.78 (s, 3H); HRMS (ESI) calcd for C$_{13}$H$_{11}$O [M + H]$^+$ m/z = 167.0703, found: 167.0703.

heptane-2,6-dione (38b)

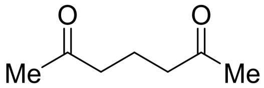

Prepared following the general procedure A employing compound **38a** (79 mg, 0.5 mmol, 1.0 equiv.), [Ir(dF(CF$_3$)ppy)$_2$(dOMebpy)]PF$_6$ (16.7 mg, 0.015 mmol, 0.03 equiv.), Cu(OAc)$_2$ (18.1 mg, 0.1 mmol, 0.2 equiv.), 2,2'-bipyridine (19.5 mg, 0.125 mmol, 0.25 equiv.), Cs$_2$CO$_3$ (245 mg, 0.75 mmol, 1.5 equiv.) and DCM (10 mL) for 48 hours. The desired product was obtained as colorless oil (54 mg, 85% yield) after purification by flash column chromatography on silica gel (petroleum ether/ethyl acetate = 10/1).

^{1}H NMR (400 MHz, CDCl$_3$) δ 2.38 (t, J = 7.1 Hz, 4H), 2.03 (s, 6H), 1.73 (p, J = 7.1 Hz, 2H); ^{13}C NMR (101 MHz, CDCl$_3$) δ 208.1, 42.2, 29.6, 17.4; HRMS (ESI) calcd for C$_7$H$_{13}$O$_2$ [M + H]$^+$ m/z = 129.0910, found: 129.0910.

1-phenylhexane-1,5-dione (39b)

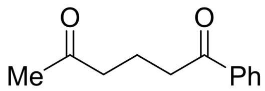

Prepared following the general procedure A employing compound **39a** (110 mg, 0.5 mmol, 1.0 equiv.), [Ir(dF(CF_3)ppy)$_2$(dOMebpy)]PF_6 (16.7 mg, 0.015 mmol, 0.03 equiv.), Cu(OAc)$_2$ (18.1 mg, 0.1 mmol, 0.2 equiv.), 2,2'-bipyridine (19.5 mg, 0.125 mmol, 0.25 equiv.), Cs_2CO_3 (245 mg, 0.75 mmol, 1.5 equiv.) and DCM (10 mL) for 48 hours. The desired product was obtained as colorless oil (71 mg, 75% yield) after purification by flash column chromatography on silica gel (petroleum ether/ethyl acetate = 10/1).

^{1}H NMR (400 MHz, $CDCl_3$) δ 7.96 (d, J = 7.4 Hz, 2H), 7.56 (t, J = 7.3 Hz, 1H), 7.46 (t, J = 7.6 Hz, 2H), 3.02 (t, J = 7.0 Hz, 2H), 2.57 (t, J = 7.0 Hz, 2H), 2.15 (s, 3H), 2.07 - 1.96 (m, 2H); ^{13}C NMR (101 MHz, $CDCl_3$) δ 208.5, 199.7, 136.8, 133.1, 128.6, 128.0, 42.6, 37.4, 29.9, 18.2; HRMS (ESI) calcd for $C_{12}H_{14}NaO_2$ $[M + Na]^+$ m/z = 213.0886, found: 213.0888.

4-(2-acetylphenyl) butan-2-one (40b)

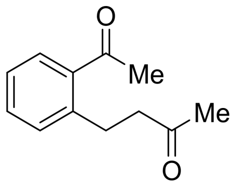

Prepared following the general procedure A employing compound **40a** (110 mg, 0.5 mmol, 1.0 equiv.), [Ir(dF(CF_3)ppy)$_2$(dOMebpy)]PF_6 (16.7 mg, 0.015 mmol, 0.03 equiv.), Cu(OAc)$_2$ (18.1 mg, 0.1 mmol, 0.2 equiv.), 2,2'-bipyridine (19.5 mg, 0.125 mmol, 0.25 equiv.), Cs_2CO_3 (245 mg, 0.75 mmol, 1.5 equiv.) and DCM (10 mL) for 48 hours. The desired product was obtained as colorless oil (66 mg, 70% yield) after purification by flash column chromatography on silica gel (petroleum ether/ethyl acetate = 10/1).

^{1}H NMR (400 MHz, $CDCl_3$) δ 7.62 (d, J = 7.5 Hz, 1H), 7.31 (t, J = 7.3 Hz, 1H), 7.19 (t, J = 7.8 Hz, 2H), 2.98 (t, J = 7.4 Hz, 2H), 2.68 (t, J = 7.4 Hz, 2H), 2.50 (s, 3H), 2.05 (s, 3H); ^{13}C NMR (101 MHz, $CDCl_3$) δ 208.1, 201.5, 141.3, 137.2, 131.6, 131.4, 129.5, 126.1, 45.2, 29.6, 29.4, 28.5; HRMS (ESI) calcd for $C_{12}H_{14}NaO_2$ $[M + Na]^+$ m/z = 213.0886, found: 213.0887.

2-(3-oxobutyl) benzaldehyde (41b)

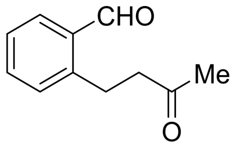

Prepared following the general procedure A employing compound **41a** (103 mg, 0.5 mmol, 1.0 equiv.), [Ir(dF(CF_3)ppy)$_2$(dOMebpy)]PF_6 (16.7 mg, 0.015 mmol, 0.03 equiv.), Cu(OAc)$_2$ (18.1 mg, 0.1 mmol, 0.2 equiv.), 2,2'-bipyridine (19.5 mg, 0.125 mmol, 0.25 equiv.), Cs_2CO_3 (245 mg, 0.75 mmol, 1.5 equiv.) and DCM (10 mL) for 48 hours. The desired product was obtained as colorless oil (53 mg, 60% yield) after purification by flash column chromatography on silica gel (petroleum ether/ethyl acetate = 10/1).

^1H NMR (400 MHz, $CDCl_3$) δ 10.19 (s, 1H), 7.80 (d, J = 7.5 Hz, 1H), 7.51 (t, J = 7.4 Hz, 1H), 7.41 (t, J = 7.0 Hz, 1H), 7.32 (d, J = 8.0 Hz, 1H), 3.28 (t, J = 7.6 Hz, 2H), 2.76 (t, J = 7.6 Hz, 2H), 2.15 (s, 3H); ^{13}C NMR (176 MHz, $CDCl_3$) δ 207.7, 192.9, 143.5, 133.9, 133.8, 131.4, 126.9, 44.9, 29.9, 27.2; HRMS (ESI) calcd for $C_{11}H_{12}NaO_2$ [M + Na]$^+$ m/z =199.0730, found: 199.0731.

2-(3-oxo-3-phenylpropyl) benzaldehyde (42b)

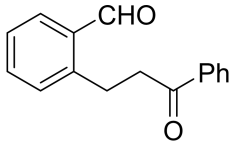

Prepared following the general procedure A employing compound **42a** (134 mg, 0.5 mmol, 1.0 equiv.), [Ir(dF(CF_3)ppy)$_2$(dOMebpy)]PF_6 (16.7 mg, 0.015 mmol, 0.03 equiv.), $Cu(OAc)_2$ (18.1 mg, 0.1 mmol, 0.2 equiv.), 2,2'-bipyridine (19.5 mg, 0.125 mmol, 0.25 equiv.), Cs_2CO_3 (245 mg, 0.75 mmol, 1.5 equiv.) and DCM (10 mL) for 48 hours. The desired product was obtained as colorless oil (59 mg, 50% yield) after purification by flash column chromatography on silica gel (petroleum ether/ethyl acetate = 10/1).
^1H NMR (400 MHz, $CDCl_3$) δ 10.24 (s, 1H), 7.97 (d, J = 7.3 Hz, 2H), 7.83 (d, J = 7.3 Hz, 1H), 7.54 (dd, J = 16.4, 7.5 Hz, 2H), 7.48 - 7.38 (m, 4H), 3.47 (t, J = 7.4 Hz, 2H), 3.32 (t, J = 7.3 Hz, 2H); ^{13}C NMR (176 MHz, $CDCl_3$) δ 199.0, 192.9, 143.7, 136.8, 133.9, 133.7, 133.1, 131.6, 128.6, 128.1, 126.9, 40.1, 27.6; HRMS (ESI) calcd for $C_{16}H_{14}NaO_2$ [M + Na]$^+$ m/z =261.0886, found: 261.0882.

tert-butyl (3-oxobutyl) (2-oxopropyl) carbamate (43b) and
tert-butyl formyl(3-hydroxy-3-methyl-4-oxopentyl) carbamate (43c)

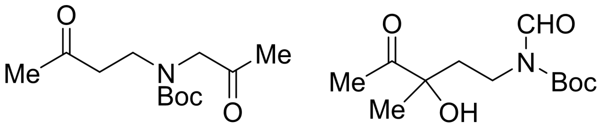

Prepared following the general procedure A employing compound **43a** (137 mg, 0.5 mmol, 1.0 equiv.), [Ir(dF(CF_3)ppy)$_2$(dOMebpy)]PF_6 (16.7 mg, 0.015 mmol, 0.03 equiv.), $Cu(OAc)_2$ (18.1 mg, 0.1 mmol, 0.2 equiv.), 2,2'-bipyridine (19.5 mg, 0.125 mmol, 0.25 equiv.), Cs_2CO_3 (245 mg, 0.75 mmol, 1.5 equiv.) and DCM (10 mL) for 48 hours. The desired products were obtained as colorless oil (**43b**, 100 mg, 75% yield; **43c**, 30 mg, 23%) after purification by flash column chromatography on silica gel (petroleum ether/ethyl acetate = 10/1).
Data for **43b**:
^1H NMR (400 MHz, $CDCl_3$) δ 4.04 (d, J = 18.4 Hz, 2H), 3.44 (dt, J = 12.0, 6.0 Hz, 2H), 2.76 (dd, J = 16.4, 10.5 Hz, 2H), 2.10 (dd, J = 22.3, 9.4 Hz, 6H), 1.41 (d, J = 32.8 Hz, 9H); ^{13}C NMR (176 MHz, $CDCl_3$) δ 208.3, 207.7, 204.3, 204.2, 155.5, 155.2, 80.3, 80.1, 59.2, 58.5, 43.6, 43.5, 43.2, 42.8, 30.2, 30.1, 28.3, 28.1, 26.8, 26.5; HRMS (ESI) calcd for $C_{12}H_{21}KNO_4$ [M + K]$^+$ m/z = 282.1102, found: 282.1126; IR ν_{max}/cm^{-1} (film) : 3446, 2961, 2927, 1651, 1363, 1080, 966, 761.
Data for **43c**:
^1H NMR (400 MHz, $CDCl_3$) δ 9.09 (s, 1H), 3.95 (s, 1H), 3.76 - 3.65 (m, 1H), 3.45 (m, 1H), 2.28 (s, 3H), 2.02 (ddd, J = 15.6, 9.9, 5.9 Hz, 1H), 1.88 (ddd, J = 13.6, 10.0, 5.1 Hz, 1H), 1.54 (s, 9H), 1.36 (s, 3H). 13C NMR (176 MHz, CDCl3) δ 211.8, 163.1, 152.1, 84.4, 77.7, 36.7, 36.1, 28.0,

25.6, 23.6. HRMS (ESI) calcd for $C_{12}H_{21}NaO_5$ [M + Na] $^+$ m/z = 282.1312, found: 282.1314; IR ν_{max}/cm^{-1} (film) : 3467, 2962, 2316, 1654, 1630, 1079, 965, 704.

4-hydroxy-1-phenylbutan-1-one (44b)

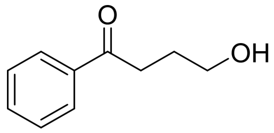

Prepared following the general procedure B employing 1-phenylcyclobutane-1-carboxylic acid (88 mg, 0.5 mmol, 1.0 equiv.), [(dF(CF_3)ppy)$_2$-Ir- -Cl]$_2$ (16.7 mg, 0.015 mmol, 0.03 equiv.), Cu(OAc)$_2$ (18.1 mg, 0.1 mmol, 0.2 equiv.), 2,2'-bipyridine (19.5 mg, 0.125 mmol, 0.25 equiv.), Selectfluor (265 mg, 0.75 mmol, 1.5 equiv.), Cs_2CO_3 (245 mg, 0.75 mmol, 1.5 equiv.) and DCM (10 mL) for 40 hours. The desired product was obtained as colorless oil (75% yield determined by ^{1}H NMR) after purification by flash column chromatography on silica gel (petroleum ether/ethyl acetate = 4/1). Data are consistent with those reported in the literature.[9]

^{1}H NMR (400 MHz, $CDCl_3$) δ 7.99 (d, J = 7.6 Hz, 2H), 7.57 (t, J = 7.6 Hz, 1H), 7.47 (t, J = 7.6 Hz, 2H), 3.76 (t, J = 6.0 Hz, 2H), 3.15 (t, J = 6.8 Hz, 2H), 2.07 - 2.00 (m, 2H); ^{13}C NMR (101 MHz, $CDCl_3$) δ 200.7, 137.0, 133.3, 128.8, 128.2, 62.5, 35.4, 27.0; HRMS (ESI) calcd for $C_{10}H_{12}NaO_2$ [M+Na]$^+$ m/z = 187.0730, found: 187.0730.

1-(4-fluorophenyl)-4-hydroxybutan-1-one (45b)

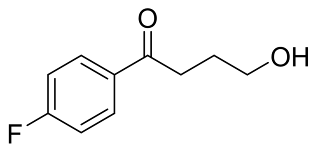

Prepared following the general procedure B employing 1-(4-fluorophenyl)cyclobutane-1-carboxylic acid (97 mg, 0.5 mmol, 1.0 equiv.), [(dF(CF_3)ppy)$_2$-Ir- -Cl]$_2$ (16.7 mg, 0.015 mmol, 0.03 equiv.), Cu(OAc)$_2$ (18.1 mg, 0.1 mmol, 0.2 equiv.), 2,2'-bipyridine (19.5 mg, 0.125 mmol, 0.25 equiv.), Selectfluor (265 mg, 0.75 mmol, 1.5 equiv.), Cs_2CO_3 (245 mg, 0.75 mmol, 1.5 equiv.) and DCM (10 mL) for 40 hours. The desired product was obtained as colorless oil (67 mg, 80% yield) after purification by flash column chromatography on silica gel (petroleum ether/ethyl acetate = 4/1). Data are consistent with those reported in the literature.[21]

^{1}H NMR (400 MHz, $CDCl_3$) δ 8.04 - 7.95 (m, 2H), 7.14 (t, J = 8.4 Hz, 2H), 3.75 (t, J = 6.0 Hz, 2H), 3.11 (t, J = 6.9 Hz, 2H), 2.07 - 1.95 (m, 2H); ^{13}C NMR (101 MHz, $CDCl_3$) δ 199.0, 165.9 (d, J = 146 Hz), 133.4, 130.9 (d, J = 5.2 Hz), 115.9 (d, J = 12.4 Hz), 62.4, 35.3, 27.0; HRMS (ESI) calcd for $C_{10}H_{13}FNaO_2$ [M+Na]$^+$ m/z = 205.0635, found: 205.0634.

1-(4-bromophenyl)-4-hydroxybutan-1-one (46b)

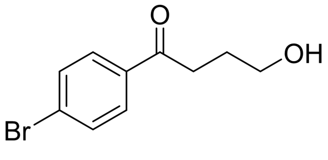

Prepared following the general procedure B employing

1-(4-bromophenyl)cyclobutane-1-carboxylic acid (127 mg, 0.5 mmol, 1.0 equiv.), [(dF(CF$_3$)ppy)$_2$-Ir- -Cl]$_2$ (16.7 mg, 0.015 mmol, 0.03 equiv.), Cu(OAc)$_2$ (18.1 mg, 0.1 mmol, 0.2 equiv.), 2,2'-bipyridine (19.5 mg, 0.125 mmol, 0.25 equiv.), Selectfluor (265 mg, 0.75 mmol, 1.5 equiv.), Cs_2CO_3 (245 mg, 0.75 mmol, 1.5 equiv.) and DCM (10 mL) for 40 hours. The desired product was obtained as colorless oil (73% yield determined by ^{1}H NMR) after purification by flash column chromatography on silica gel (petroleum ether/ethyl acetate = 4/1). Data are consistent with those reported in the literature.[21]

^{1}H NMR (400 MHz, $CDCl_3$) δ 7.84 (d, J = 8.6 Hz, 2H), 7.60 (d, J = 8.6 Hz, 2H), 3.74 (t, J = 6.0 Hz, 2H), 3.10 (t, J = 6.9 Hz, 2H), 2.05 - 1.98 (m, 2H); ^{13}C NMR (101 MHz, $CDCl_3$) δ 199.5, 135.7, 132.1, 129.8, 128.5, 62.4, 35.3, 26.9; HRMS (ESI) calcd for $C_{10}H_{13}BrNaO_2$ $[M+Na]^+$ m/z = 264.9835, found: 264.9838.

5-hydroxy-1-phenylpentan-1-one (47b)

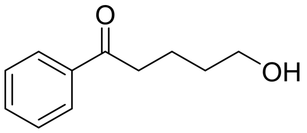

Prepared following the general procedure B employing 1-phenylcyclopentane-1-carboxylic acid (95 mg, 0.5 mmol, 1.0 equiv.), [(dF(CF$_3$)ppy)$_2$-Ir- -Cl]$_2$ (16.7 mg, 0.015 mmol, 0.03 equiv.), Cu(OAc)$_2$ (18.1 mg, 0.1 mmol, 0.2 equiv.), 2,2'-bipyridine (19.5 mg, 0.125 mmol, 0.25 equiv.), Selectfluor (265 mg, 0.75 mmol, 1.5 equiv.), Cs_2CO_3 (245 mg, 0.75 mmol, 1.5 equiv.) and DCM (10 mL) for 40 hours. The desired product was obtained as colorless oil (70% yield determined by ^{1}H NMR) after purification by flash column chromatography on silica gel (petroleum ether/ethyl acetate = 4/1). Data are consistent with those reported in the literature.[21]

^{1}H NMR (400 MHz, $CDCl_3$) δ 7.97 (d, J = 7.2 Hz, 2H), 7.57 (t, J = 7.3 Hz, 1H), 7.47 (t, J = 7.6 Hz, 2H), 3.68 (t, J = 6.3 Hz, 2H), 3.04 (t, J = 7.1 Hz, 2H), 1.90 - 1.81 (m, 2H), 1.72 - 1.62 (m, 2H); ^{13}C NMR (101 MHz, $CDCl_3$) δ 200.5, 137.1, 133.2, 128.8, 128.2, 62.6, 38.2, 32.4, 20.3; HRMS (ESI) calcd for $C_{11}H_{14}KO_2$ $[M+K]^+$ m/z = 217.0625, found: 217.0629.

6-hydroxy-1-phenylhexan-1-one (48b)

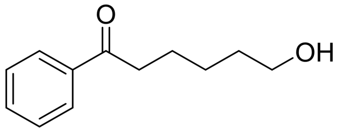

Prepared following the general procedure B employing 1-phenylcyclohexane-1-carboxylic acid (102 mg, 0.5 mmol, 1.0 equiv.), [(dF(CF$_3$)ppy)$_2$-Ir- -Cl]$_2$ (16.7 mg, 0.015 mmol, 0.03 equiv.), Cu(OAc)$_2$ (18.1 mg, 0.1 mmol, 0.2 equiv.), 2,2'-bipyridine (19.5 mg, 0.125 mmol, 0.25 equiv.), Selectfluor (265 mg, 0.75 mmol, 1.5 equiv.), Cs_2CO_3 (245 mg, 0.75 mmol, 1.5 equiv.) and DCM (10 mL) for 40 hours. The desired product was obtained as colorless oil (72 mg, 75% yield) after purification by flash column chromatography on silica gel (petroleum ether/ethyl acetate = 4/1). Data are consistent with those reported in the literature.[22]

^{1}H NMR (400 MHz, $CDCl_3$) δ 7.96 (d, J = 9.0 Hz, 2H), 7.56 (t, J = 7.3 Hz, 1H), 7.46 (t, J = 7.7 Hz, 2H), 3.68 (t, J = 6.5 Hz, 2H), 3.00 (t, J = 7.3 Hz, 2H), 1.82 - 1.73 (m, 2H), 1.67 - 1.58 (m, 2H), 1.52 - 1.41 (m, 2H); ^{13}C NMR (101 MHz, $CDCl_3$) δ 200.6, 137.1, 133.1, 128.7, 128.2, 62.8, 38.6,

32.6, 25.6, 24.0; HRMS (ESI) calcd for $C_{12}H_{16}NaO_2$ $[M+Na]^+$ m/z =215.1043, found: 215.1042.

3-(2-hydroxyethoxy)-1-phenylpropan-1-one (49b)

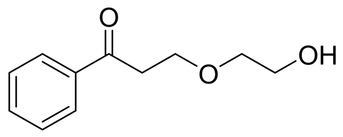

Prepared following the general procedure B employing **49a** (103 mg, 0.5 mmol, 1.0 equiv.), $[(dF(CF_3)ppy)_2$-Ir- -Cl$]_2$ (16.7 mg, 0.015 mmol, 0.03 equiv.), $Cu(OAc)_2$ (18.1 mg, 0.1 mmol, 0.2 equiv.), 2,2'-bipyridine (19.5 mg, 0.125 mmol, 0.25 equiv.), Selectfluor (265 mg, 0.75 mmol, 1.5 equiv.), Cs_2CO_3 (245 mg, 0.75 mmol, 1.5 equiv.) and DCM (10 mL) for 40 hours. The desired product was obtained as colorless oil (77 mg, 80% yield) after purification by flash column chromatography on silica gel (petroleum ether/ethyl acetate = 4/1). Data are consistent with those reported in the literature.[23]

^{1}H NMR (400 MHz, $CDCl_3$) δ 7.98 (d, J = 7.1 Hz, 2H), 7.58 (t, J = 7.6 Hz, 1H), 7.48 (t, J = 7.5 Hz, 2H), 3.94 (t, J = 6.0 Hz, 2H), 3.77 - 3.70 (m, 2H), 3.66 - 3.59 (m, 2H), 3.28 (t, J = 6.2 Hz, 2H); ^{13}C NMR (101 MHz, $CDCl_3$) δ 198.6, 137.0, 133.5, 128.8, 128.3, 72.2, 65.9, 61.8, 38.8; HRMS (ESI) calcd for $C_{11}H_{14}NaO_3$ $[M+Na]^+$ m/z =217.0835, found: 217.0836.

1-(4-fluorophenyl)-3-(2-hydroxyethoxy)propan-1-one (50b)

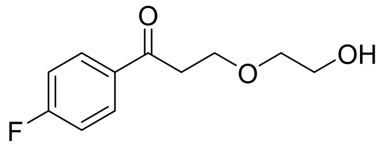

Prepared following the general procedure B employing **50a** (112 mg, 0.5 mmol, 1.0 equiv.), $[(dF(CF_3)ppy)_2$-Ir- -Cl$]_2$ (16.7 mg, 0.015 mmol, 0.03 equiv.), $Cu(OAc)_2$ (18.1 mg, 0.1 mmol, 0.2 equiv.), 2,2'-bipyridine (19.5 mg, 0.125 mmol, 0.25 equiv.), Selectfluor (265 mg, 0.75 mmol, 1.5 equiv.), Cs_2CO_3 (245 mg, 0.75 mmol, 1.5 equiv.) and DCM (10 mL) for 40 hours. The desired product was obtained as colorless oil (75 mg, 73% yield) after purification by flash column chromatography on silica gel (petroleum ether/ethyl acetate = 4/1).

^{1}H NMR (400 MHz, $CDCl_3$) δ 8.00 (dd, J = 8.7, 5.5 Hz, 2H), 7.14 (t, J = 8.5 Hz, 2H), 3.93 (t, J = 6.1 Hz, 2H), 3.77 - 3.71 (m, 2H), 3.64 - 3.58 (m, 2H), 3.24 (t, J = 6.1 Hz, 2H); ^{13}C NMR (101 MHz, $CDCl_3$) δ 197.0, 166.0 (d, J = 146 Hz), 133.5 (d, J = 1.7 Hz), 131.0 (d, J = 5.3 Hz), 115.9 (d, J = 12.5 Hz), 72.2, 65.9, 61.8, 38.7; HRMS (ESI) calcd for $C_{11}H_{13}FNaO_3$ $[M+Na]^+$ m/z = 235.0741, found: 235.0740; IR v_{max}/cm^{-1} (film) : 2971, 1735, 1701, 1370, 1340, 1152, 1024, 853, 777.

1-(4-chlorophenyl)-3-(2-hydroxyethoxy)propan-1-one (51b)

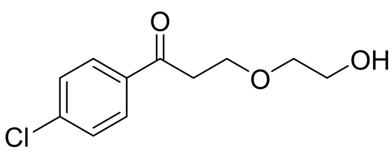

Prepared following the general procedure B employing **51a** (120 mg, 0.5 mmol, 1.0 equiv.), $[(dF(CF_3)ppy)_2$-Ir- -Cl$]_2$ (16.7 mg, 0.015 mmol, 0.03 equiv.), $Cu(OAc)_2$ (18.1 mg, 0.1 mmol, 0.2

equiv.), 2,2'-bipyridine (19.5 mg, 0.125 mmol, 0.25 equiv.), Selectfluor (265 mg, 0.75 mmol, 1.5 equiv.), Cs_2CO_3 (245 mg, 0.75 mmol, 1.5 equiv.) and DCM (10 mL) for 40 hours. The desired product was obtained as colorless oil (80 mg, 70% yield) after purification by flash column chromatography on silica gel (petroleum ether/ethyl acetate = 4/1).

^{1}H NMR (400 MHz, $CDCl_3$) δ 7.91 (d, J = 8.5 Hz, 2H), 7.45 (d, J = 8.4 Hz, 2H), 3.93 (t, J = 6.0 Hz, 2H), 3.76 - 3.71 (m, 2H), 3.64 - 3.59 (m, 2H), 3.24 (t, J = 6.0 Hz, 2H); ^{13}C NMR (101 MHz, $CDCl_3$) δ 197.4, 140.0, 135.3, 129.7, 129.1, 72.2, 65.8, 61.8, 38.7; HRMS (ESI) calcd for $C_{11}H_{13}ClNaO_3$ [M+Na]$^+$ m/z = 251.0445, found: 251.0442; IR ν_{max}/cm^{-1} (film) : 2926, 1735, 1718, 1684, 1339, 1261, 762.

3-(2-hydroxyethoxy)-1-(4-methoxyphenyl)propan-1-one (52b)

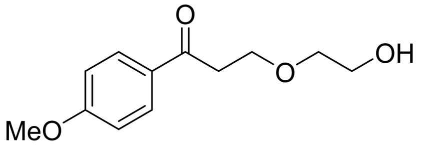

Prepared following the general procedure B employing **52a** (118 mg, 0.5 mmol, 1.0 equiv.), [(dF(CF_3)ppy)$_2$-Ir-   -Cl]$_2$ (16.7 mg, 0.015 mmol, 0.03 equiv.), $Cu(OAc)_2$ (18.1 mg, 0.1 mmol, 0.2 equiv.), 2,2'-bipyridine (19.5 mg, 0.125 mmol, 0.25 equiv.), Selectfluor (265 mg, 0.75 mmol, 1.5 equiv.), Cs_2CO_3 (245 mg, 0.75 mmol, 1.5 equiv.) and DCM (10 mL) for 40 hours. The desired product was obtained as colorless oil (56 mg, 50% yield) after purification by flash column chromatography on silica gel (petroleum ether/ethyl acetate = 4/1). Data are consistent with those reported in the literature.[24]

^{1}H NMR (400 MHz, $CDCl_3$) δ 7.96 (d, J = 8.7 Hz, 2H), 6.94 (d, J = 8.7 Hz, 2H), 3.93 (t, J = 6.2 Hz, 2H), 3.88 (s, 3H), 3.77 - 3.71 (m, 2H), 3.64 - 3.59 (m, 2H), 3.22 (t, J = 6.1 Hz, 2H); ^{13}C NMR (101 MHz, $CDCl_3$) δ 197.1, 163.8, 130.6, 130.2, 113.9, 72.2, 66.1, 61.8, 55.6, 38.4; HRMS (ESI) calcd for $C_{12}H_{16}NaO_4$, [M+Na]$^+$ m/z = 247.0941, found: 247.0940.

1-([1,1'-biphenyl]-4-yl)-3-(2-hydroxyethoxy)propan-1-one (53b)

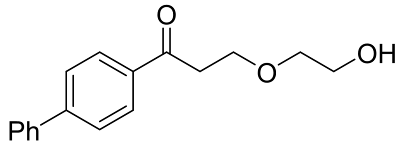

Prepared following the general procedure B employing **53a** (141 mg, 0.5 mmol, 1.0 equiv.), [(dF(CF_3)ppy)$_2$-Ir-   -Cl]$_2$ (16.7 mg, 0.015 mmol, 0.03 equiv.), $Cu(OAc)_2$ (18.1 mg, 0.1 mmol, 0.2 equiv.), 2,2'-bipyridine (19.5 mg, 0.125 mmol, 0.25 equiv.), Selectfluor (265 mg, 0.75 mmol, 1.5 equiv.), Cs_2CO_3 (245 mg, 0.75 mmol, 1.5 equiv.) and DCM (10 mL) for 40 hours. The desired product was obtained as colorless oil (77 mg, 68% yield) after purification by flash column chromatography on silica gel (petroleum ether/ethyl acetate = 4/1).

^{1}H NMR (400 MHz, $CDCl_3$) δ 8.05 (d, J = 8.3 Hz, 2H), 7.70 (d, J = 8.3 Hz, 2H), 7.63 (d, J = 7.3 Hz, 2H), 7.47 (d, J = 7.7 Hz, 2H), 7.41 (t, J = 7.2 Hz, 1H), 3.96 (t, J = 6.1 Hz, 2H), 3.77 - 3.73 (m, 2H), 3.66 - 3.61 (m, 2H), 3.31 (t, J = 6.1 Hz, 2H); ^{13}C NMR (101 MHz, $CDCl_3$) δ 198.2, 146.2, 139.9, 135.7, 129.1, 128.9, 128.4, 127.4, 127.4, 72.2, 66.0, 61.8, 38.8; HRMS (ESI) calcd for $C_{17}H_{18}NaO_3$ [M+Na]$^+$ m/z = 293.1148, found: 293.1148; IR ν_{max}/cm^{-1} (film) : 2962, 1685, 1458, 1261, 1093, 799.

4-(2-acetylphenyl)-4-(3,4-dichlorophenyl) butan-2-one (54b)

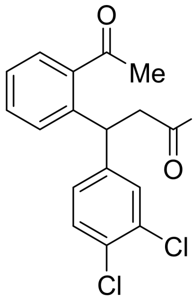

Prepared following the general procedure A employing **54a** (183 mg, 0.5 mmol, 1.0 equiv.), [Ir(dF(CF_3)ppy)$_2$(dOMebpy)]PF_6 (16.7 mg, 0.015 mmol, 0.03 equiv.), $Cu(OAc)_2$ (18.1 mg, 0.1 mmol, 0.2 equiv.), 2,2'-bipyridine (19.5 mg, 0.125 mmol, 0.25 equiv.), Cs_2CO_3 (245 mg, 0.75 mmol, 1.5 equiv.) and DCM (10 mL) for 48 hours. The desired product was obtained as colorless oil (138 mg, 83% yield) after purification by flash column chromatography on silica gel (petroleum ether/ethyl acetate = 10/1).

^{1}H NMR (400 MHz, $CDCl_3$) δ 7.60 (dd, J = 7.7, 1.2 Hz, 1H), 7.39 (m, 1H), 7.36 (d, J = 2.1 Hz, 1H), 7.33 (d, J = 8.3 Hz, 1H), 7.27 (m, J = 7.6, 1.2 Hz, 1H), 7.20 (dd, J = 7.8 Hz, 1H), 7.16 (dd, J = 8.4, 2.1 Hz, 1H), 5.29 (t, J = 7.5 Hz, 1H), 3.21 (dd, J = 17.1, 8.0 Hz, 1H), 3.08 (dd, J = 17.1, 7.0 Hz, 1H), 2.59 (s, 3H), 2.10 (s, 3H); ^{13}C NMR (101 MHz, $CDCl_3$) δ 205.8, 202.9, 143.8, 142.1, 138.8, 132.3, 131.4, 130.3, 130.3, 129.9, 128.6, 128.5, 127.7, 126.4, 49.5, 39.6, 30.0, 29.9; HRMS (ESI) calcd for $C_{18}H_{16}NaO_2$ [M + Na]$^+$ m/z =357.0470, found: 357.0473; IR ν_{max}/cm^{-1} (film) : 3064, 2963, 1716, 1687, 1470, 1357, 1251, 1029, 960, 814, 759, 597.

(3aS,5aS,6R,7R,9aS,9bS)-3a,6-dimethyl-6-(3-oxo-3-phenylpropyl)dodecahydro-1H-cyclopenta[a]naphthalene-7-carbaldehyde (55b)

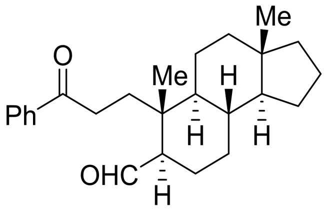

Prepared following the general procedure B employing **55a** (190 mg, 0.5 mmol, 1.0 equiv.), [(dF(CF_3)ppy)$_2$-Ir- -Cl]$_2$ (16.7 mg, 0.015 mmol, 0.03 equiv.), $Cu(OAc)_2$ (18.1 mg, 0.1 mmol, 0.2 equiv.), 2,2'-bipyridine (19.5 mg, 0.125 mmol, 0.25 equiv.), Selectfluor (265 mg, 0.75 mmol, 1.5 equiv.), Cs_2CO_3 (245 mg, 0.75 mmol, 1.5 equiv.) and DCM (10 mL) for 40 hours. The desired product was obtained as colorless oil (40 mg, 22% yield, brms 50%) after purification by flash column chromatography on silica gel (petroleum ether/ethyl acetate = 10/1).

^{1}H NMR (400 MHz, $CDCl_3$) δ 9.76 (d, J = 3.8 Hz, 1H), 8.02 - 7.93 (m, 2H), 7.57 (t, J = 7.3 Hz, 1H), 7.48 (t, J = 7.4 Hz, 2H), 3.18 (ddd, J = 16.6, 12.1, 4.9 Hz, 1H), 2.77 (ddd, J = 16.2, 12.0, 4.2 Hz, 1H), 2.28 (d, J = 12.1 Hz, 1H), 2.01 - 1.93 (m, 1H), 1.84 (t, J = 11.7 Hz, 1H), 1.77 (dd, J = 7.8, 4.5 Hz, 1H), 1.73 - 1.64 (m, 5H), 1.47 (s, 1H), 1.36 (d, J = 11.8 Hz, 1H), 1.25 (s, 1H), 1.19 - 1.13 (m, 3H), 1.08 (s, 3H), 1.01 - 0.95 (m, 2H), 0.90 (d, J = 16.5 Hz, 2H), 0.77 (d, J = 8.8 Hz, 1H), 0.71 (s, 3H); ^{13}C NMR (176 MHz, $CDCl_3$) δ 206.1, 200.3, 136.9, 133.1, 128.6, 128.2, 55.6, 54.5,

47.9, 40.7, 40.3, 38.5, 38.3, 35.3, 32.4, 31.6, 30.7, 25.5, 22.0, 20.5, 20.1, 17.5, 17.4; HRMS (ESI) calcd for $C_{25}H_{34}NaO_2$ [M + Na] $^+$ m/z = 389.2451, found: 389.2450; IR ν_{max}/cm^{-1} (film): 3468, 2829, 2026, 1656, 1631, 1355, 1276, 722, 697, 667.

(3R,3aR,5aS,6R,7R,9aS,9bS)-3a,6-dimethyl-3-((R)-6-methylheptan-2-yl)-6-(3-oxo-3-phenylpropyl)dodecahydro-1H-cyclopenta[a]naphthalene-7-carbaldehyde (56b)

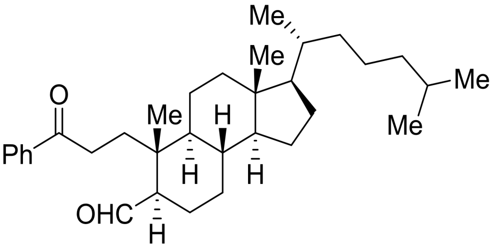

Prepared following the general procedure B employing **56a** (246 mg, 0.5 mmol, 1.0 equiv.), [(dF(CF$_3$)ppy)$_2$-Ir- -Cl]$_2$ (16.7 mg, 0.015 mmol, 0.03 equiv.), Cu(OAc)$_2$ (18.1 mg, 0.1 mmol, 0.2 equiv.), 2,2'-bipyridine (19.5 mg, 0.125 mmol, 0.25 equiv.), Selectfluor (265 mg, 0.75 mmol, 1.5 equiv.), Cs_2CO_3 (245 mg, 0.75 mmol, 1.5 equiv.) and DCM (10 mL) for 40 hours. The desired product was obtained as colorless oil (42 mg, 18% yield， brms 35%) after purification by flash column chromatography on silica gel (petroleum ether/ethyl acetate = 10/1).

^{1}H NMR (400 MHz, $CDCl_3$) δ 9.88 (s, 1H), 7.93 (d, J = 7.6 Hz, 2H), 7.54 (t, J = 7.3 Hz, 1H), 7.45 (t, J = 7.5 Hz, 2H), 3.20 - 3.10 (m, 1H), 2.73 (dd, J = 16.0, 9.6 Hz, 1H), 2.52 - 2.43 (m, 1H), 2.32 (dd, J = 15.2, 3.2 Hz, 2H), 1.98 (d, J = 13.0 Hz, 1H), 1.79 (s, 2H), 1.31 - 1.23 (m, 9H), 1.17 (d, J = 12.6 Hz, 3H), 1.13 - 1.04 (m, 6H), 0.97 (s, 3H), 0.92 (s, 3H), 0.87 (d, J = 6.5 Hz, 3H), 0.85 (d, J = 1.9 Hz, 3H), 0.83 (d, J = 1.9 Hz, 3H), 0.64 (s, 3H). ^{13}C NMR (176 MHz, $CDCl_3$) δ 203.4, 200.2, 137.3, 133.1, 128.7, 128.2, 56.3, 56.1, 50.1, 49.9, 42.3, 40.5, 40.2, 39.7, 39.5, 36.1, 35.8, 35.3, 31.4, 29.7, 28.2, 28.2, 28.0, 24.2, 23.8, 22.8, 22.6, 22.0, 18.6, 15.7, 11.9; HRMS (ESI) calcd for $C_{33}H_{50}NaO_2$ [M + Na] $^+$ m/z = 501.3703, found: 501.3709; IR ν_{max}/cm^{-1} (film): 3451, 2892, 2026, 1637, 1356, 1260, 1077, 773, 696, 667, 606.

General procedure C for the reaction

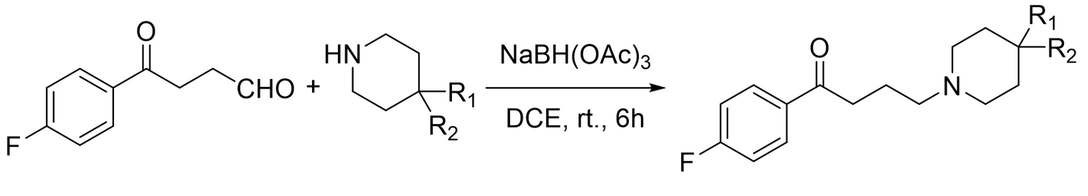

A solution of **12b** (101 mg, 0.525 mmol), which obtained followed procedure A and the corresponding piperazine derivate (1.04 equiv.) in anhydrous DCE (5 mL) was added Sodium triacetoxyborohydride (212 mg, 1.0 mmol, 2.0 equiv.) at room temperature under an atmosphere of argon. The resulting mixture allowed to stir for 6 hours. The reaction mixture was quenched with sat. $NaHCO_3$ and extracted by DCM (3*20 mL). The combined organic layers were dried Na_2SO_4, filtered and concentrated in vacuo to afford the crude product. Purification by flash column chromatography afforded the desired product.

1-(4-fluorophenyl)-4-(piperidin-1-yl) butan-1-one (Primaperone)

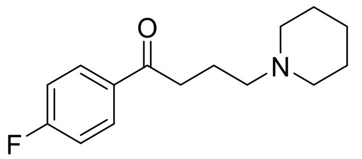

Prepared following the general procedure C employing **12b** (90 mg, 0.5 mmol, 1.0 equiv.), piperidine (44 mg, 0.52 mmol, 1.04 equiv.), sodium triacetoxyborohydride (212 mg, 1.0 mmol, 2.0 equiv.), and dry DCE (5 mL) for 6 hours. The desired product was obtained as white solid (106 mg, 85% yield) after purification by flash column chromatography on silica gel (petroleum ether/ethyl acetate = 5/1).

^{1}H NMR (400 MHz, $CDCl_3$) δ 7.97 - 7.91 (m, 2H), 7.09 - 7.00 (m, 2H), 2.90 (t, J = 7.2 Hz, 2H), 2.44 - 2.13 (m, 6H), 1.94 - 1.80 (m, 2H), 1.50 - 1.44 (m, 4H), 1.38 - 1.32 (m, 2H); ^{13}C NMR (101 MHz, $CDCl_3$) δ 198.4, 166.7, 164.2, 133.5, 133.4, 130.6, 130.5, 115.5, 115.3, 58.3, 54.3, 36.2, 25.7, 24.3, 21.5; HRMS (ESI) calcd for $C_{15}H_{21}FNO$ [M + H]$^+$ m/z = 250.1602, found: 250.1600.

1-(4-fluorophenyl)-4-(4-methylpiperidin-1-yl) butan-1-one (Melperone)

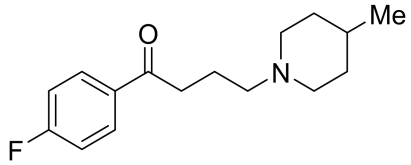

Prepared following the general procedure C employing **12b** (90 mg, 0.5 mmol, 1.0 equiv.), 4-methylpiperidine (47 mg, 0.52 mmol, 1.04 equiv.), sodium triacetoxyborohydride (212 mg, 1.0 mmol, 2.0 equiv.), and dry DCE (5 mL) for 6 hours. The desired product was obtained as white solid (109 mg, 83% yield) after purification by flash column chromatography on silica gel (petroleum ether/ethyl acetate = 5/1).

^{1}H NMR (400 MHz, $CDCl_3$) δ 7.98 - 7.91 (m, 2H), 7.11 - 7.02 (m, 2H), 2.90 (t, J = 7.2 Hz, 2H), 2.81 (d, J = 11.6 Hz, 2H), 2.33 (dd, J = 13.6, 6.5 Hz, 2H), 1.93 - 1.81 (m, 4H), 1.57 - 1.48 (m, 2H), 1.31-1.22 (m, 1H), 1.11 (ddd, J = 15.5, 12.2, 3.7 Hz, 2H), 0.84 (d, J = 6.5 Hz, 3H); ^{13}C NMR (101 MHz, $CDCl_3$) δ 198.3, 166.8, 164.3, 133.4, 133.3, 130.6, 130.5, 115.6, 115.3, 57.7, 53.6, 36.1, 33.6, 30.5, 21.6, 21.2; HRMS (ESI) calcd for $C_{16}H_{23}FNO$ [M + H]$^+$ m/z = 264.1758, found: 264.1753.

4-(4-(4-chlorophenyl)-4-hydroxypiperidin-1-yl)-1-(4-fluorophenyl) butan-1-one (Haloperidol)

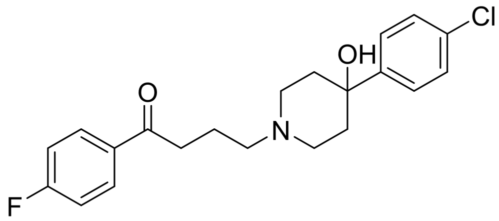

Prepared following the general procedure C employing **12b** (90 mg, 0.5 mmol, 1.0 equiv.), 4-(4-chlorophenyl) piperidin-4-ol (110 mg, 0.52 mmol, 1.04 equiv.), sodium triacetoxyborohydride (212 mg, 1.0 mmol, 2.0 equiv.), and dry DCE (5 mL) for 6 hours. The desired product was obtained as white solid (150 mg, 80% yield) after purification by flash

column chromatography on silica gel (DCM/MeOH = 20/1).

^{1}H NMR (400 MHz, $CDCl_3$) δ 8.06 - 7.98 (m, 2H), 7.41 - 7.35 (m, 2H), 7.32 - 7.27 (m, 2H), 7.17 - 7.09 (m, 2H), 2.98 (t, J = 7.0 Hz, 2H), 2.78 (d, J = 11.3 Hz, 2H), 2.47 (t, J = 7.2 Hz, 2H), 2.45 - 2.37 (m, 2H), 2.04 - 1.94 (m, 4H), 1.69 - 1.65 (m, 2H); ^{13}C NMR (101 MHz, $CDCl_3$) δ 198.4, 166.9, 164.3, 146.9, 133.7, 133.7, 132.7, 130.7, 130.6, 128.4, 126.1, 115.7, 115.5, 71.1, 57.8, 49.3, 38.4, 36.2, 21.9; HRMS (ESI) calcd for $C_{21}H_{24}FNO_2$ $[M + H]^+$ m/z = 376.1474, found: 376.1476.

3.3 Supplementary Figures of NMR Spectra Data

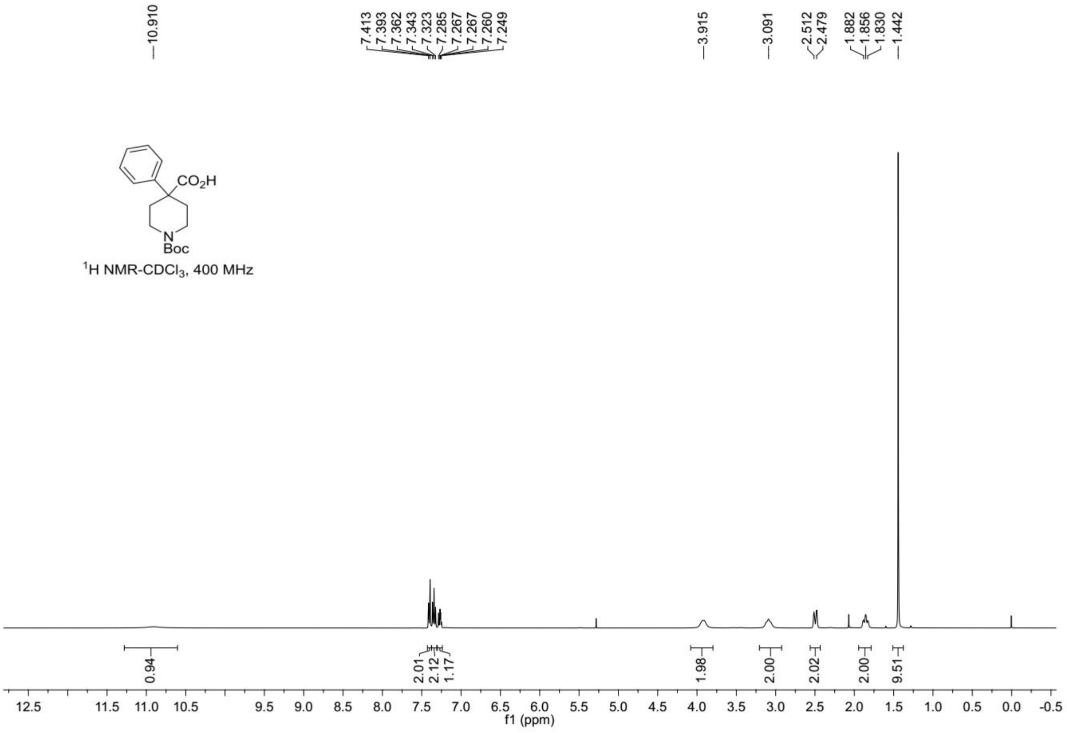

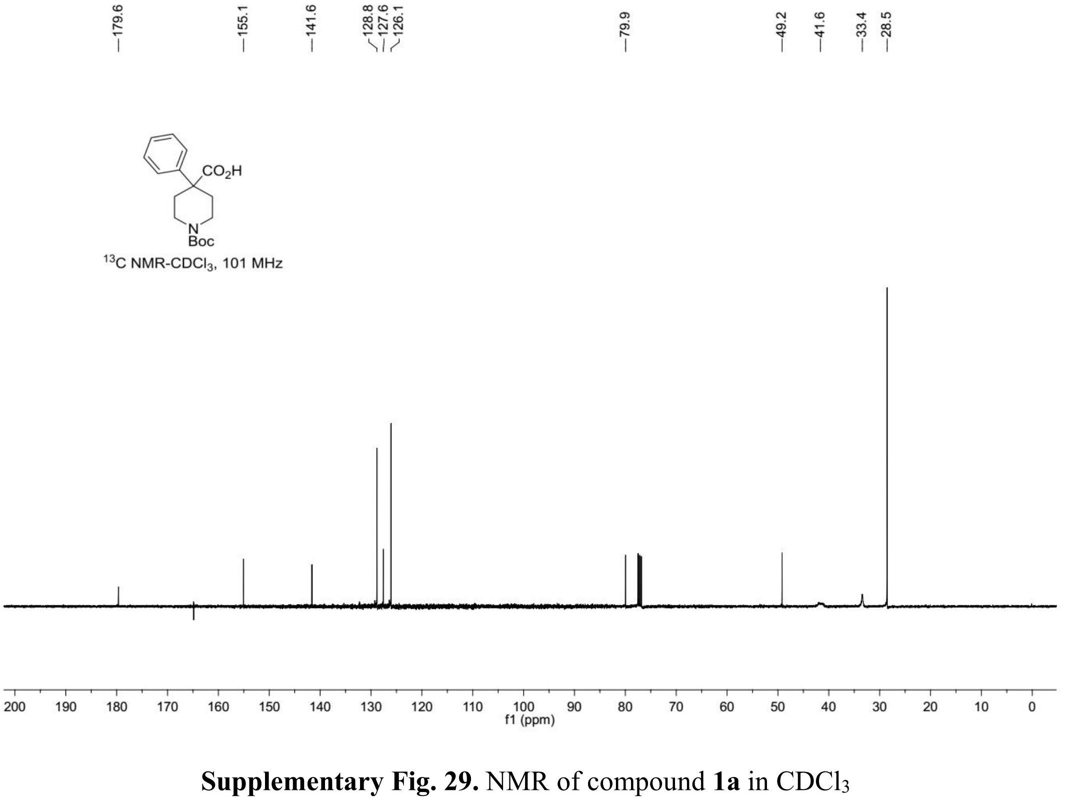

Supplementary Fig. 29. NMR of compound **1a** in $CDCl_3$

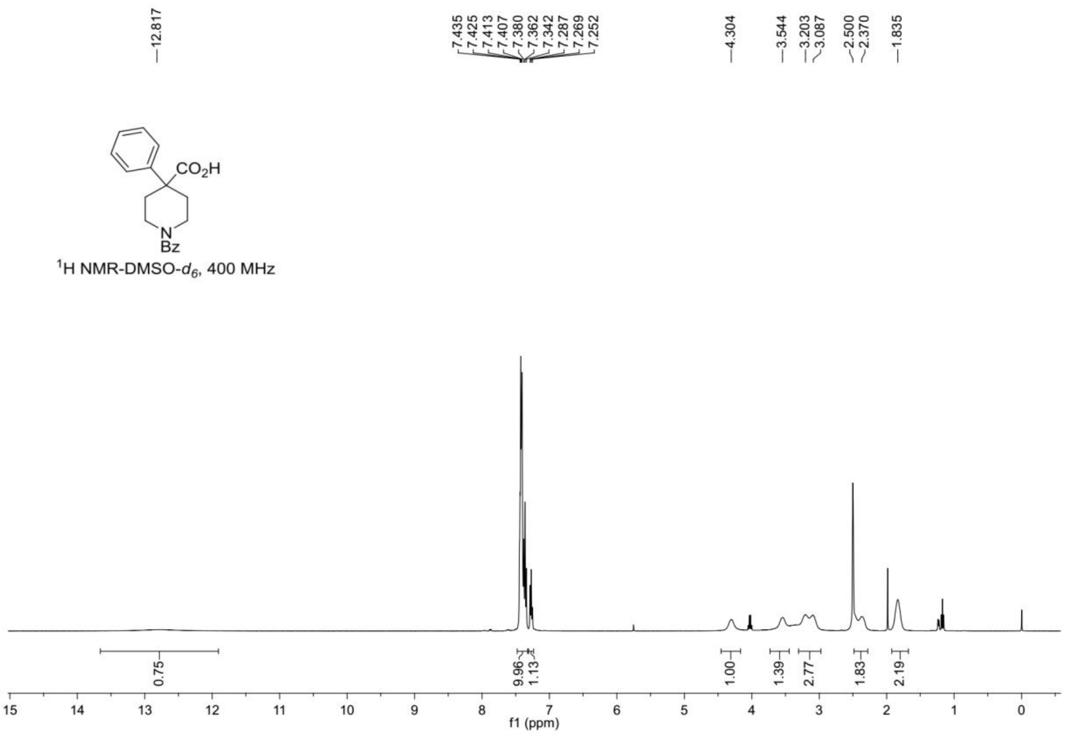

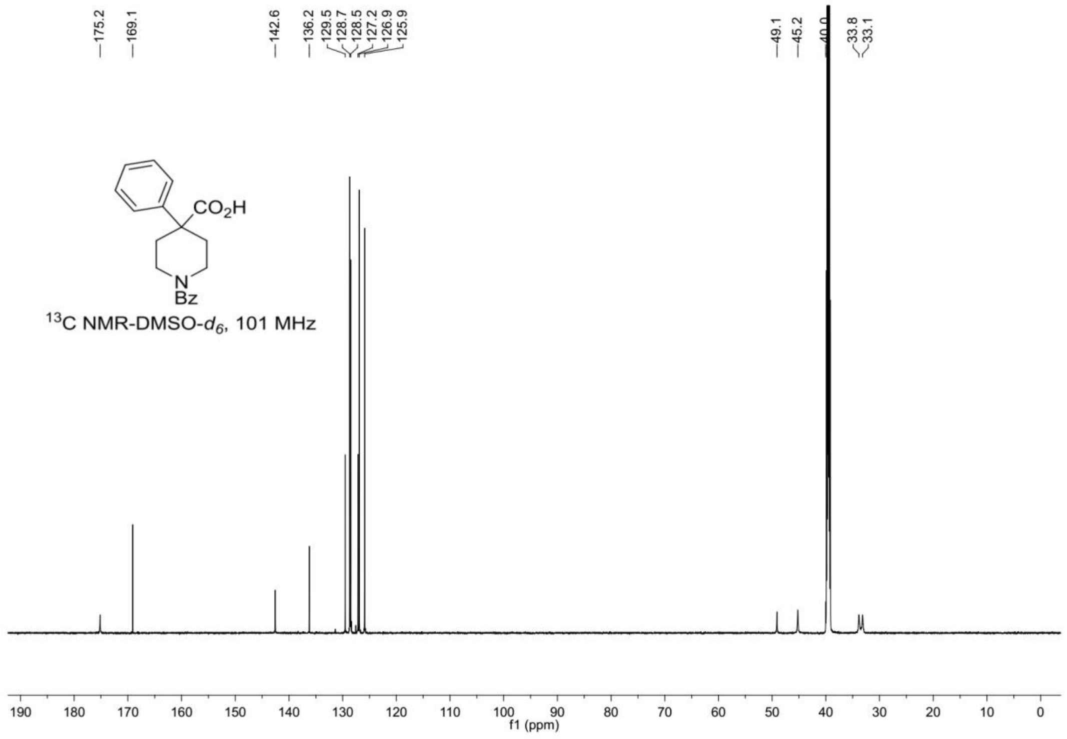

Supplementary Fig. 30. NMR of compound **2a** in DMSO-d_6

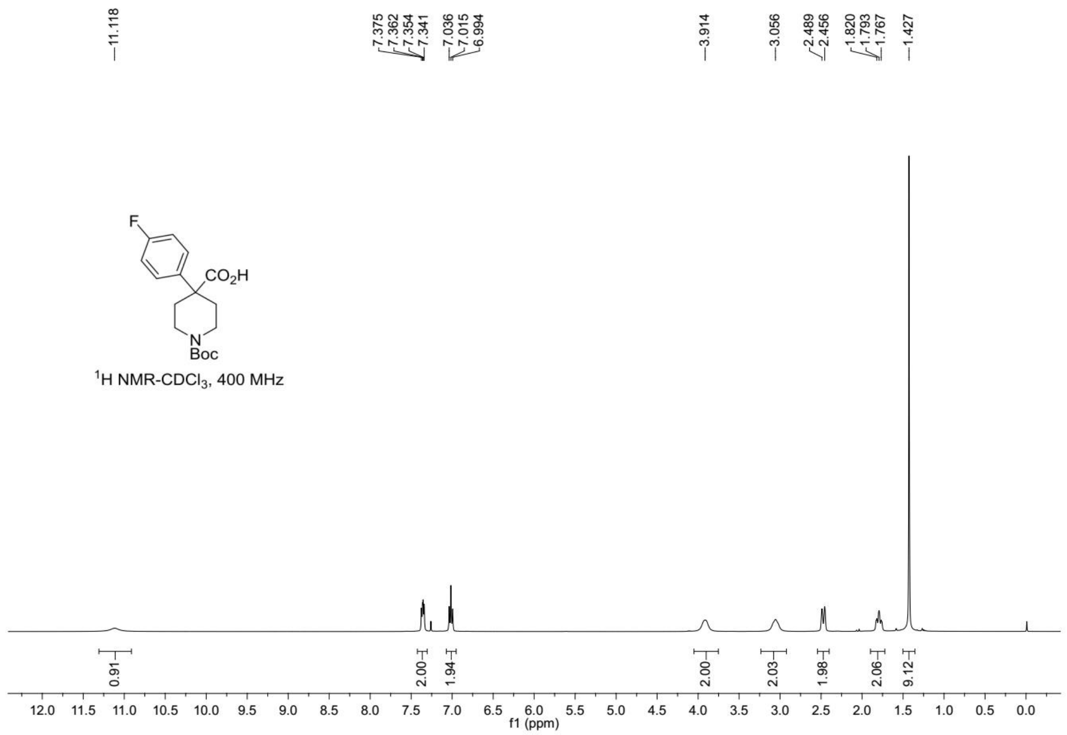

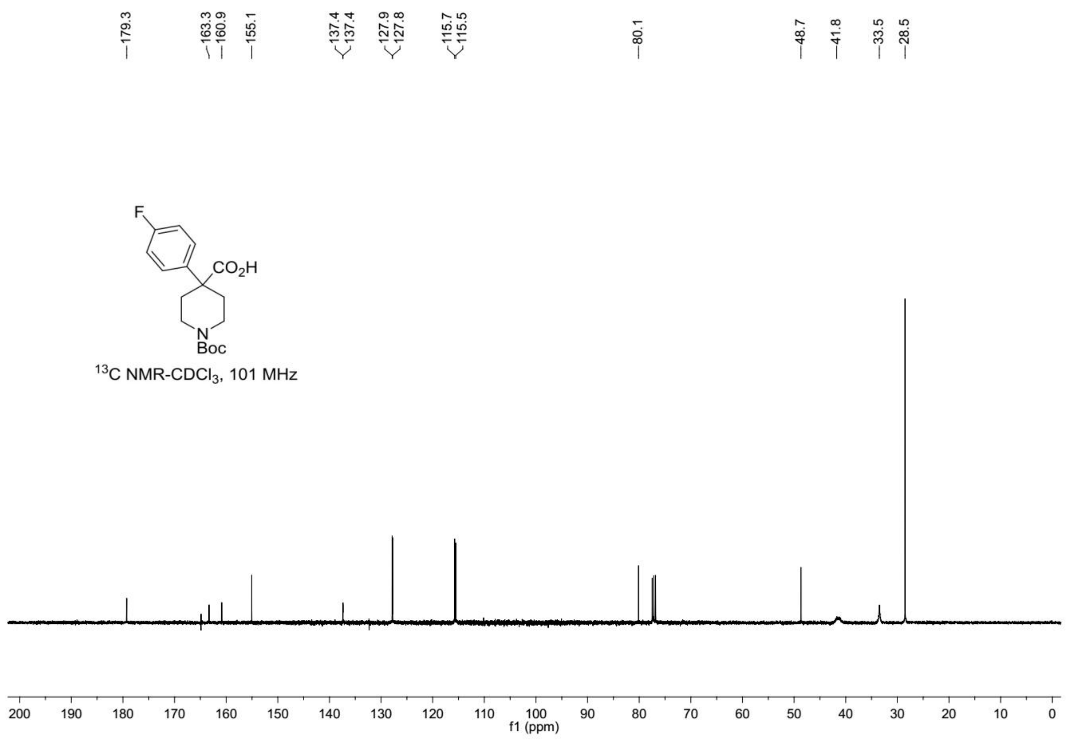

Supplementary Fig. 31. NMR of compound **3a** in $CDCl_3$

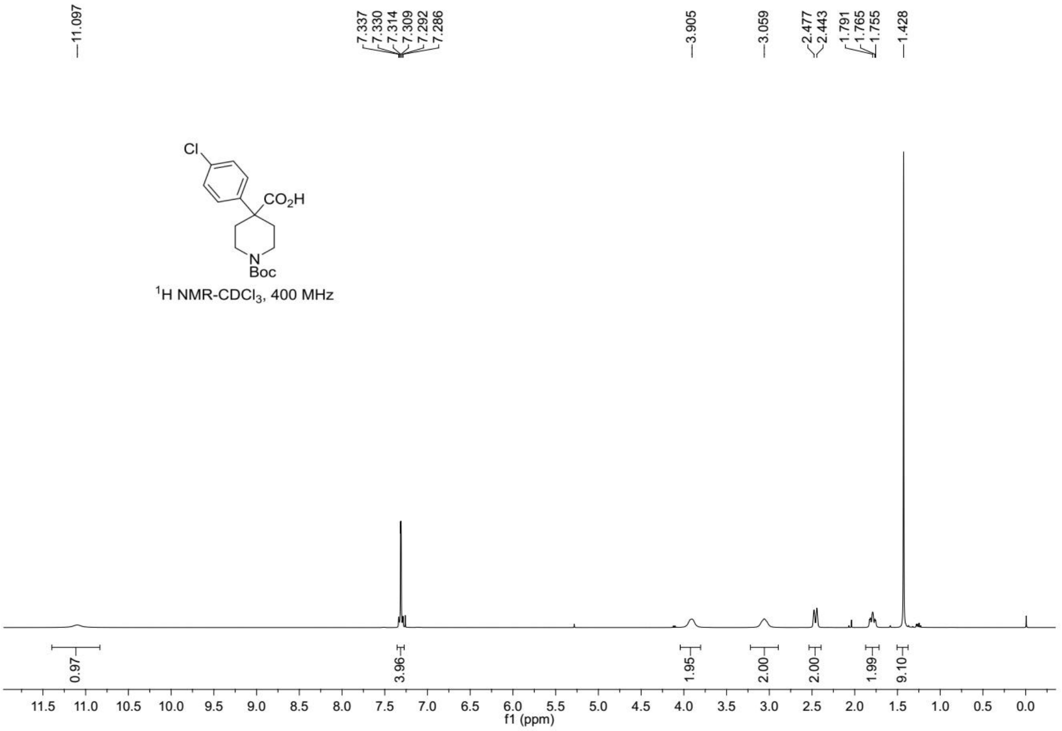

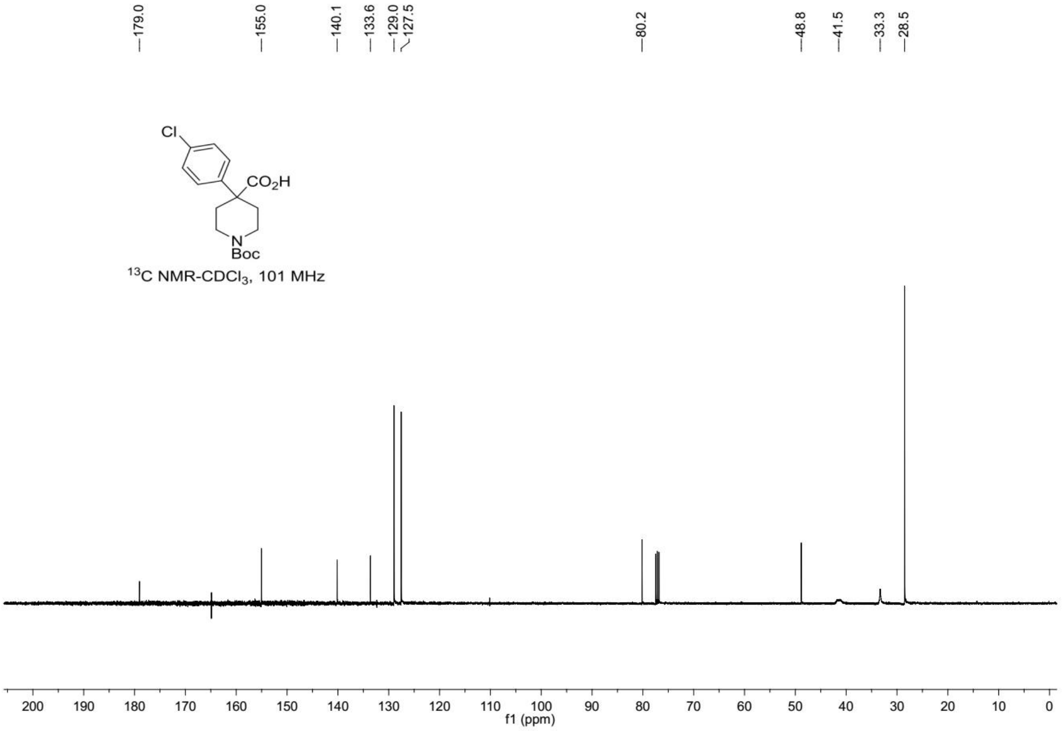

Supplementary Fig. 32. NMR of compound **4a** in $CDCl_3$

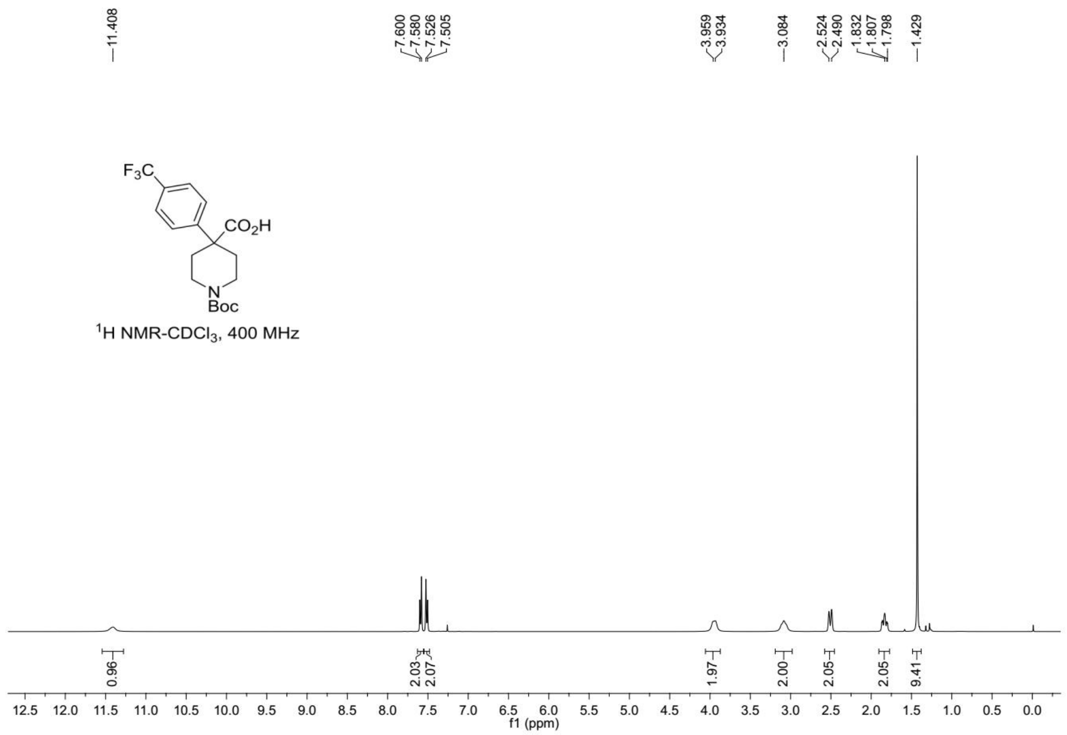

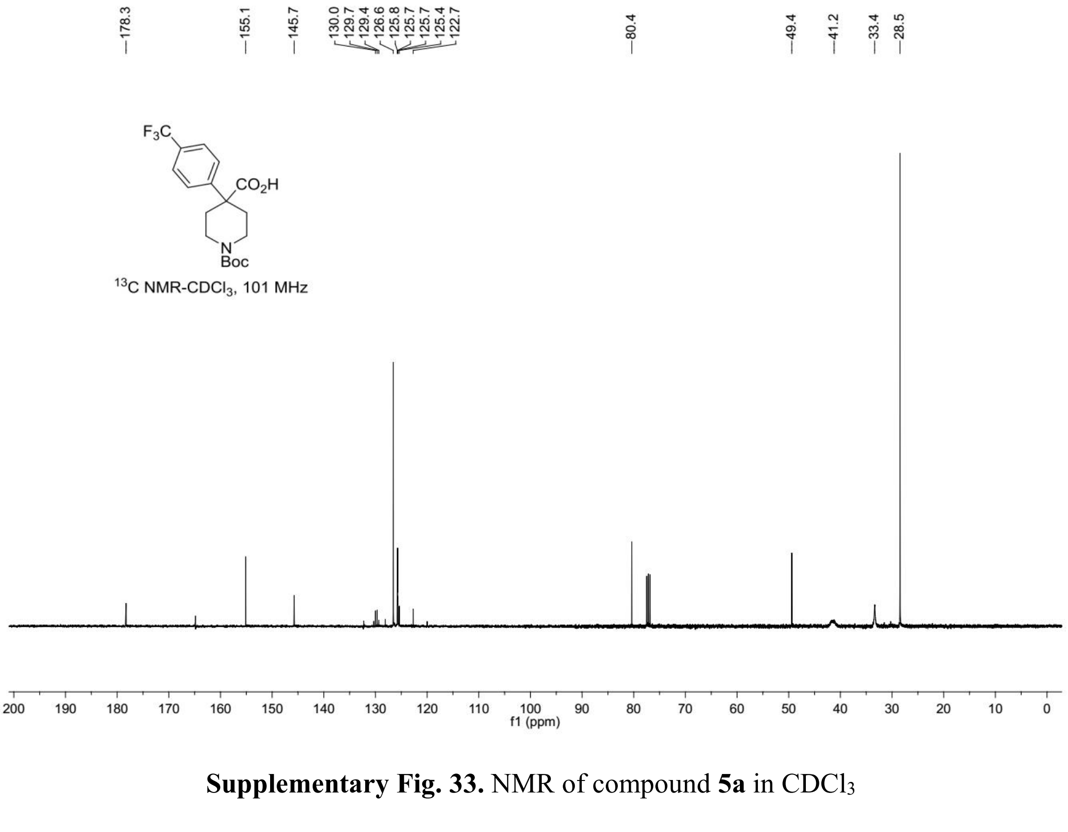

Supplementary Fig. 33. NMR of compound **5a** in $CDCl_3$

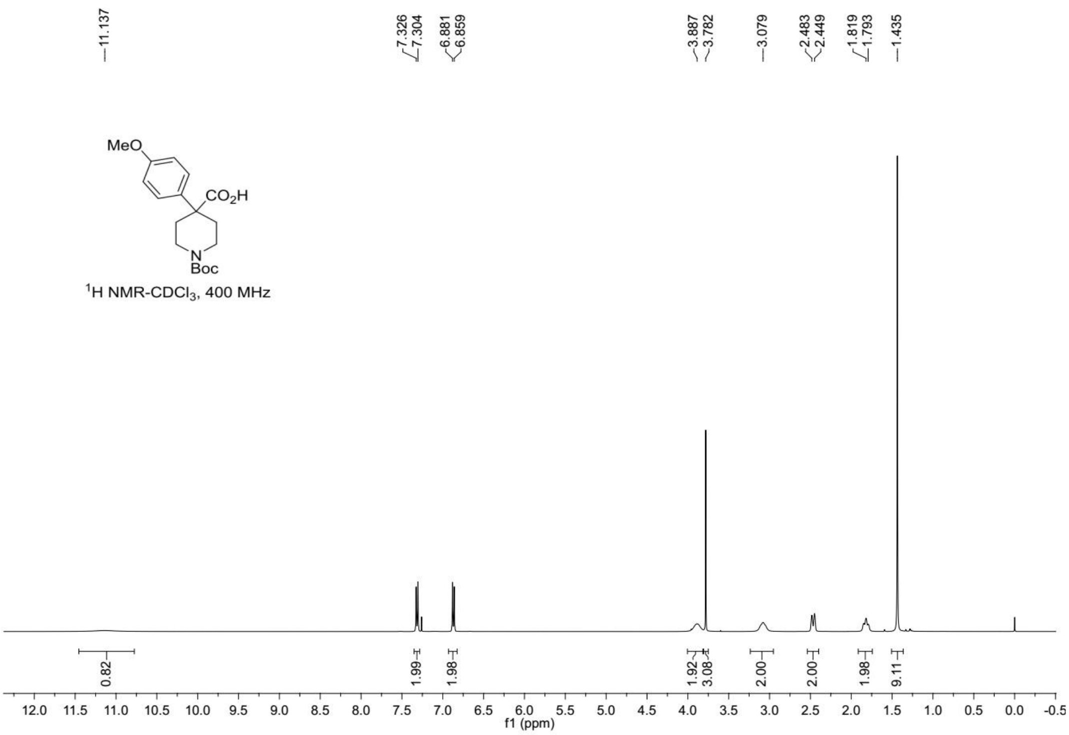

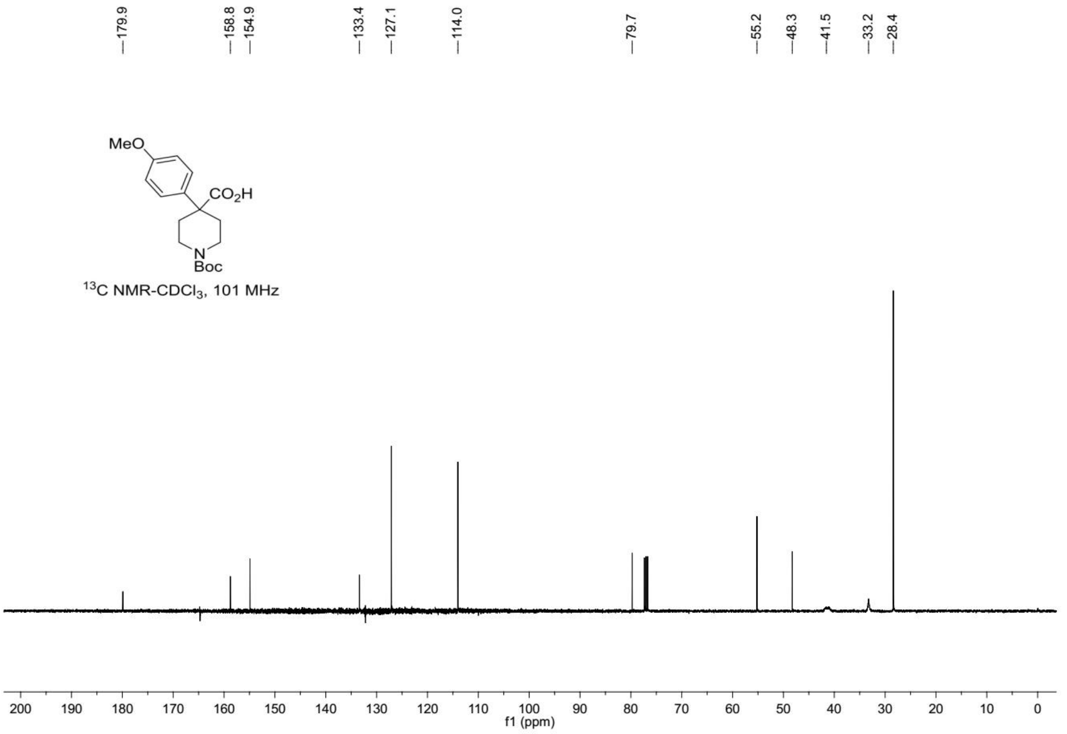

Supplementary Fig. 34. NMR of compound **6a** in $CDCl_3$

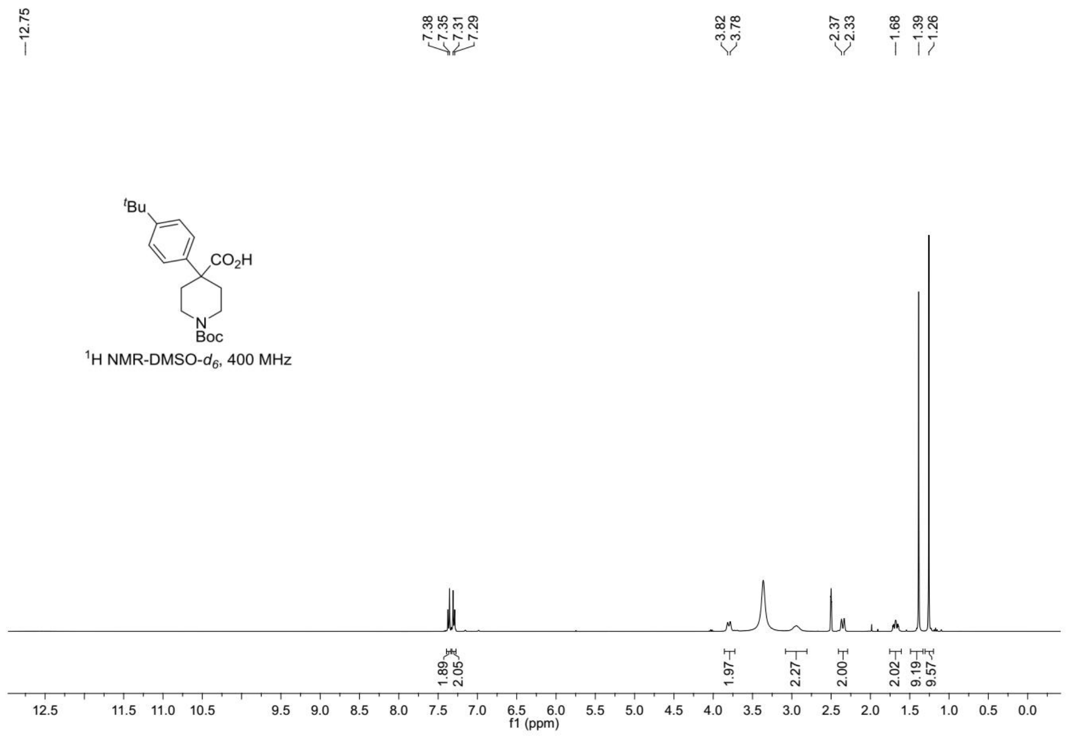

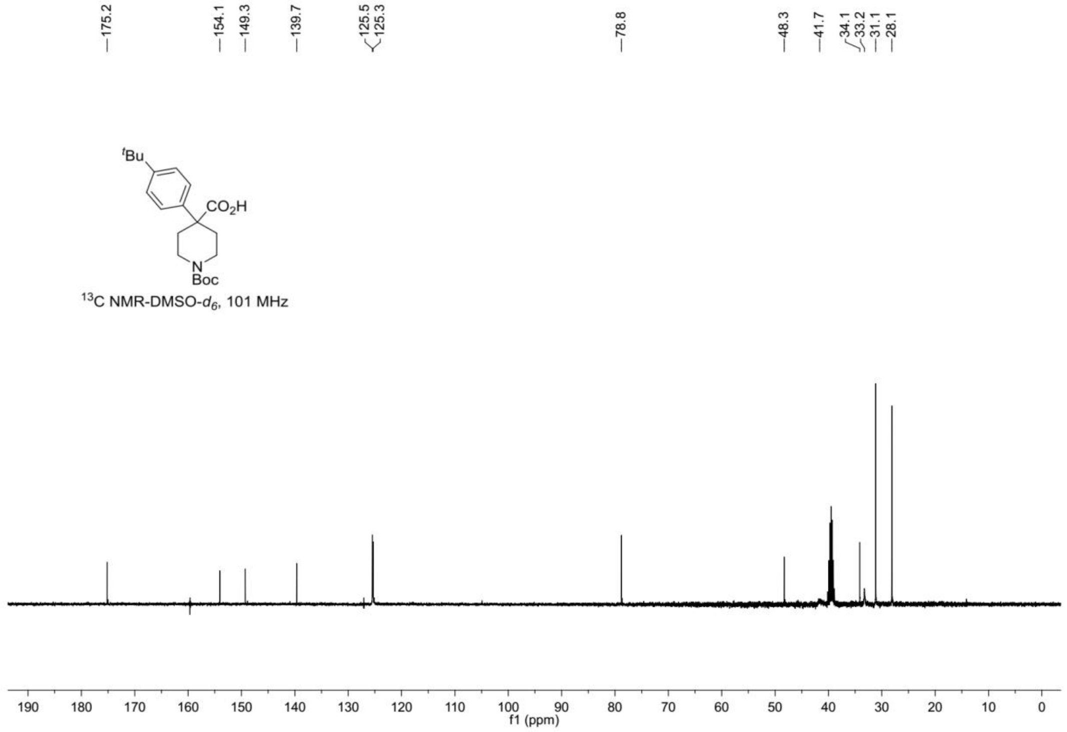

Supplementary Fig. 35. NMR of compound **7a** in DMSO-d_6

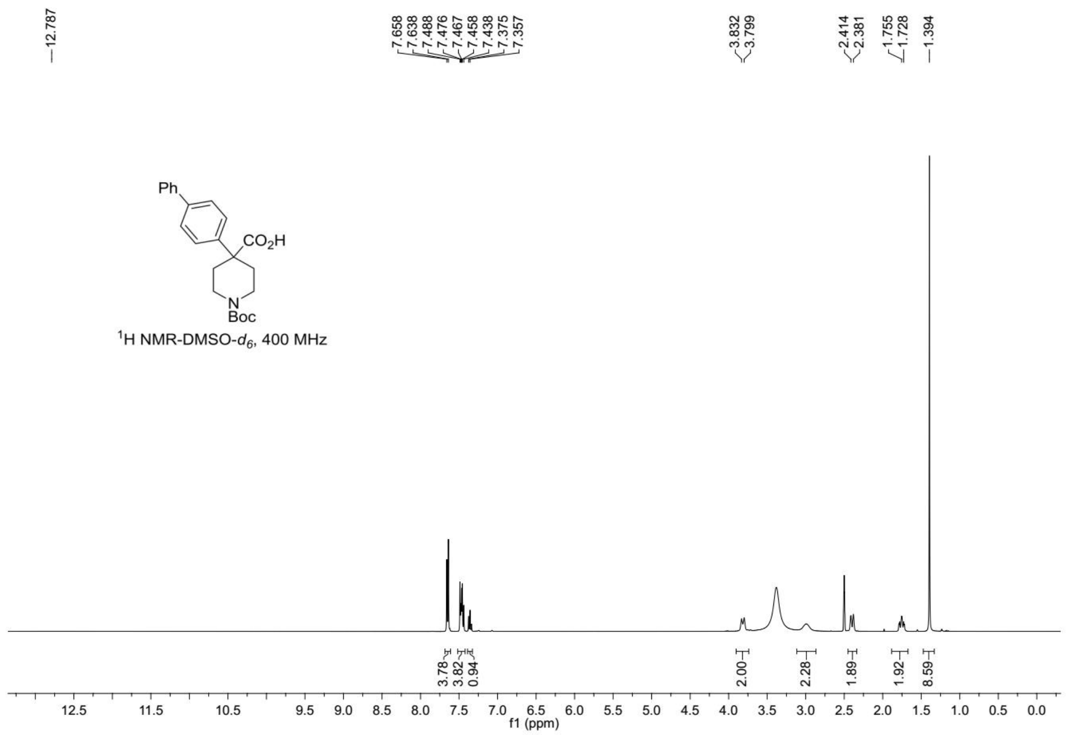

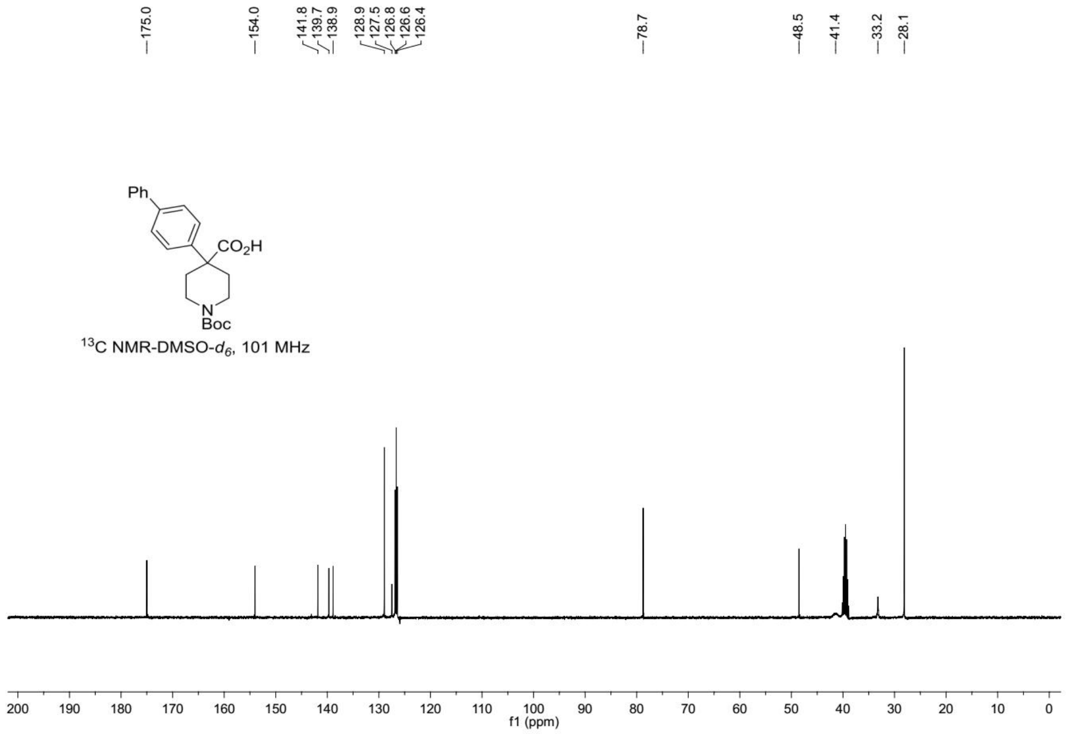

Supplementary Fig. 36. NMR of compound **8a** in DMSO-d_6

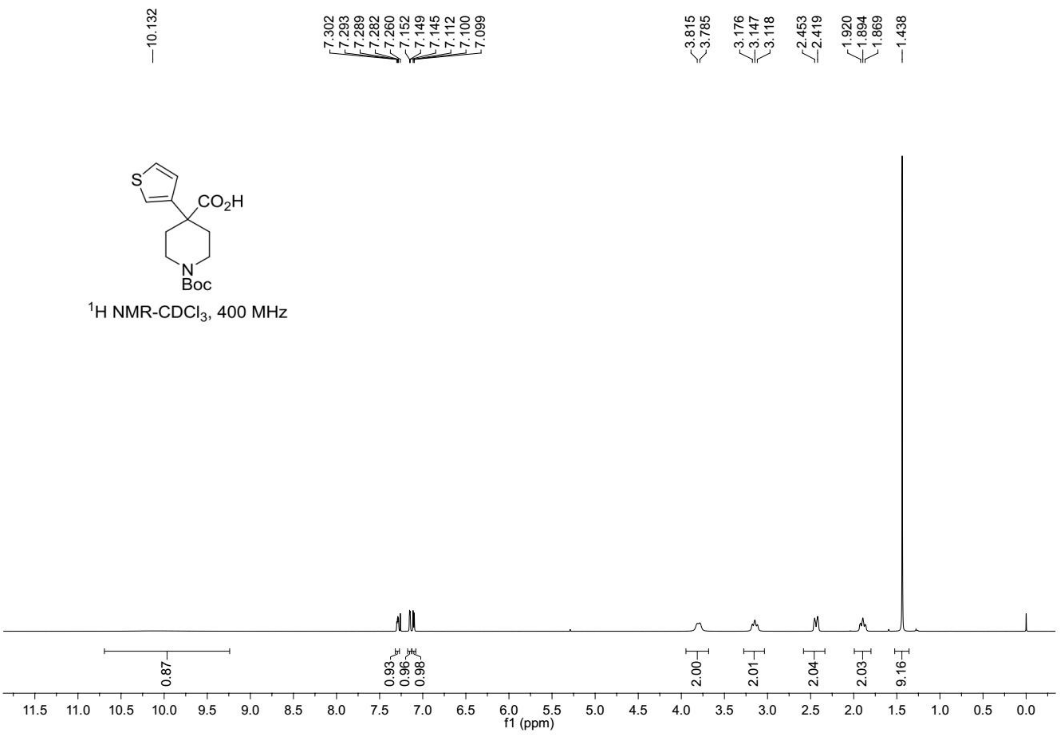

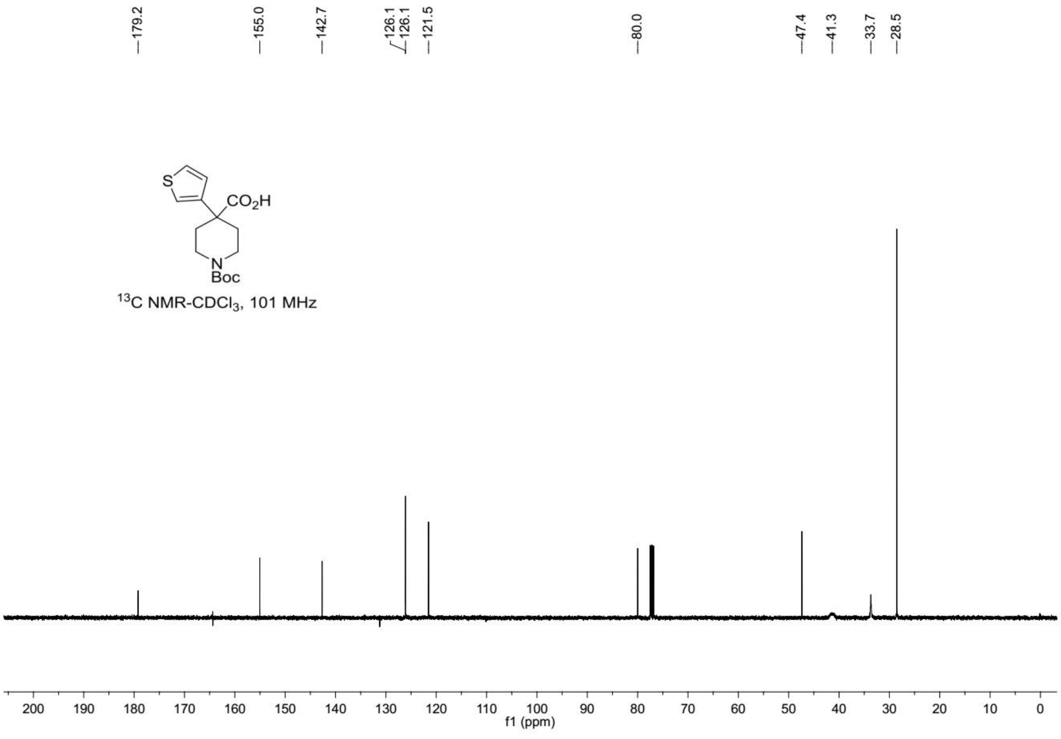

Supplementary Fig. 37. NMR of compound **9a** in $CDCl_3$

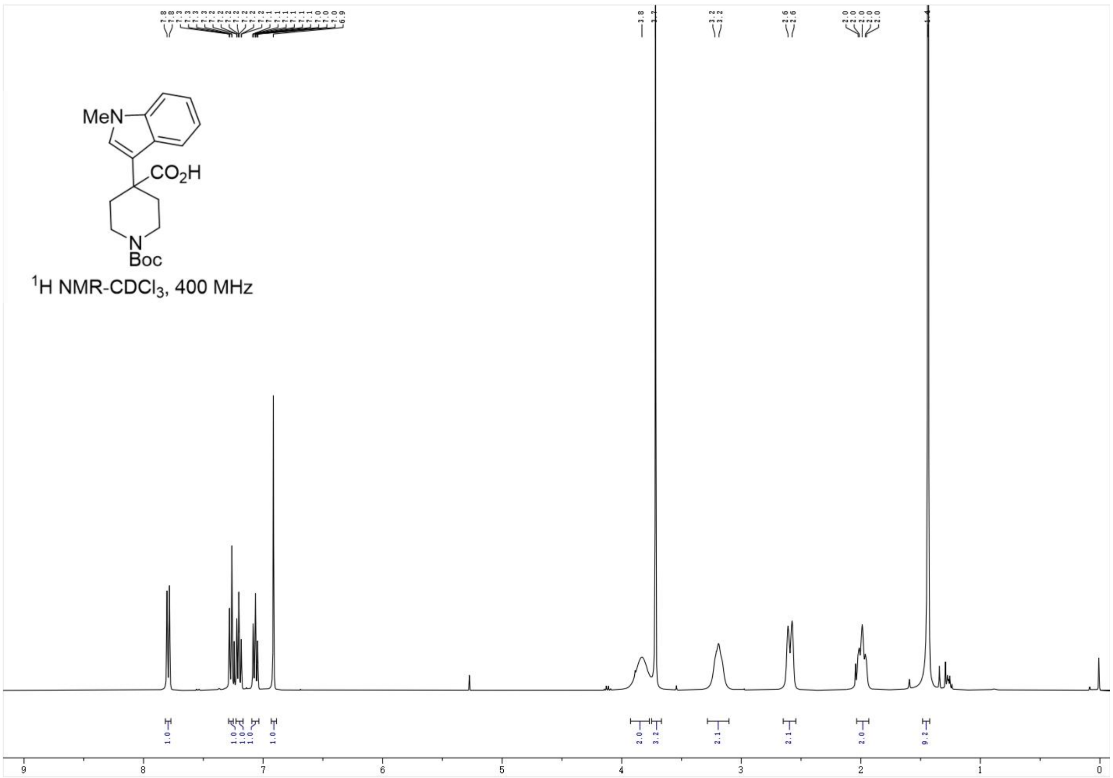

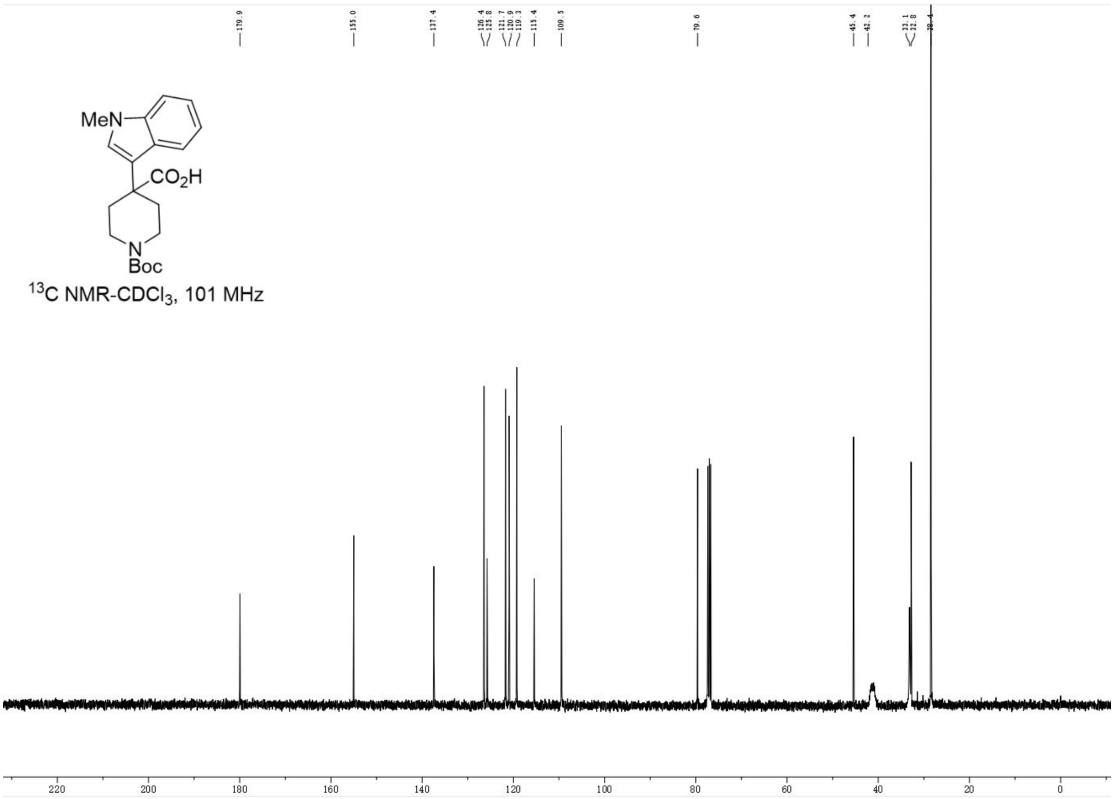

Supplementary Fig. 38. NMR of compound **10a** in $CDCl_3$

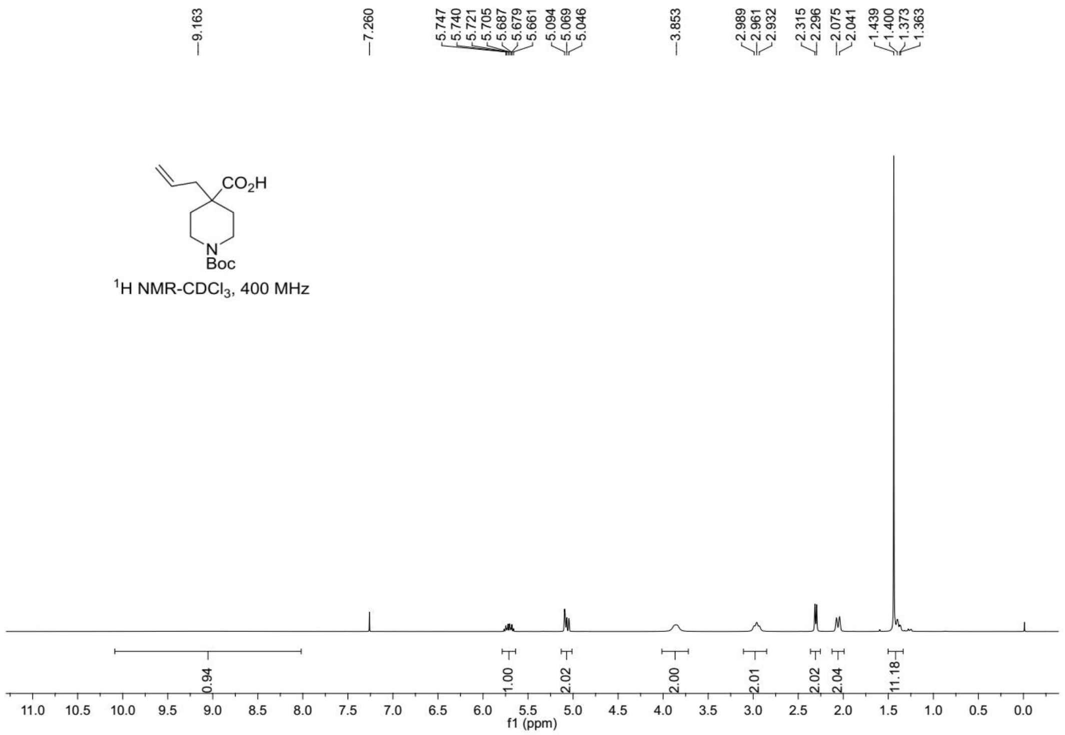

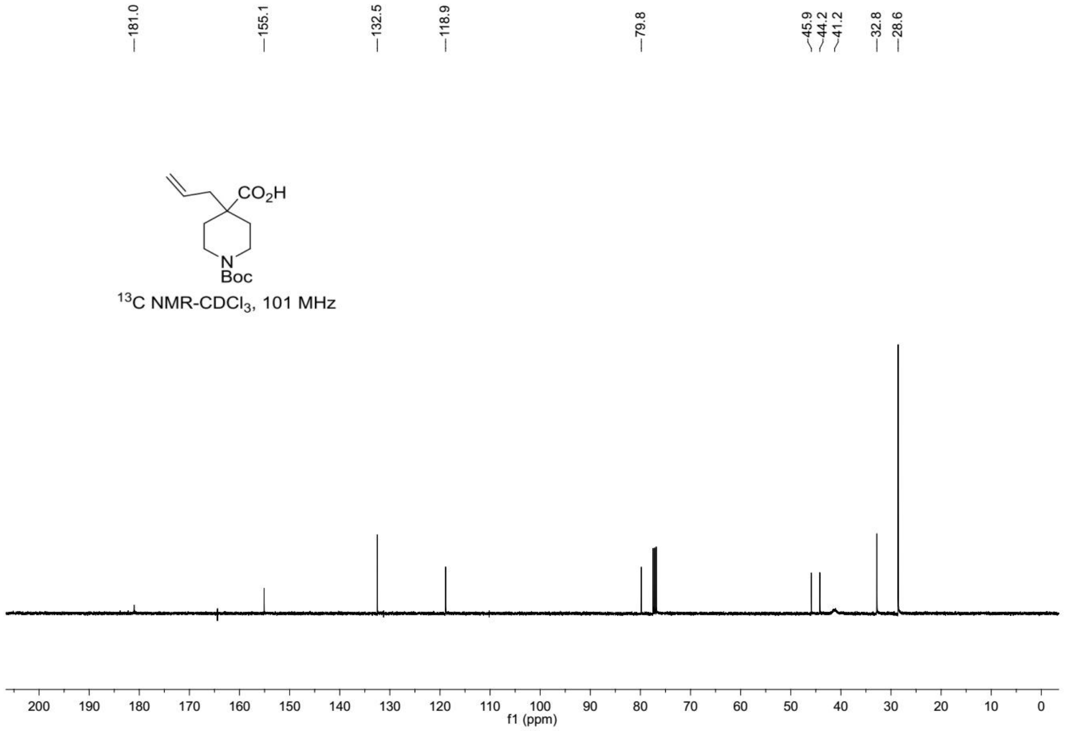

Supplementary Fig. 39. NMR of compound **17a** in $CDCl_3$

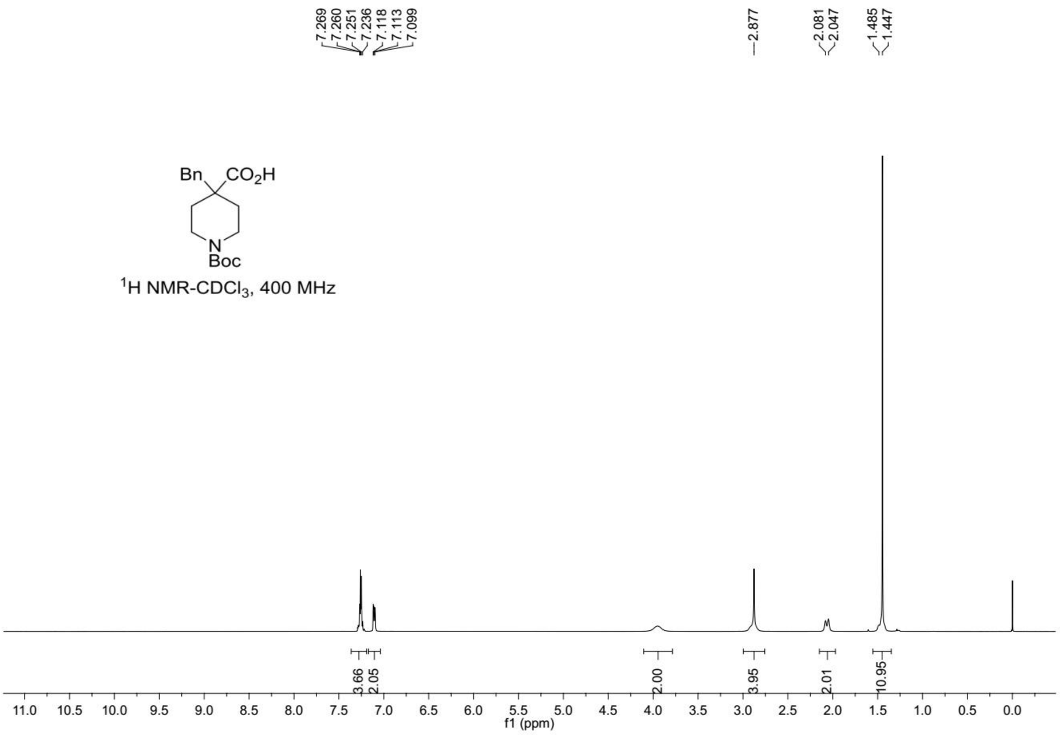

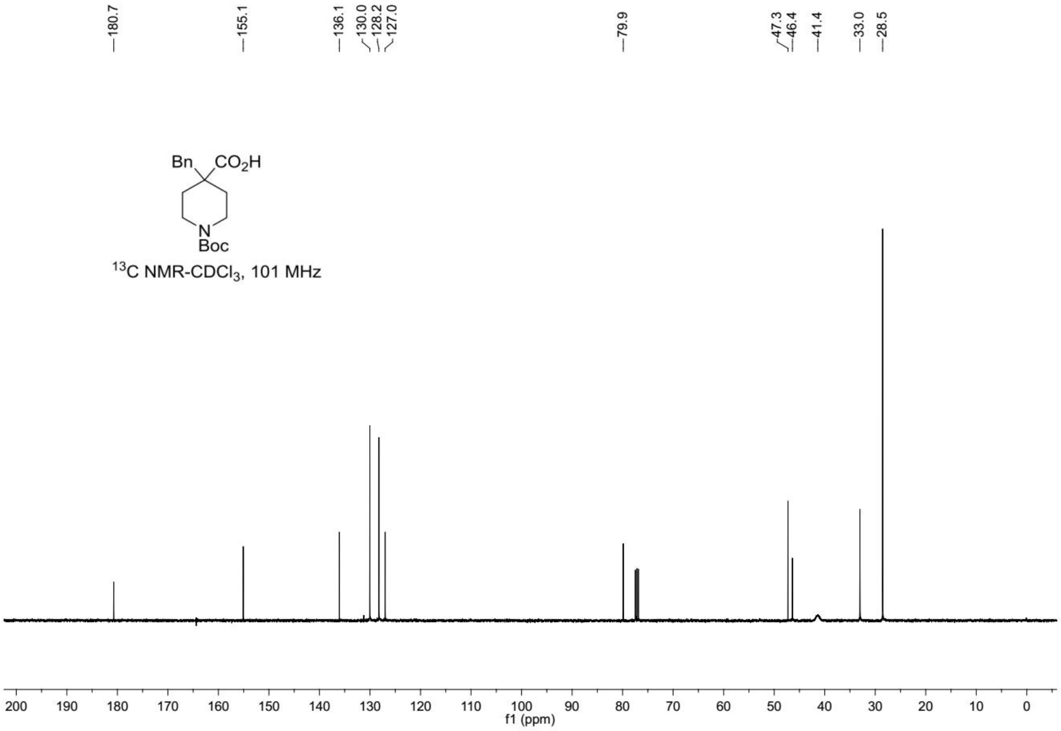

Supplementary Fig. 40. NMR of compound **18a** in $CDCl_3$

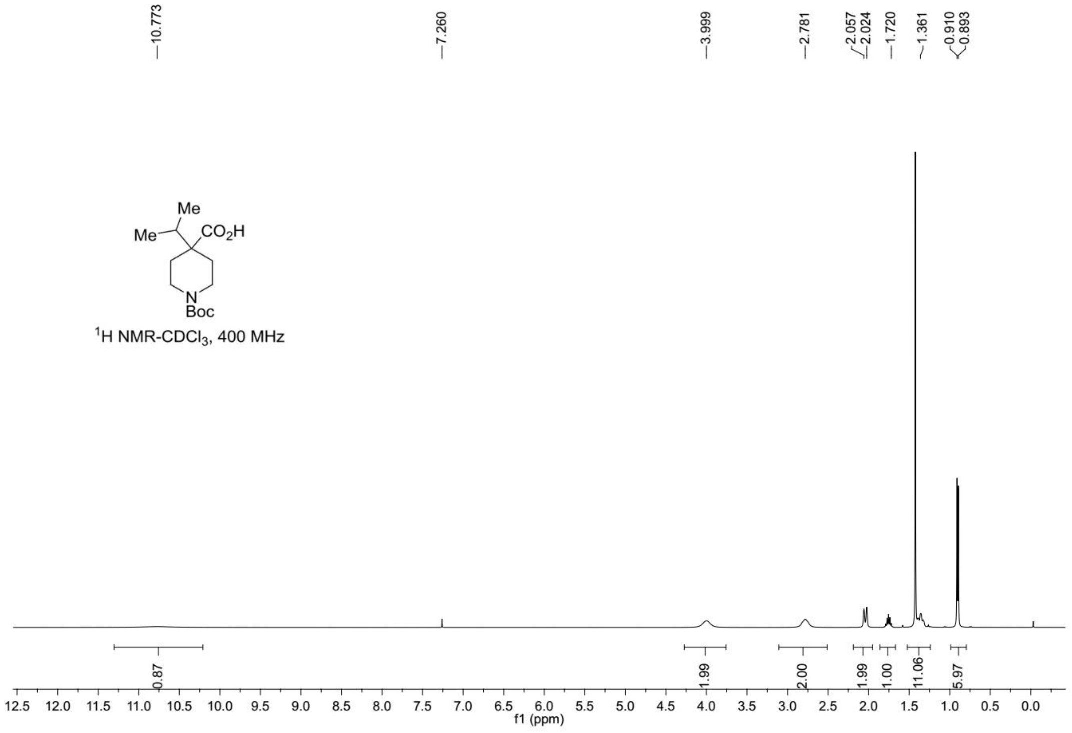

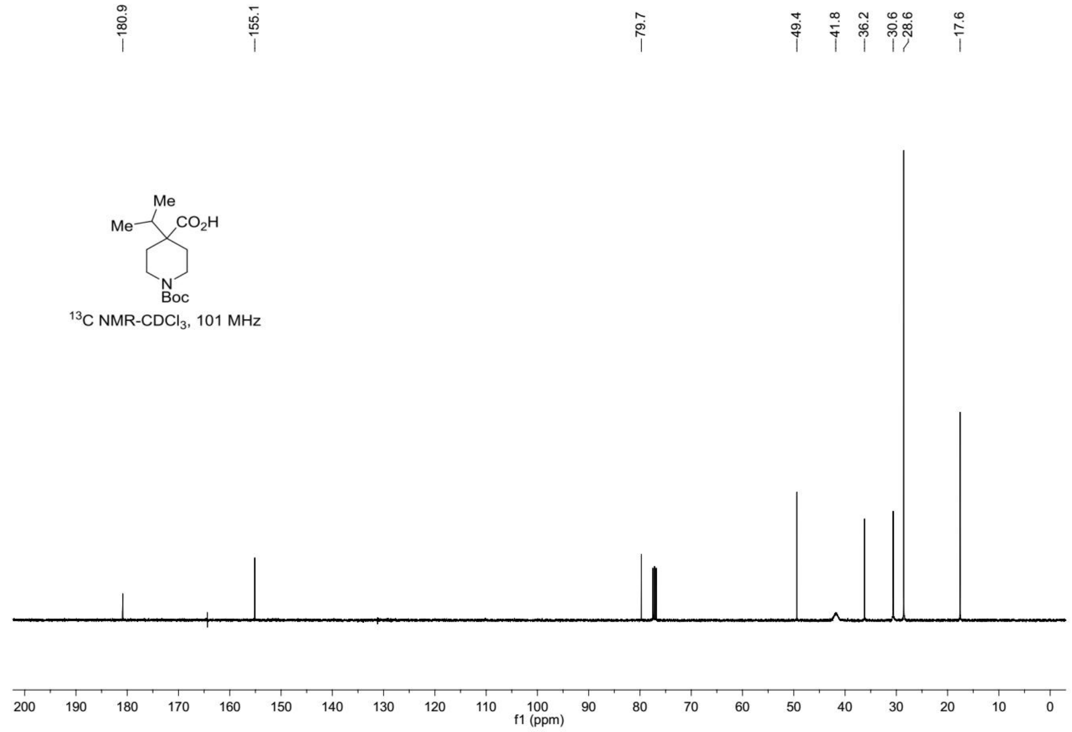

Supplementary Fig. 41. NMR of compound **19a** in $CDCl_3$

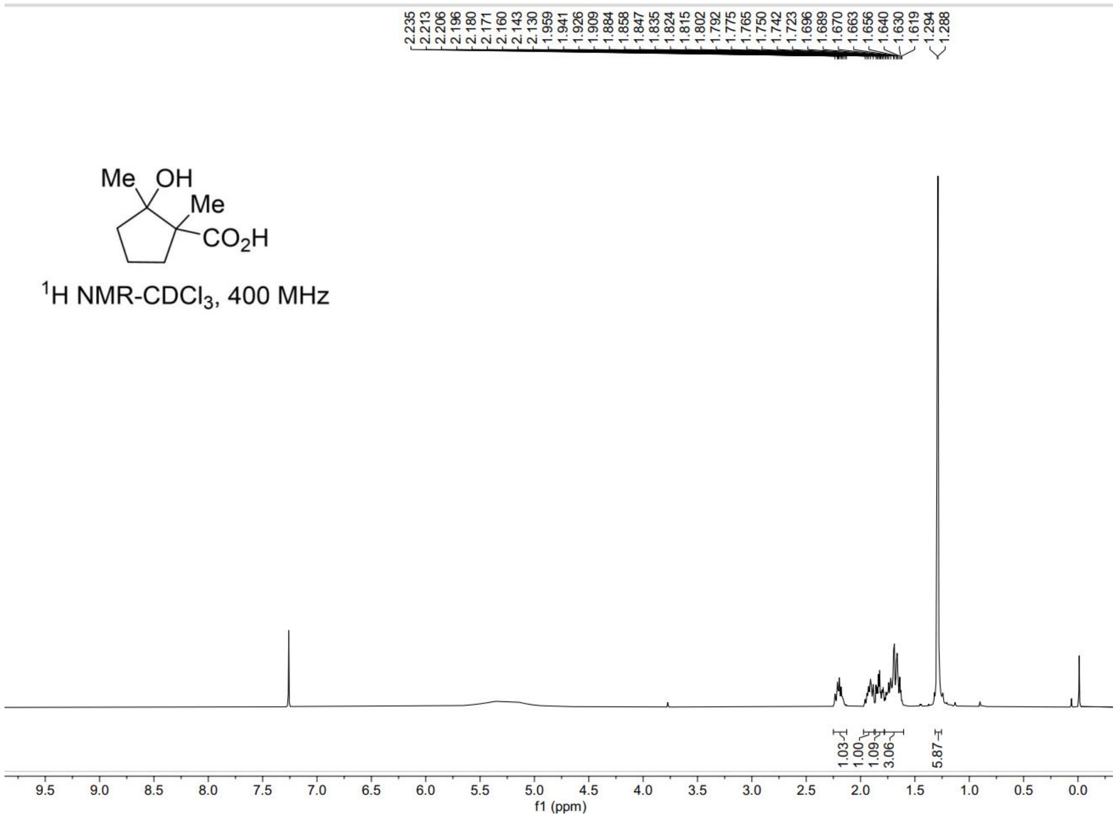

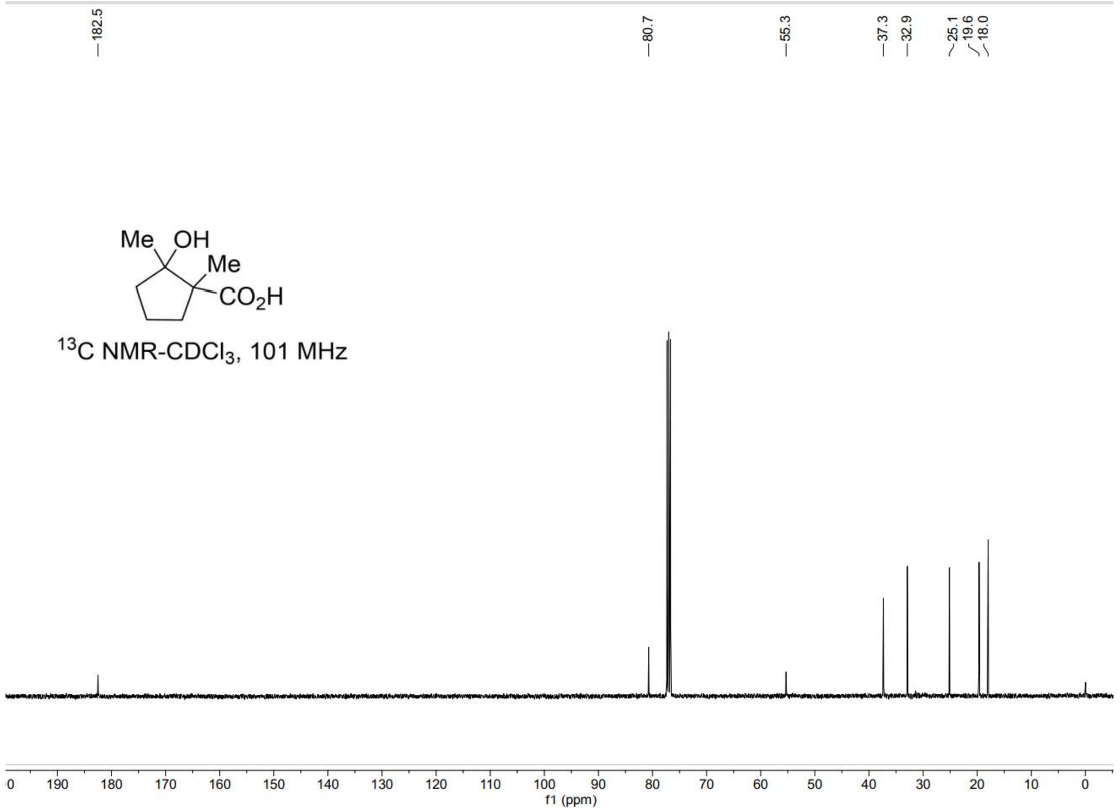

Supplementary Fig. 42. NMR of compound **38a** in $CDCl_3$

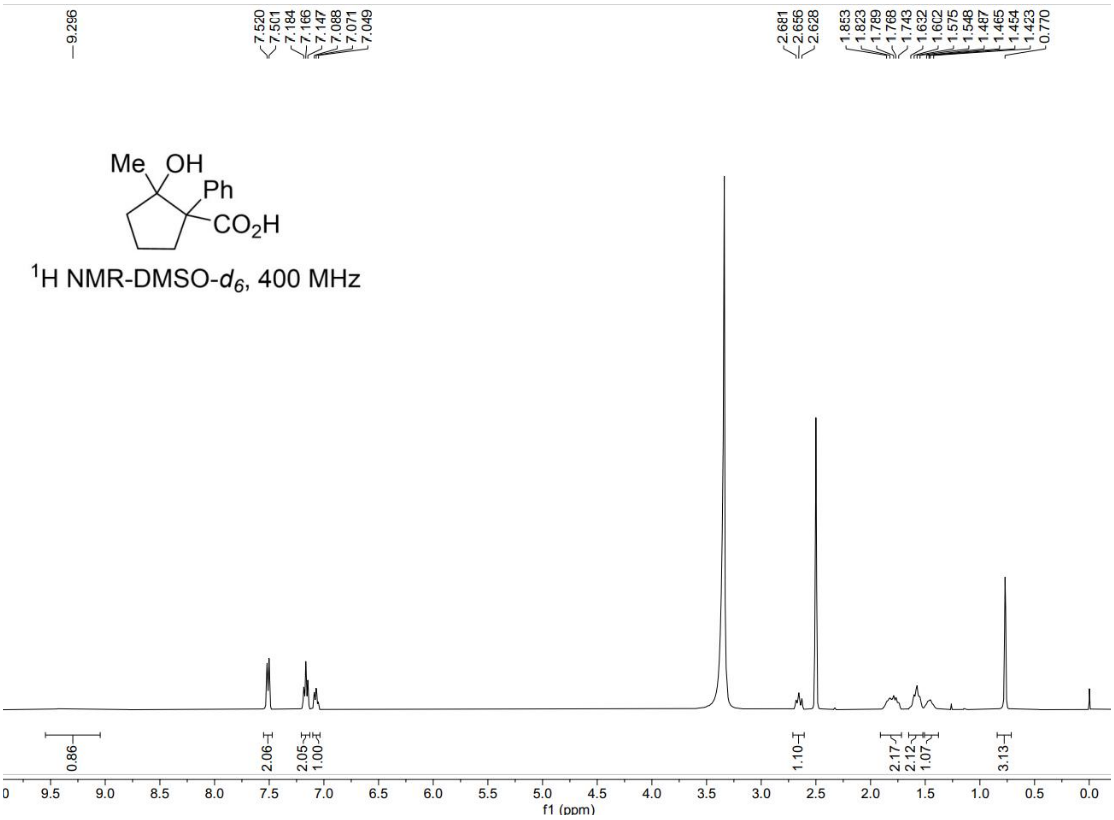

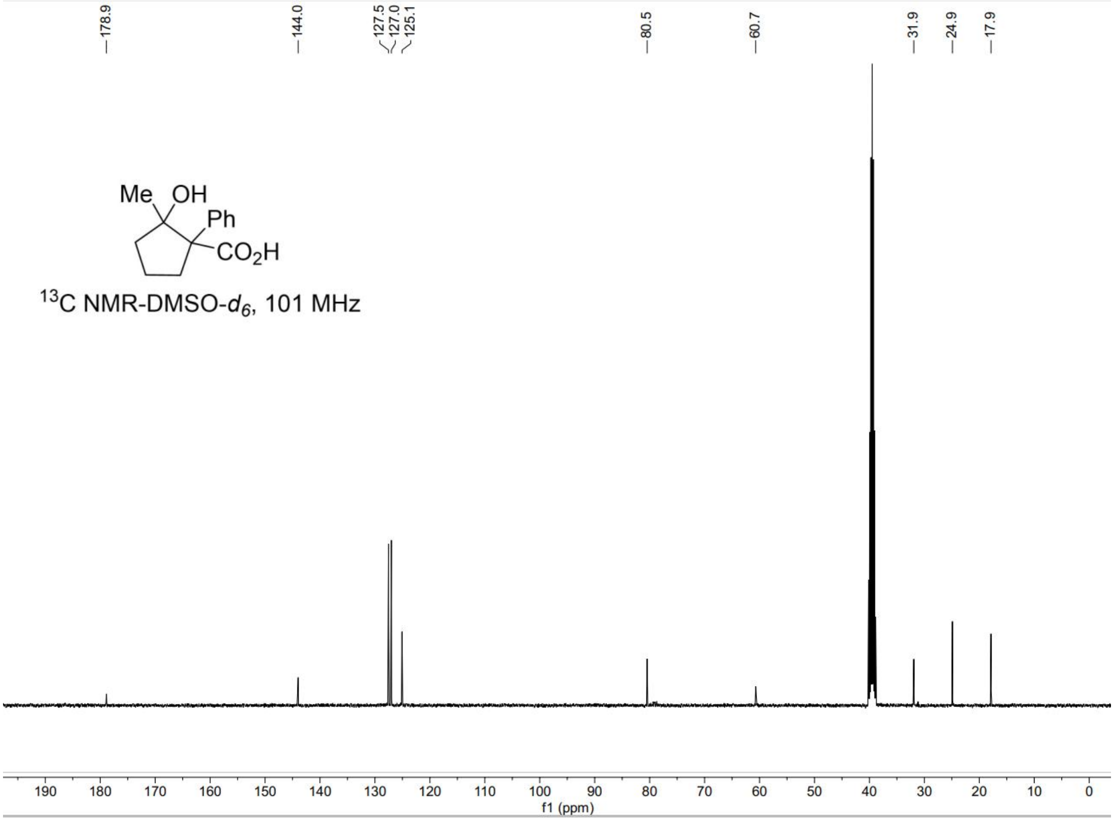

Supplementary Fig. 43. NMR of compound **39a** in $CDCl_3$ and DMSO-d_6

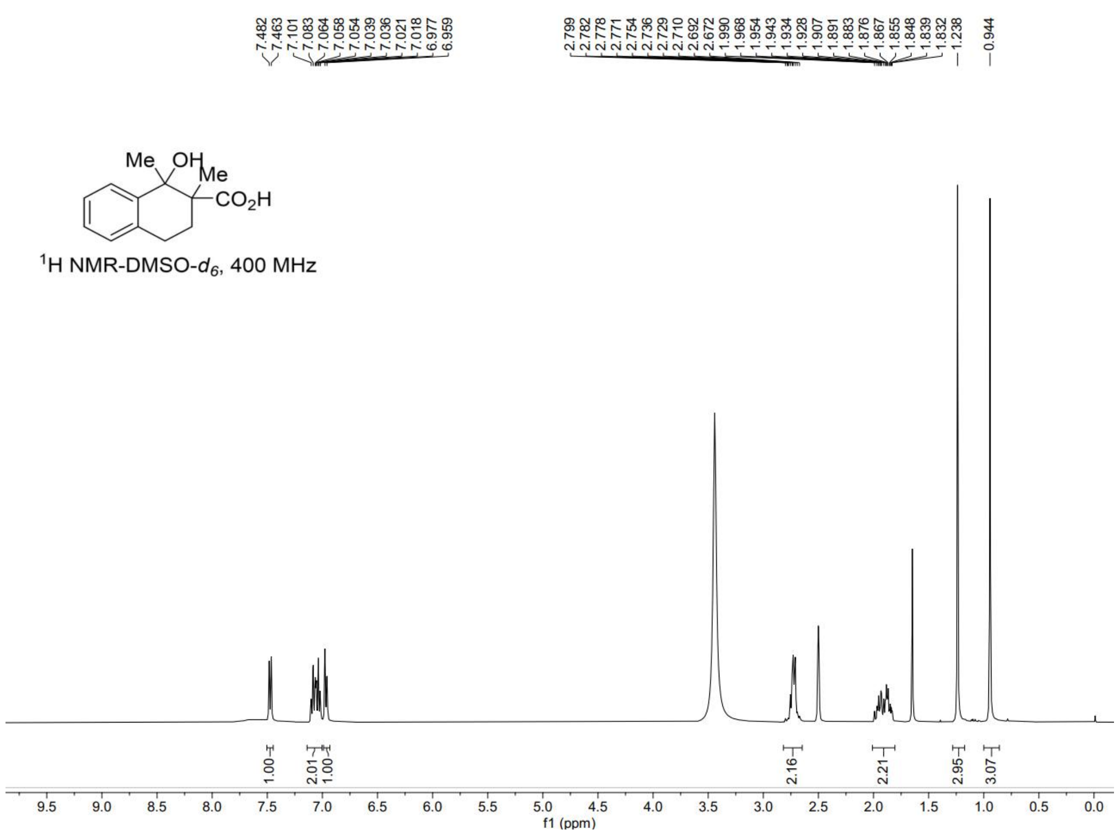

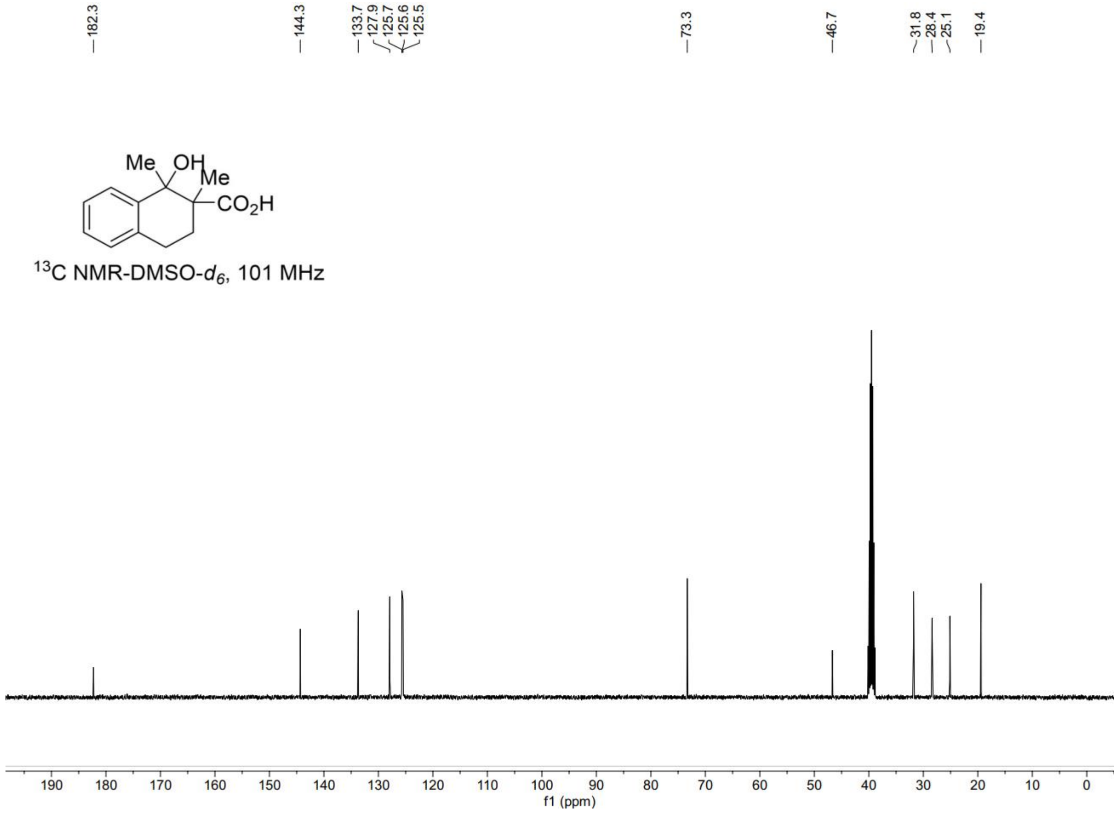

Supplementary Fig. 44. NMR of compound **40a** in DMSO-d_6

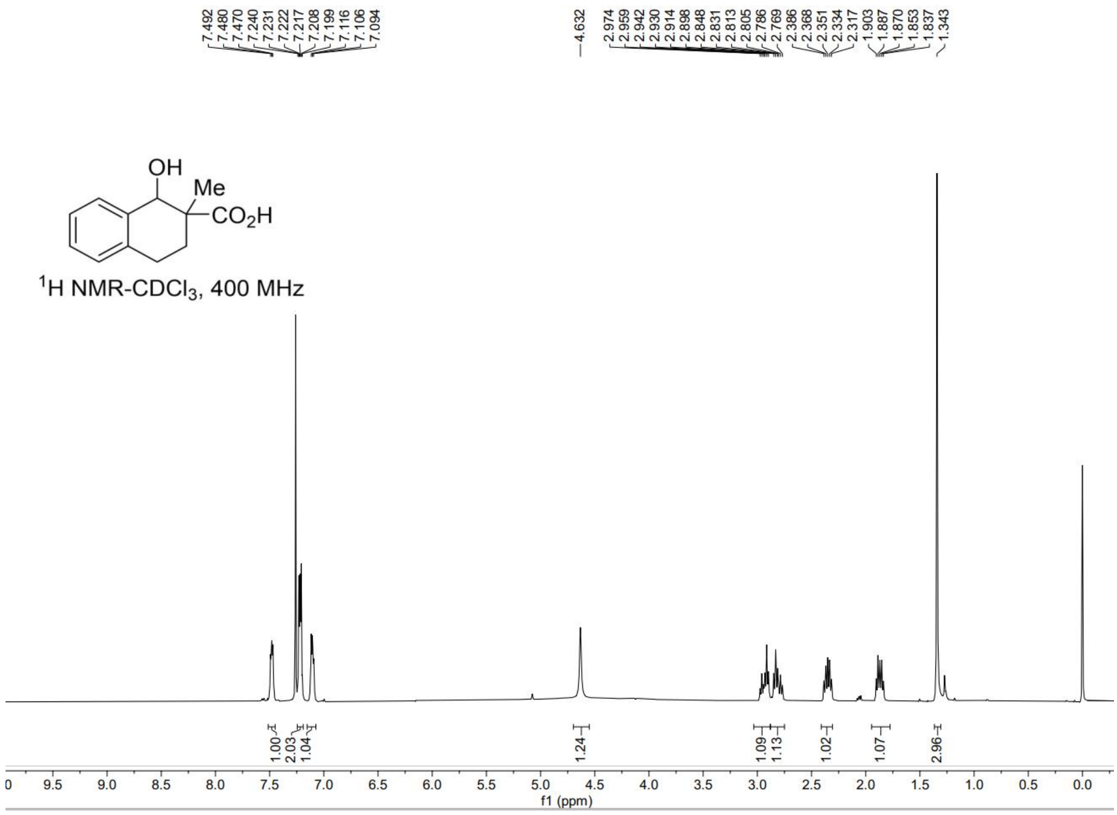

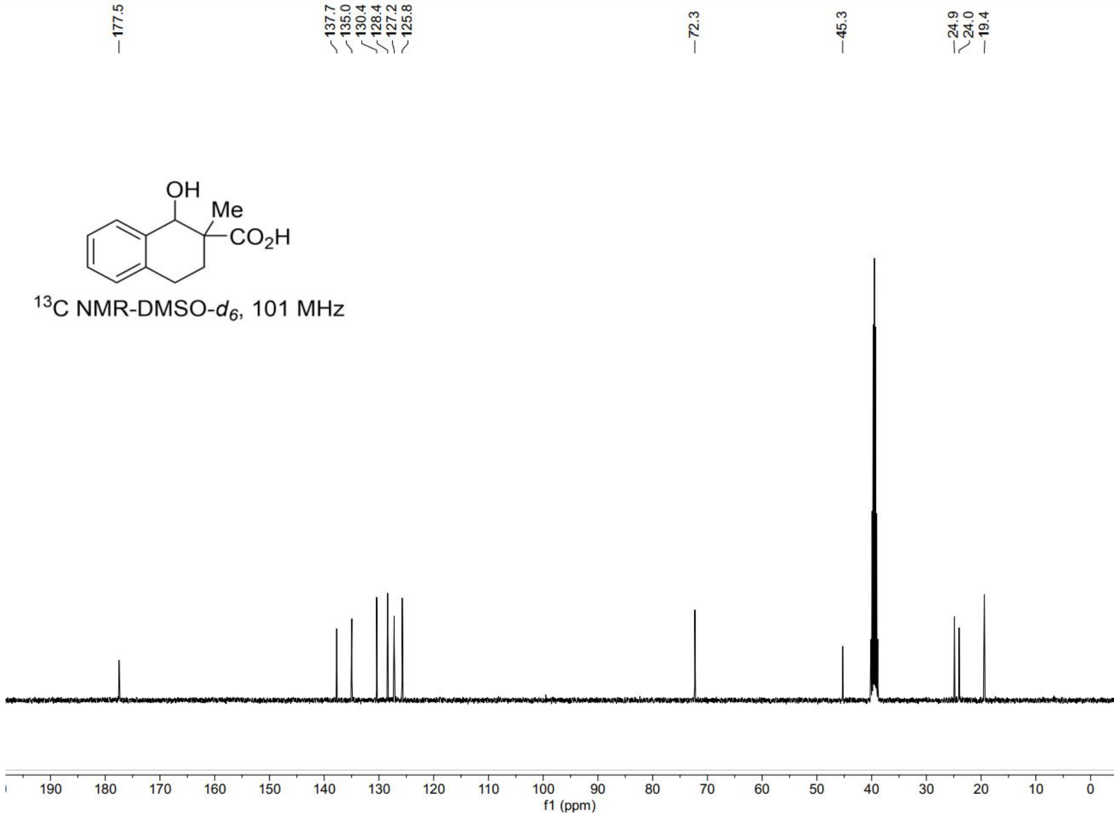

Supplementary Fig. 45. NMR of compound **41a** in $CDCl_3$ and DMSO-d_6

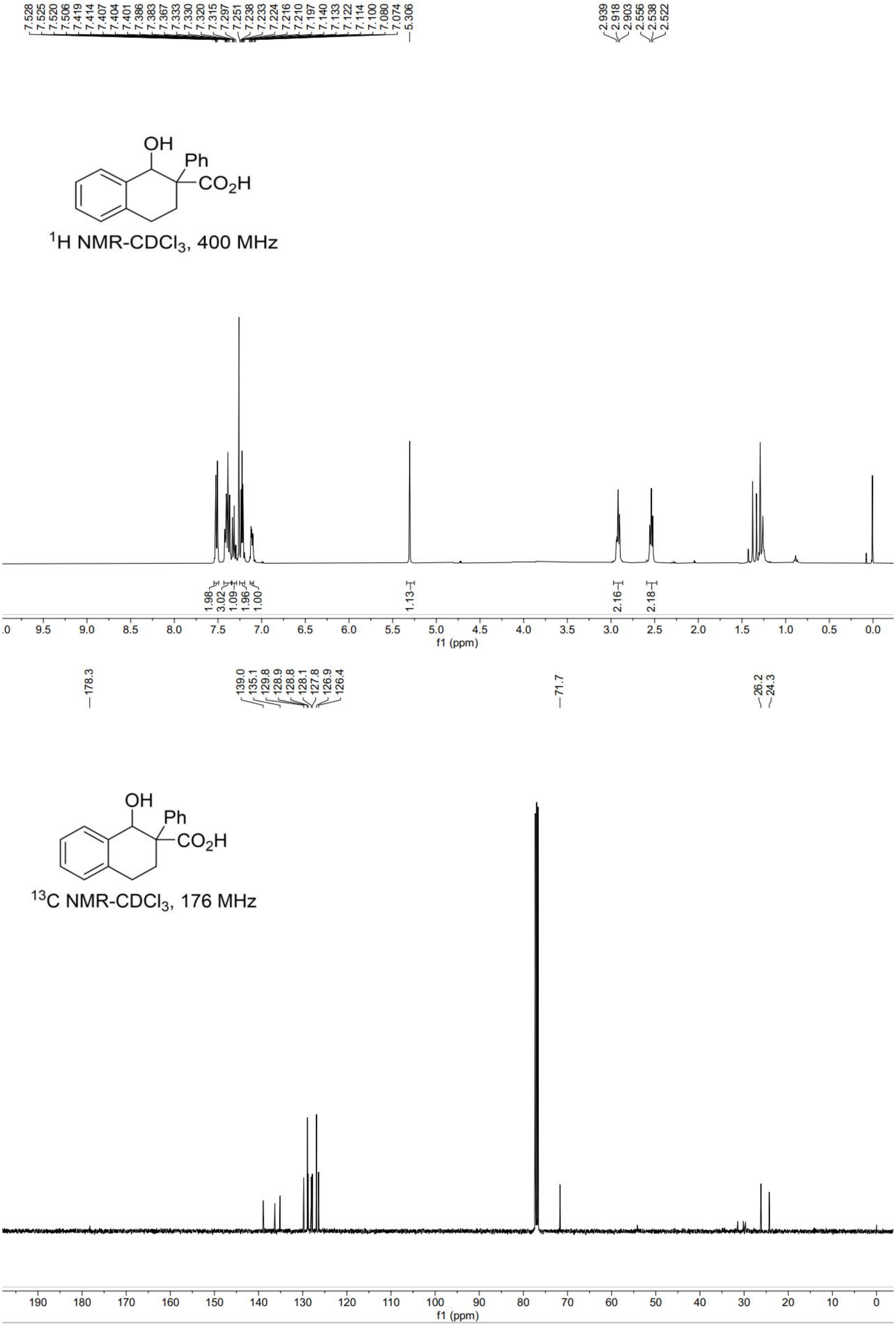

Supplementary Fig. 46. NMR of compound **42a** in $CDCl_3$

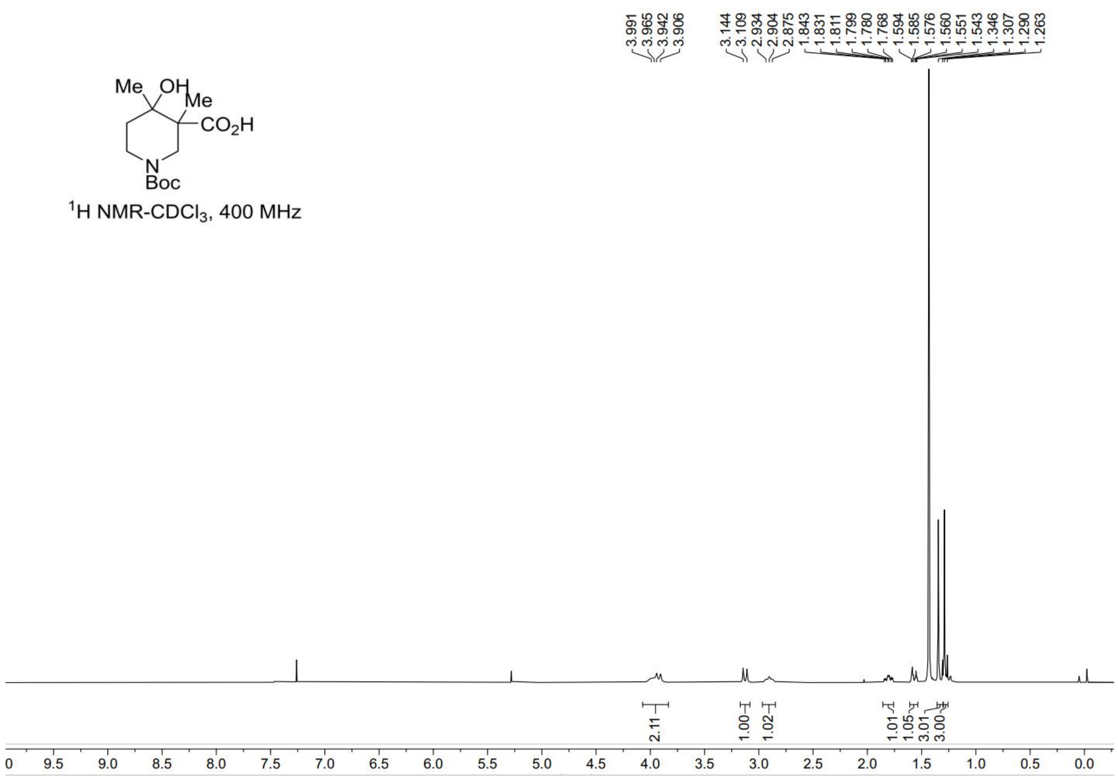

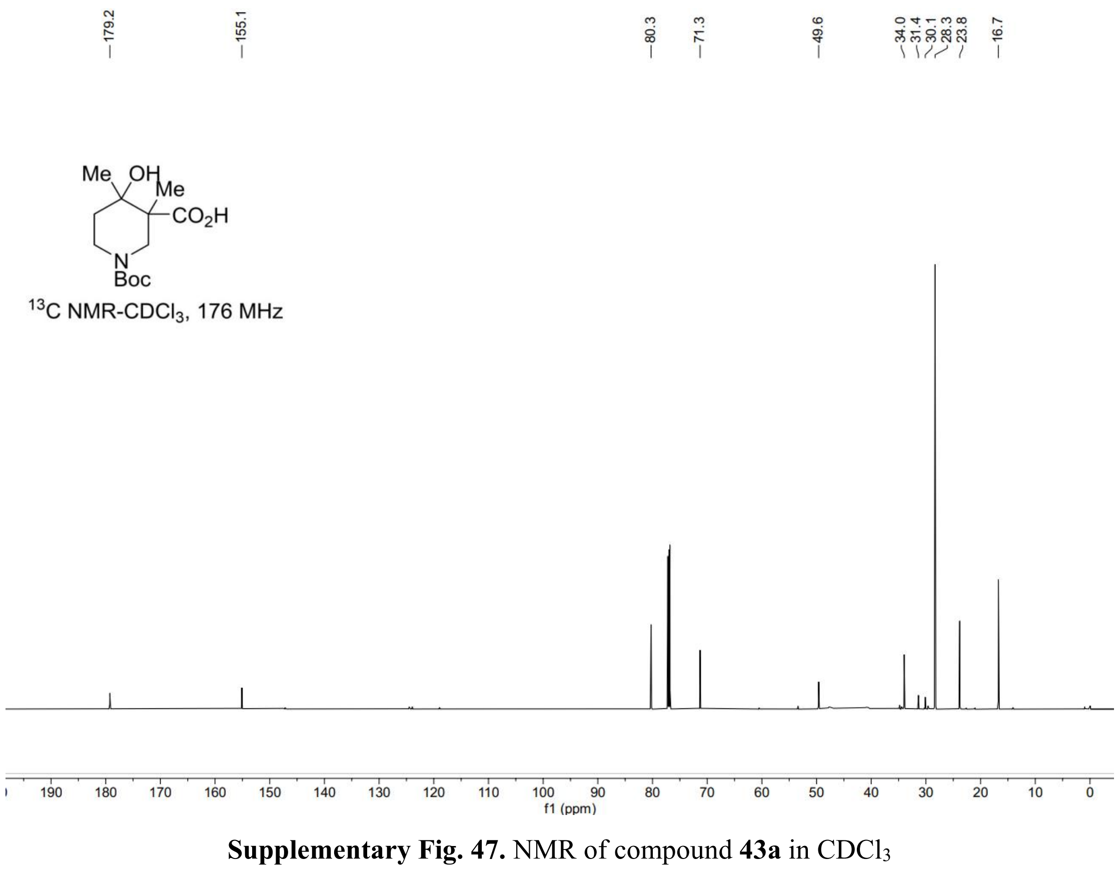

Supplementary Fig. 47. NMR of compound **43a** in $CDCl_3$

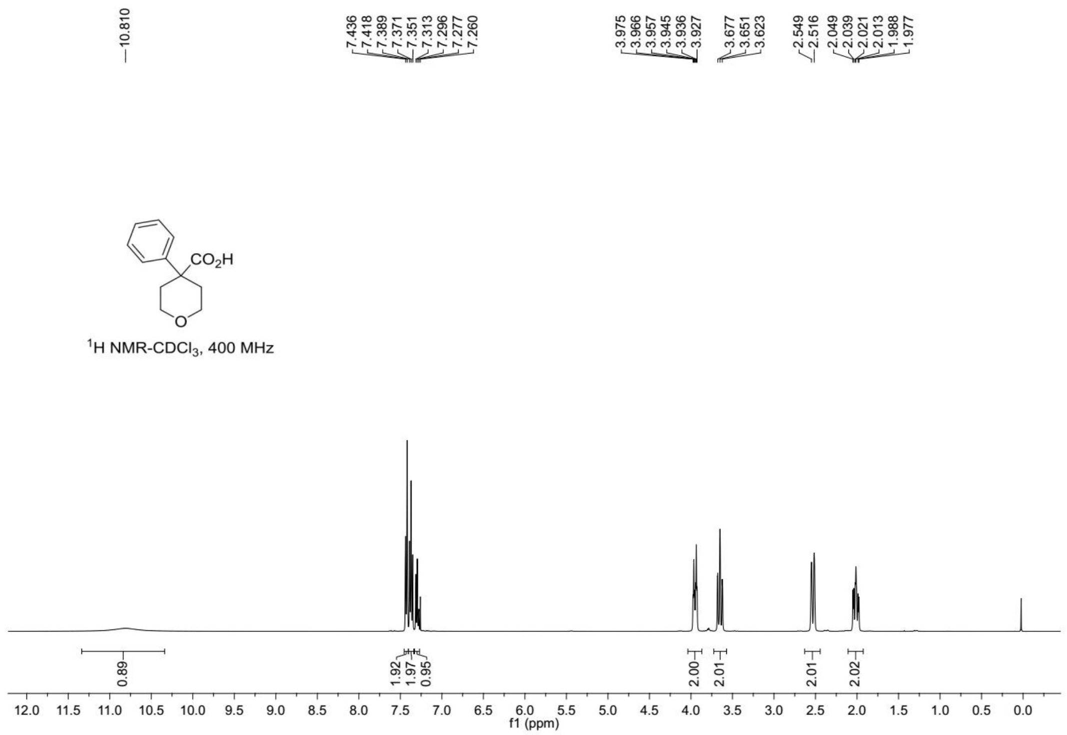

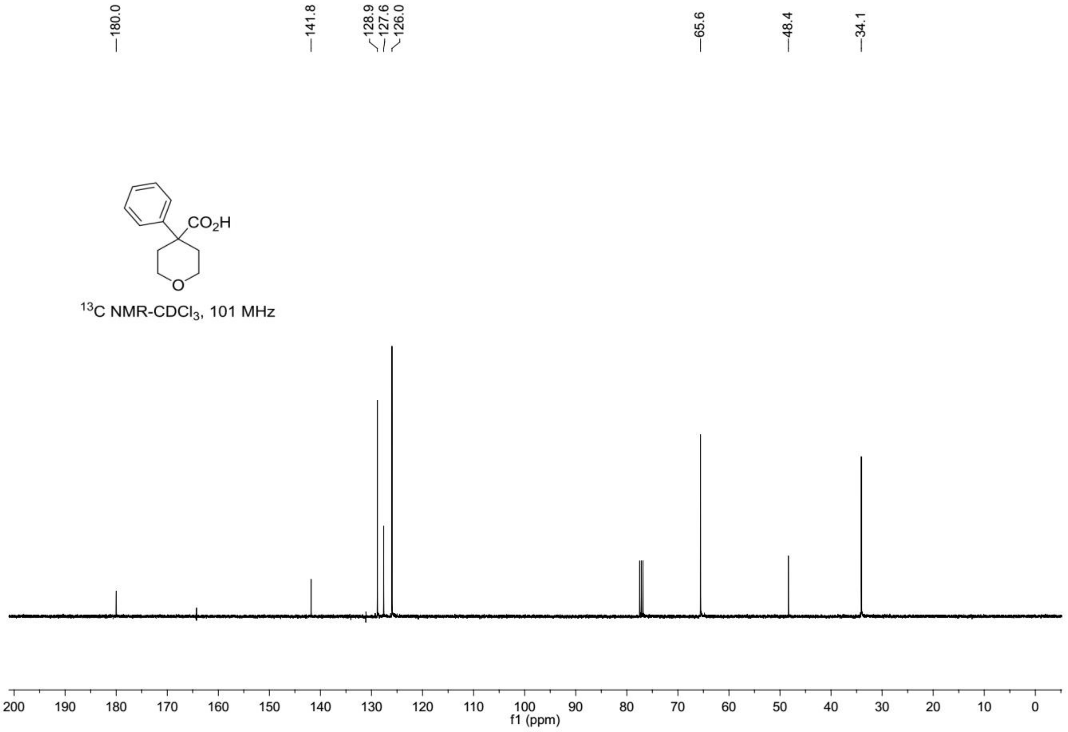

Supplementary Fig. 48. NMR of compound **49a** in $CDCl_3$

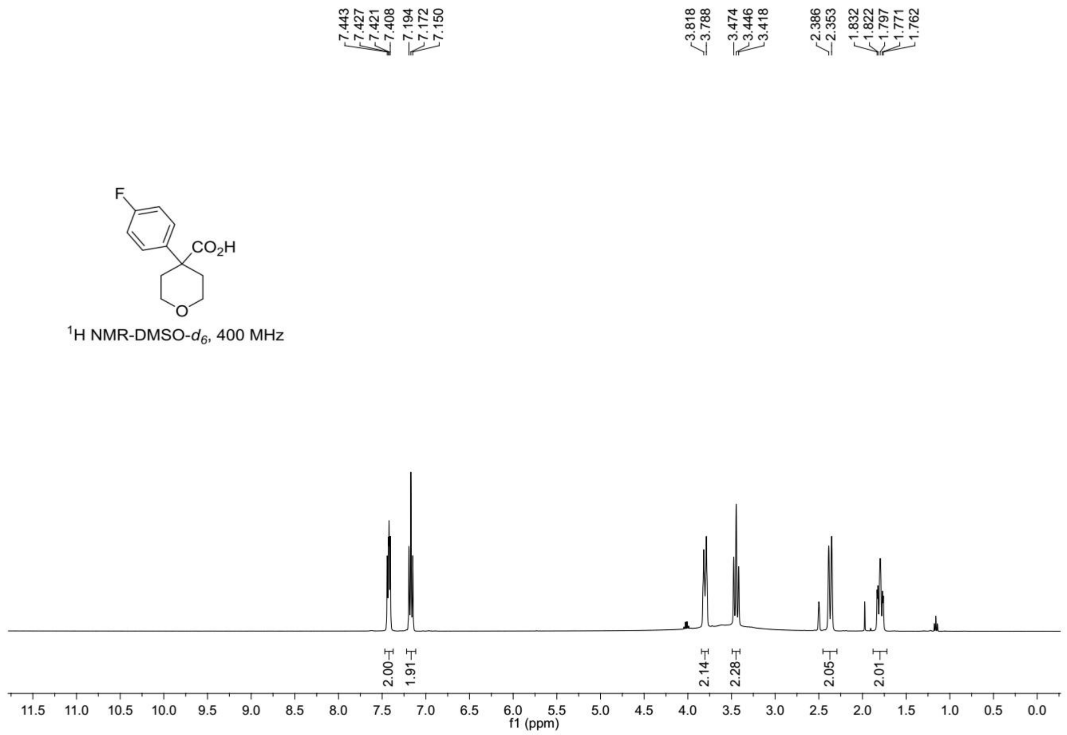

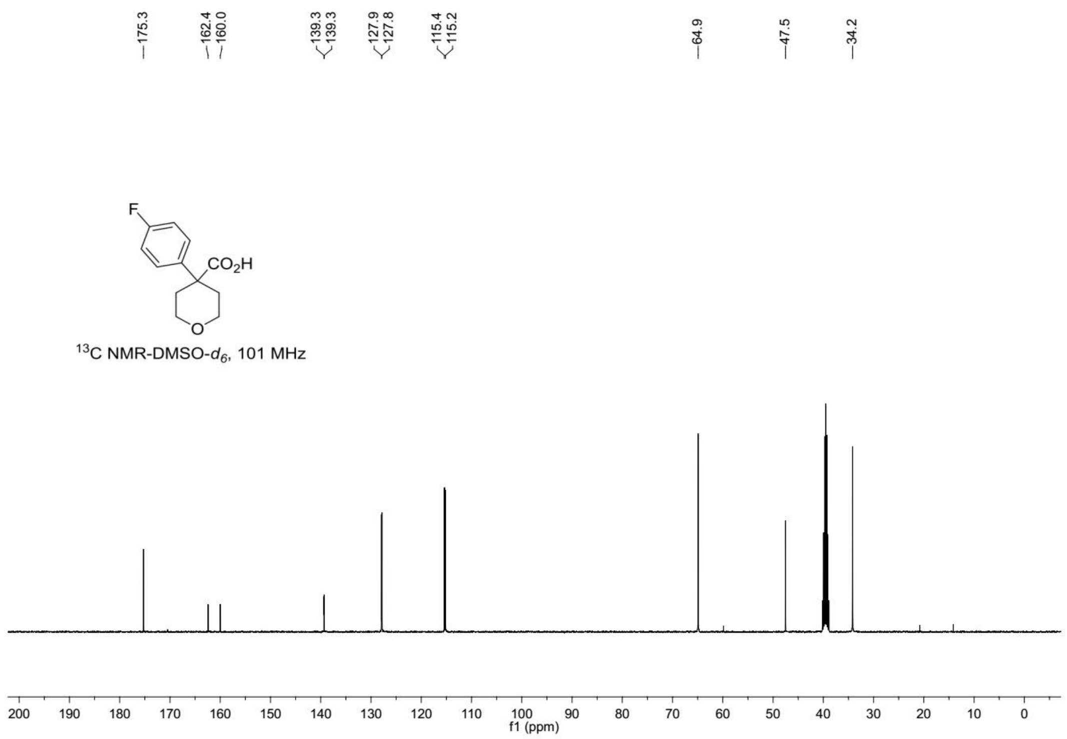

Supplementary Fig. 49. NMR of compound **50a** in DMSO-d_6

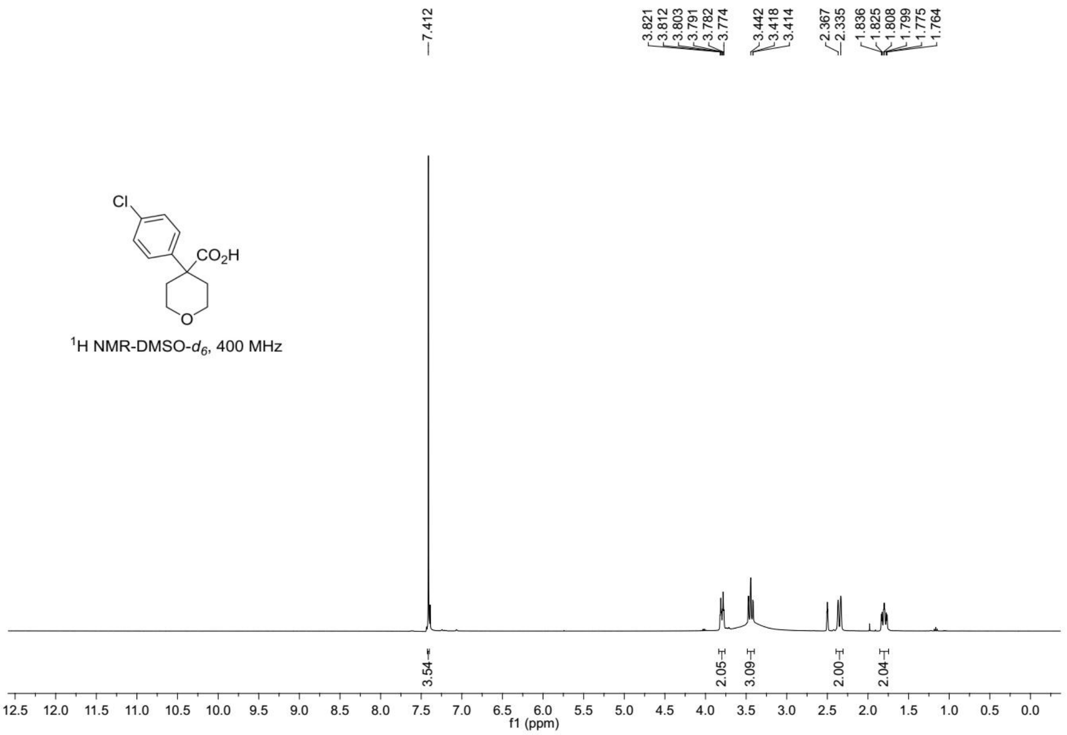

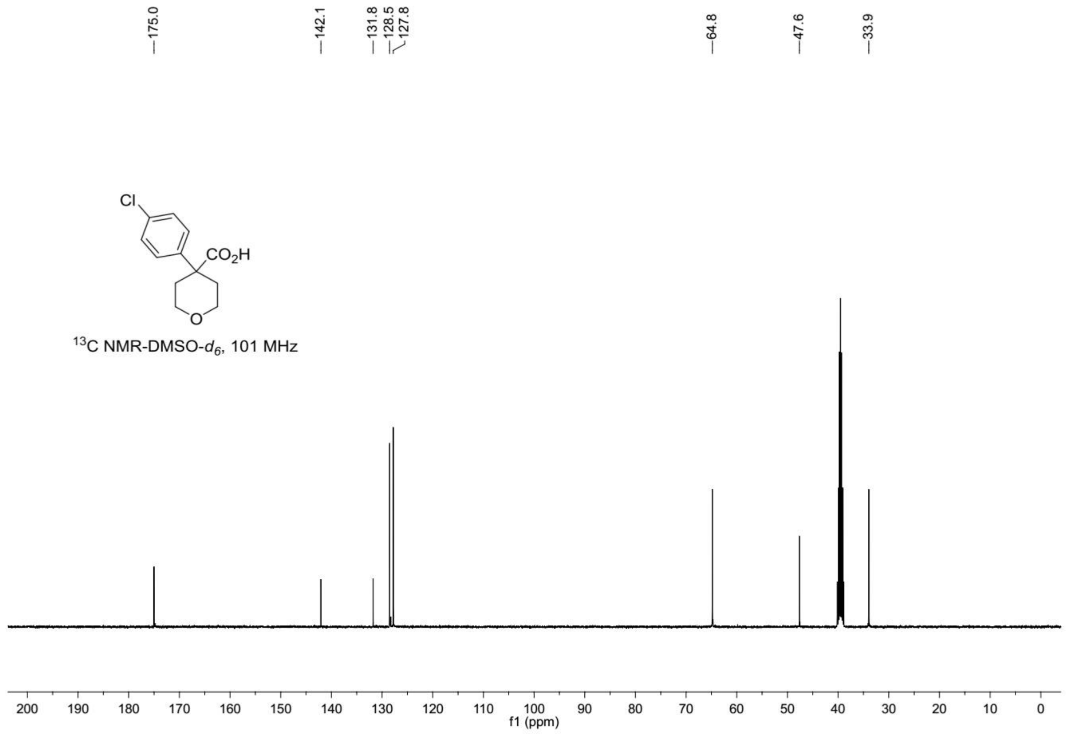

Supplementary Fig. 50. NMR of compound **51a** in DMSO-d_6

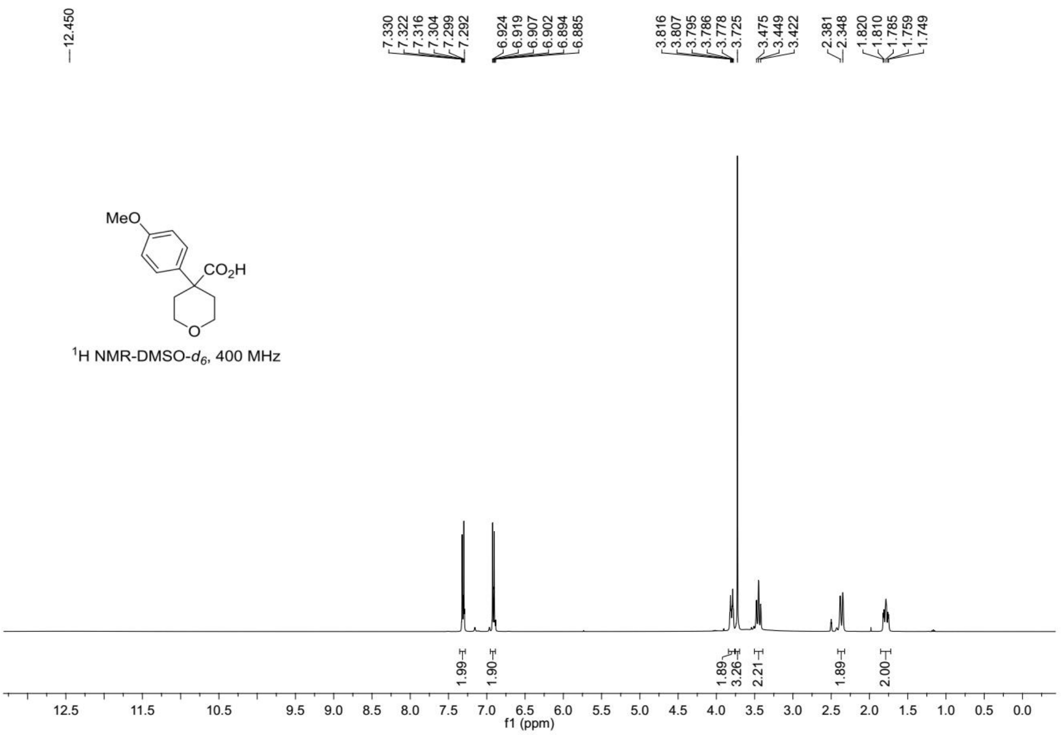

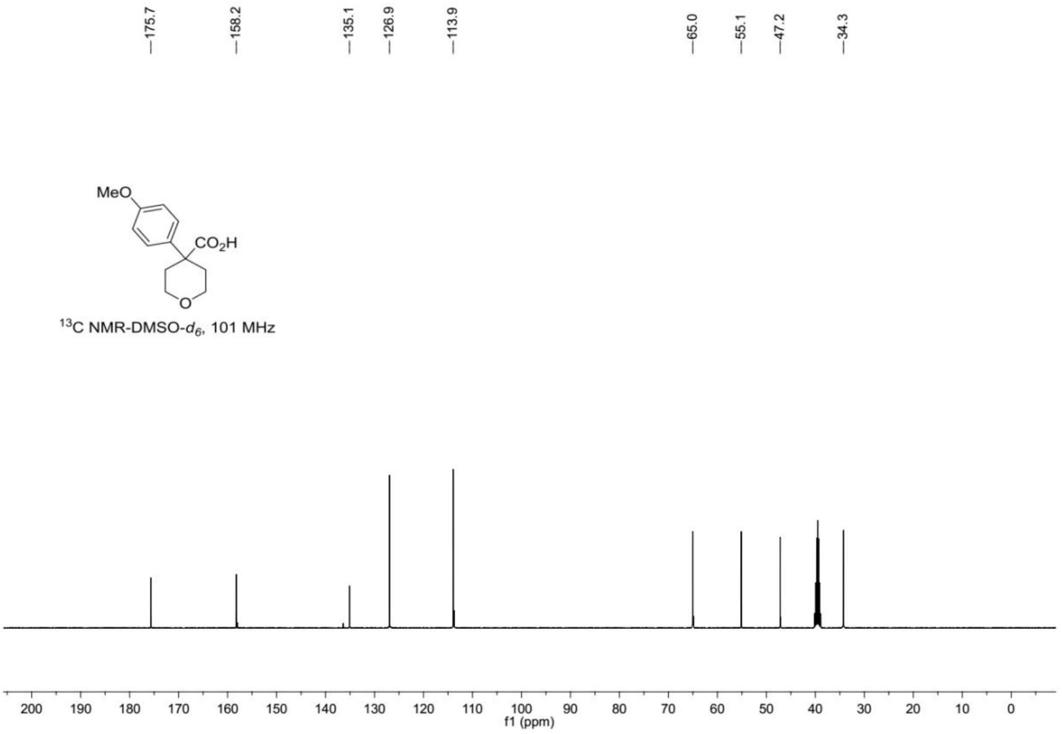

Supplementary Fig. 51. NMR of compound **52a** in DMSO-d_6

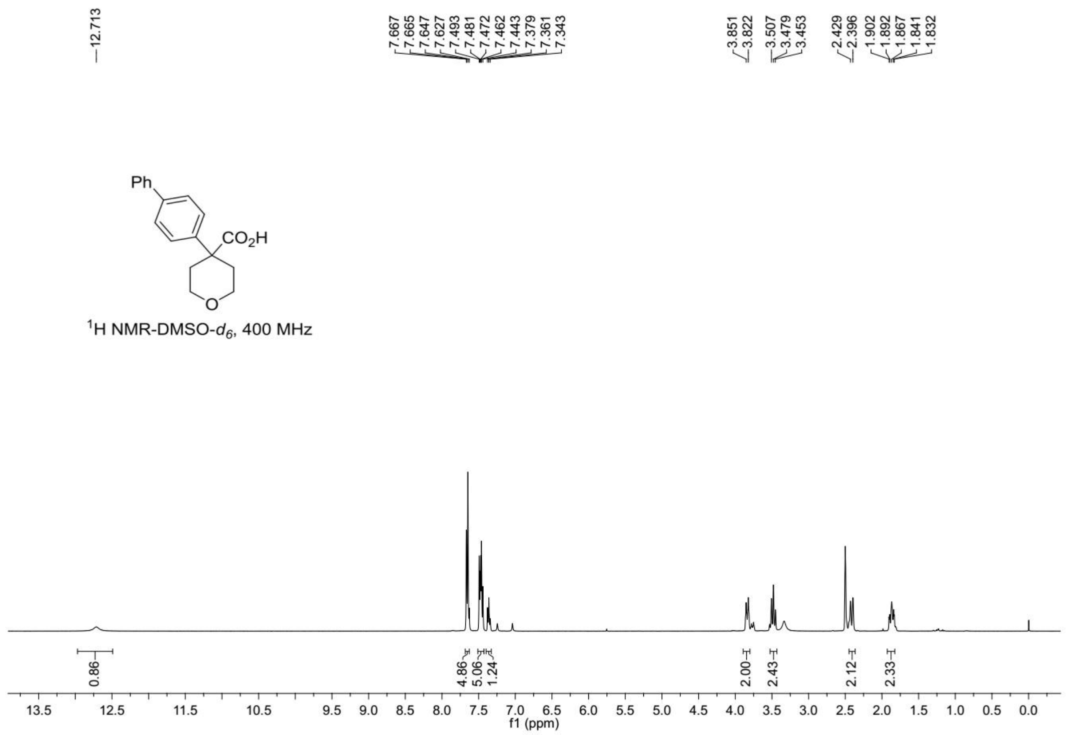

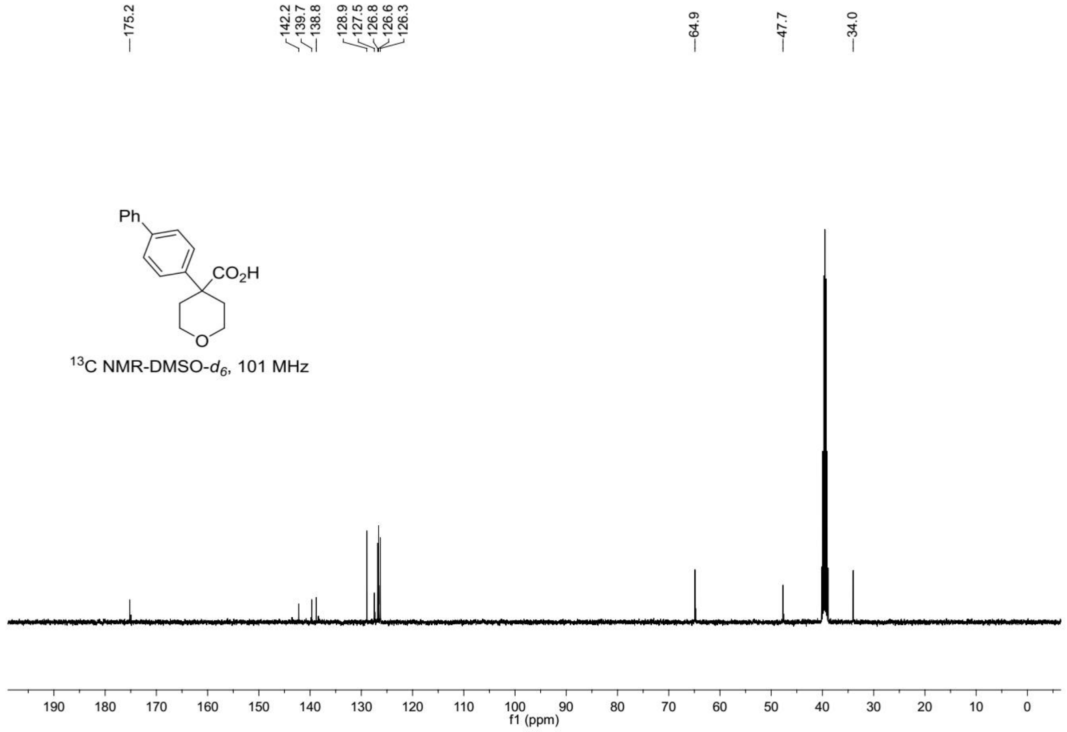

Supplementary Fig. 52. NMR of compound **53a** in DMSO-d_6

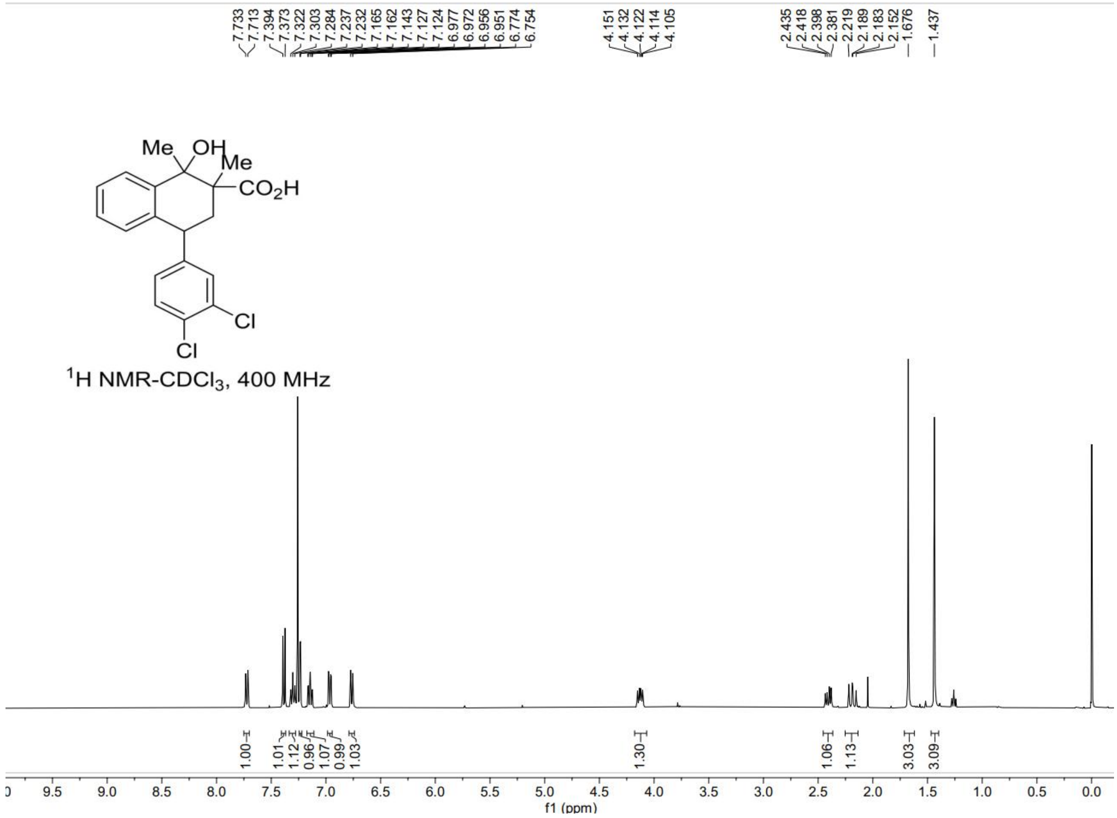

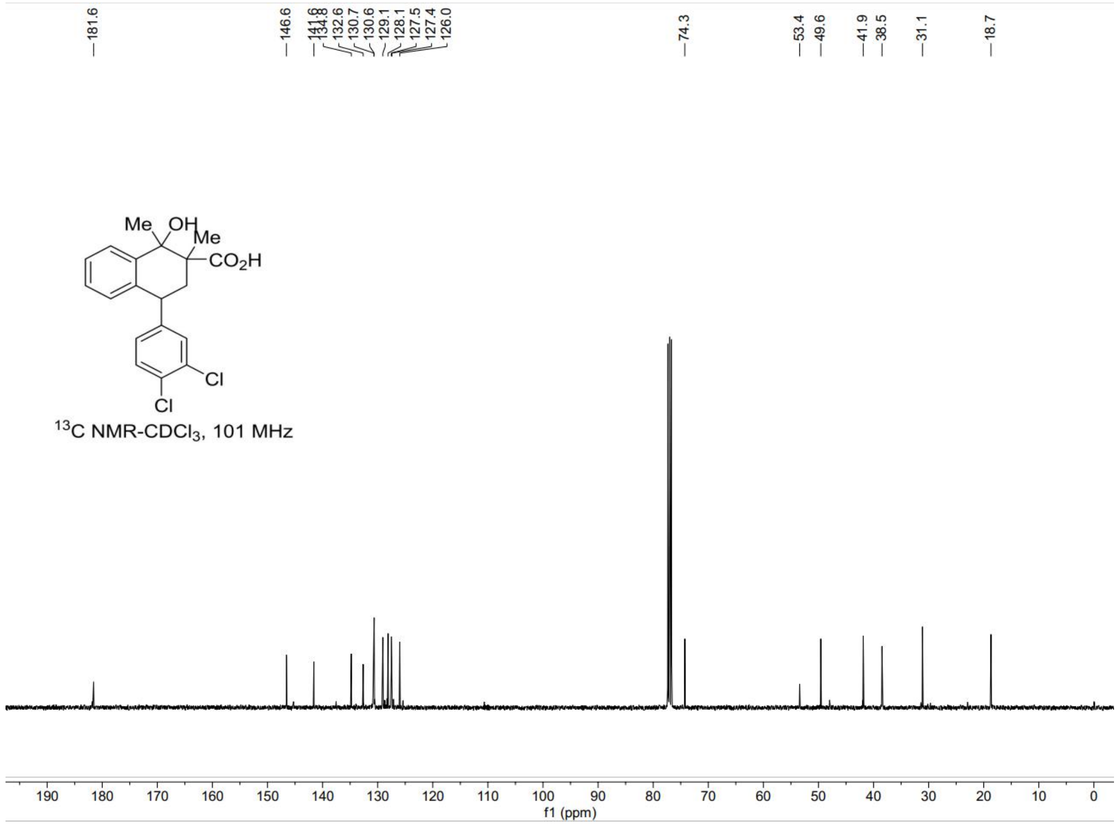

Supplementary Fig. 53. NMR of compound **54a** in $CDCl_3$

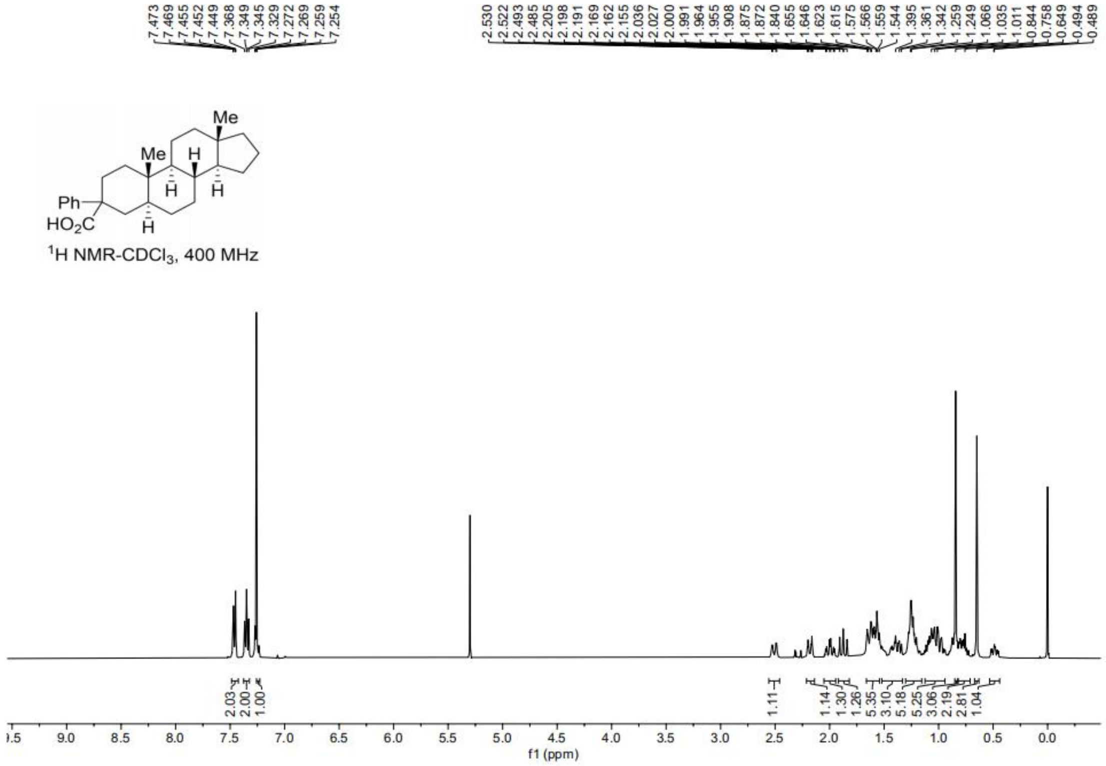

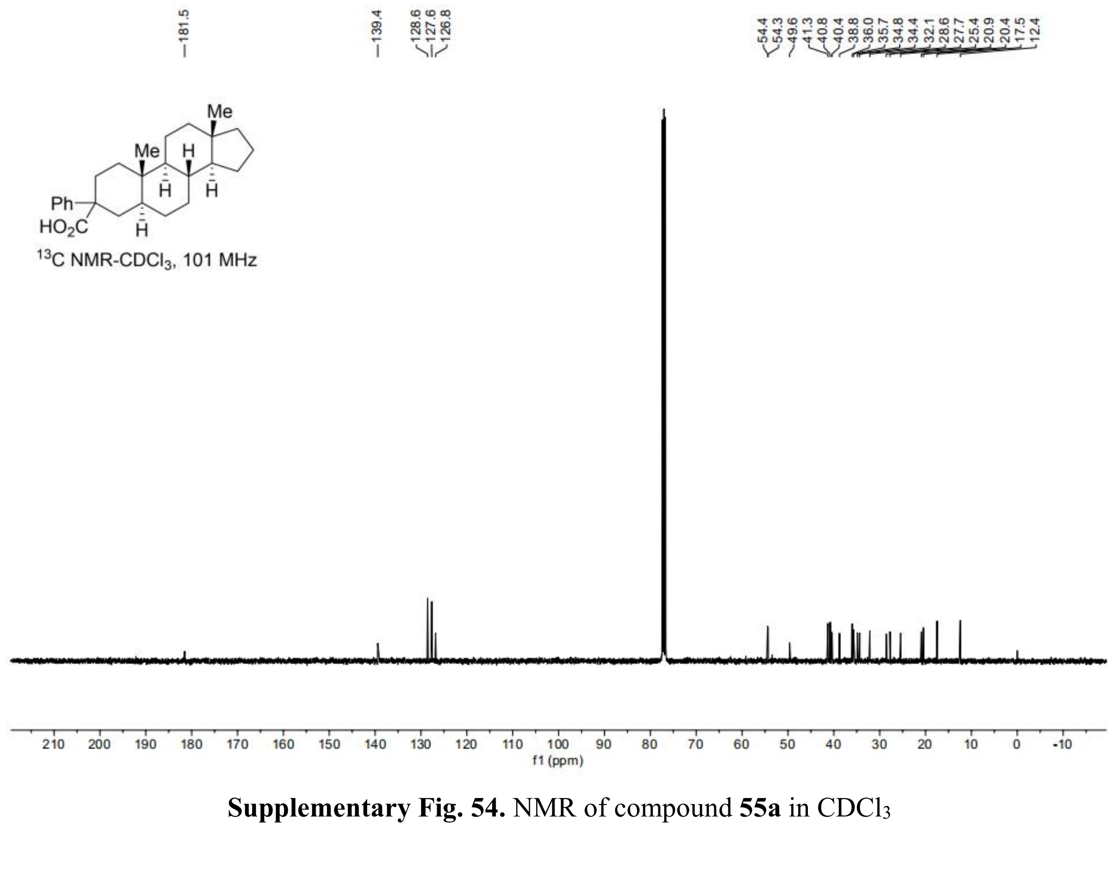

Supplementary Fig. 54. NMR of compound **55a** in $CDCl_3$

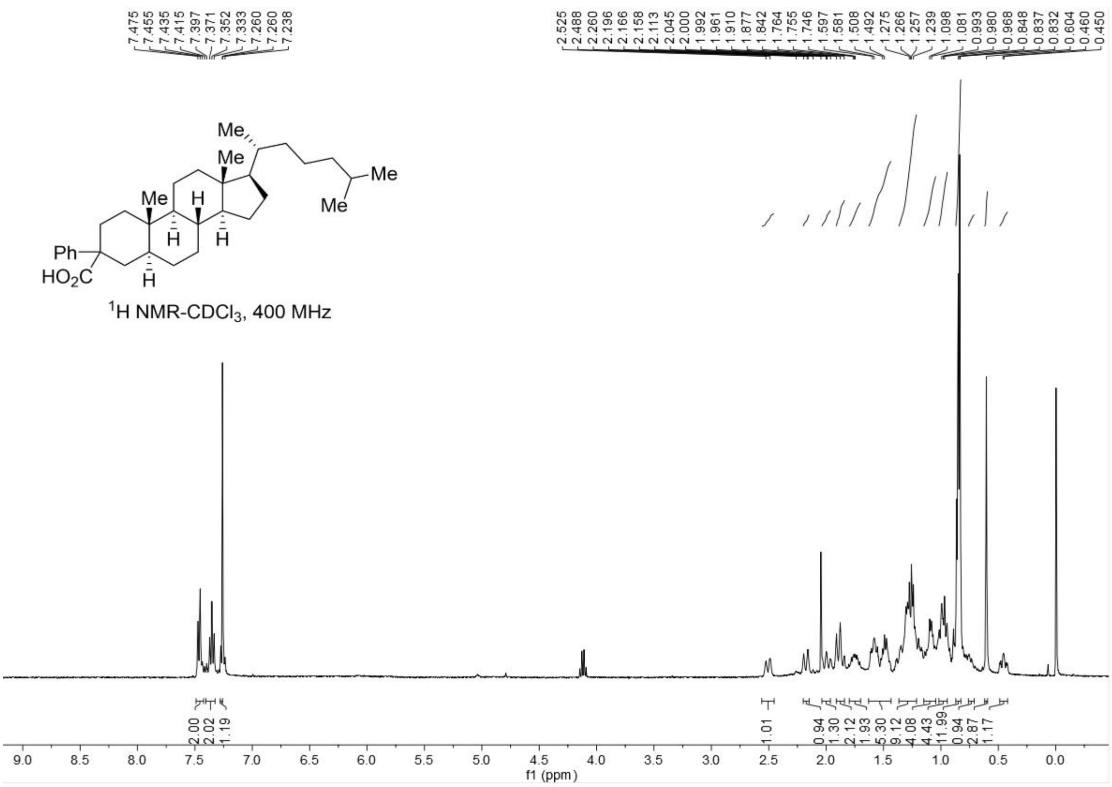

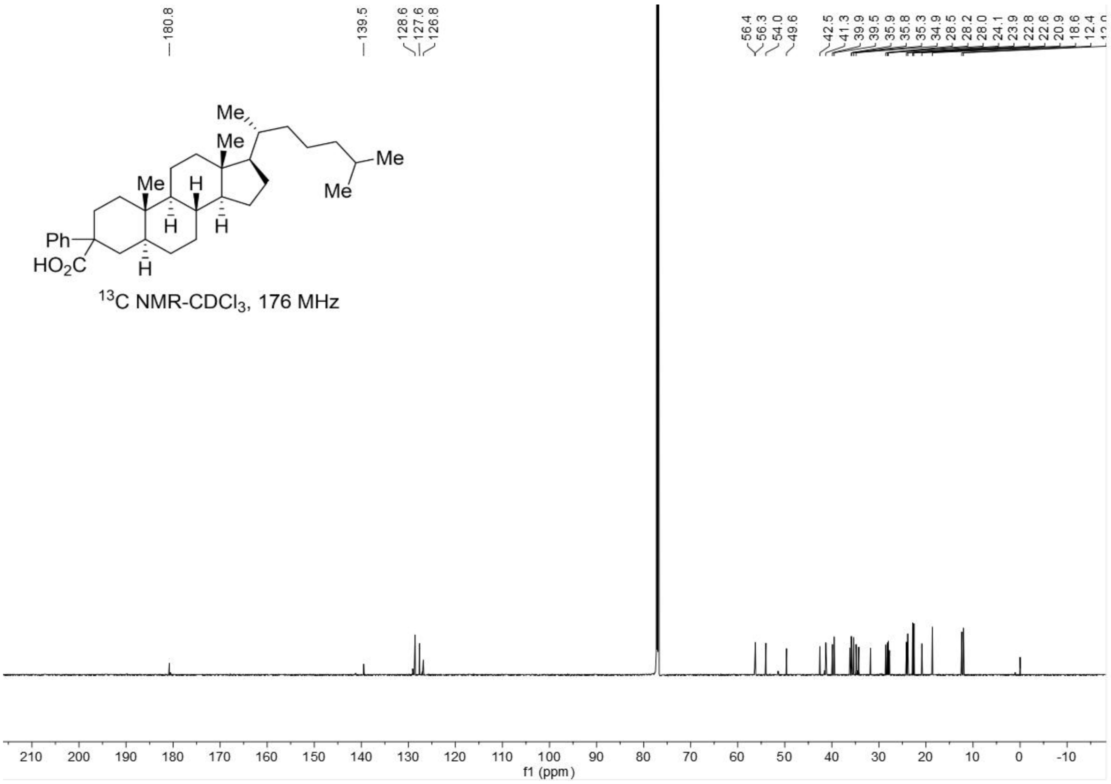

Supplementary Fig. 55. NMR of compound **56a** in $CDCl_3$

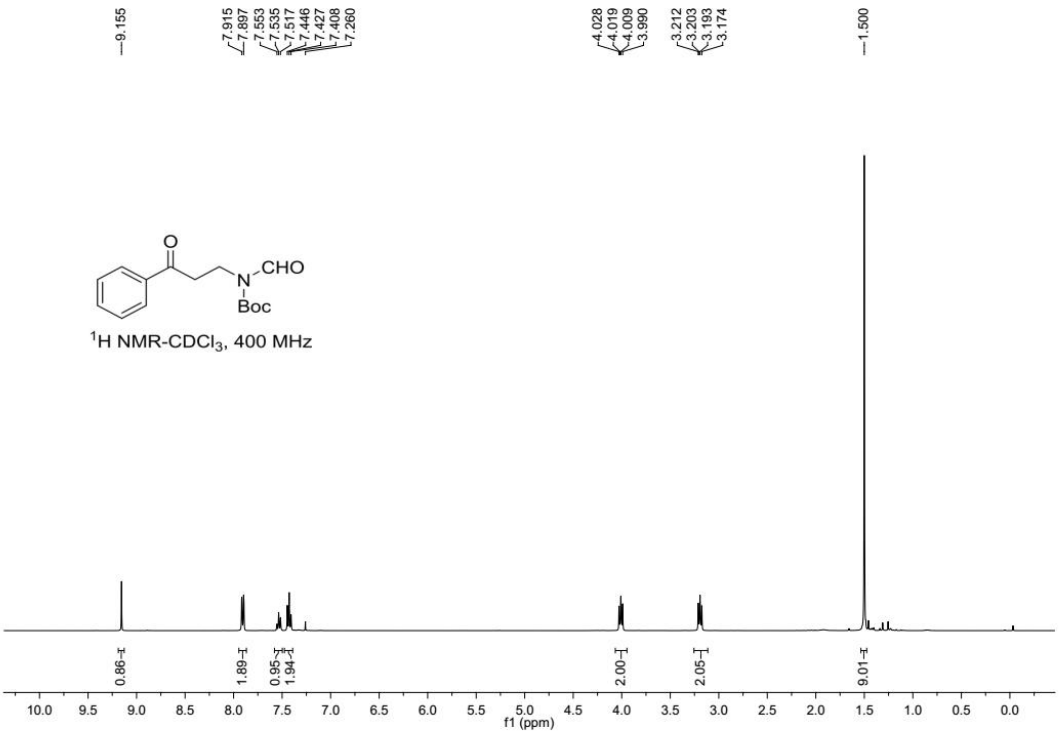

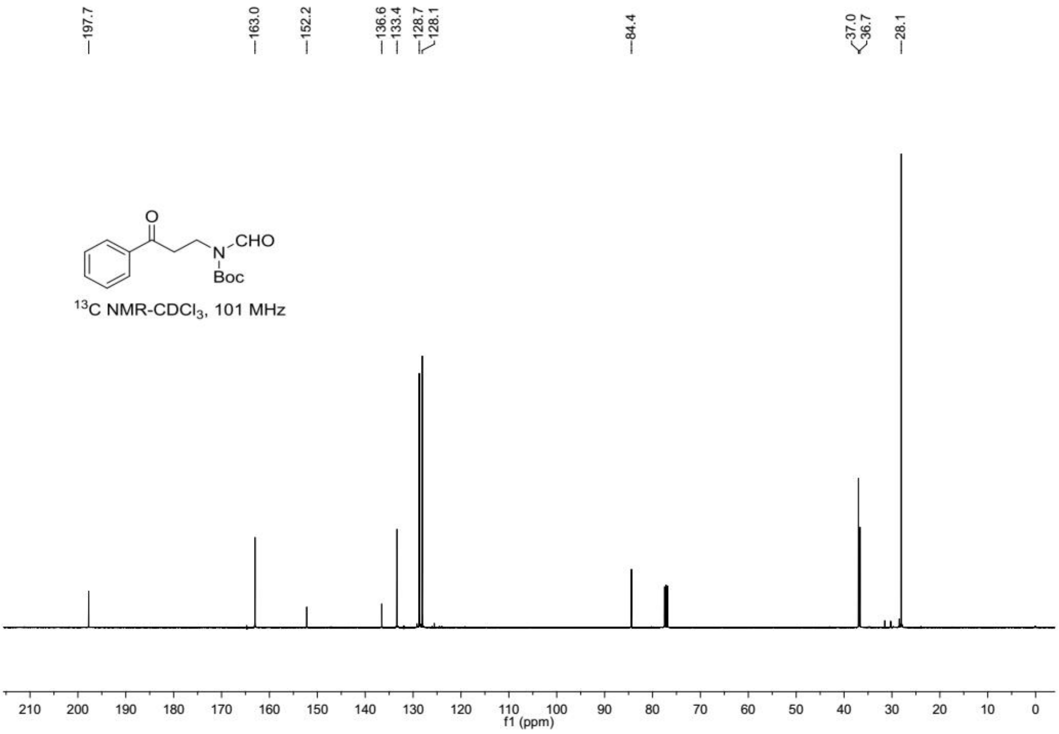

Supplementary Fig. 56. NMR of compound **1b** in $CDCl_3$

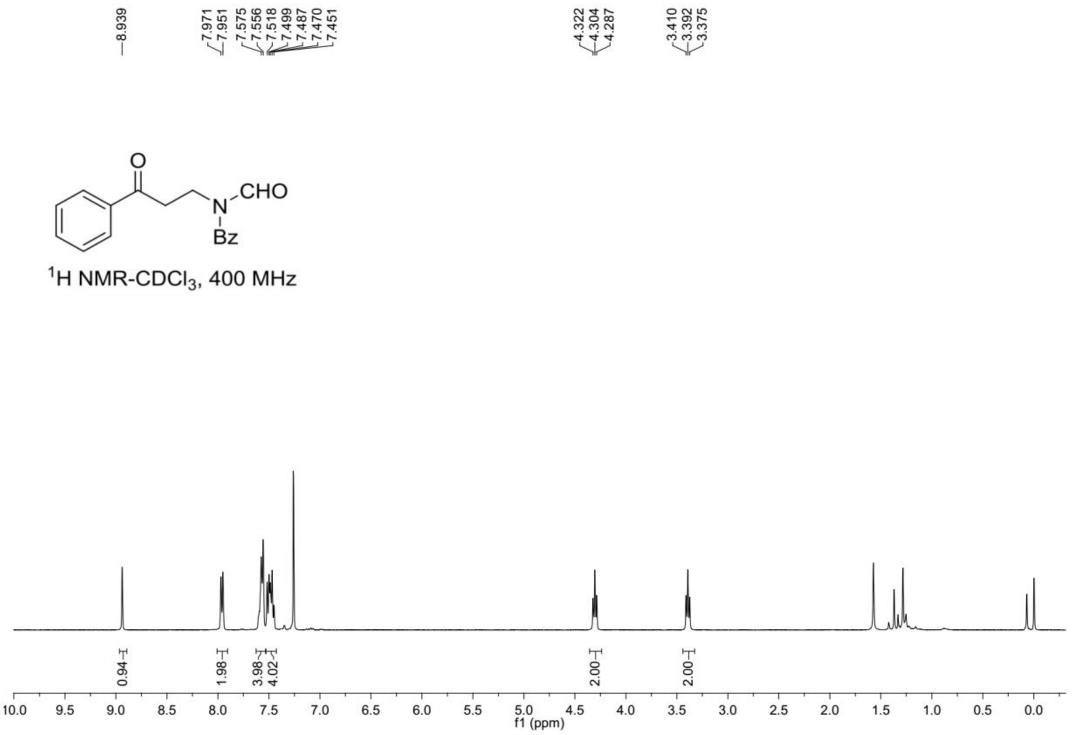

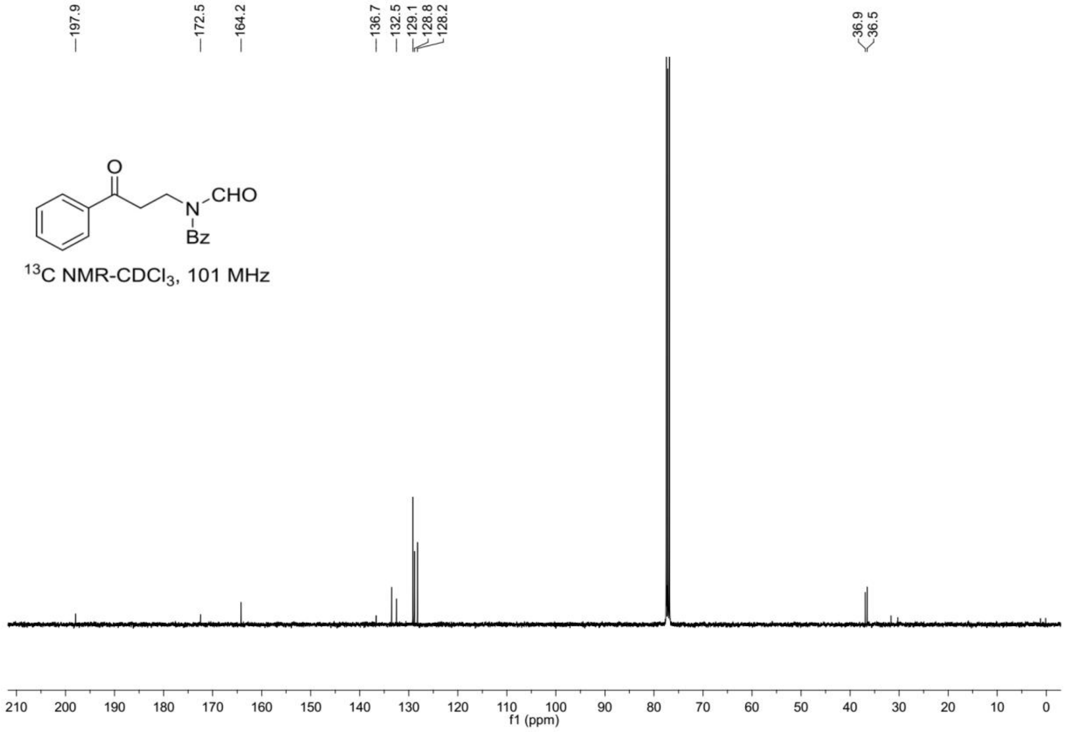

Supplementary Fig. 57. NMR of compound **2b** in $CDCl_3$

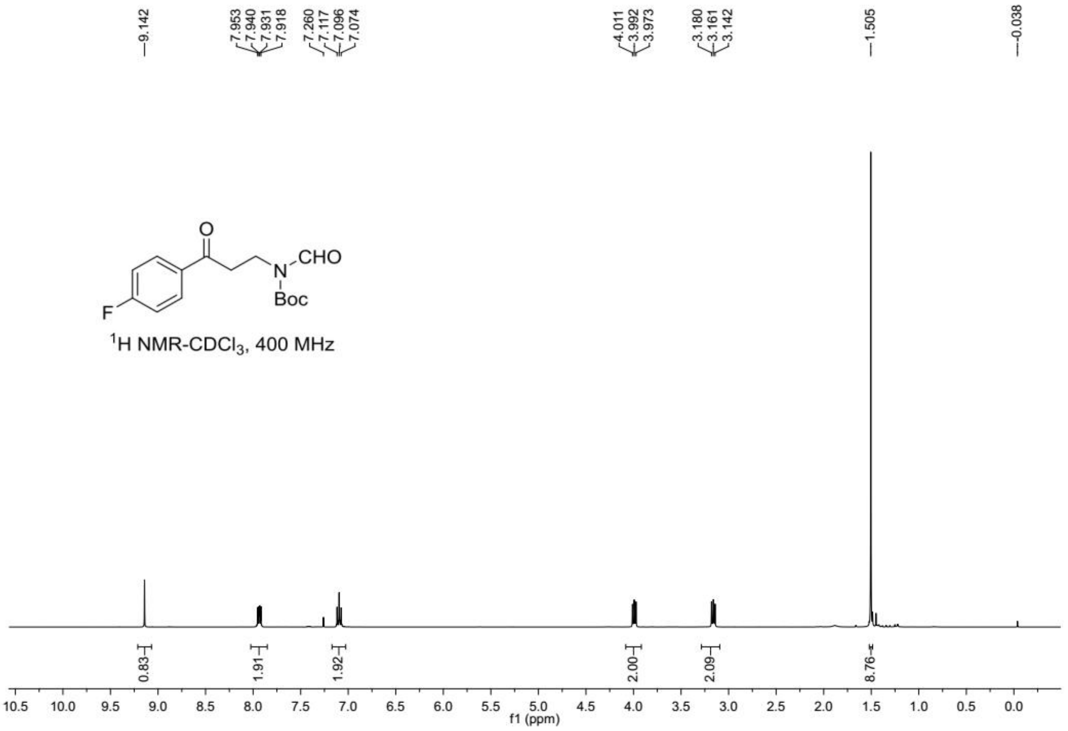

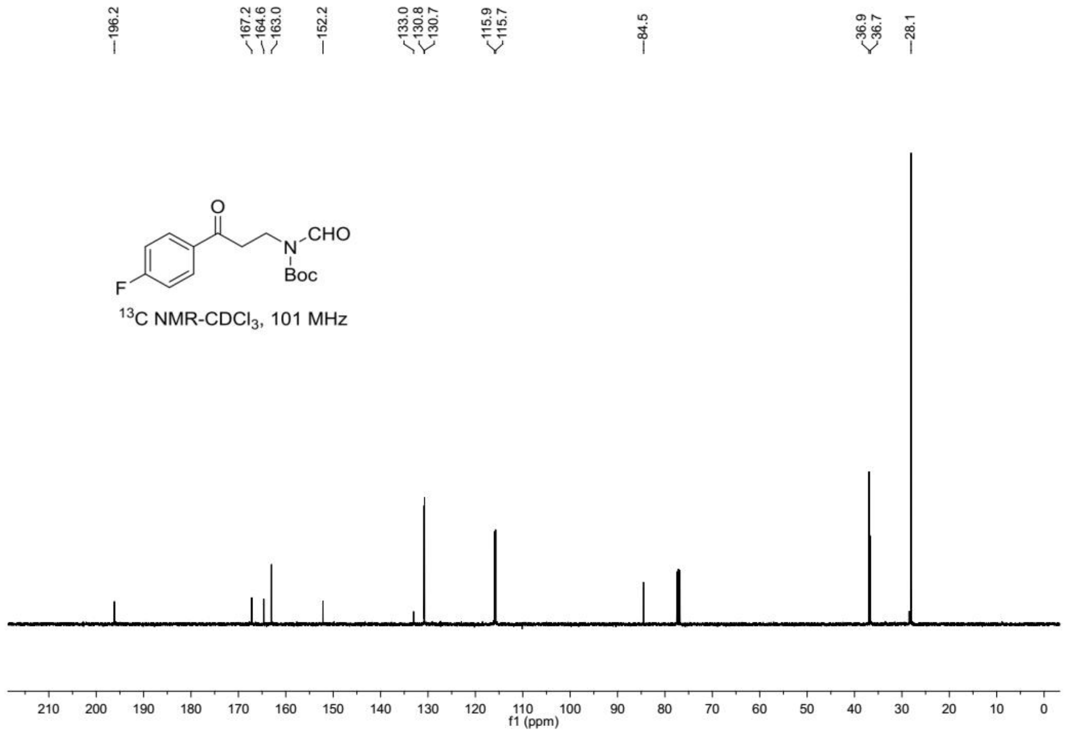

Supplementary Fig. 58. NMR of compound **3b** in $CDCl_3$

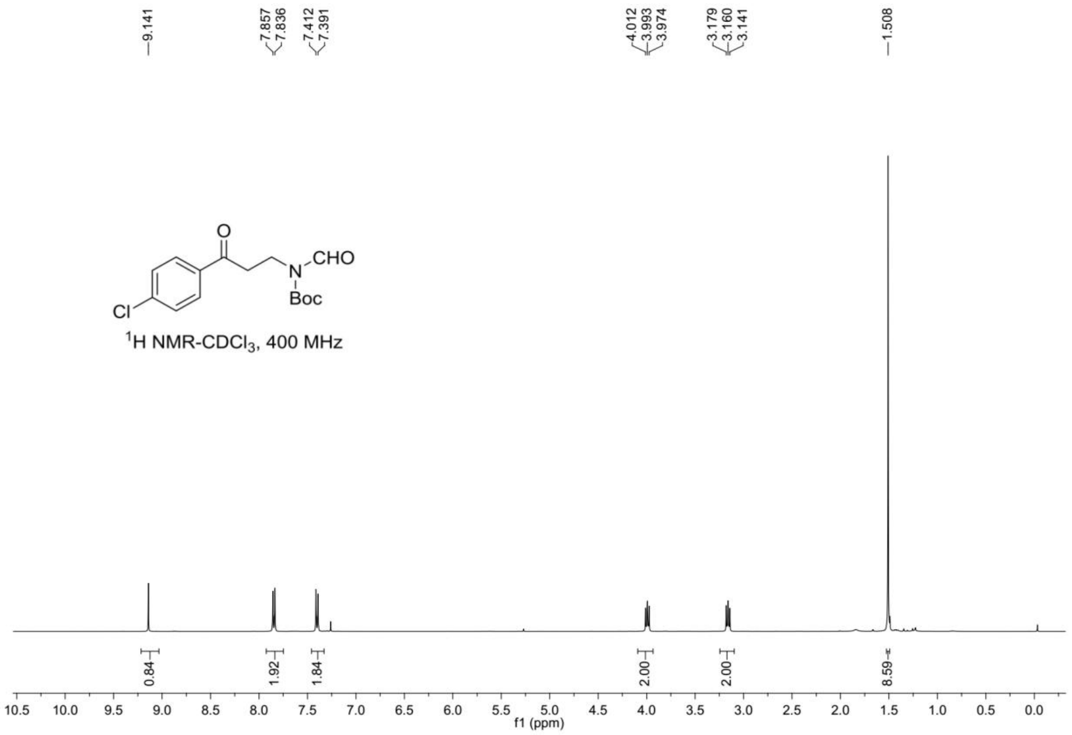

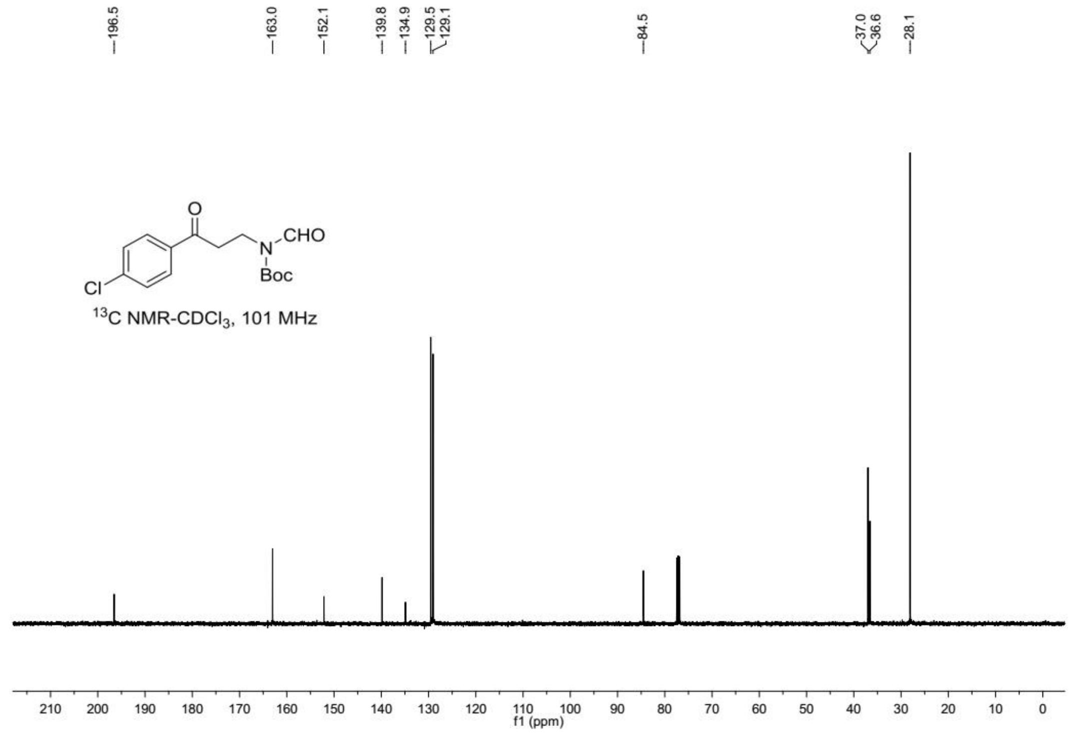

Supplementary Fig. 59. NMR of compound **4b** in $CDCl_3$

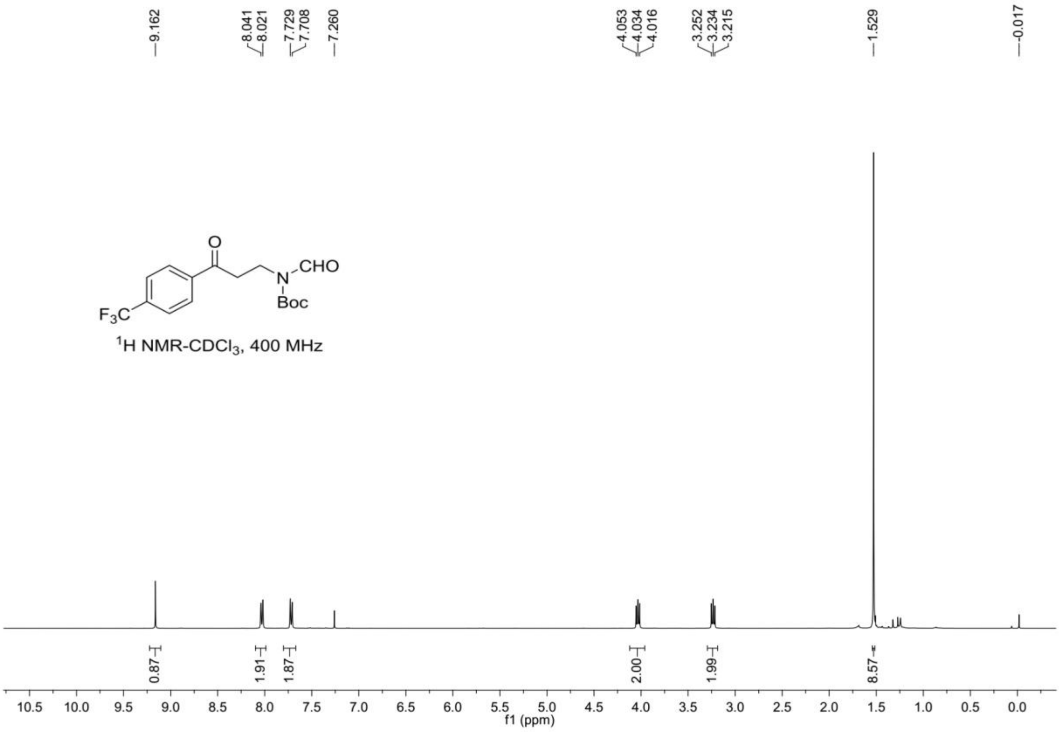

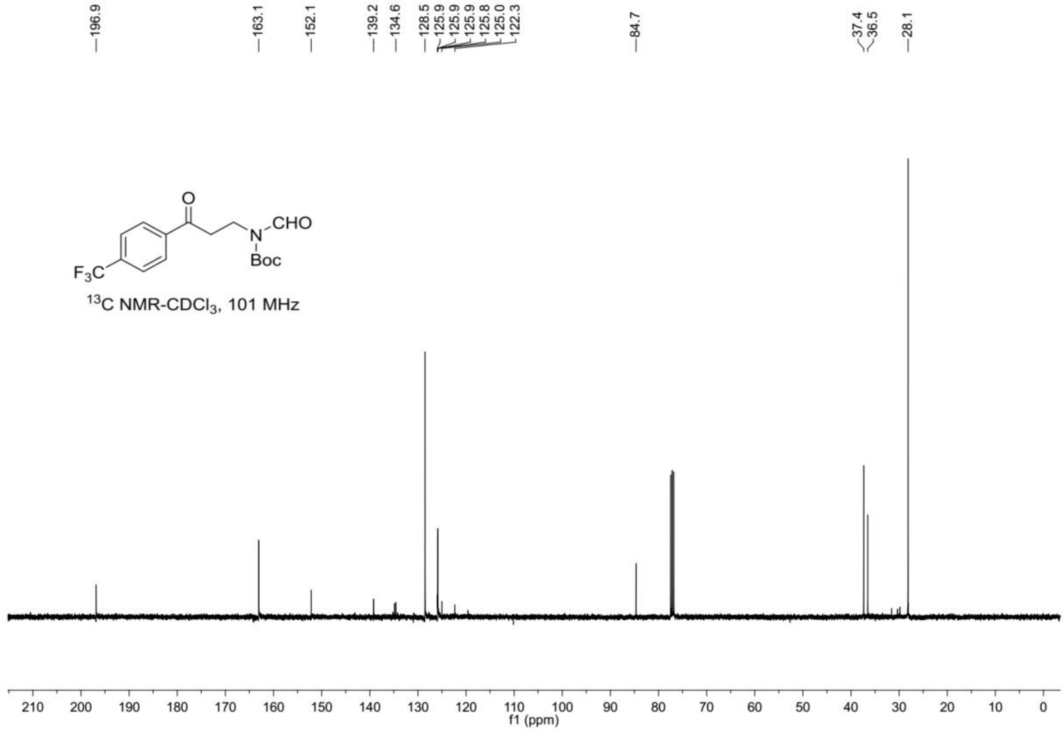

Supplementary Fig. 60. NMR of compound **5b** in $CDCl_3$

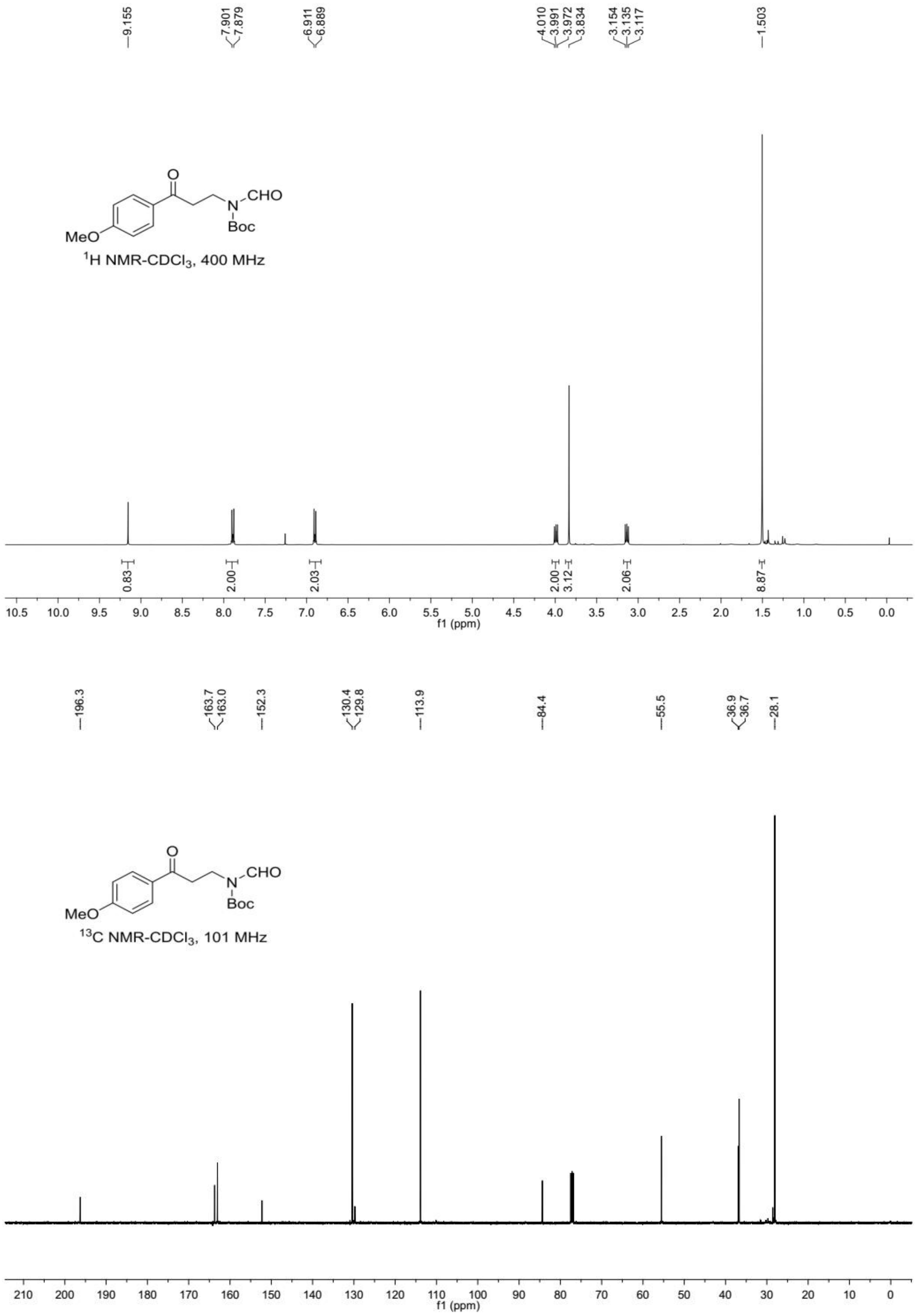

Supplementary Fig. 61. NMR of compound **6b** in $CDCl_3$

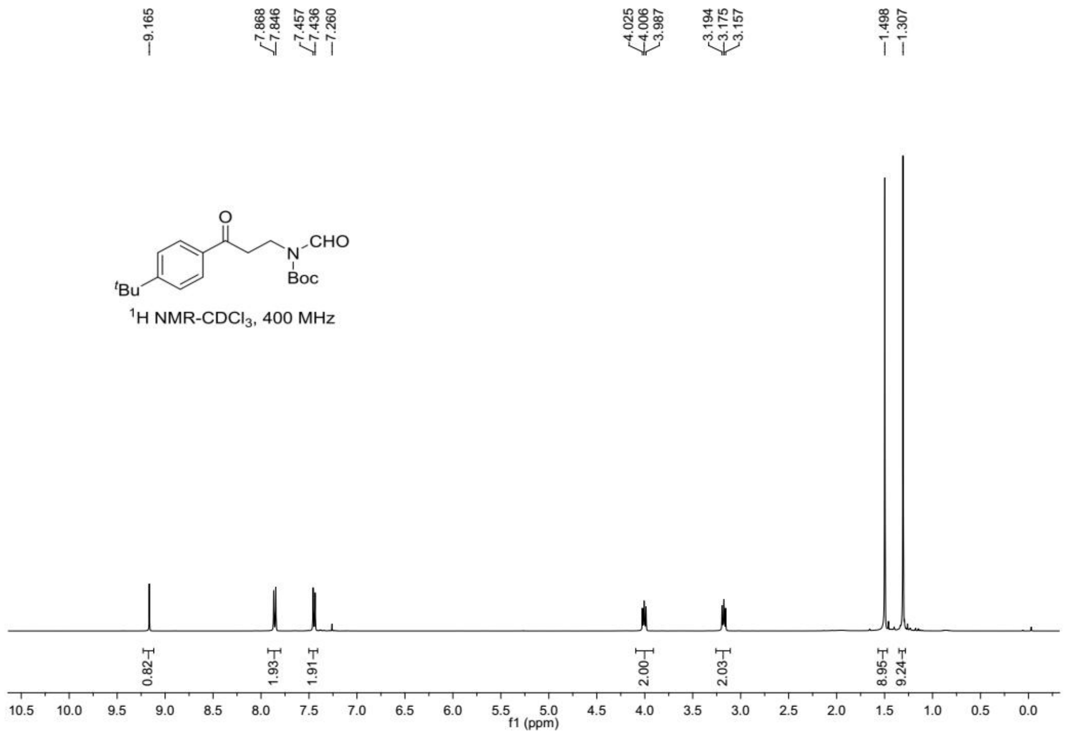

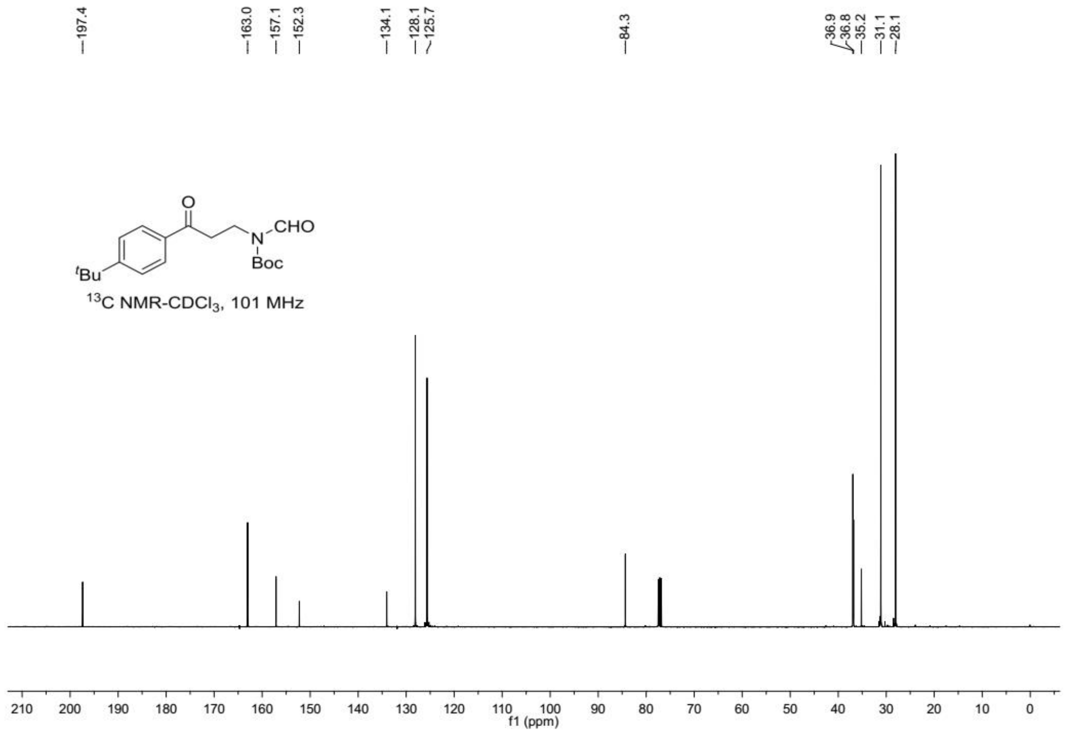

Supplementary Fig. 62. NMR of compound **7b** in $CDCl_3$

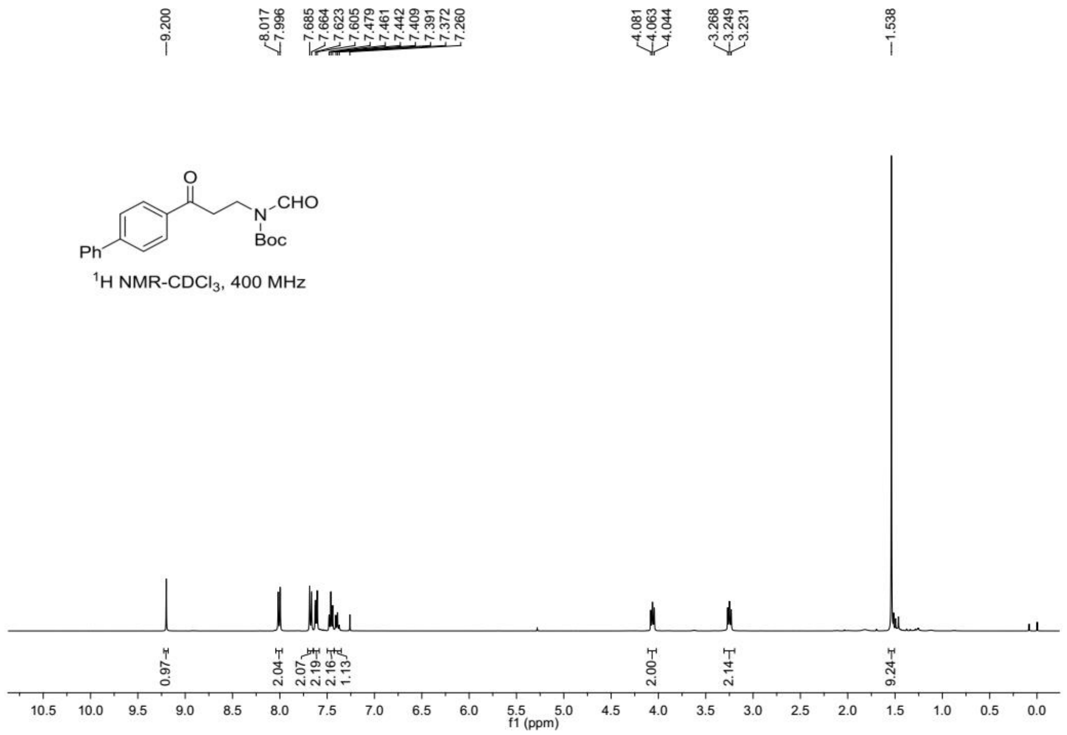

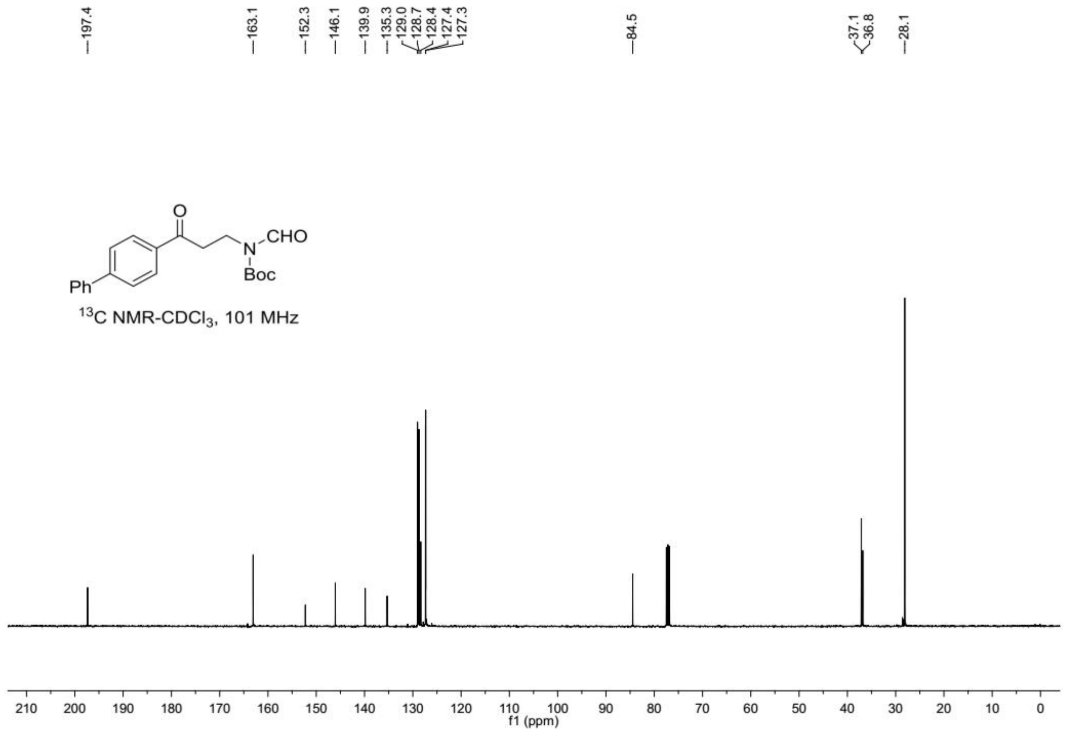

Supplementary Fig. 63. NMR of compound **8b** in $CDCl_3$

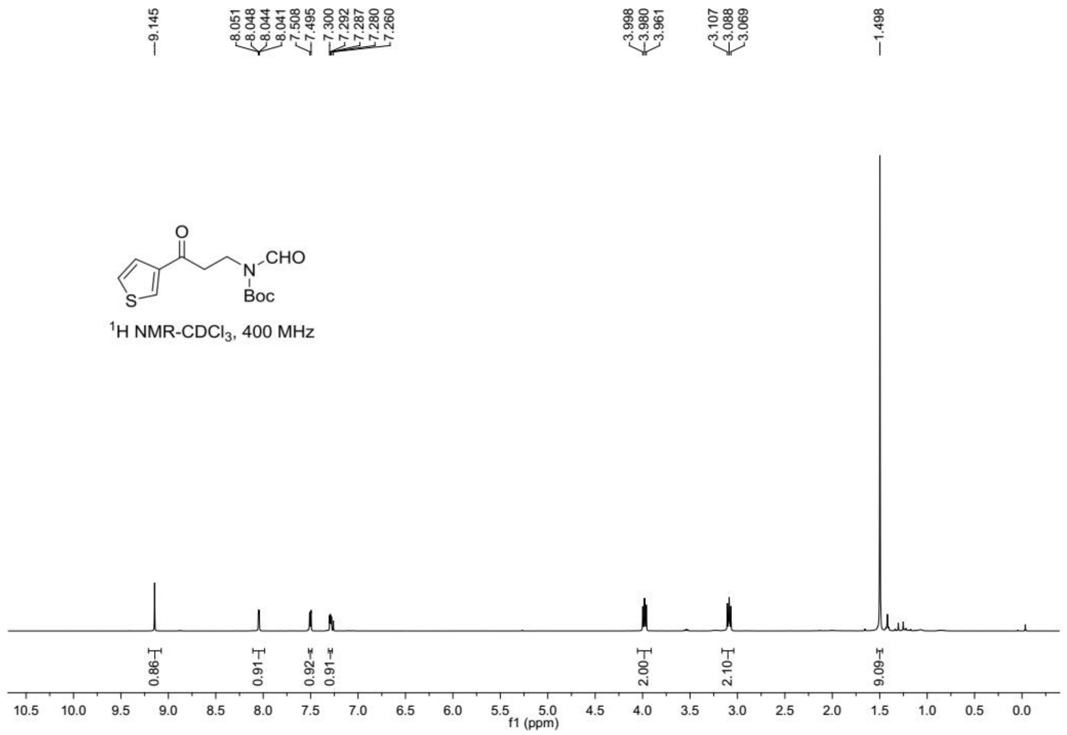

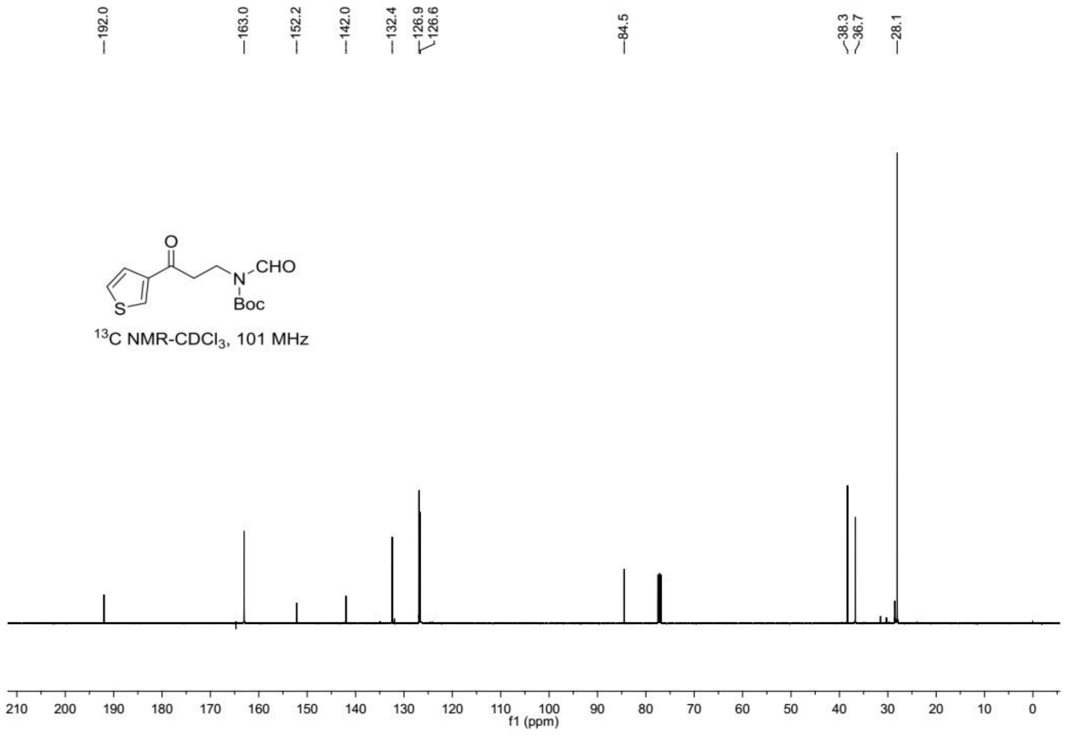

Supplementary Fig. 64. NMR of compound **9b** in $CDCl_3$

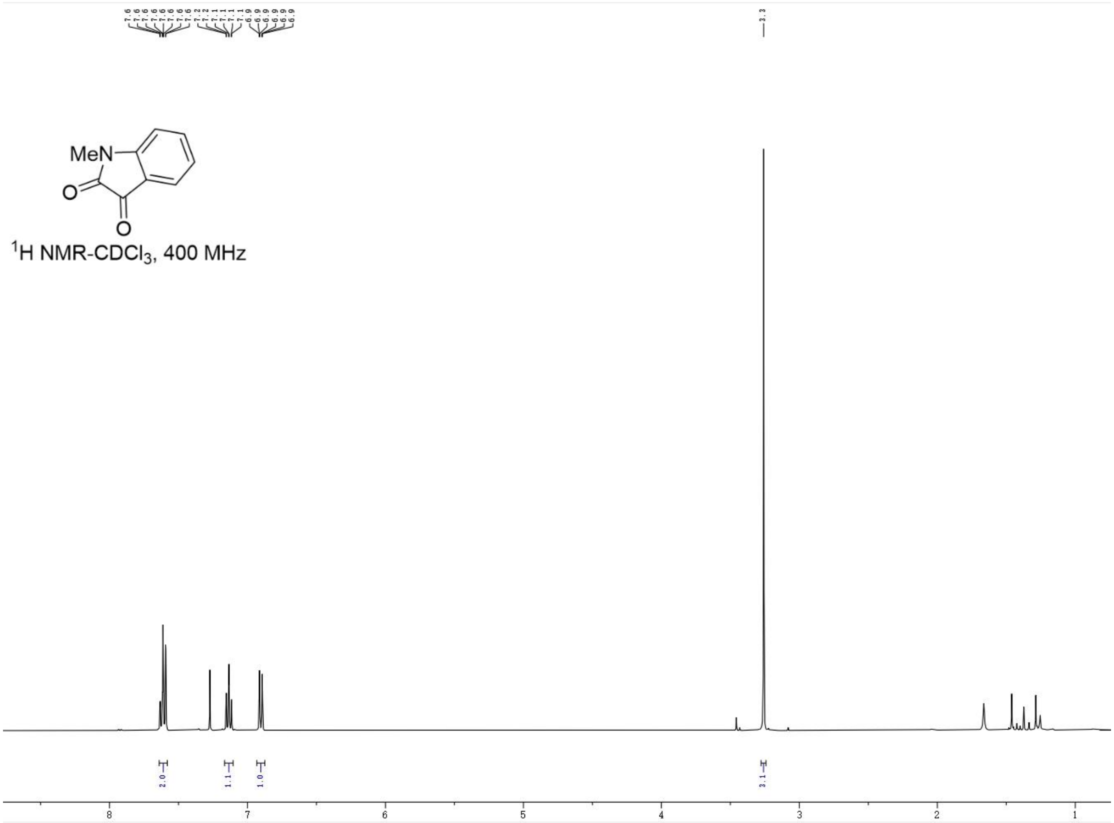

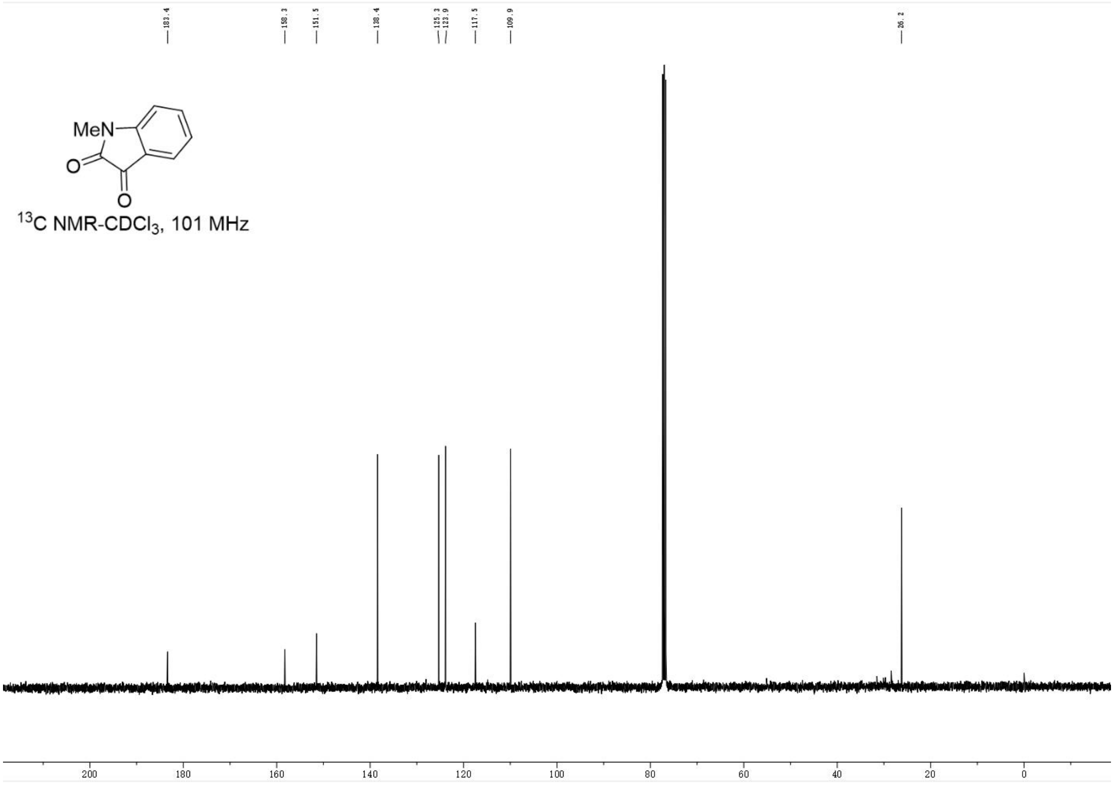

Supplementary Fig. 65. NMR of compound **10b** in $CDCl_3$

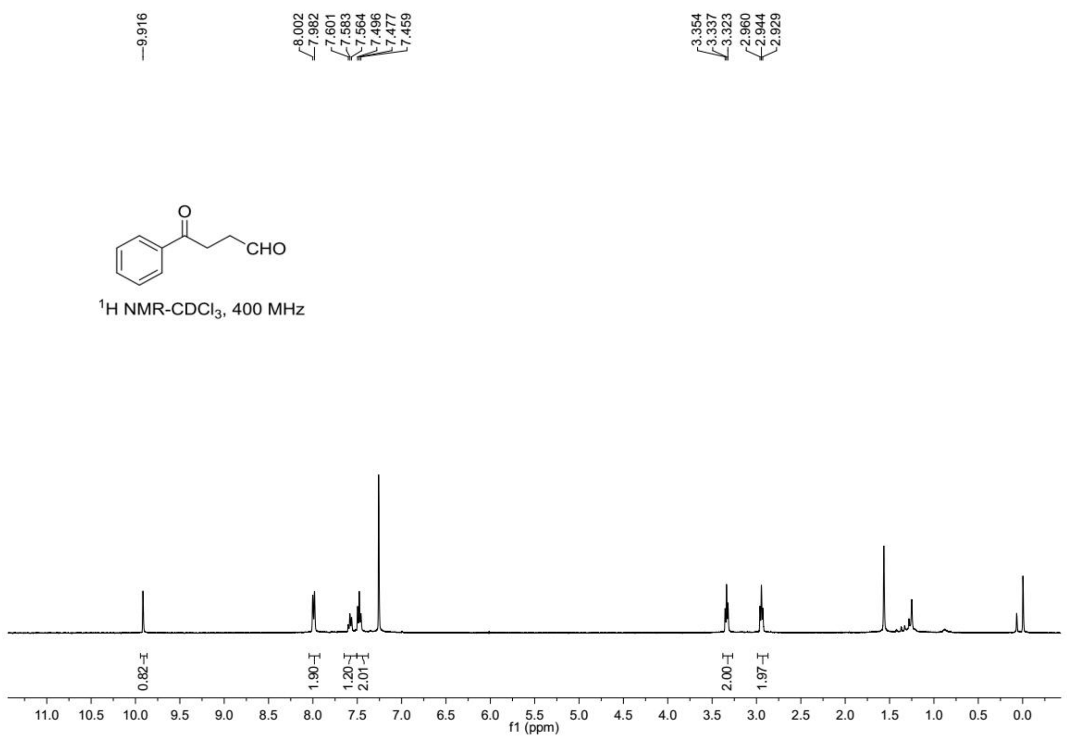

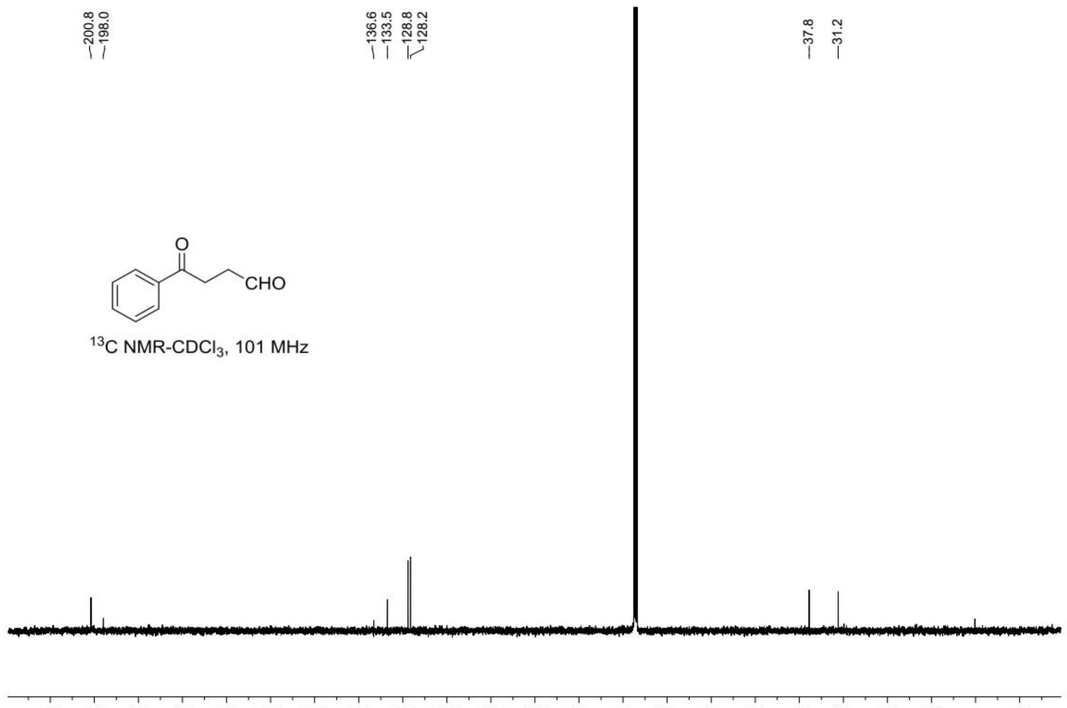

Supplementary Fig. 66. NMR of compound **11b** in $CDCl_3$

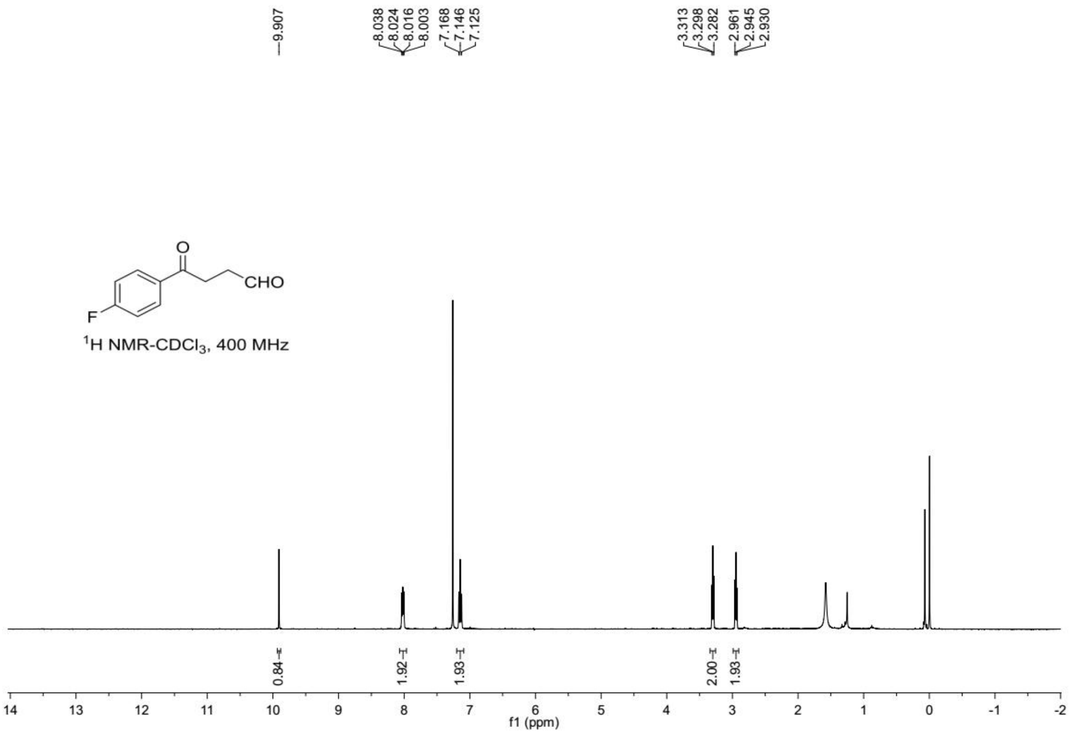

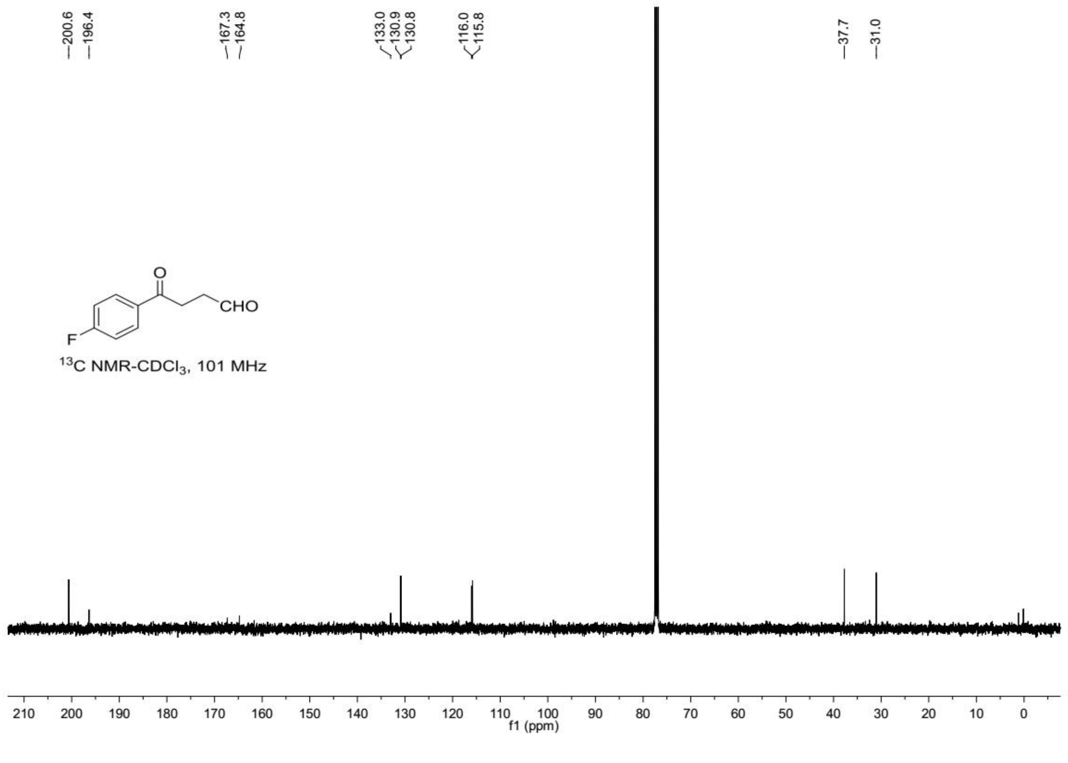

Supplementary Fig. 67. NMR of compound **12b** in $CDCl_3$

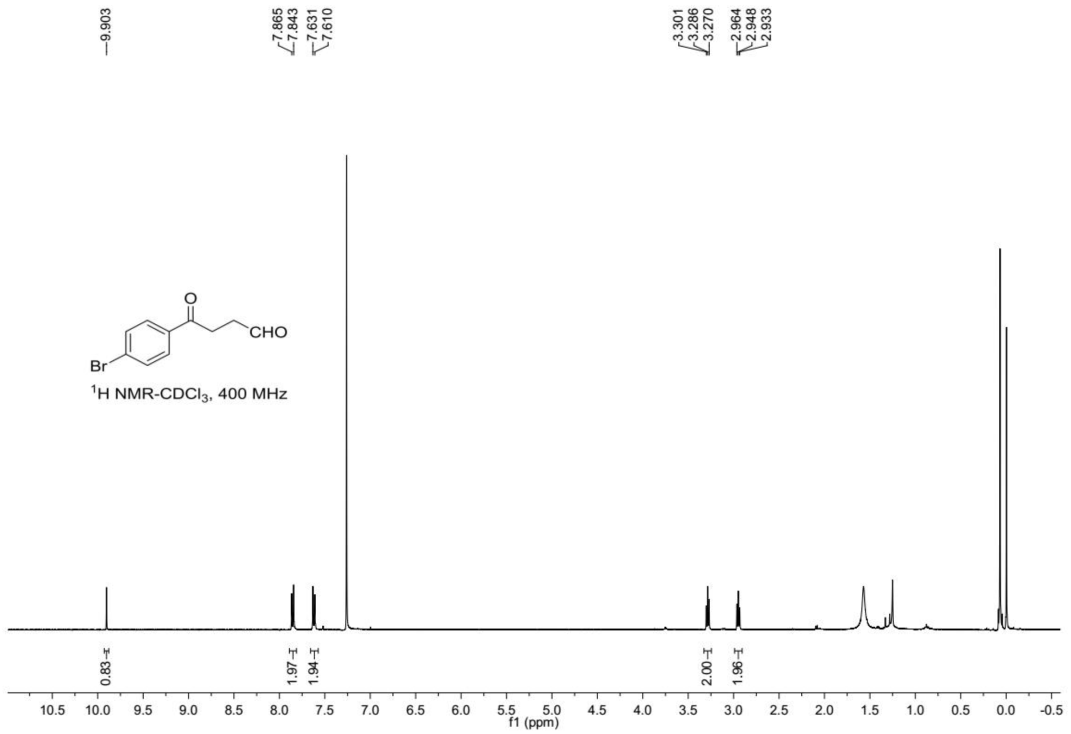

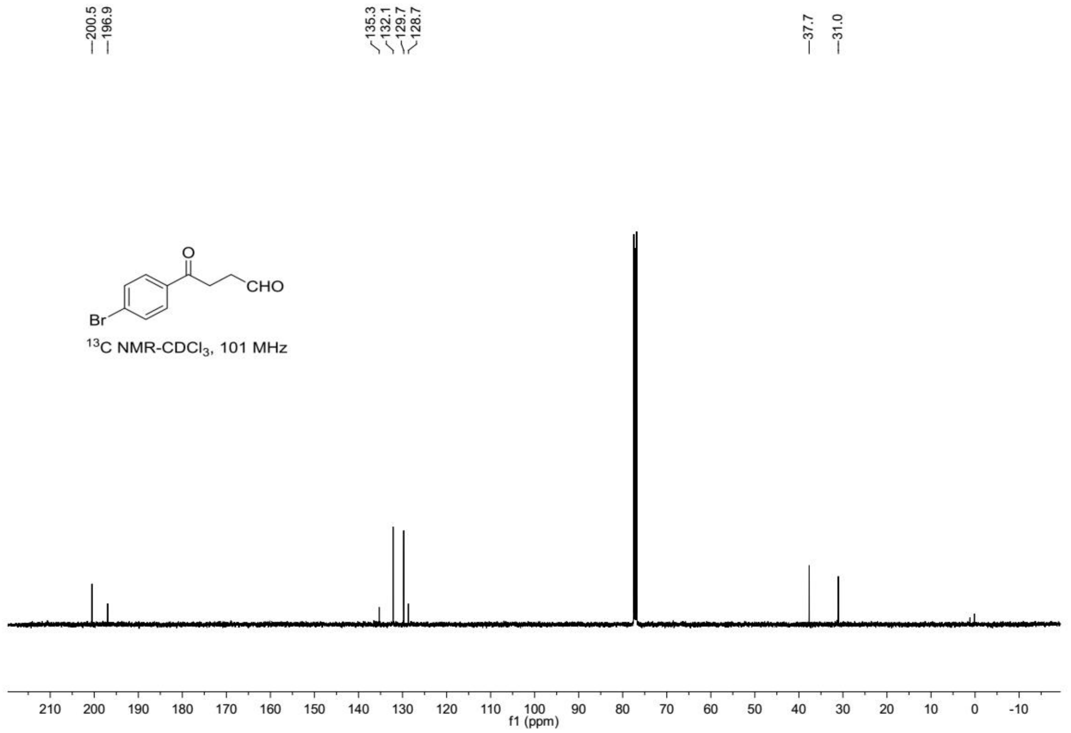

Supplementary Fig. 68. NMR of compound **13b** in $CDCl_3$

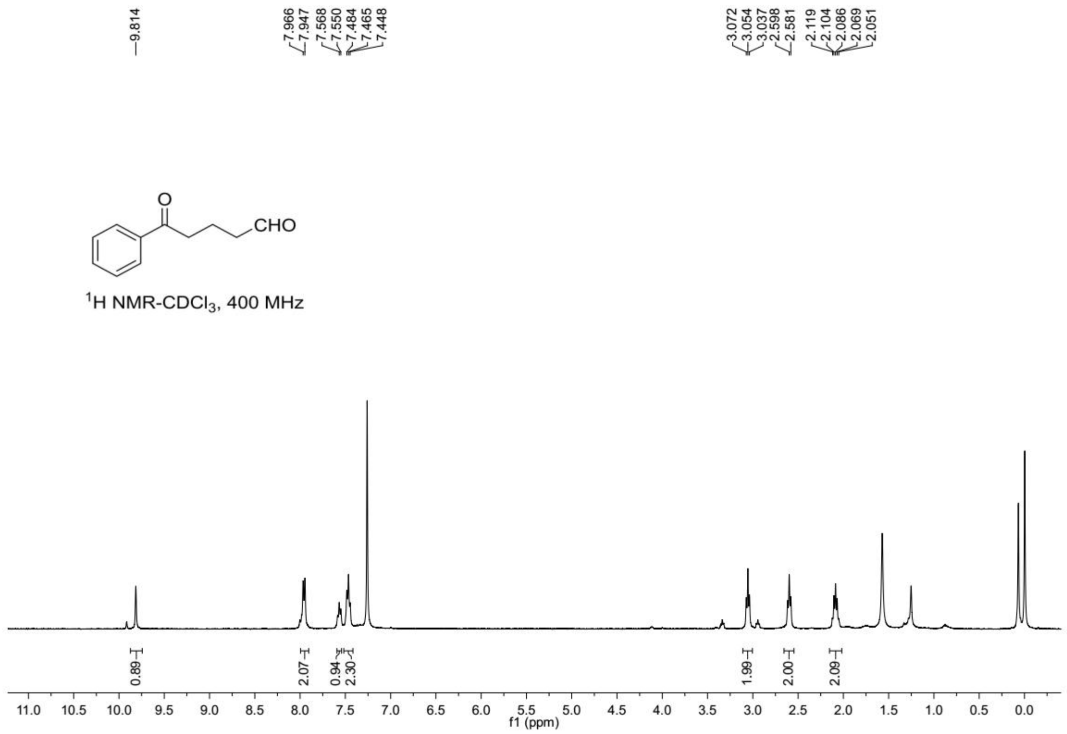

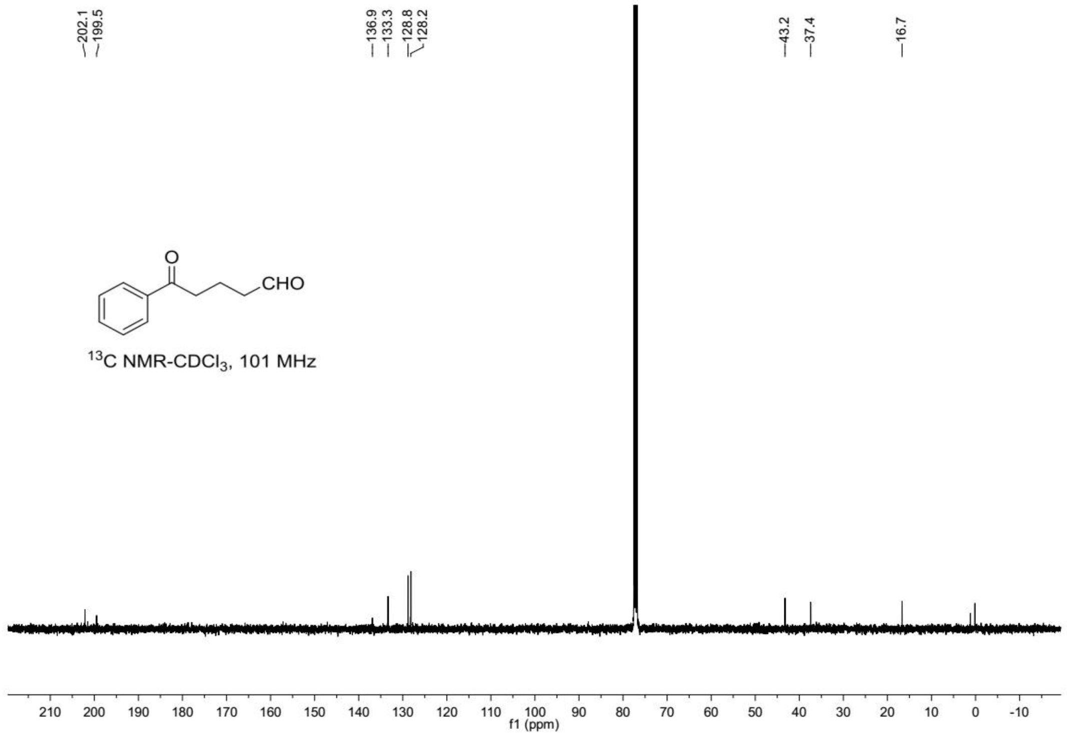

Supplementary Fig. 69. NMR of compound **14b** in CDCl$_3$

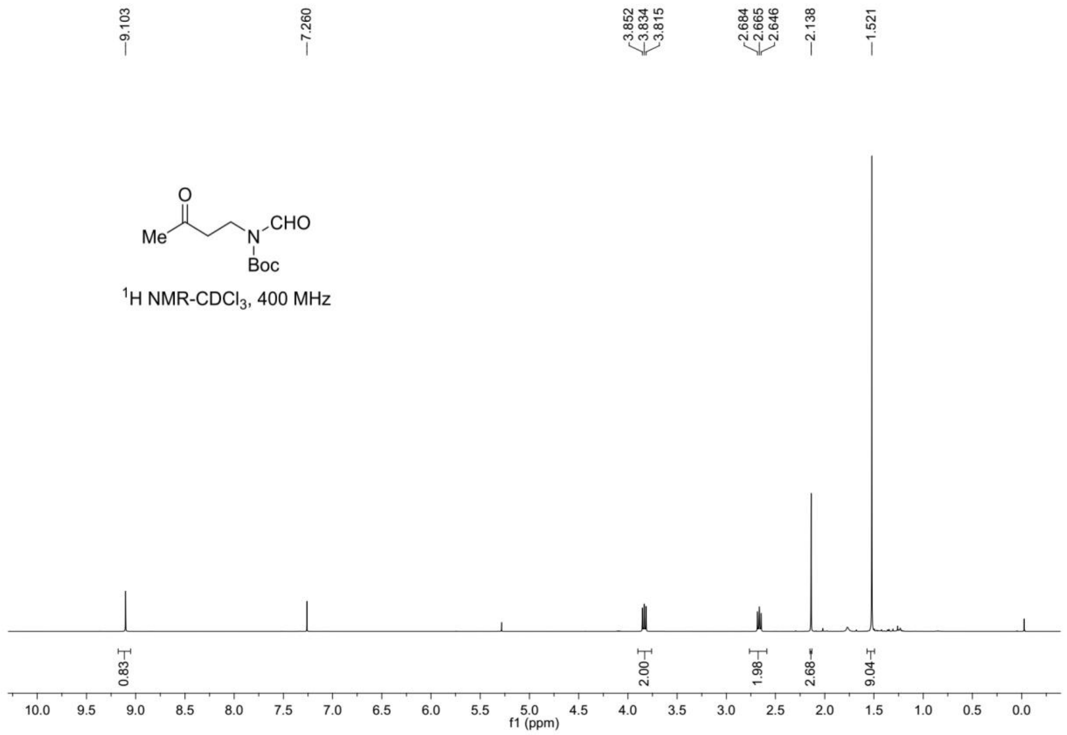

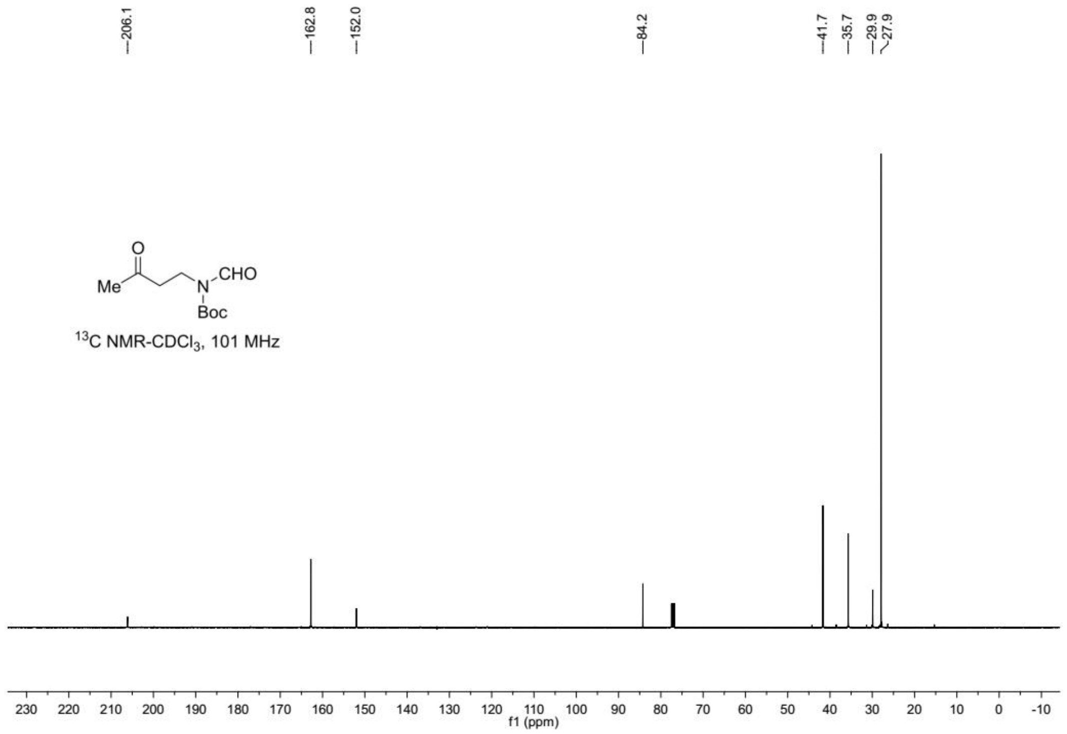

Supplementary Fig. 70. NMR of compound **15b**, **21b** in $CDCl_3$

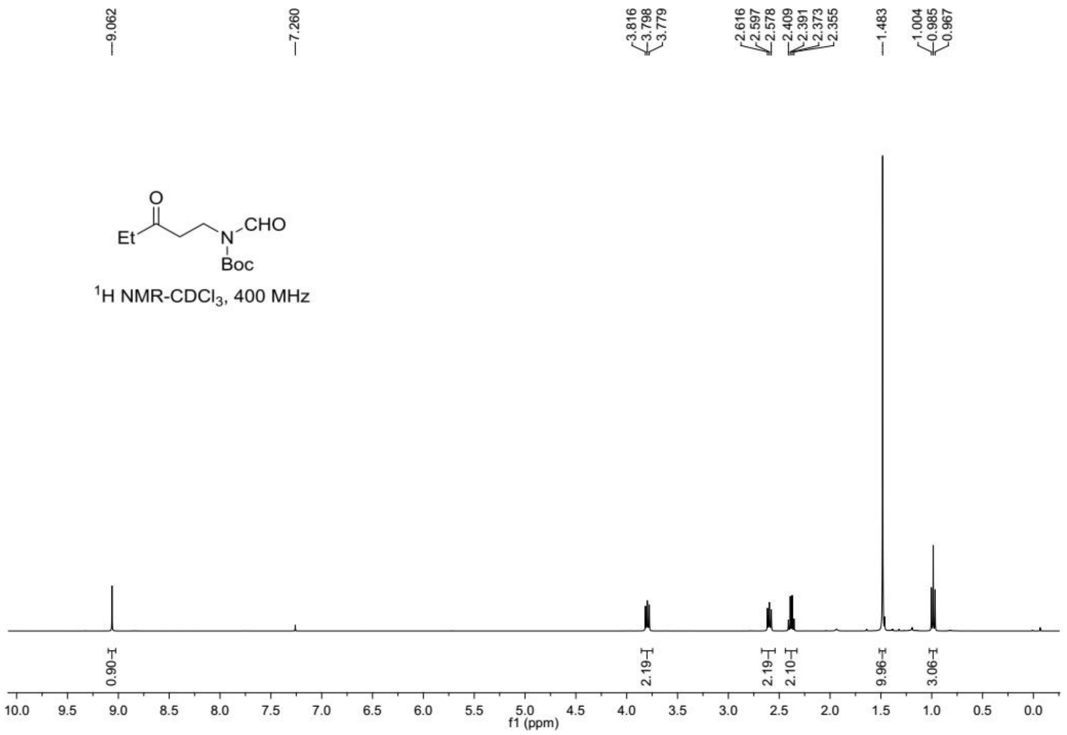

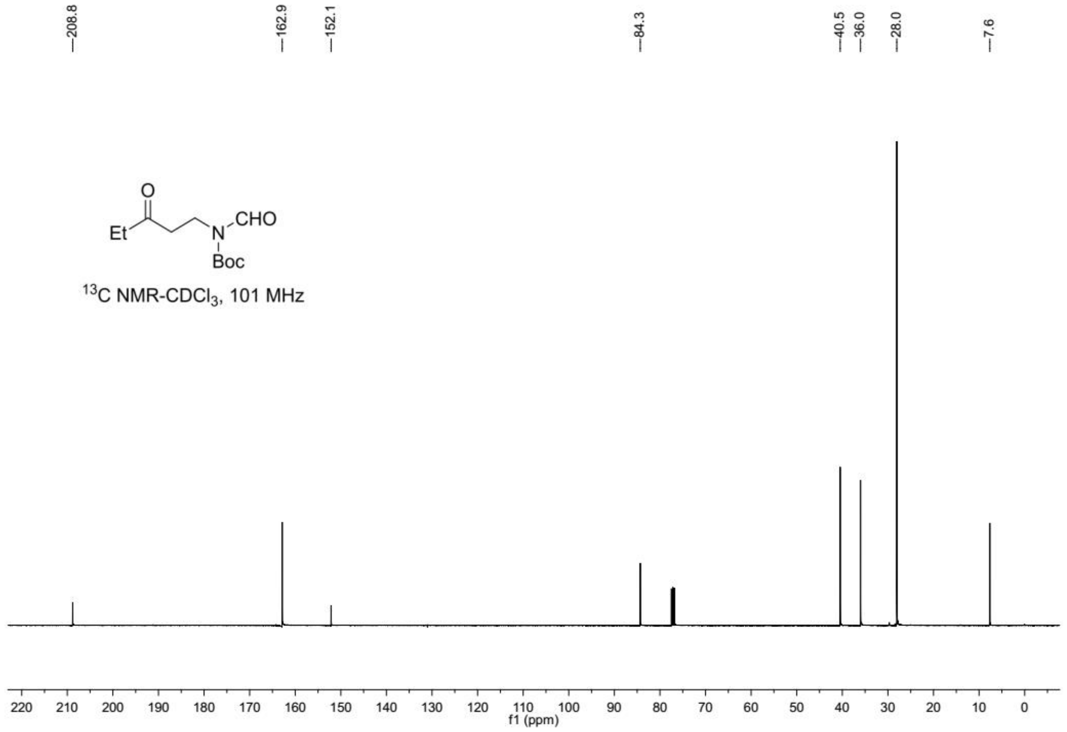

Supplementary Fig. 71. NMR of compound **16b** in $CDCl_3$

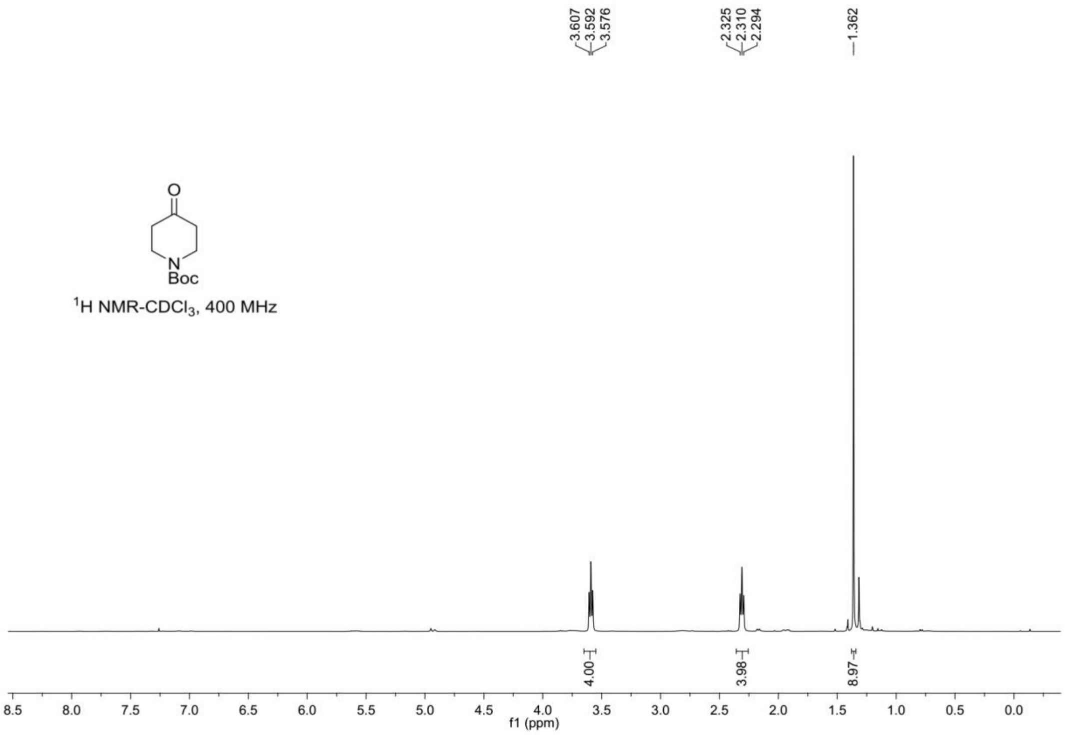

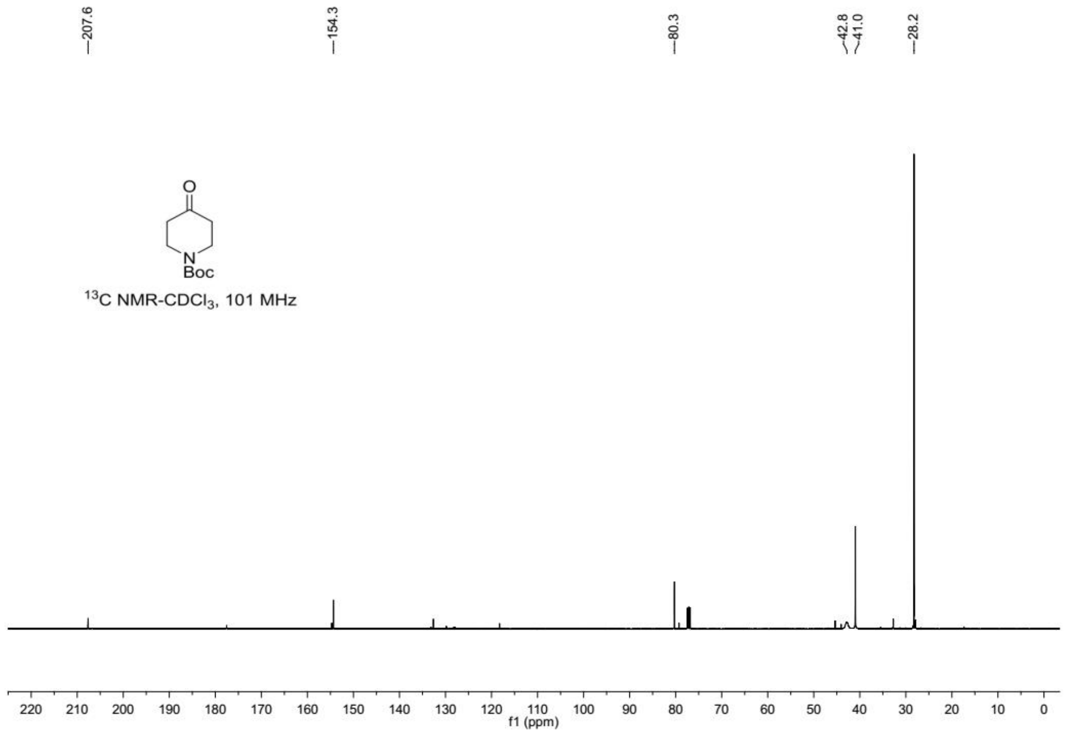

Supplementary Fig. 72. NMR of compound **10c**, **17b**, **18b**, **19b** in $CDCl_3$

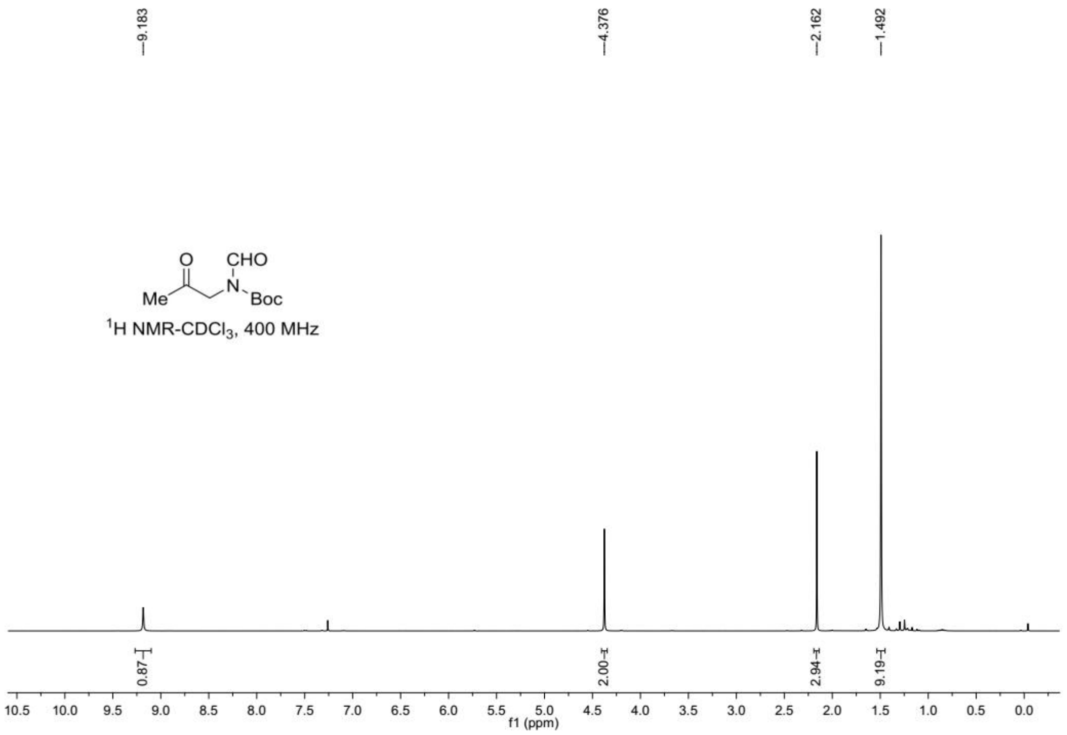

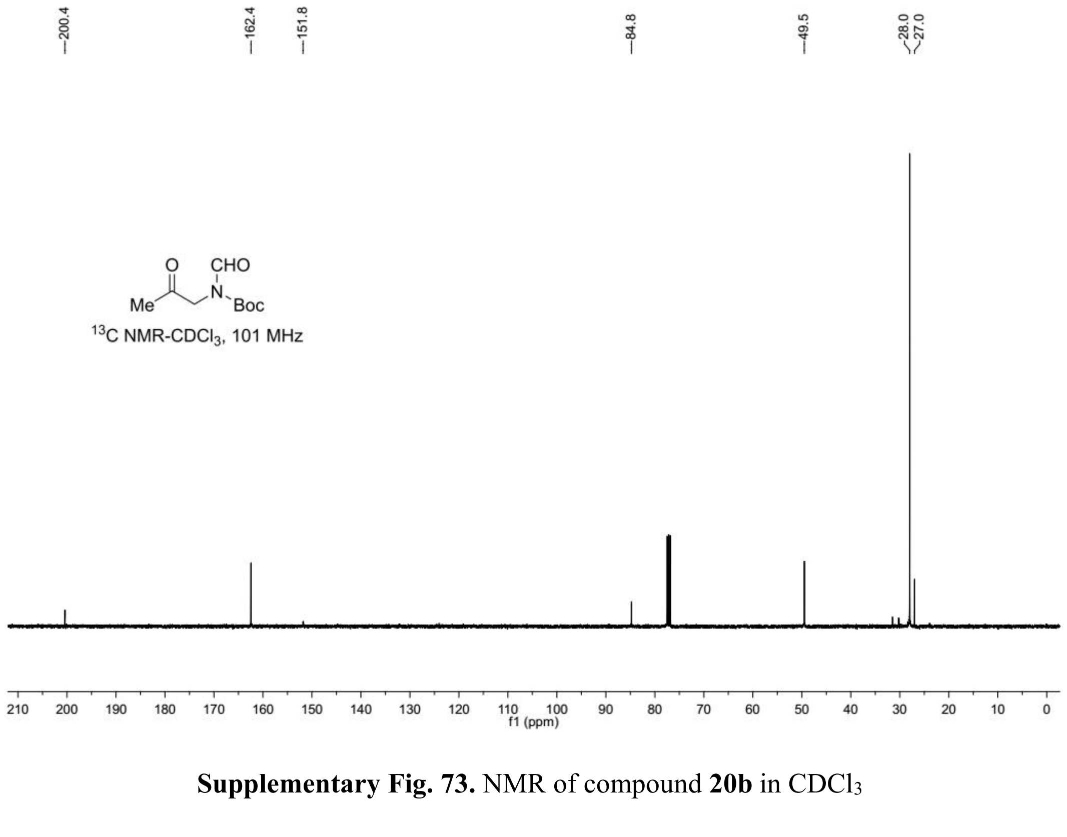

Supplementary Fig. 73. NMR of compound **20b** in $CDCl_3$

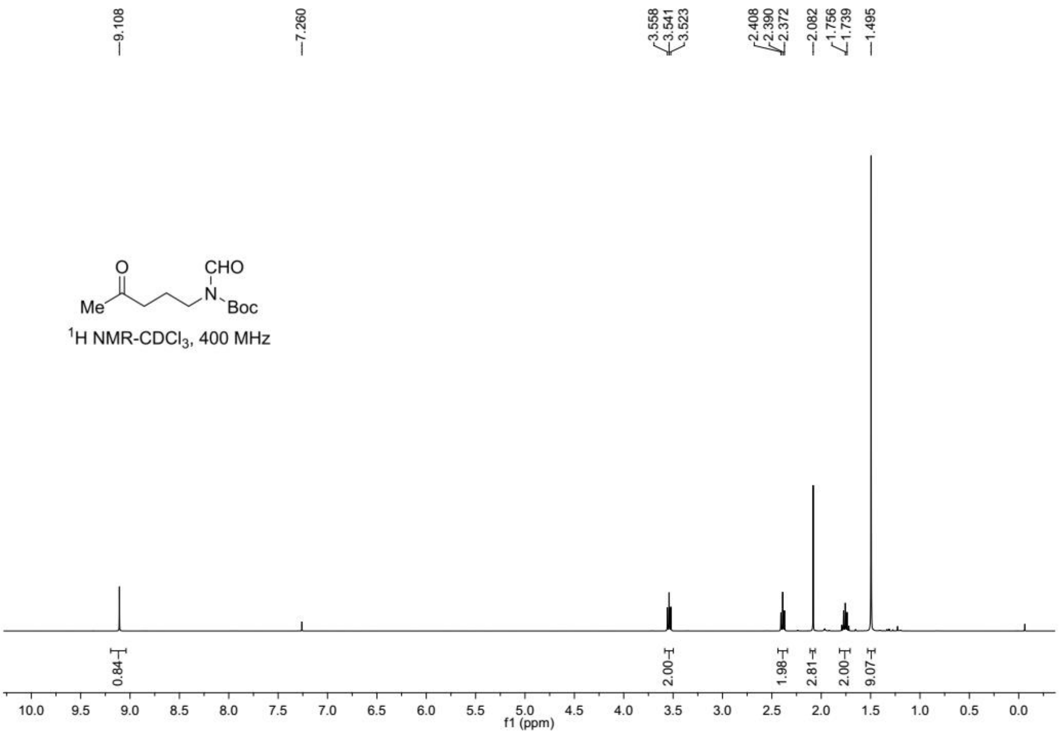

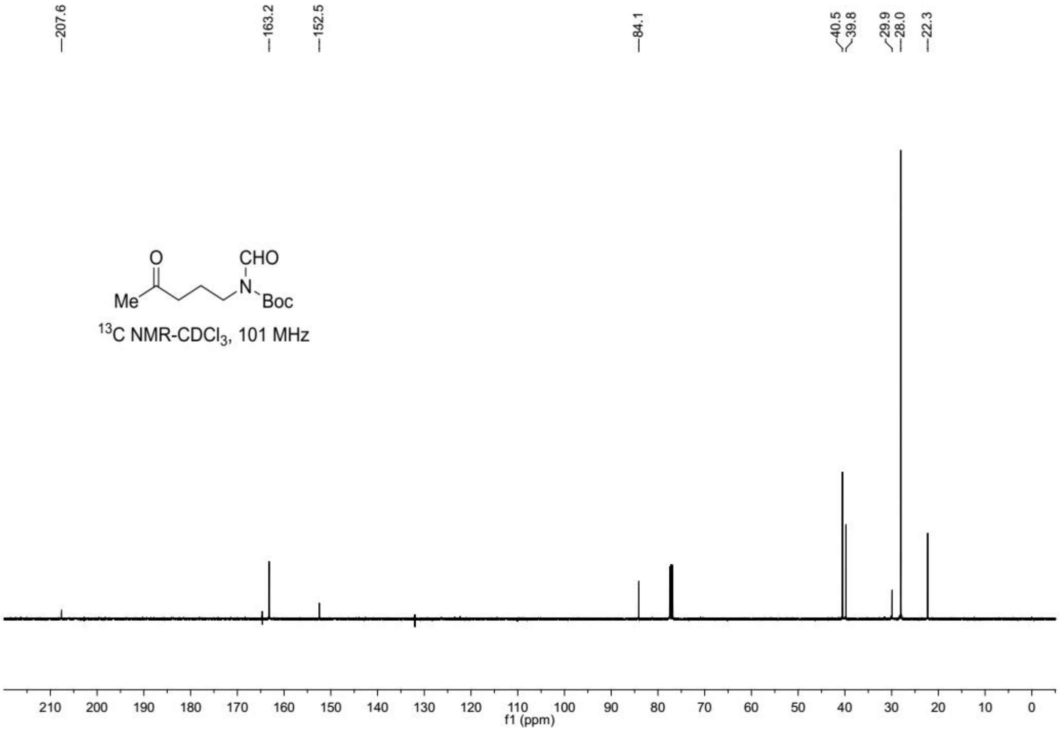

Supplementary Fig. 74. NMR of compound **22b** in $CDCl_3$

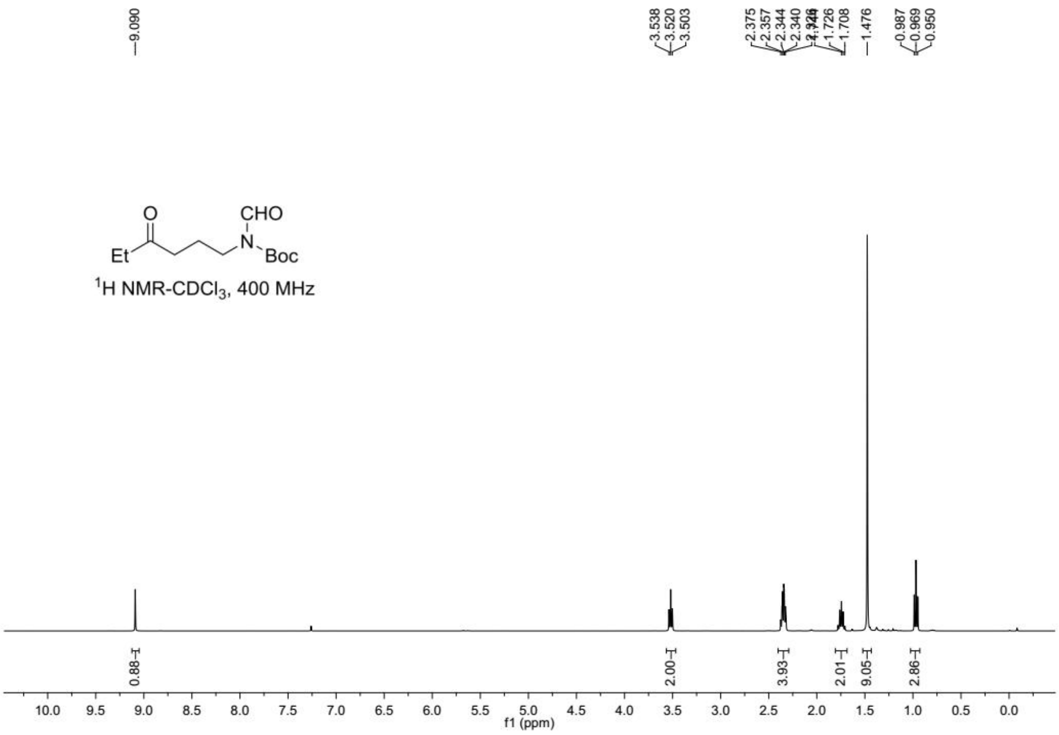

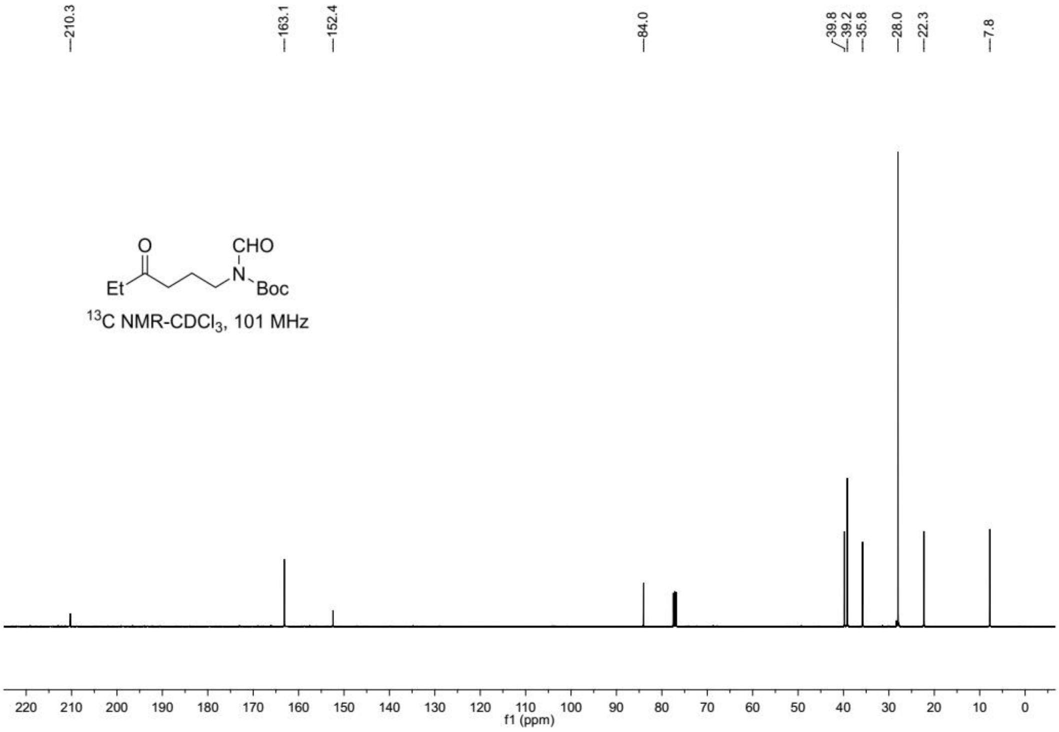

Supplementary Fig. 75. NMR of compound **23b** in $CDCl_3$

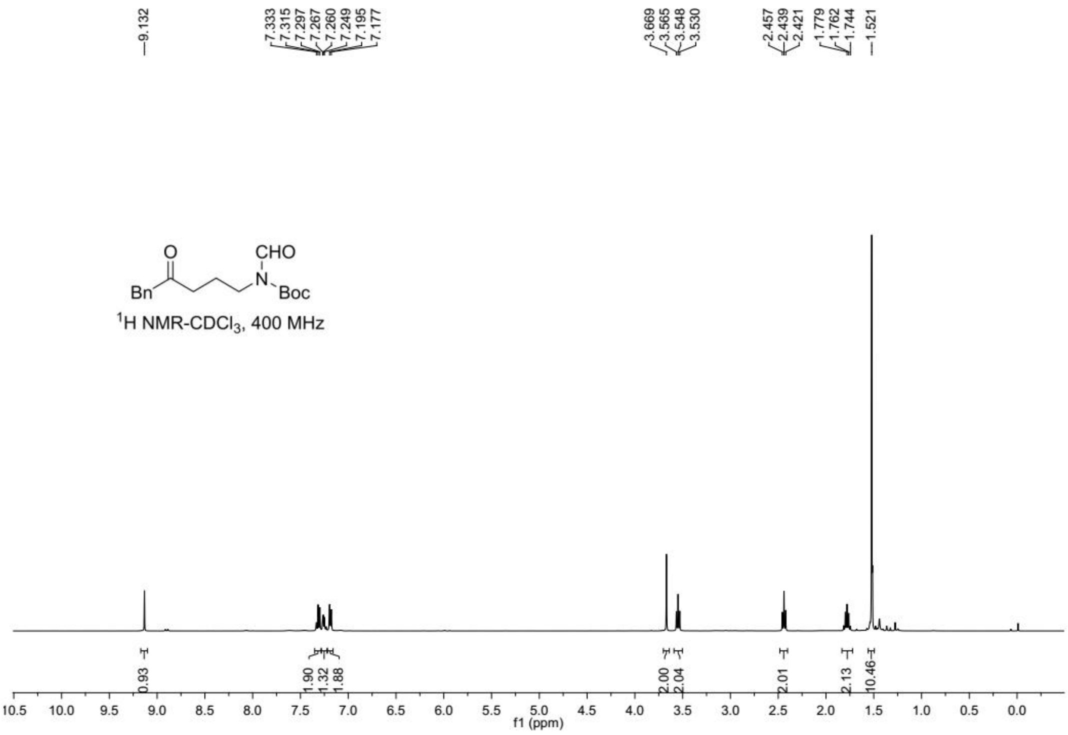

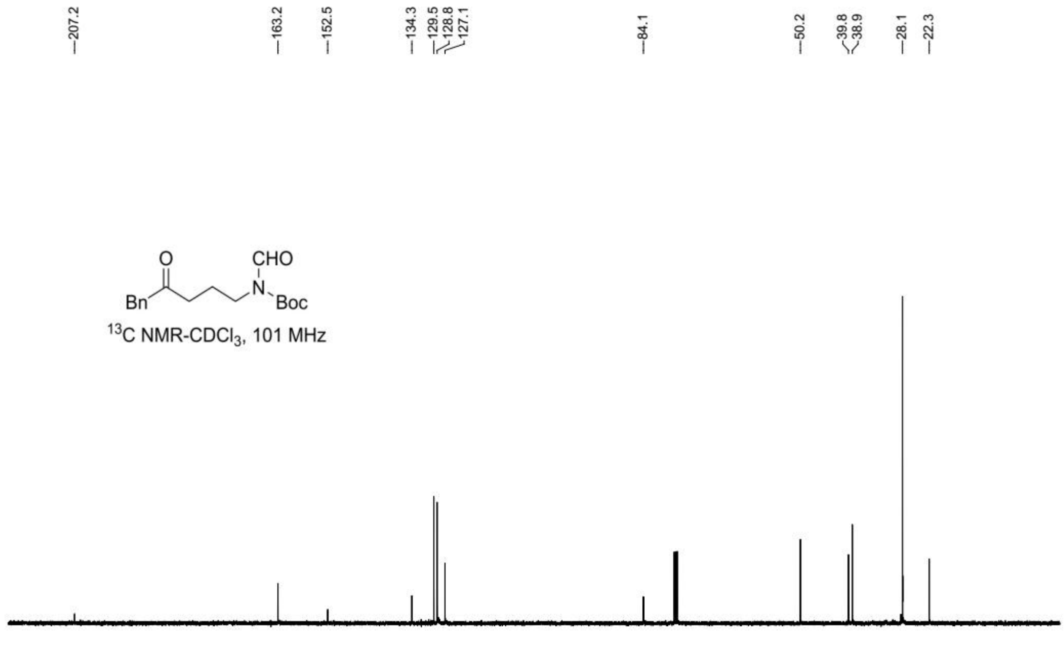

Supplementary Fig. 76. NMR of compound **24b** in $CDCl_3$

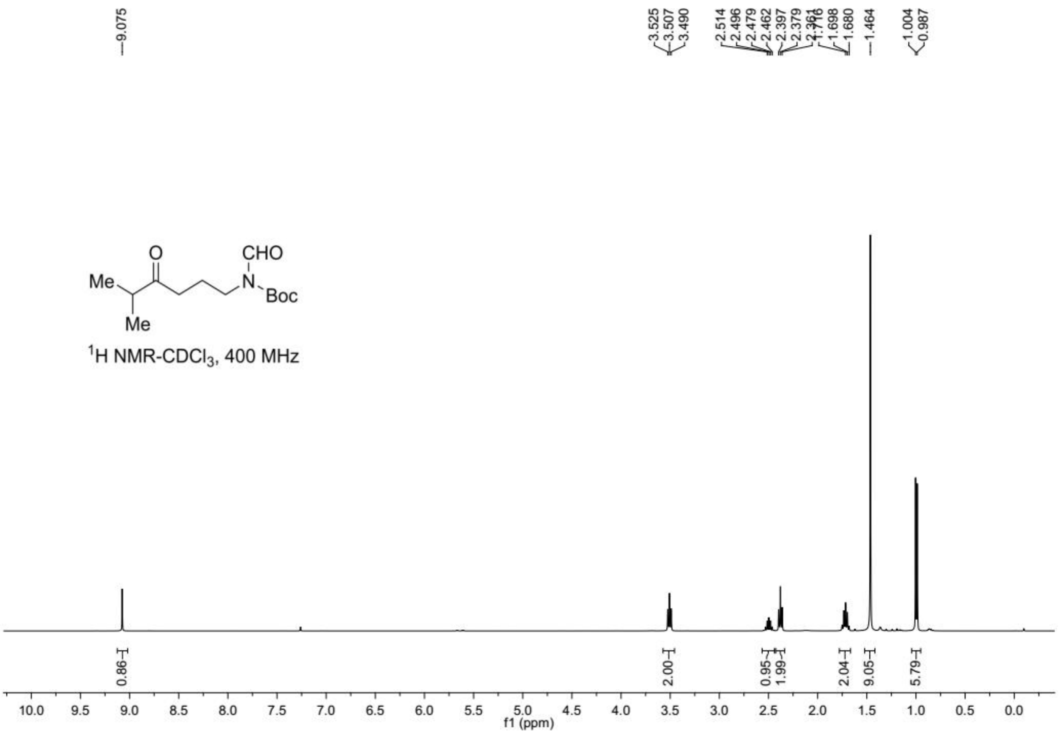

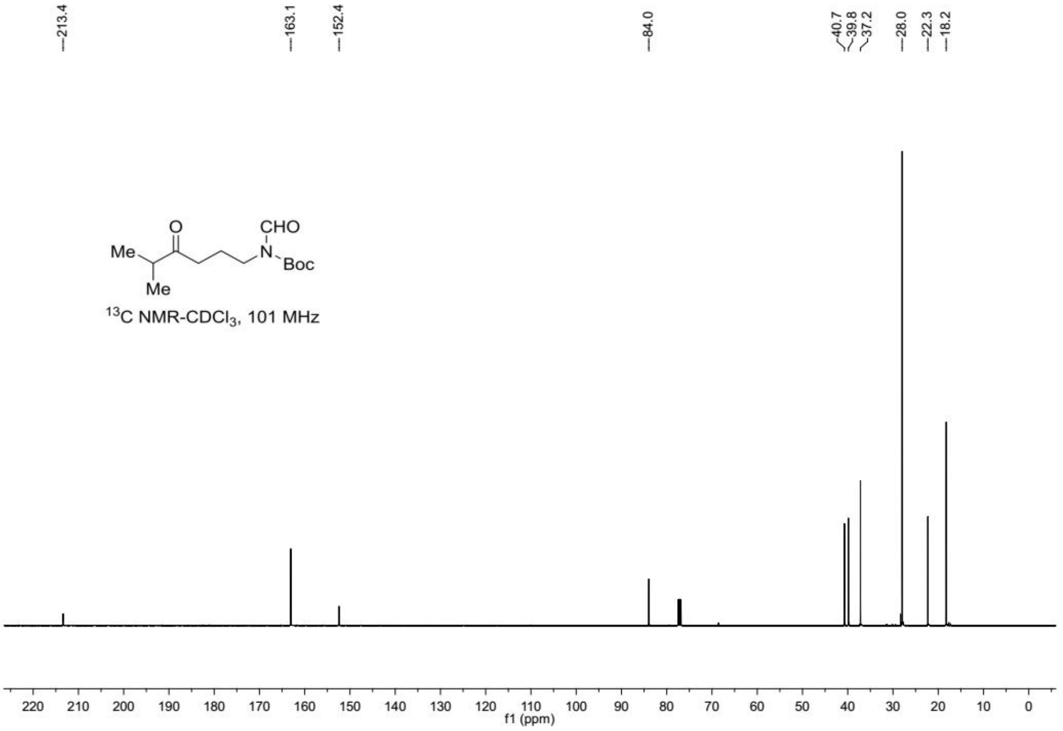

Supplementary Fig. 77. NMR of compound **25b** in $CDCl_3$

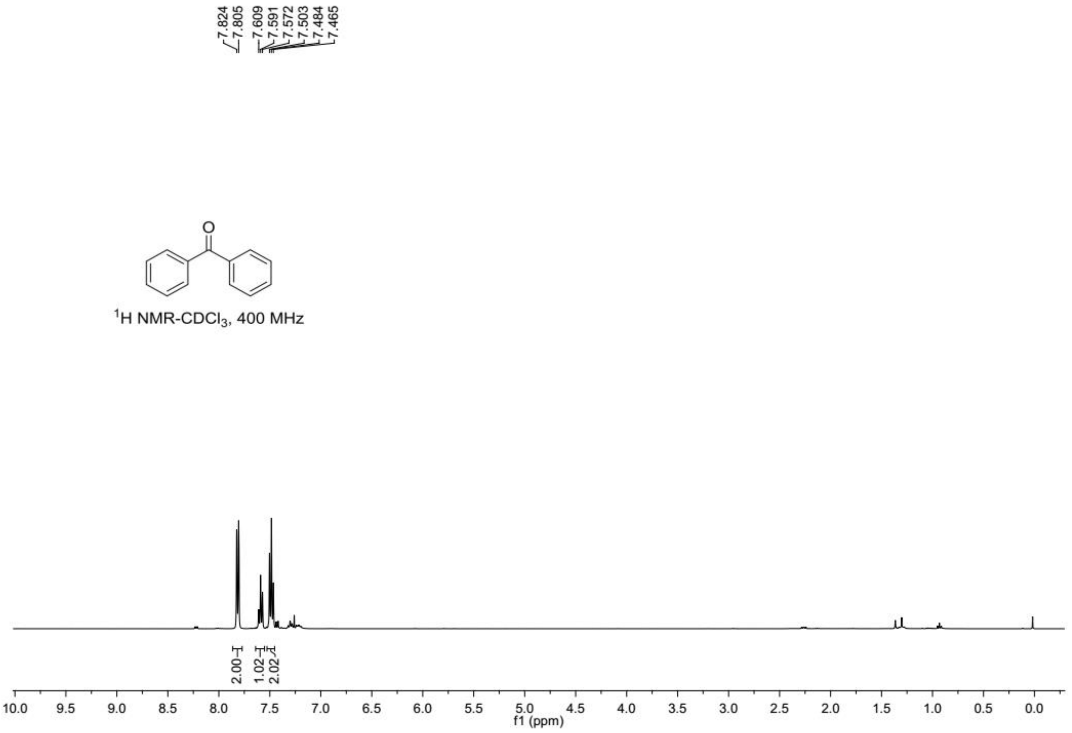

Supplementary Fig. 78. NMR of compound **26b**, **27b** in $CDCl_3$

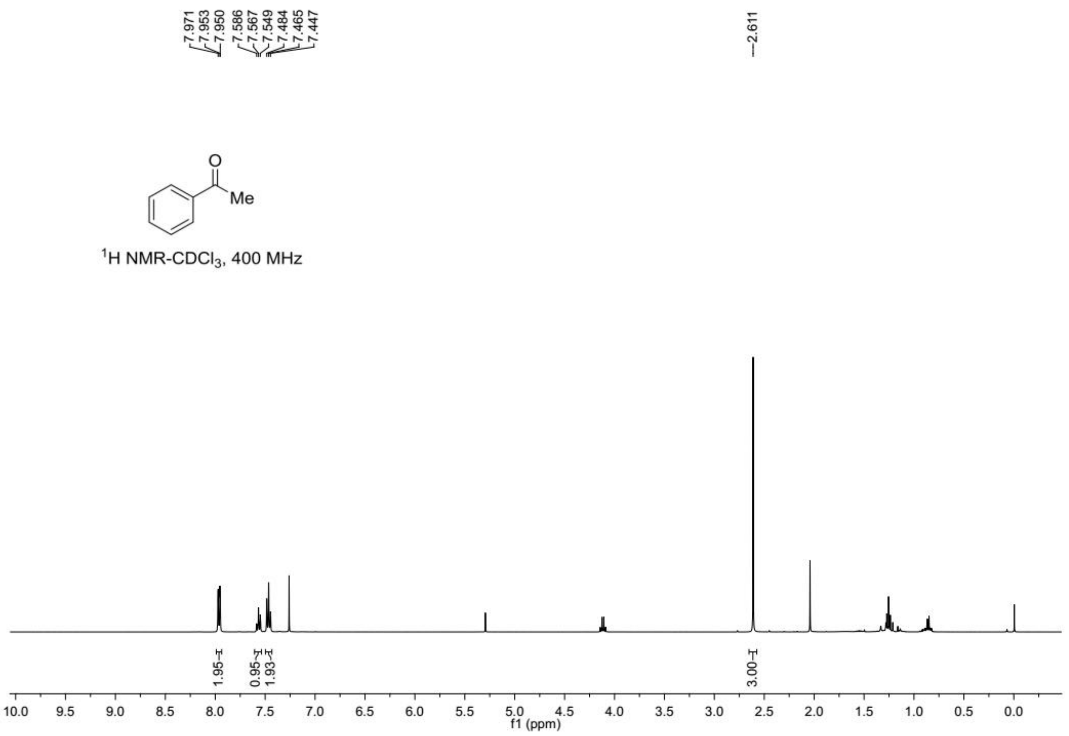

Supplementary Fig. 79. NMR of compound **28b**, **29b**, **37b** in $CDCl_3$

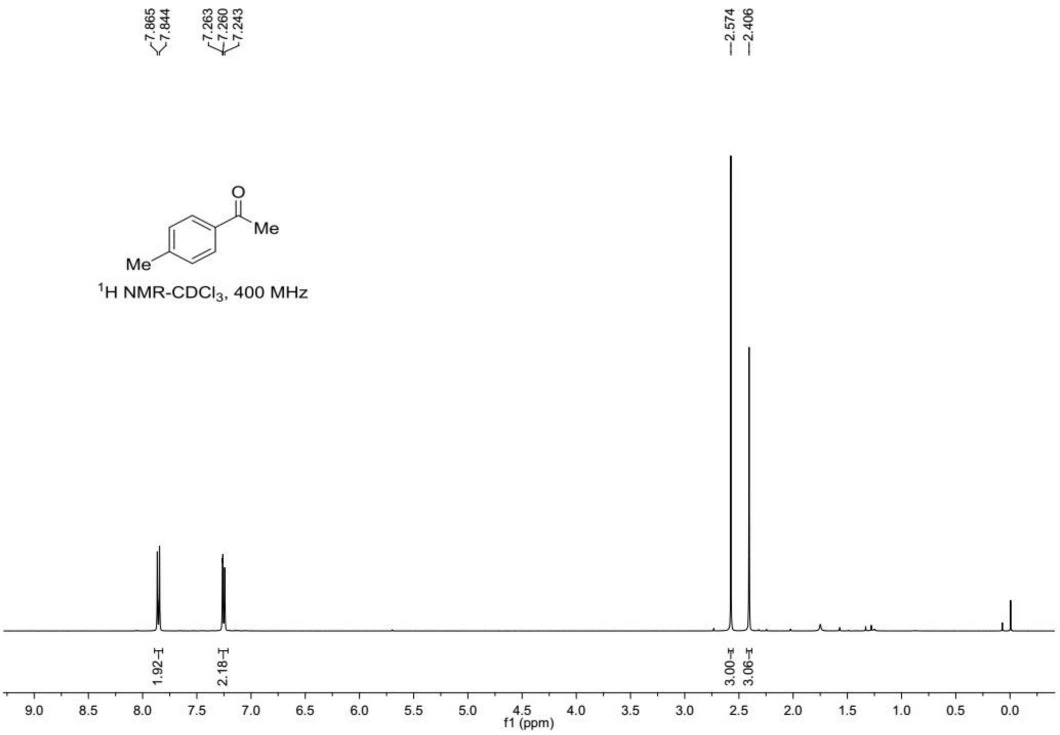

Supplementary Fig. 80. NMR of compound **30b** in $CDCl_3$

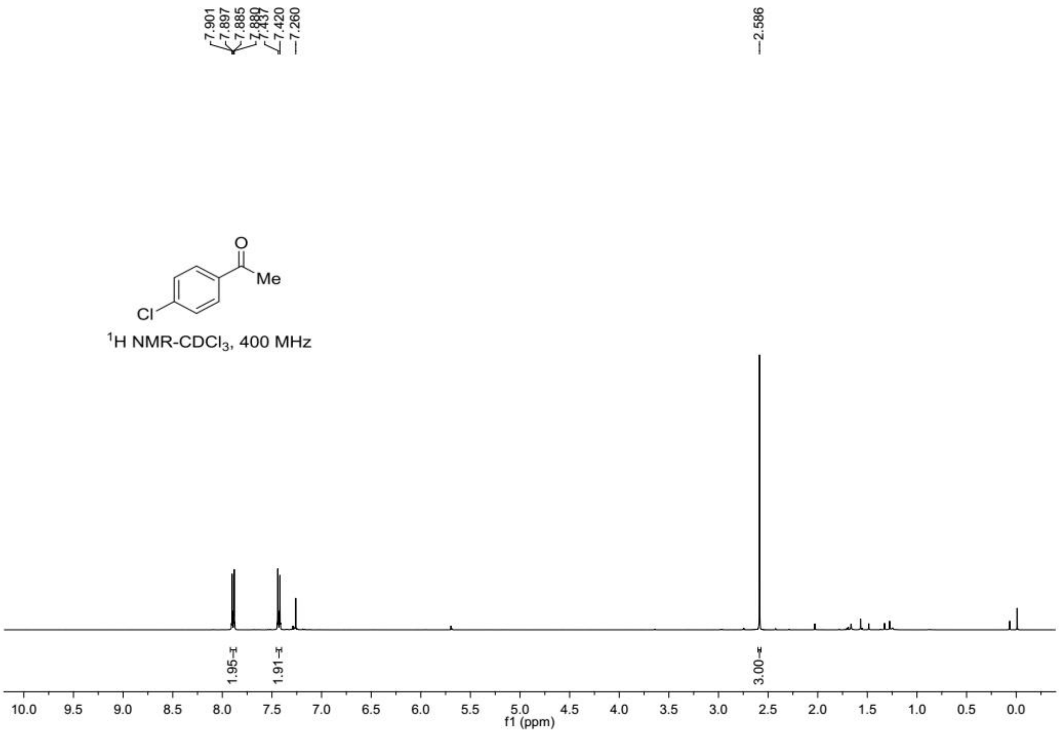

Supplementary Fig. 81. NMR of compound **31b** in $CDCl_3$

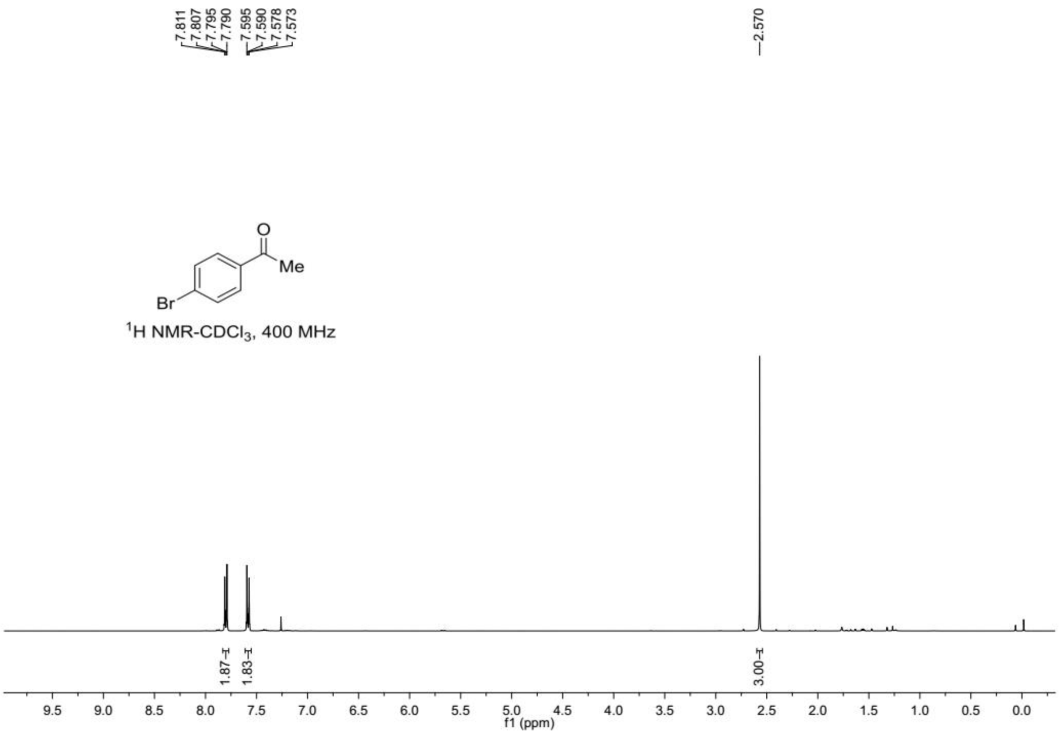

Supplementary Fig. 82. NMR of compound **32b** in $CDCl_3$

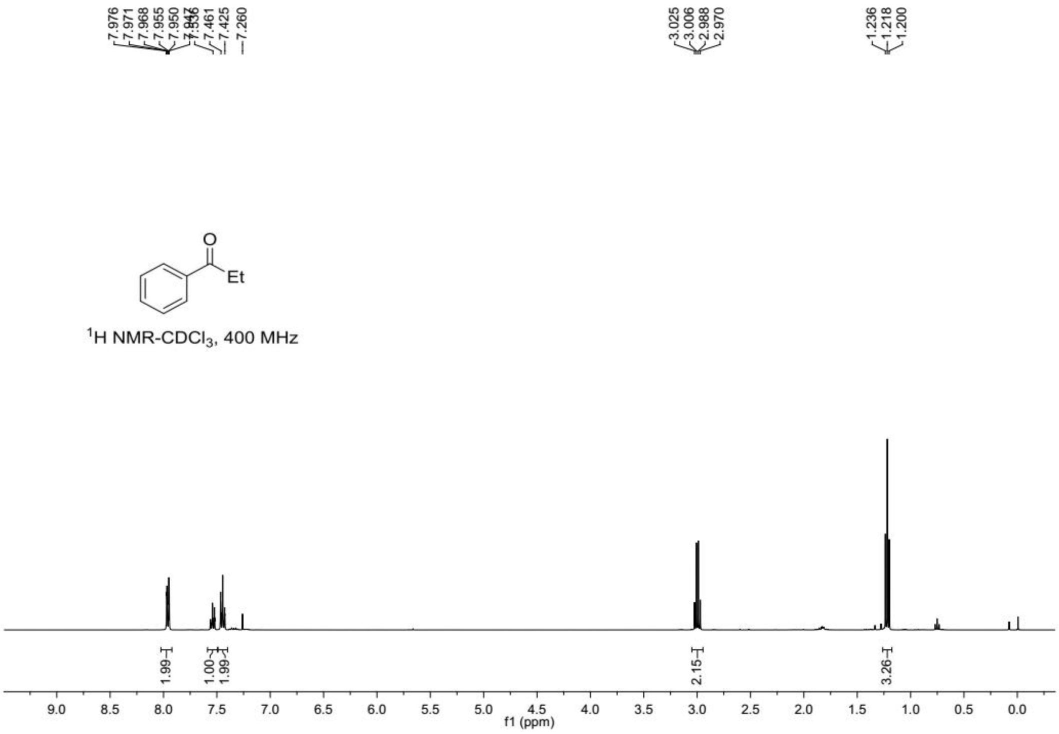

Supplementary Fig. 83. NMR of compound **33b** in $CDCl_3$

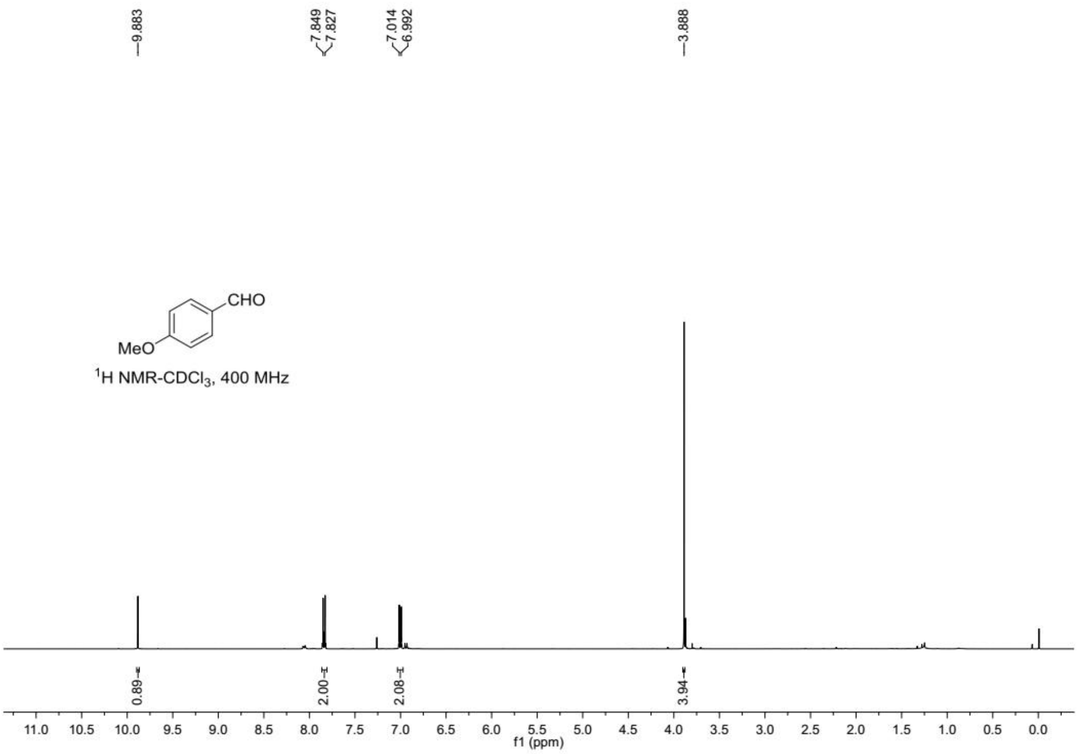

Supplementary Fig. 84. NMR of compound **34b** in $CDCl_3$

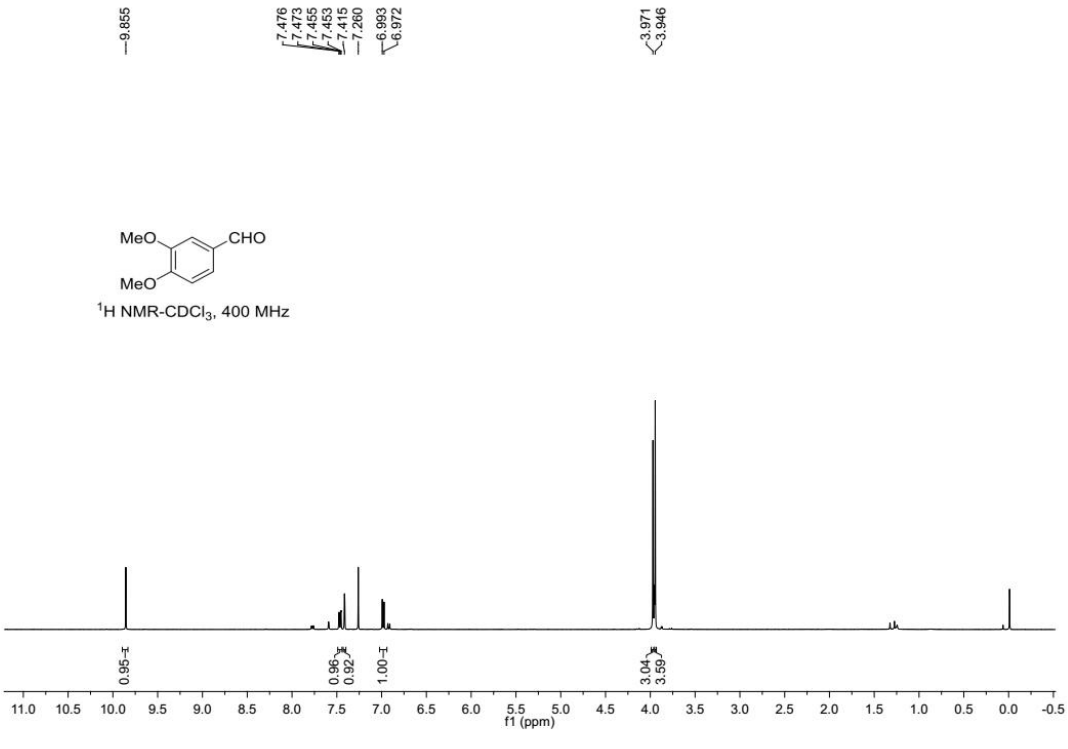

Supplementary Fig. 85. NMR of compound **35b** in $CDCl_3$

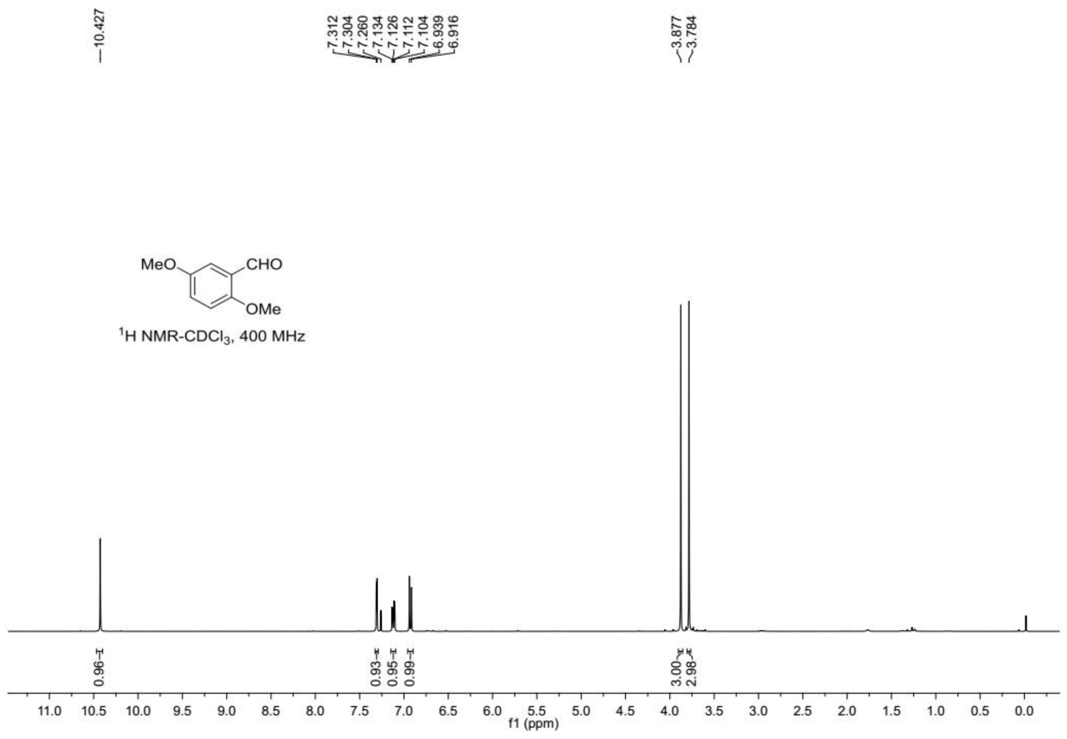

Supplementary Fig. 86. NMR of compound **36b** in $CDCl_3$

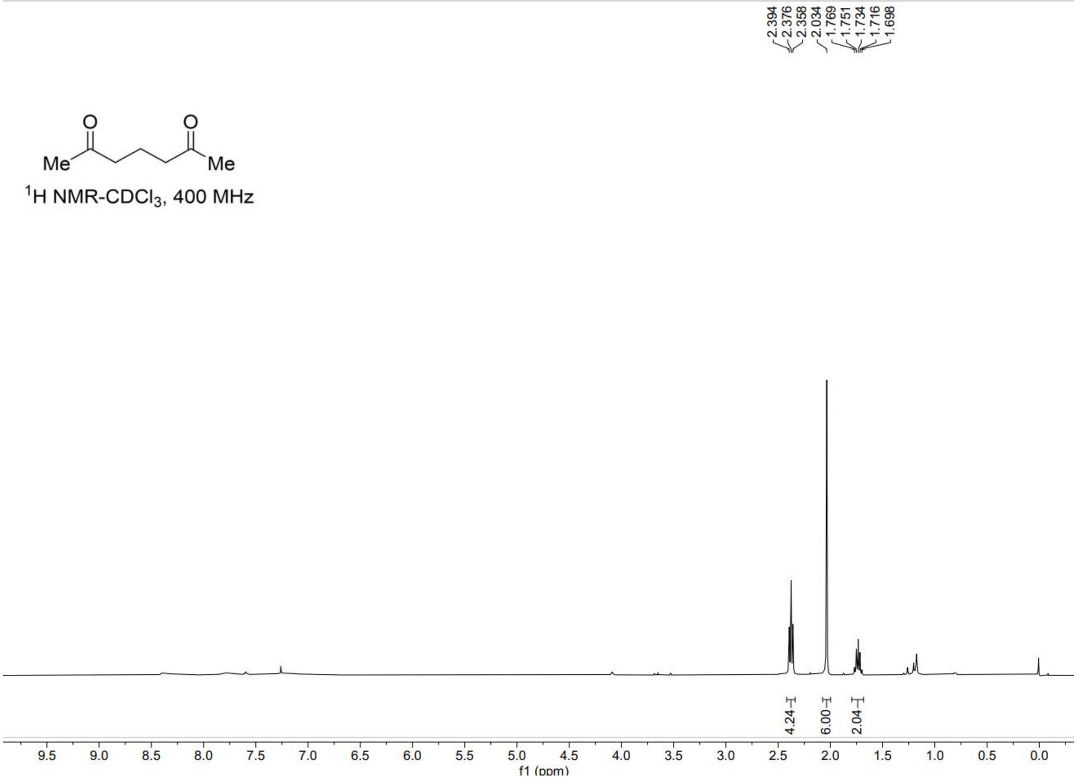

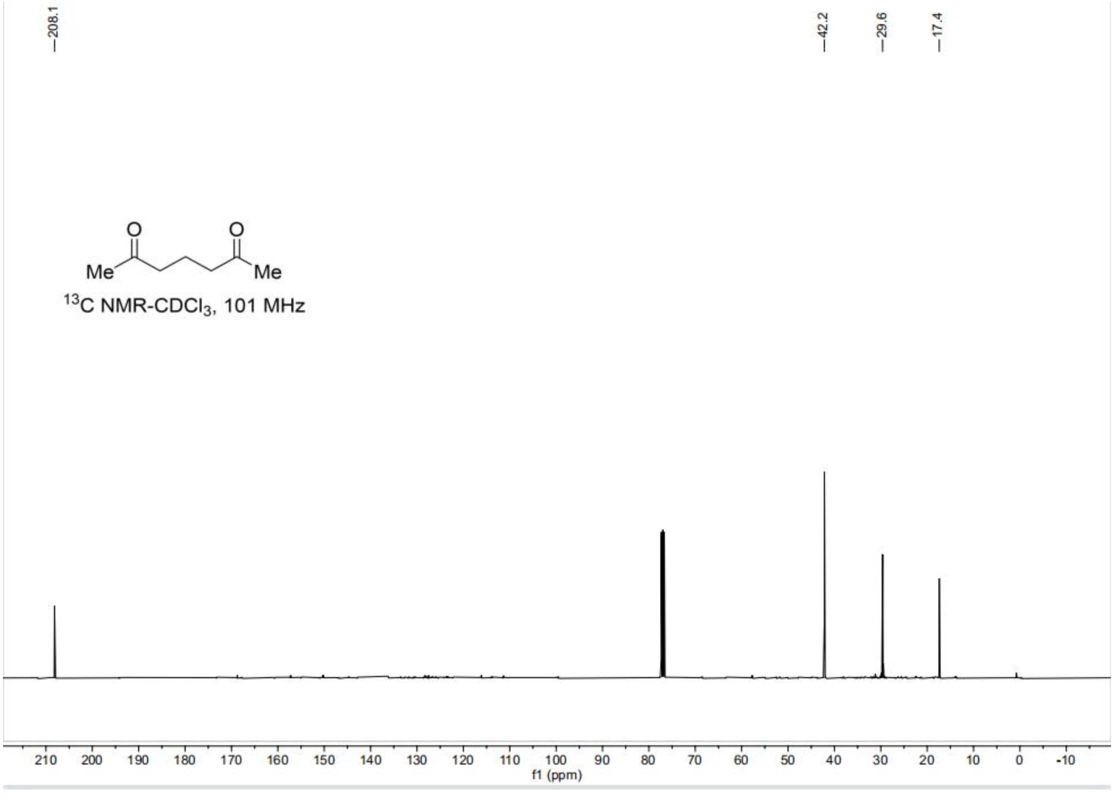

Supplementary Fig. 87. NMR of compound **38b** in $CDCl_3$

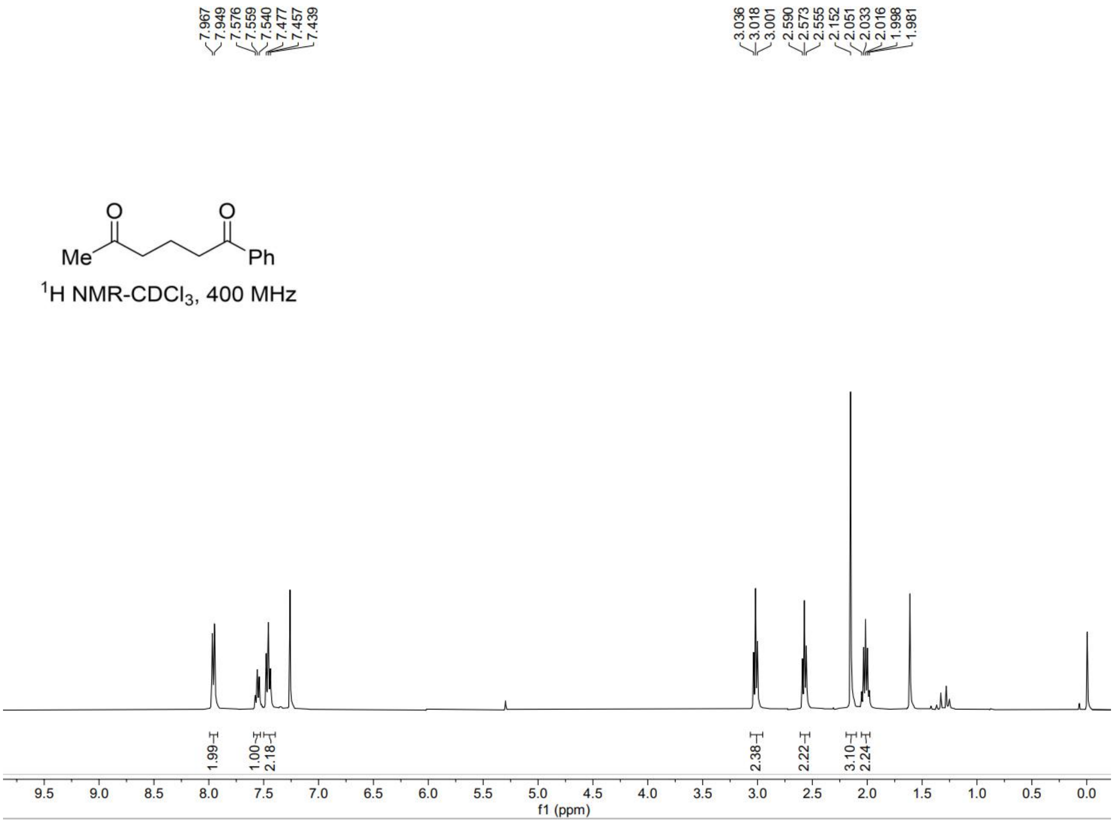

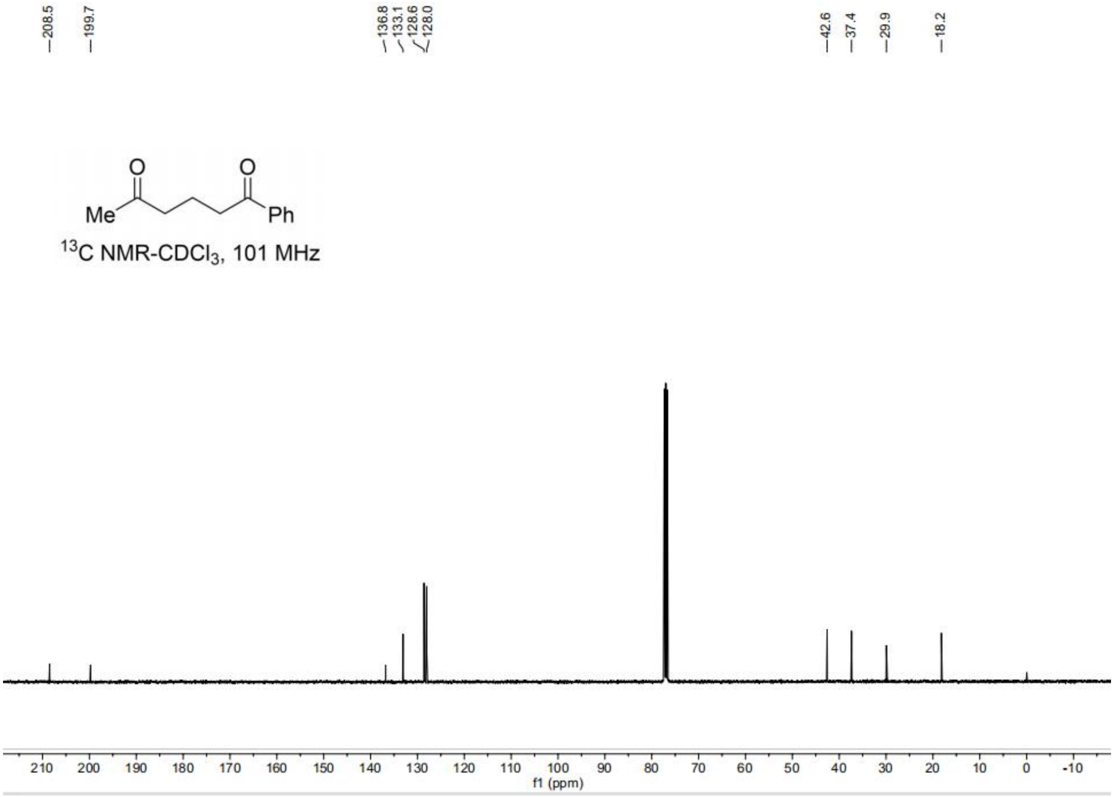

Supplementary Fig. 88. NMR of compound **39b** in $CDCl_3$

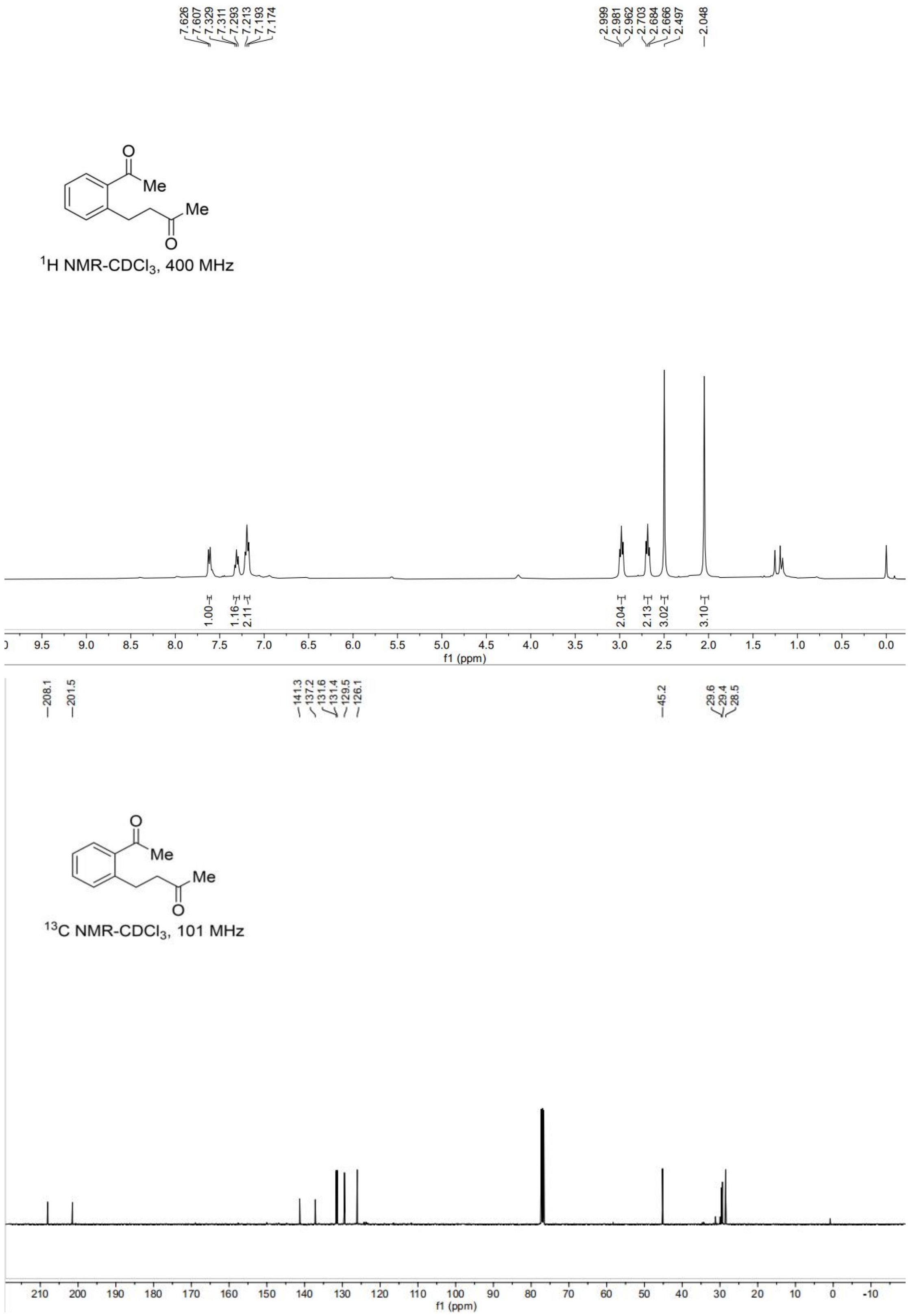

Supplementary Fig. 89. NMR of compound **40b** in $CDCl_3$

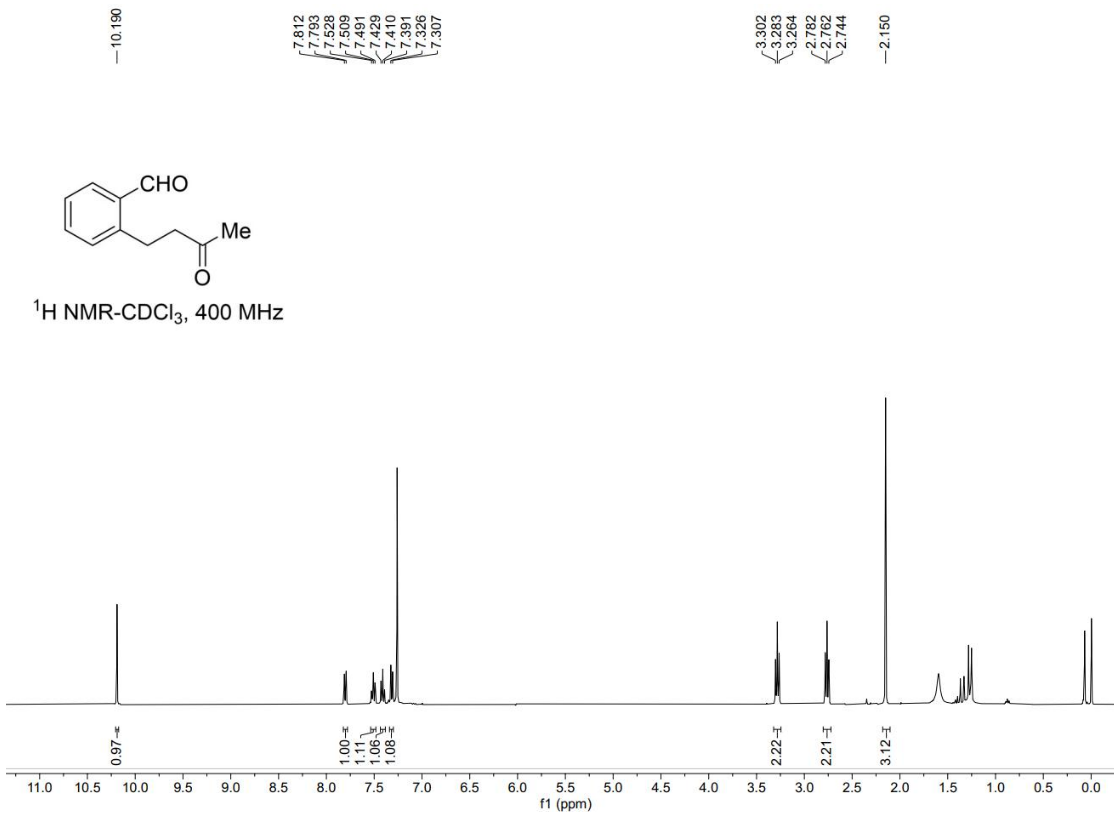

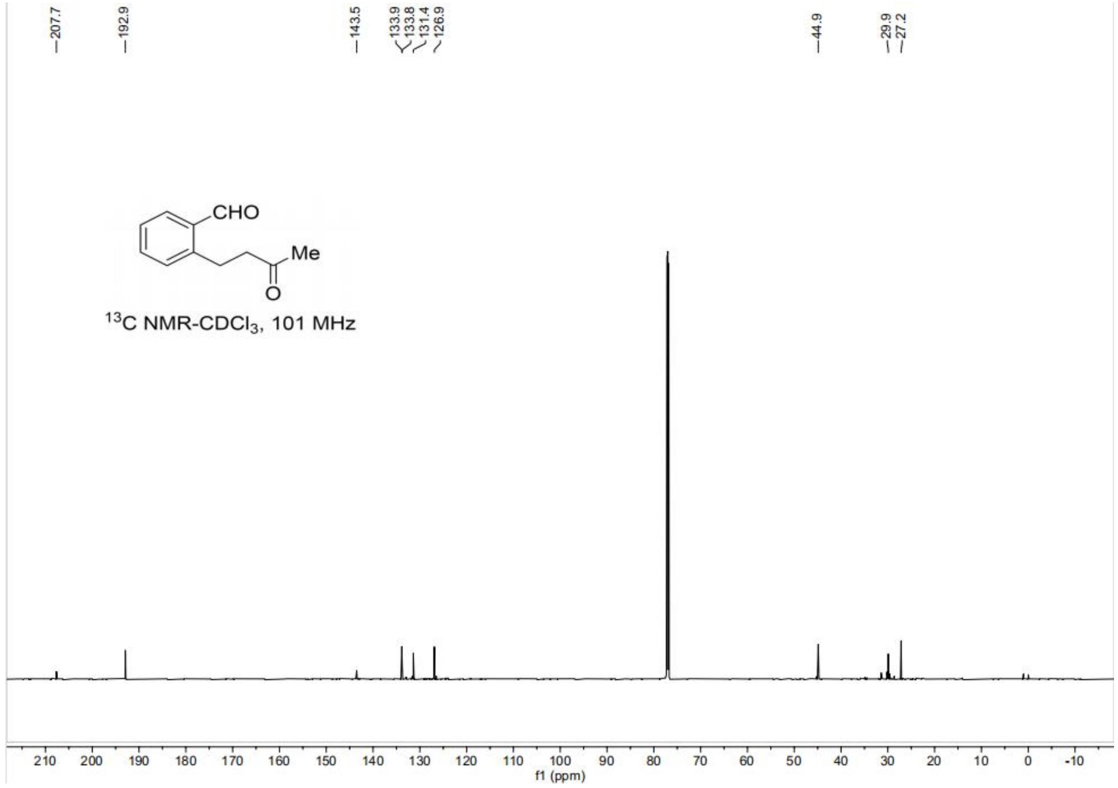

Supplementary Fig. 90. NMR of compound **41b** in $CDCl_3$

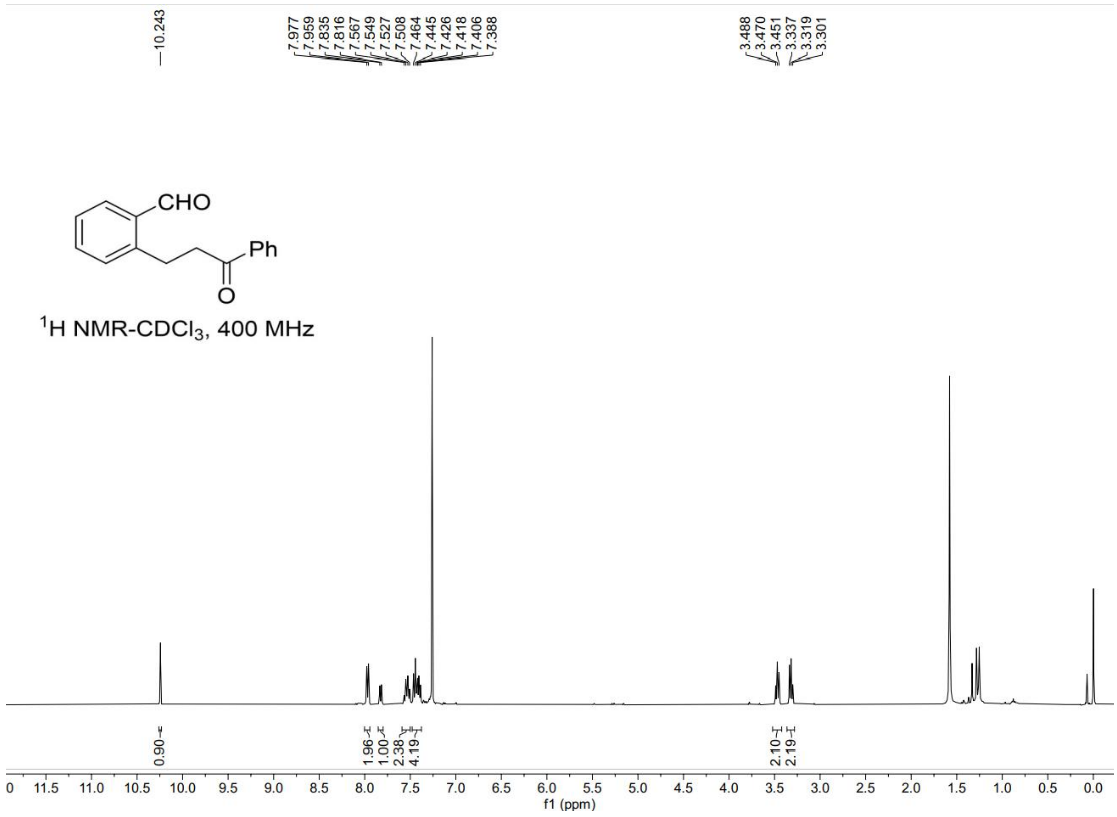

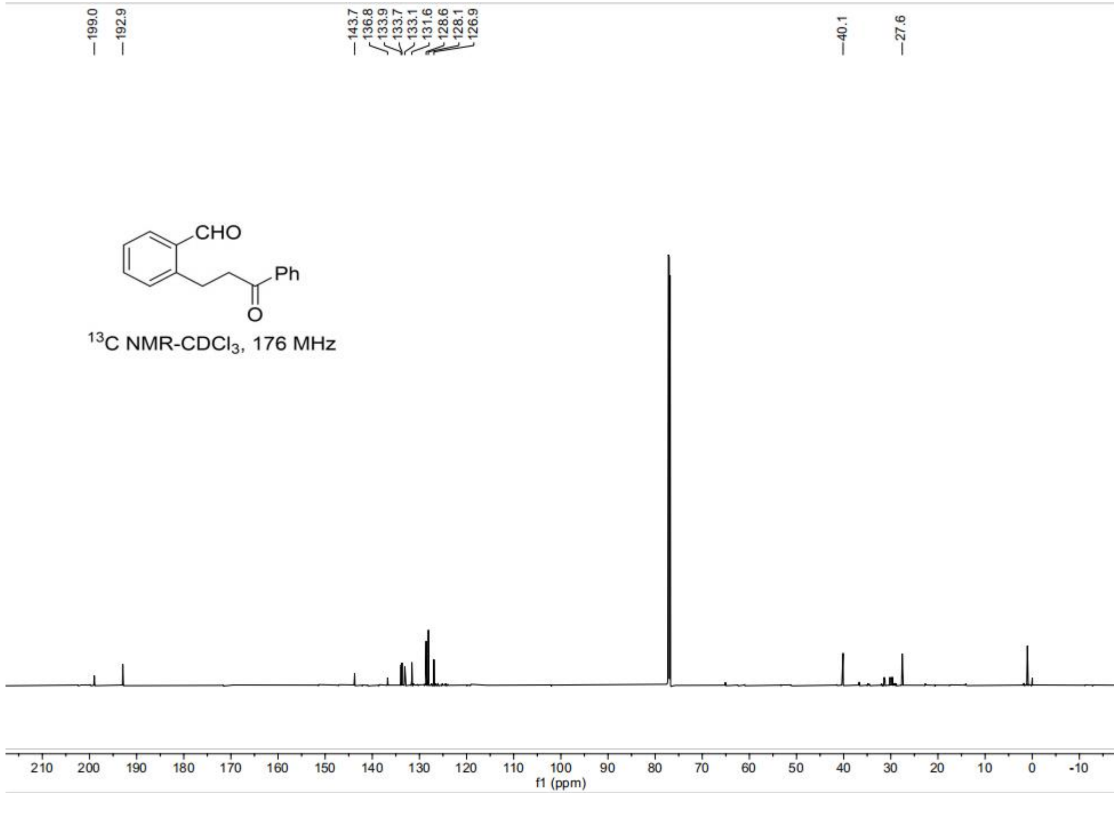

Supplementary Fig. 91. NMR of compound **42b** in $CDCl_3$

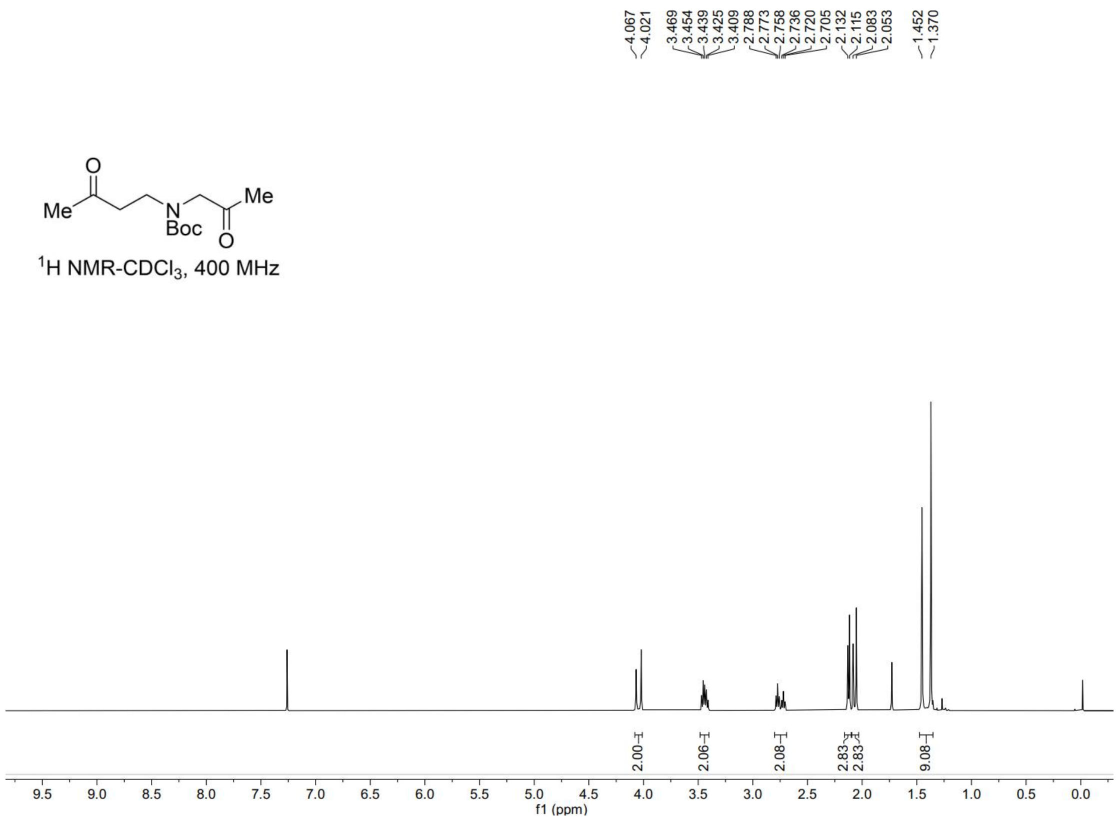

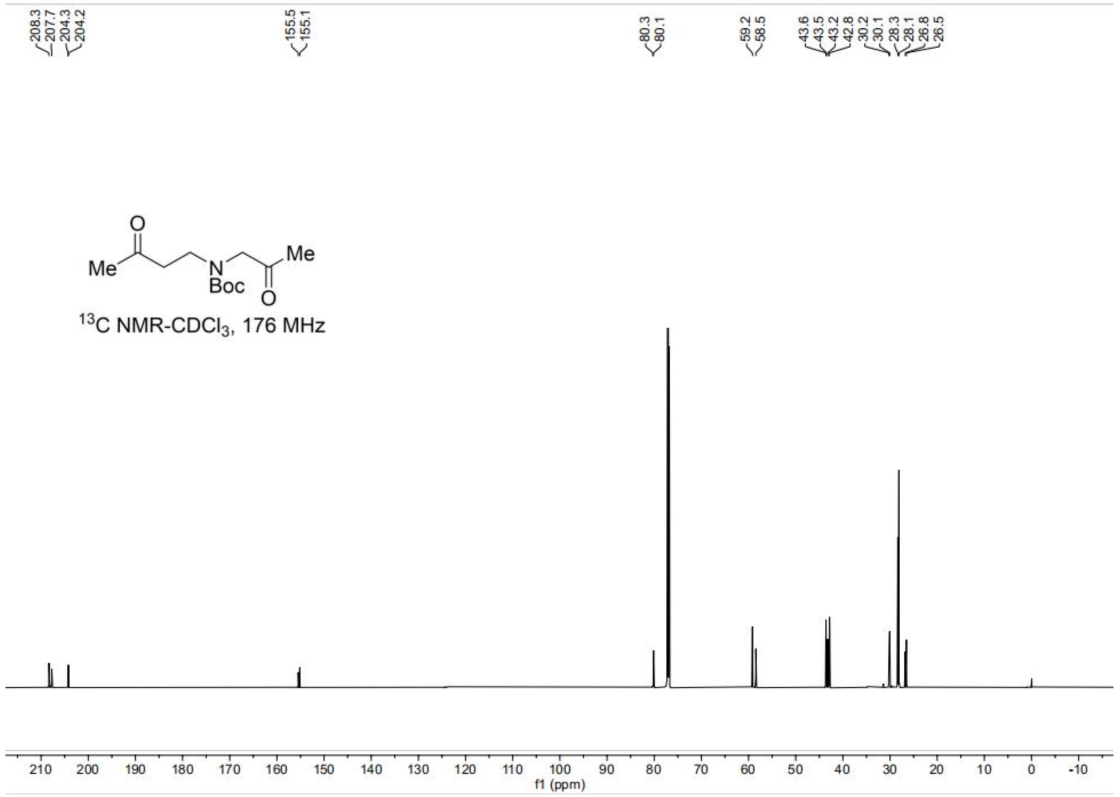

Supplementary Fig. 92. NMR of compound **43b** in $CDCl_3$

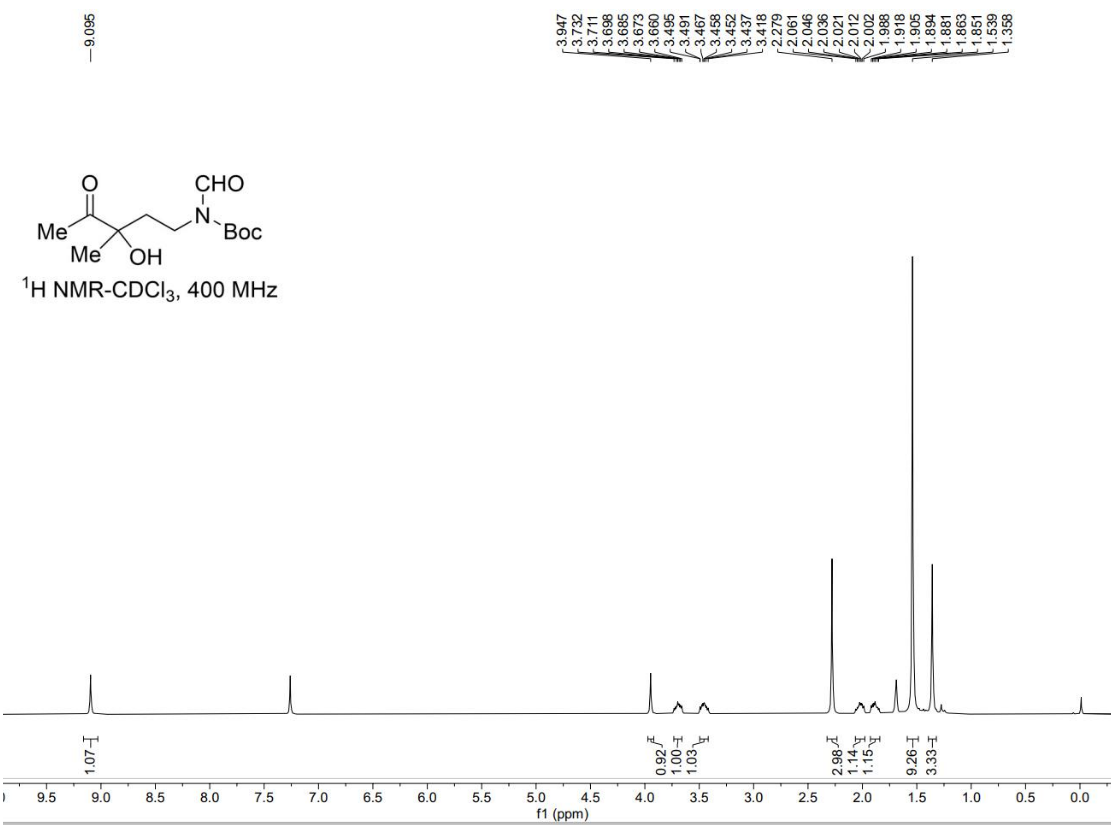

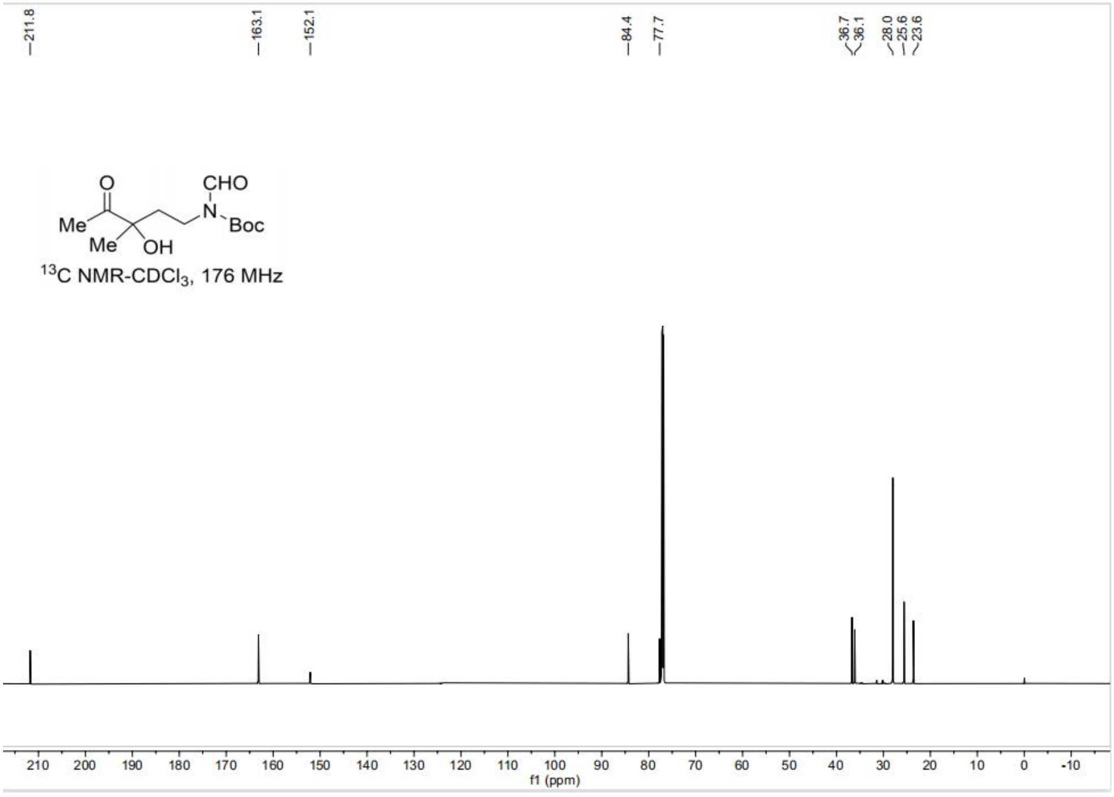

Supplementary Fig. 93. NMR of compound **43c** in $CDCl_3$

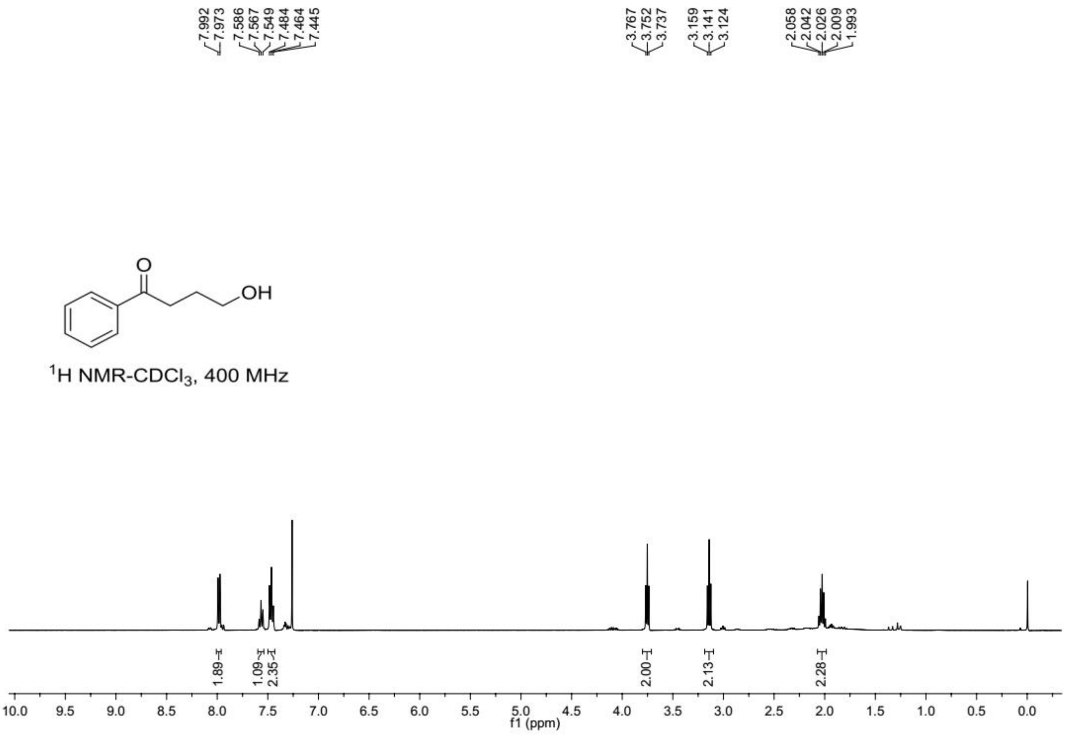

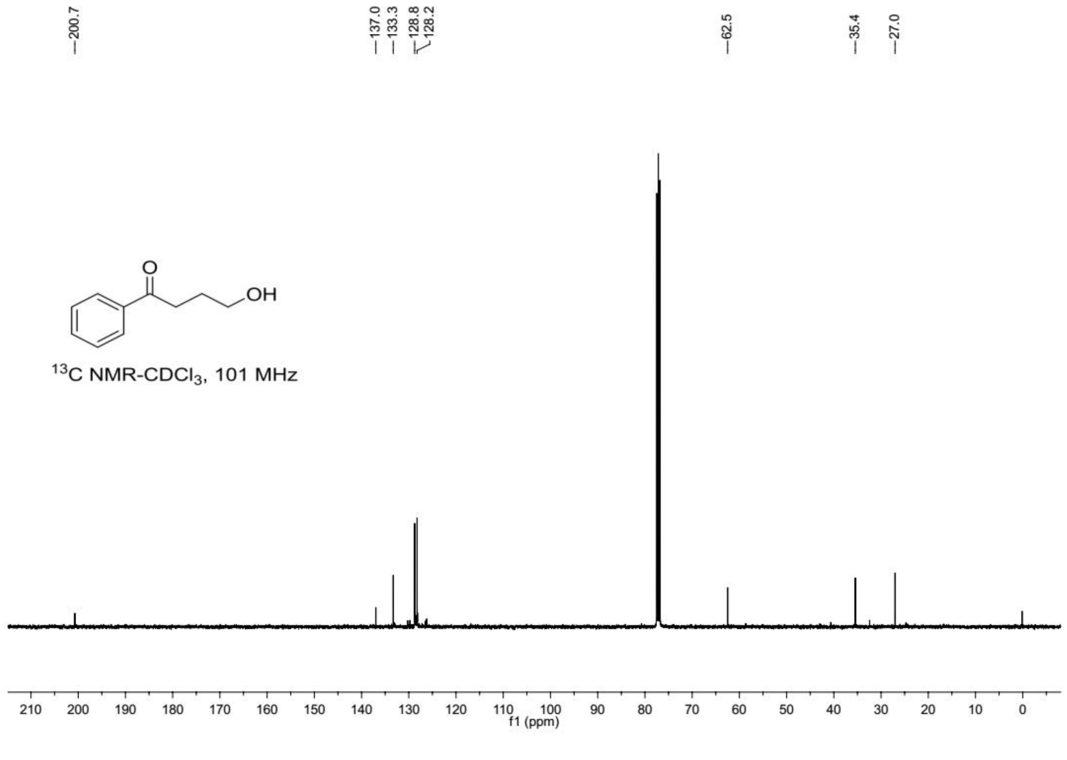

Supplementary Fig. 94. NMR of compound **44b** in $CDCl_3$

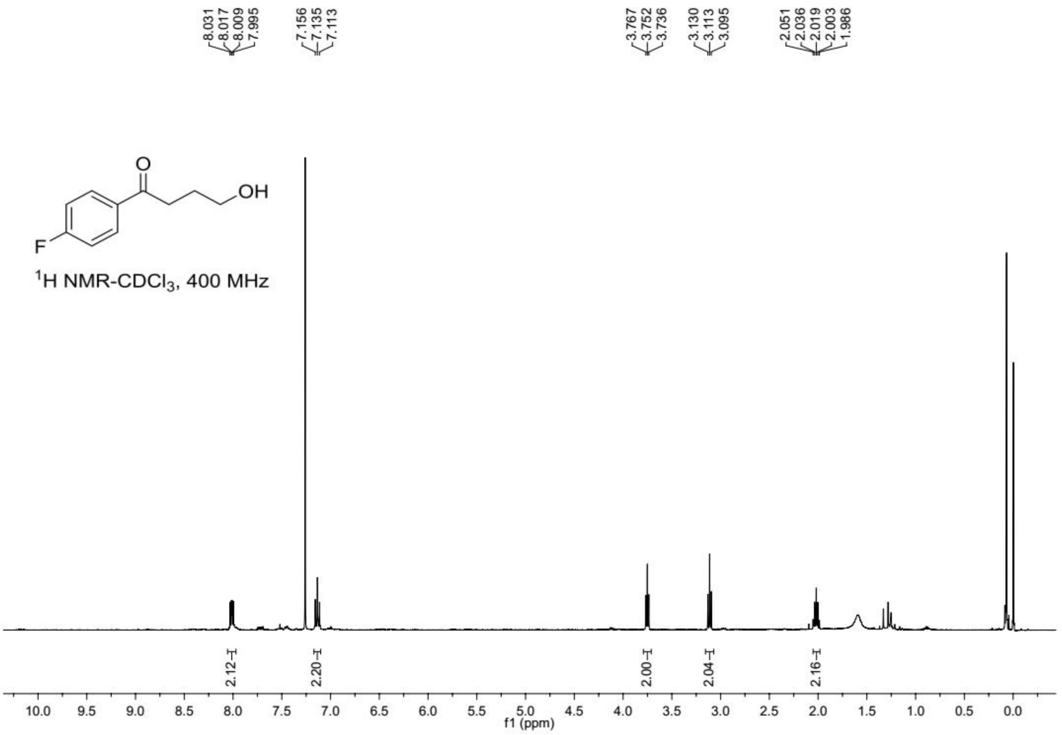

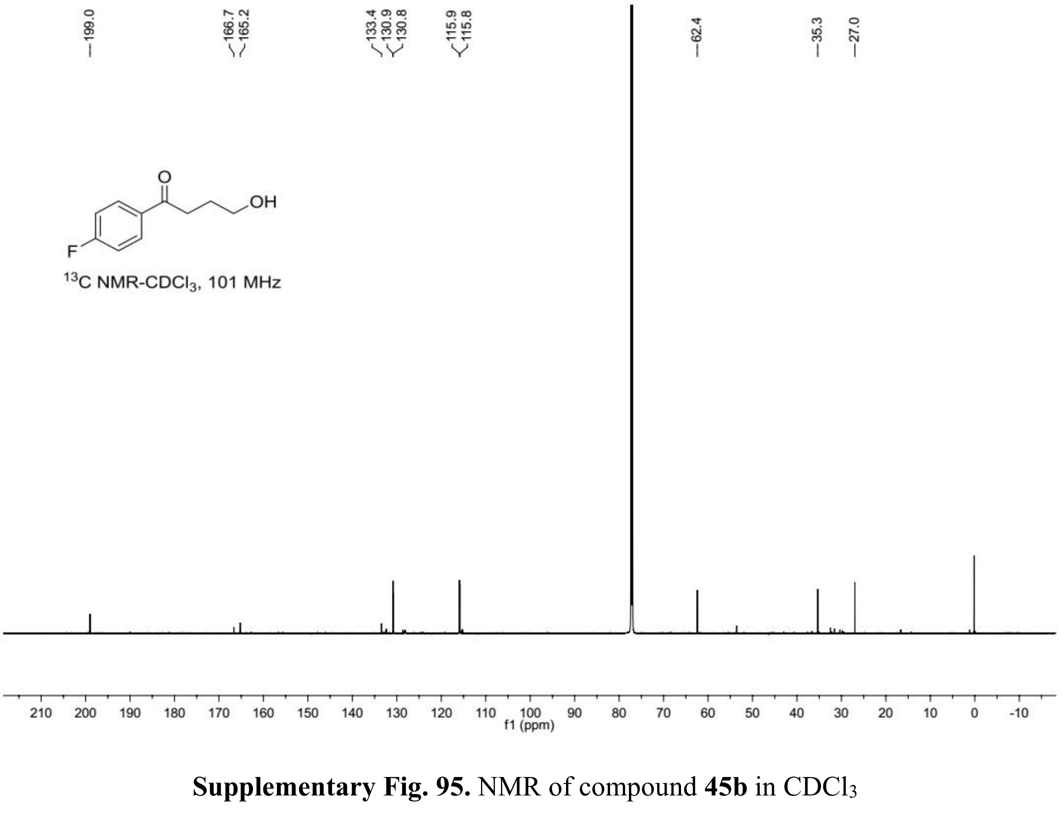

Supplementary Fig. 95. NMR of compound **45b** in $CDCl_3$

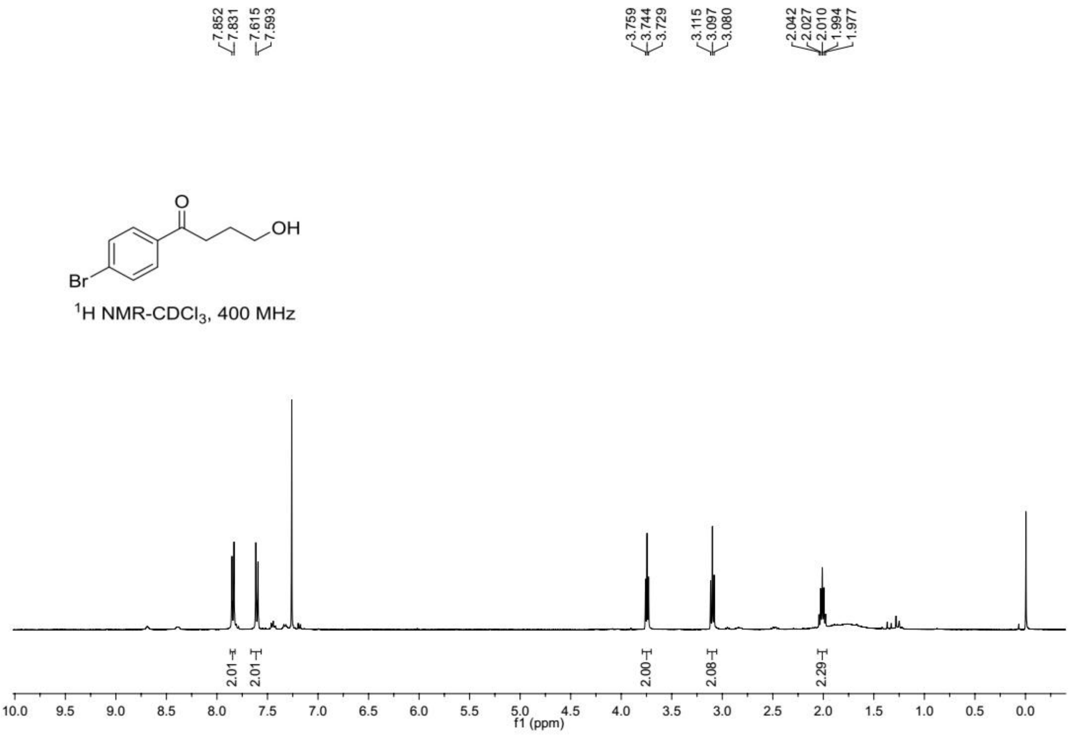

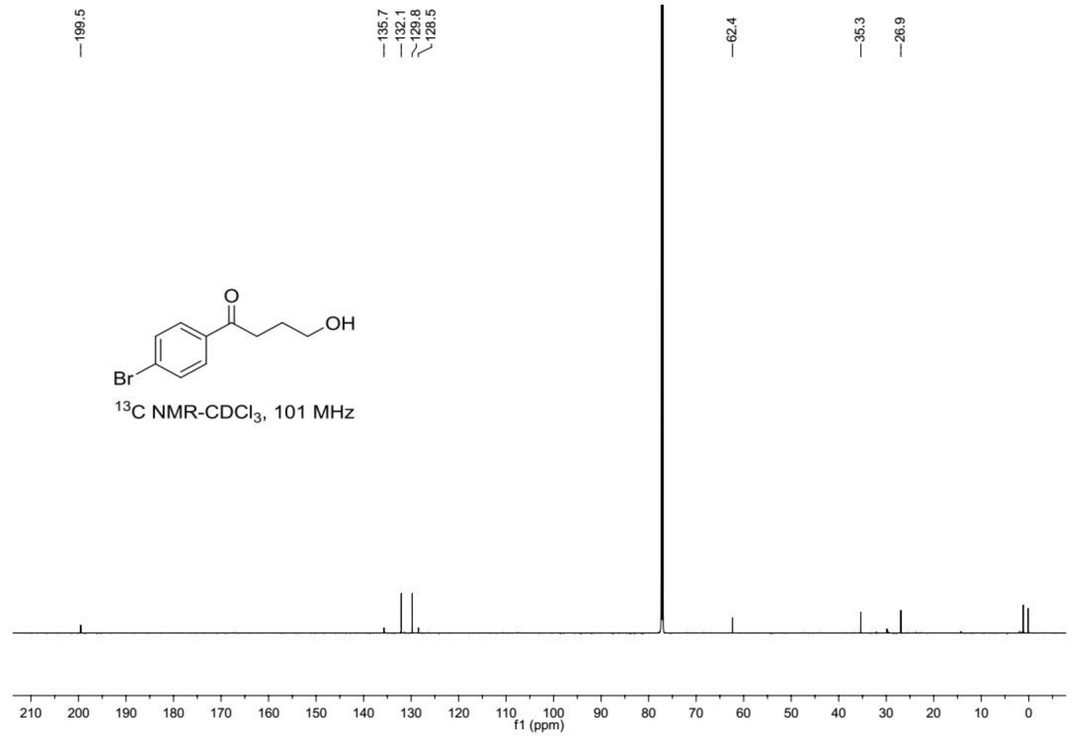

Supplementary Fig. 96. NMR of compound **46b** in $CDCl_3$

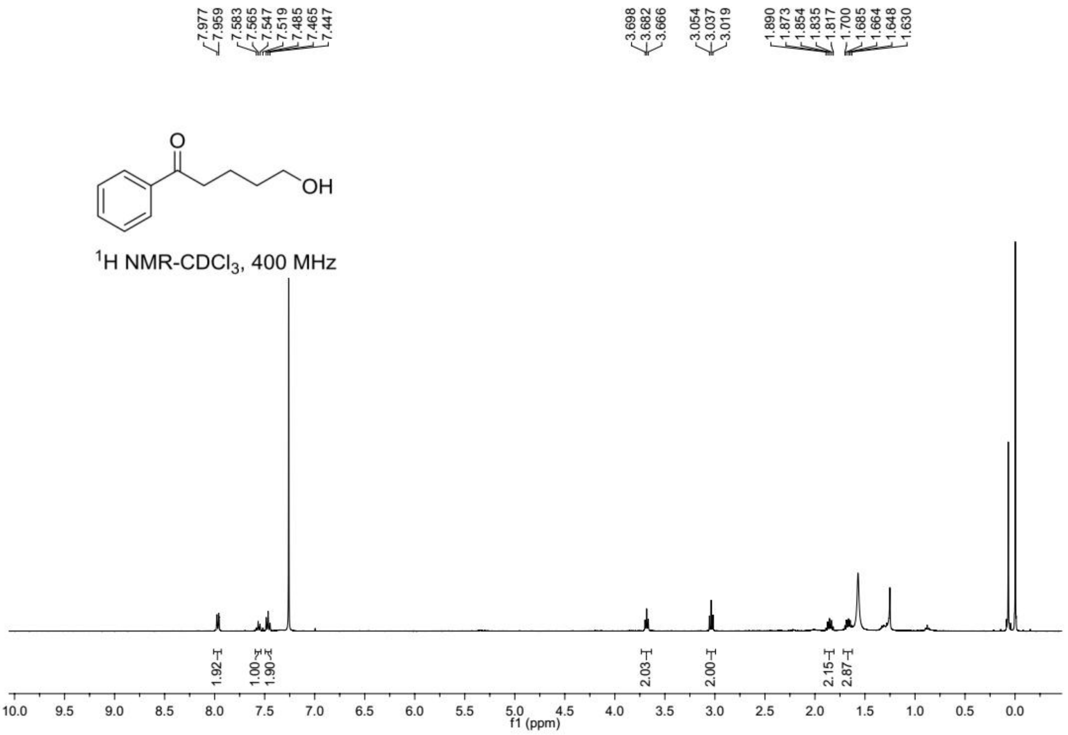

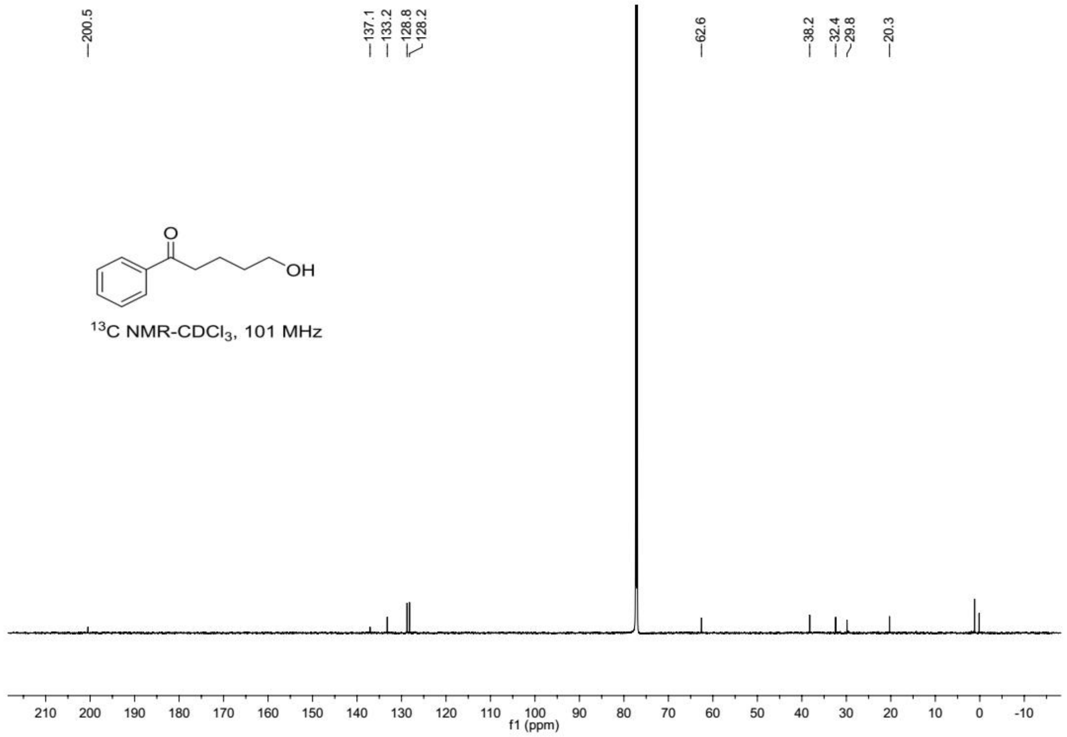

Supplementary Fig. 97. NMR of compound **47b** in $CDCl_3$

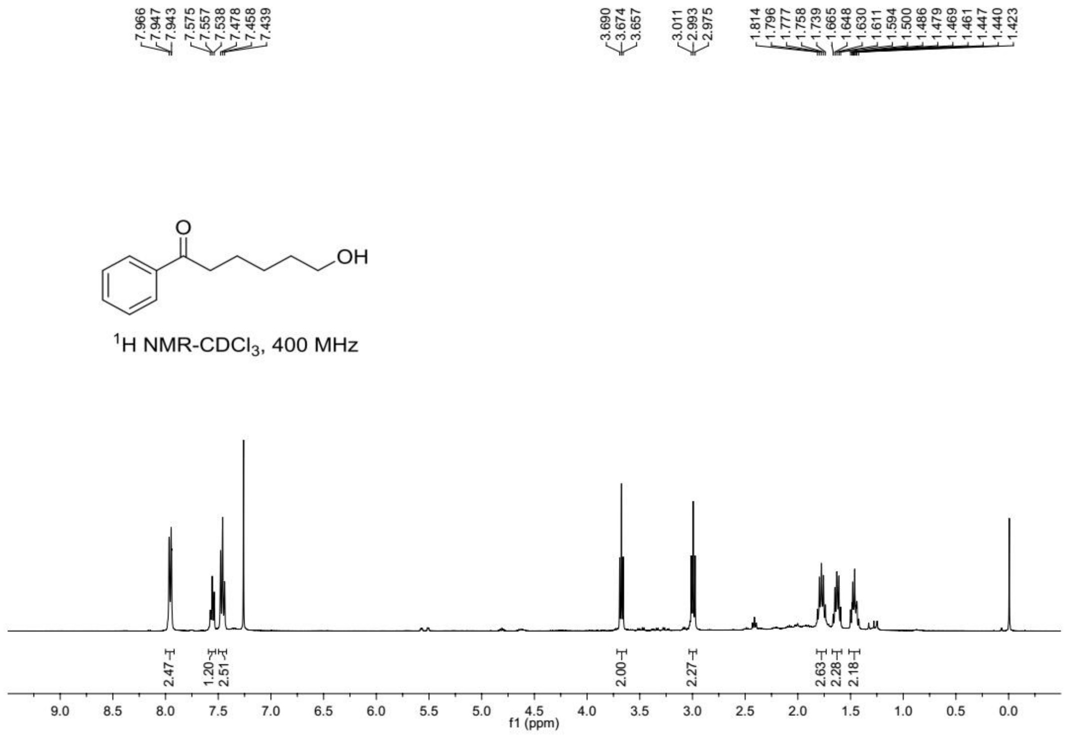

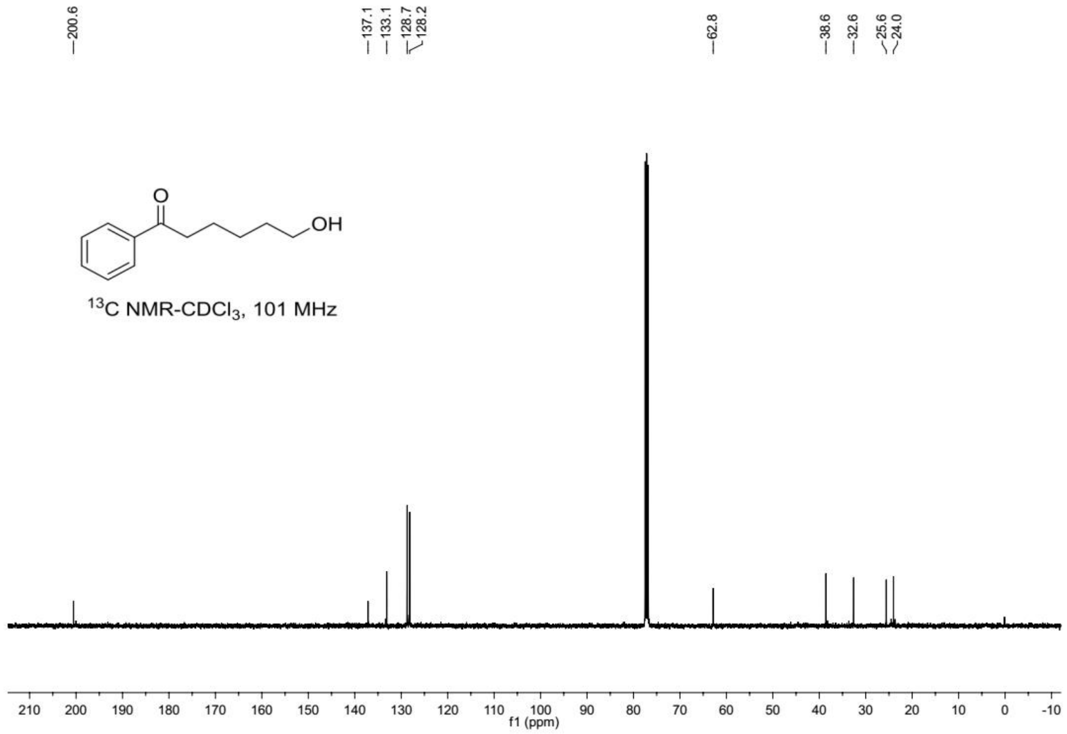

Supplementary Fig. 98. NMR of compound **48b** in $CDCl_3$

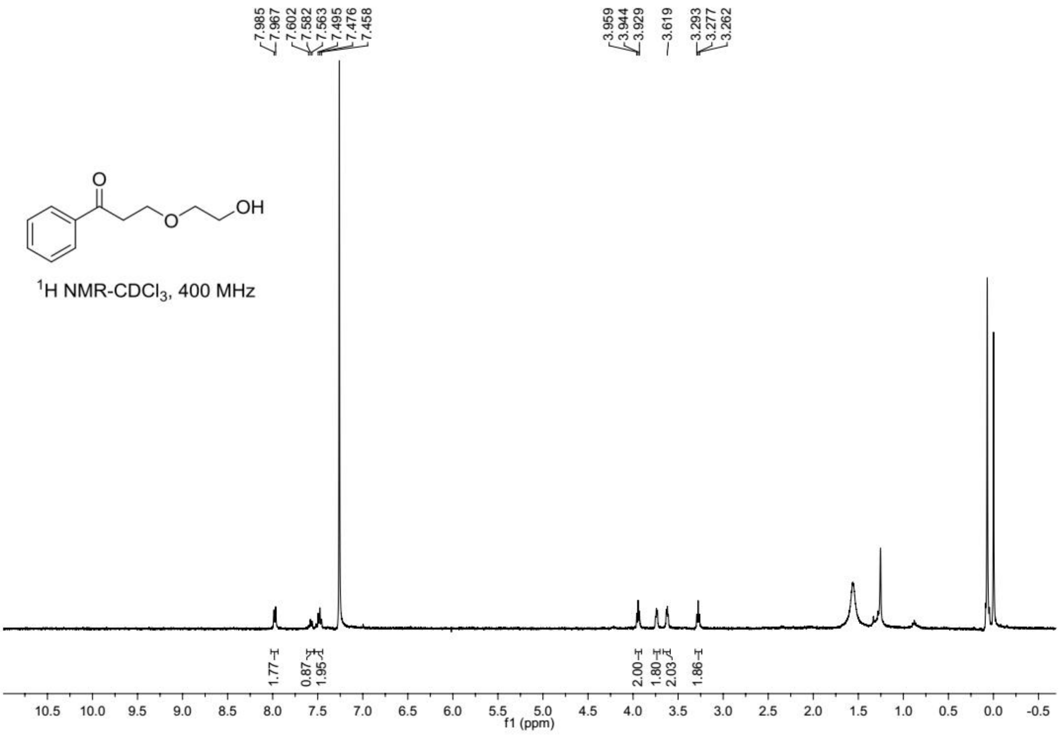

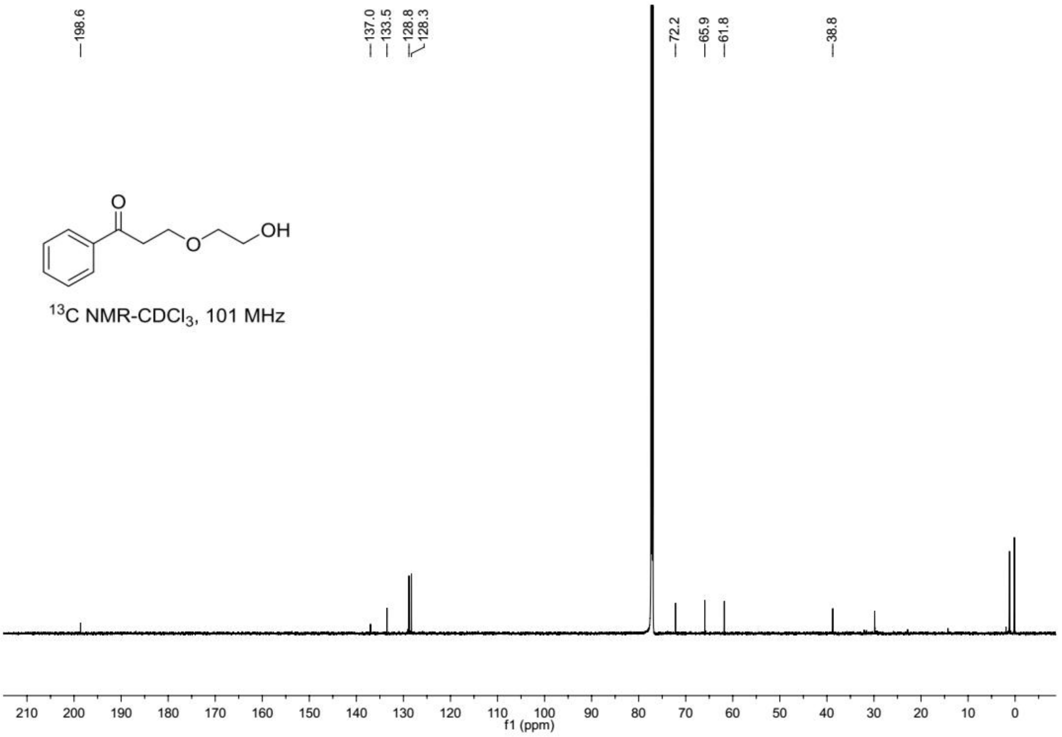

Supplementary Fig. 99. NMR of compound **49b** in $CDCl_3$

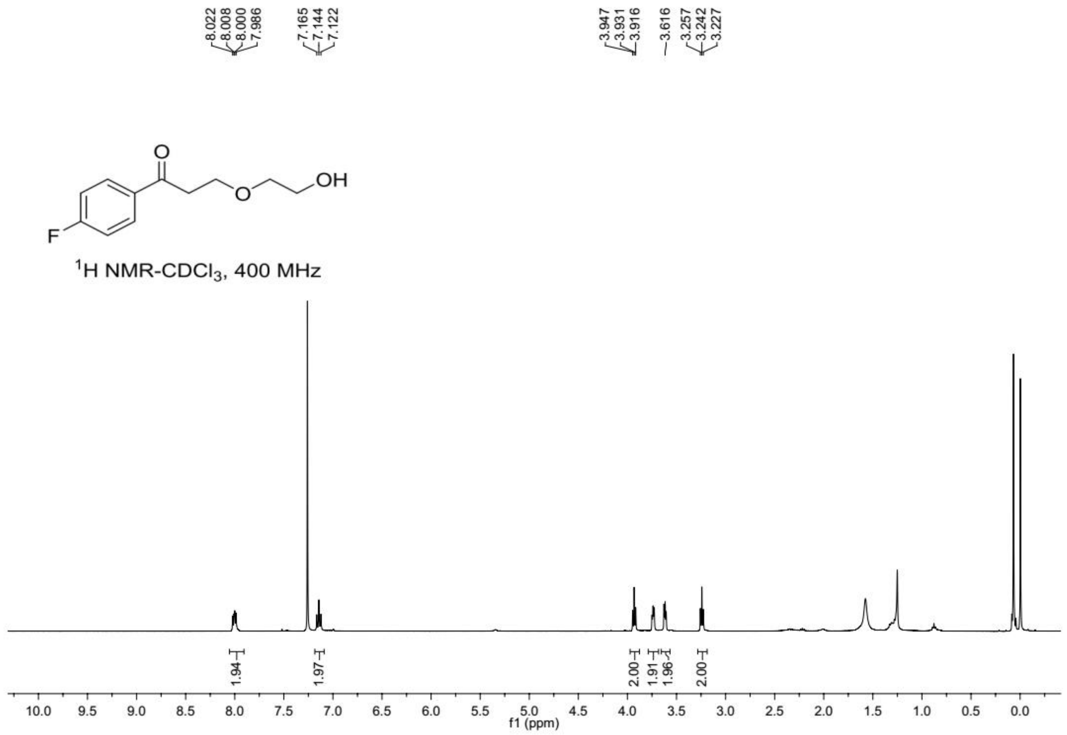

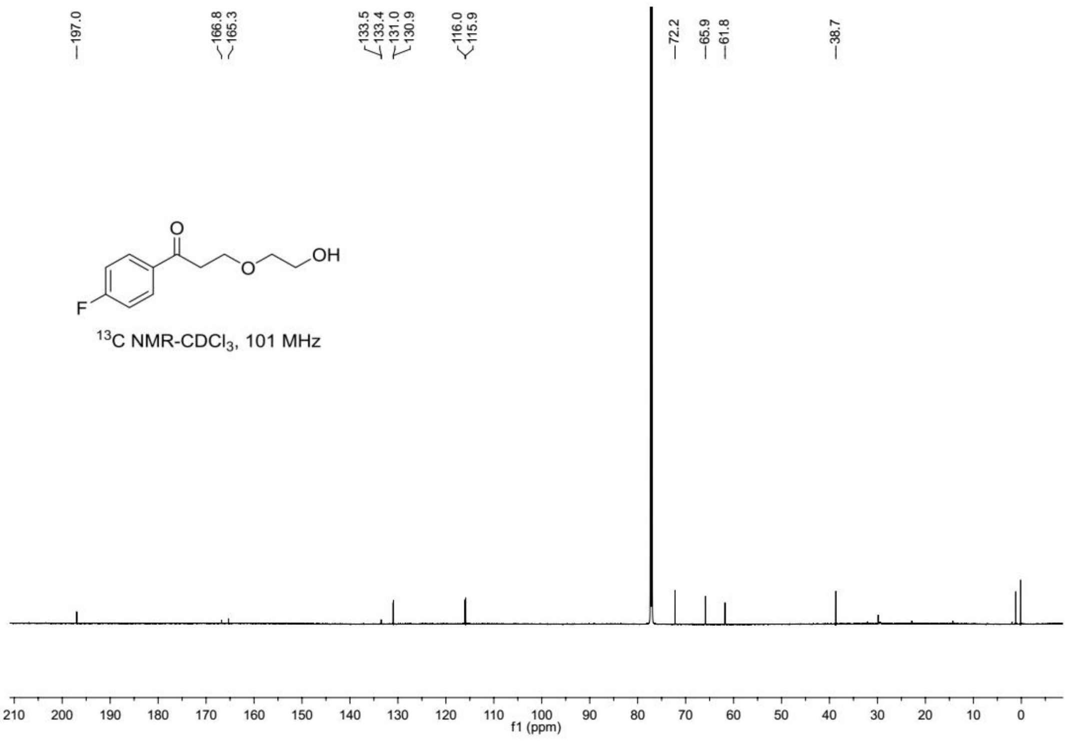

Supplementary Fig. 100. NMR of compound **50b** in $CDCl_3$

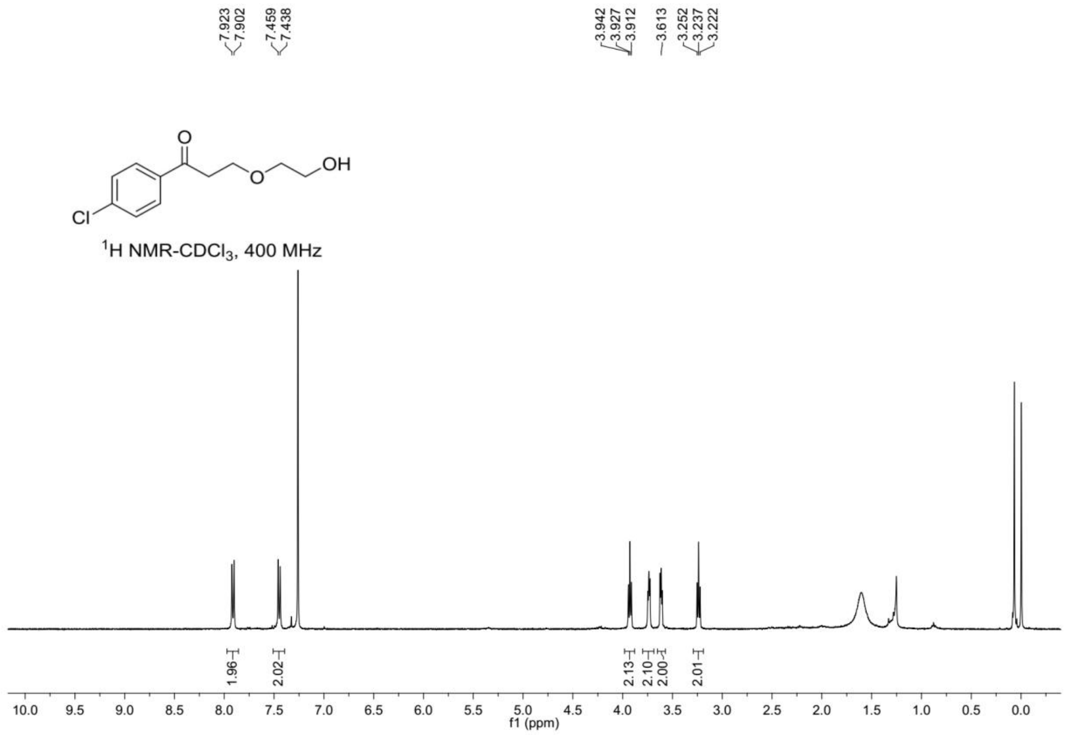

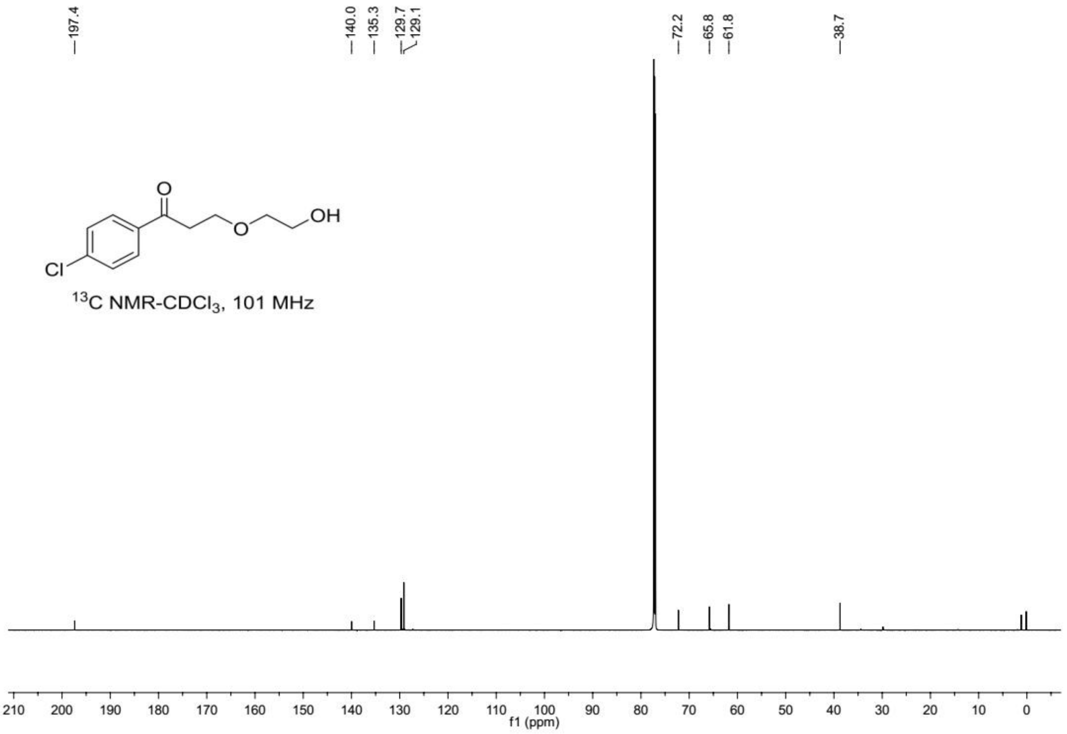

Supplementary Fig. 101. NMR of compound **51b** in $CDCl_3$

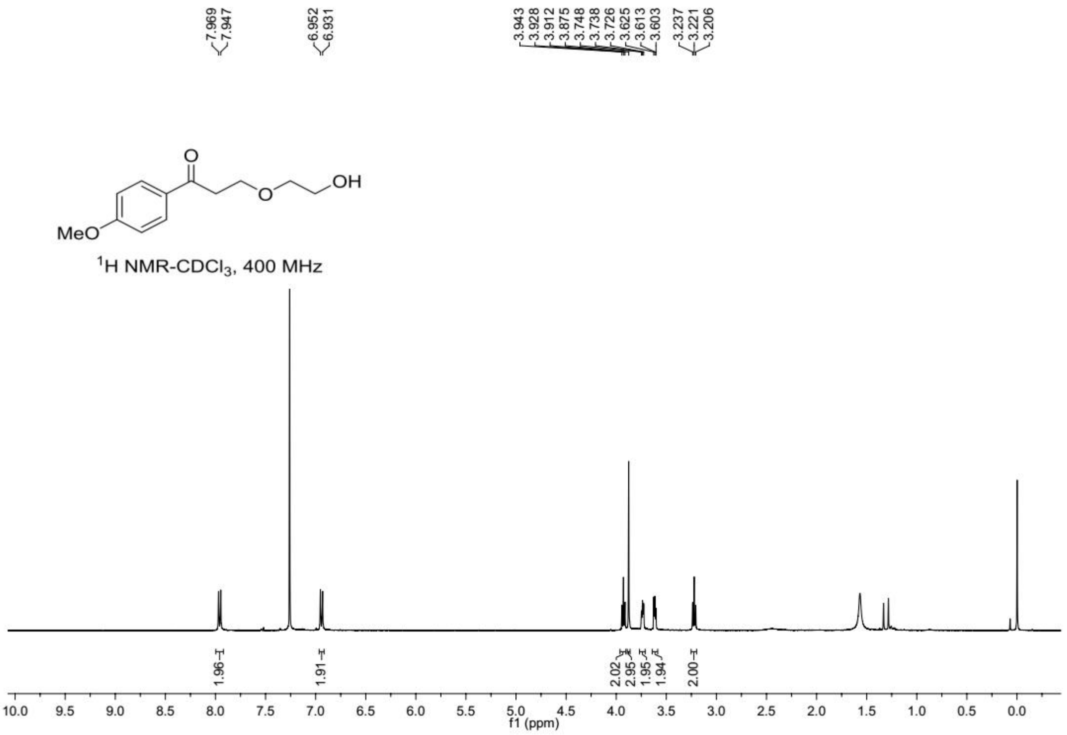

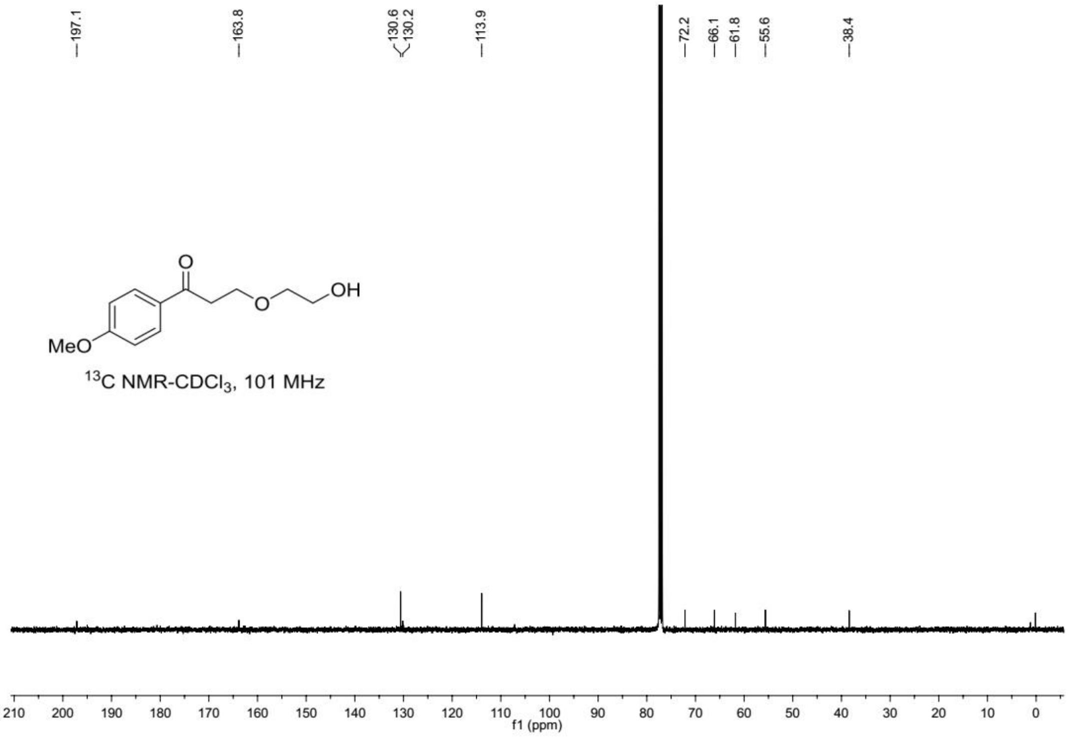

Supplementary Fig. 102. NMR of compound **52b** in $CDCl_3$

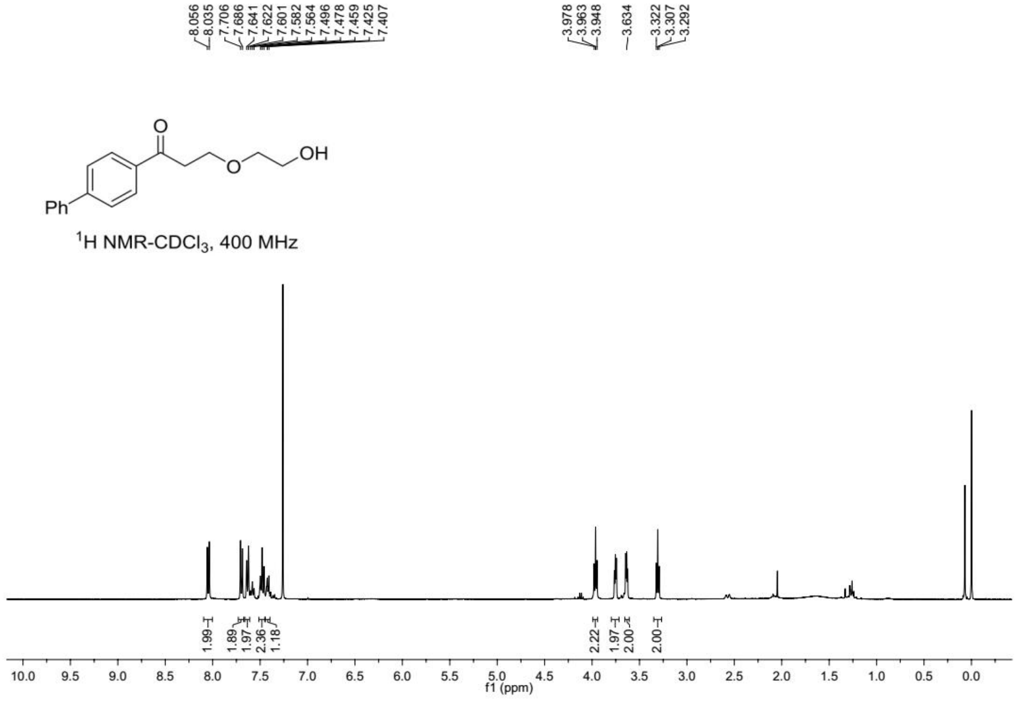

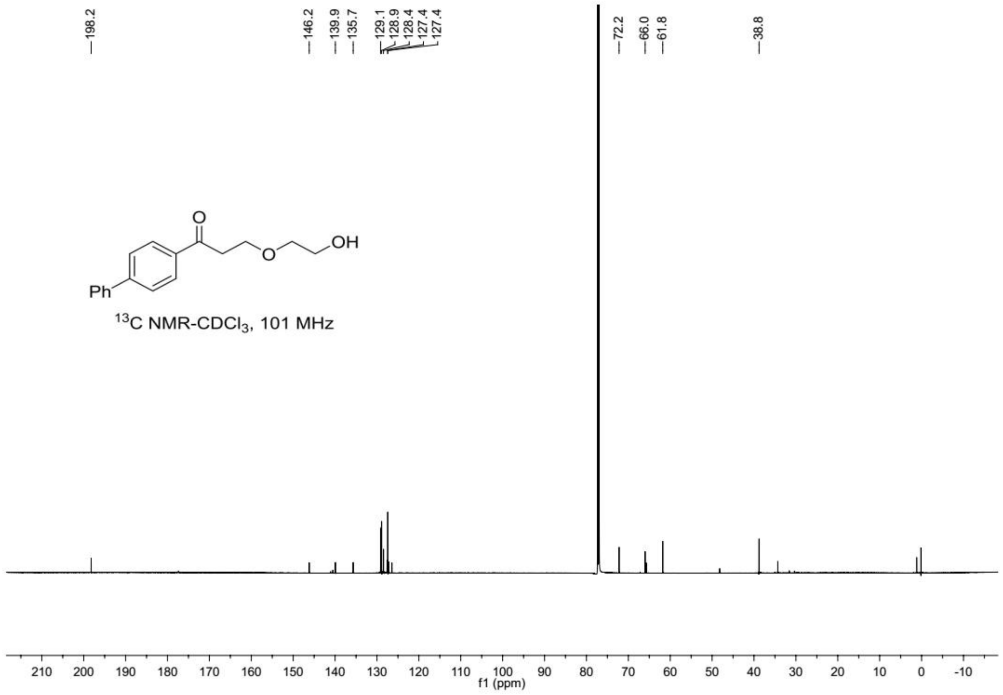

Supplementary Fig. 103. NMR of compound **53b** in $CDCl_3$

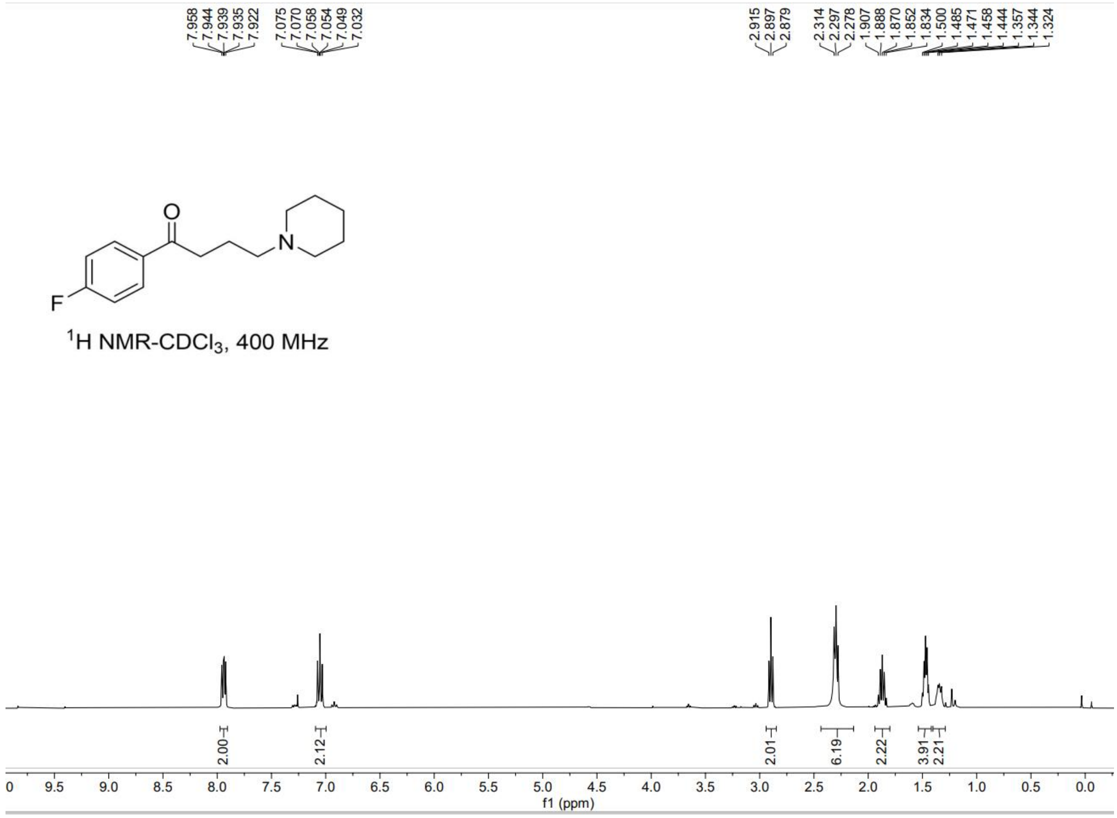

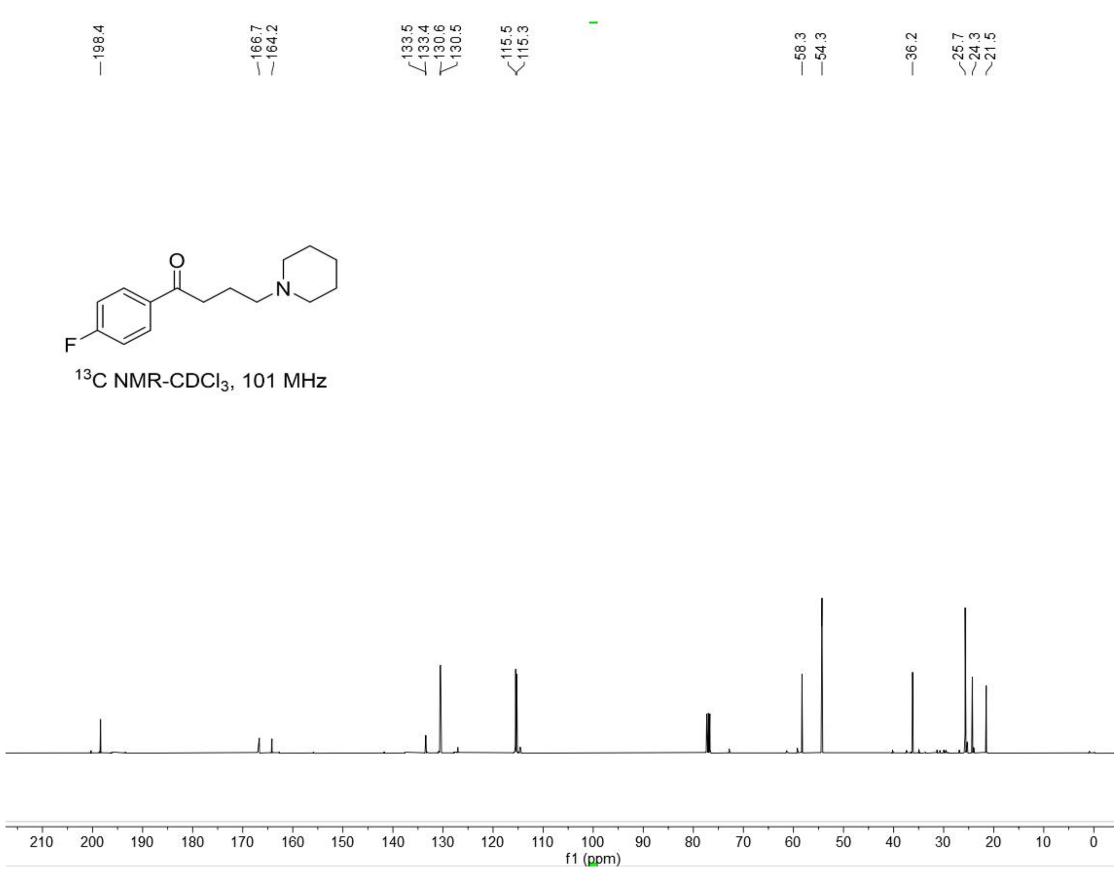

Supplementary Fig. 104. NMR of Primaperone in $CDCl_3$

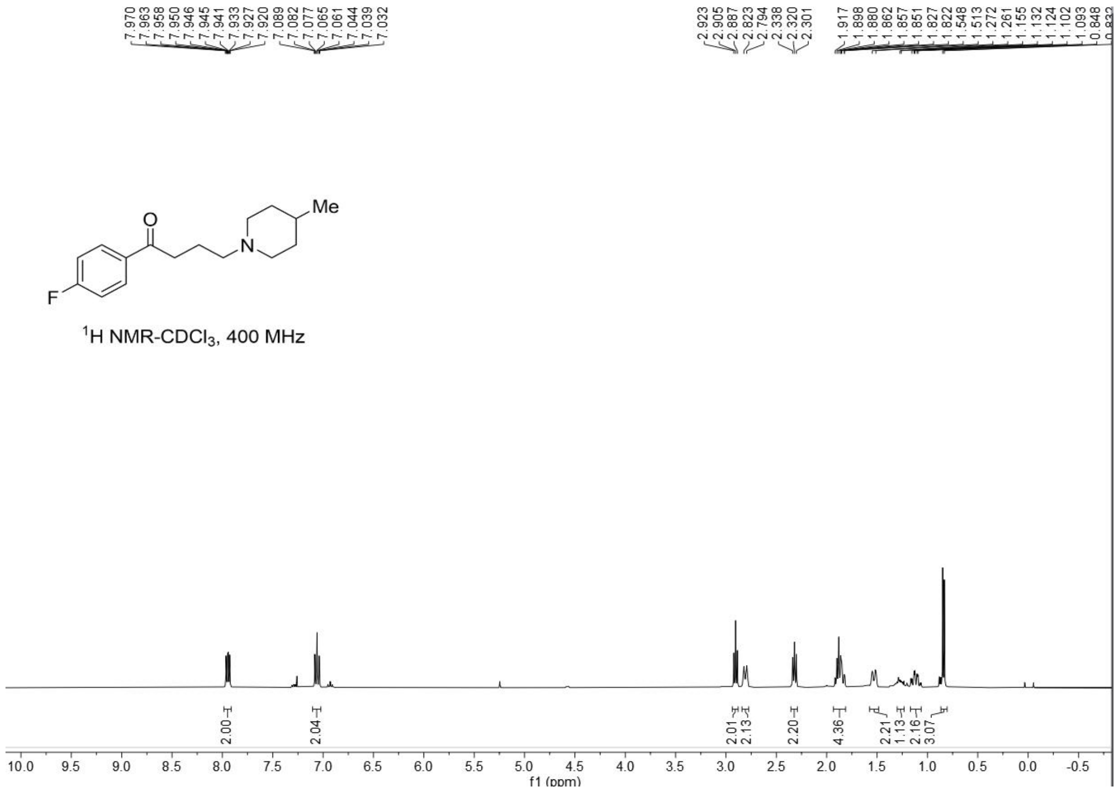

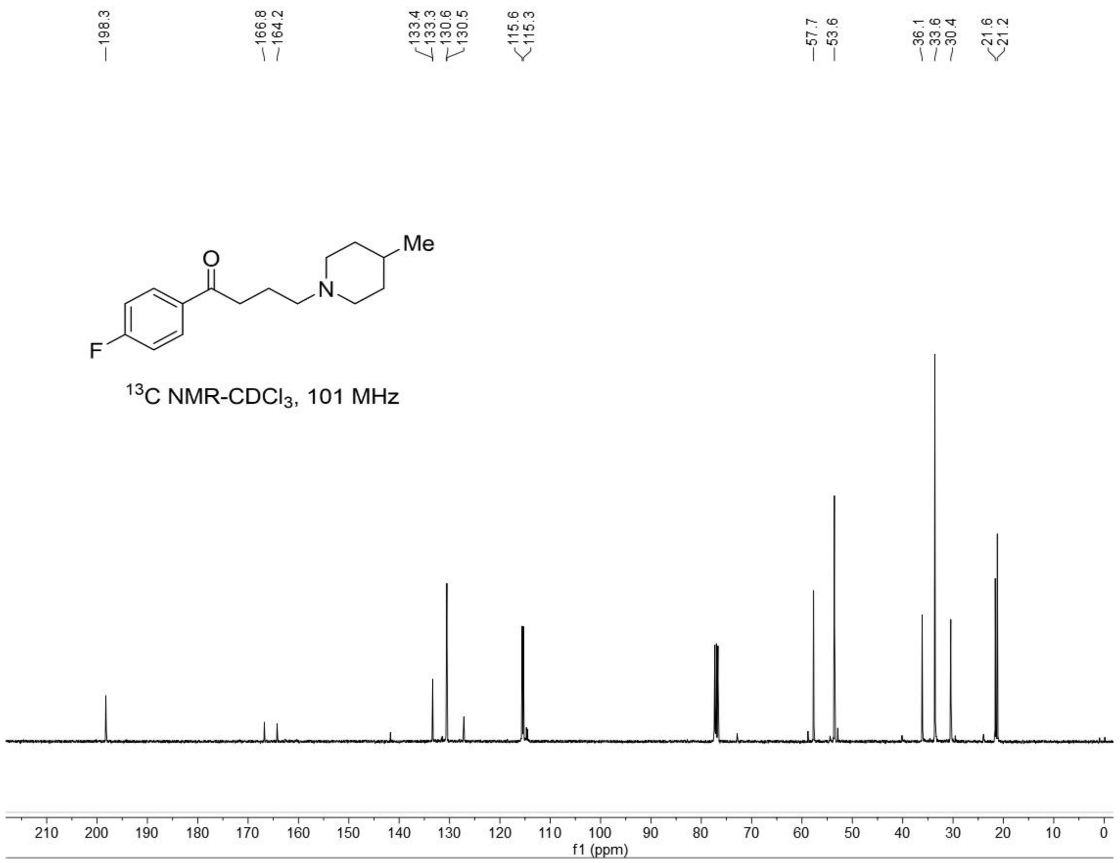

Supplementary Fig. 105. NMR of Melperone in $CDCl_3$

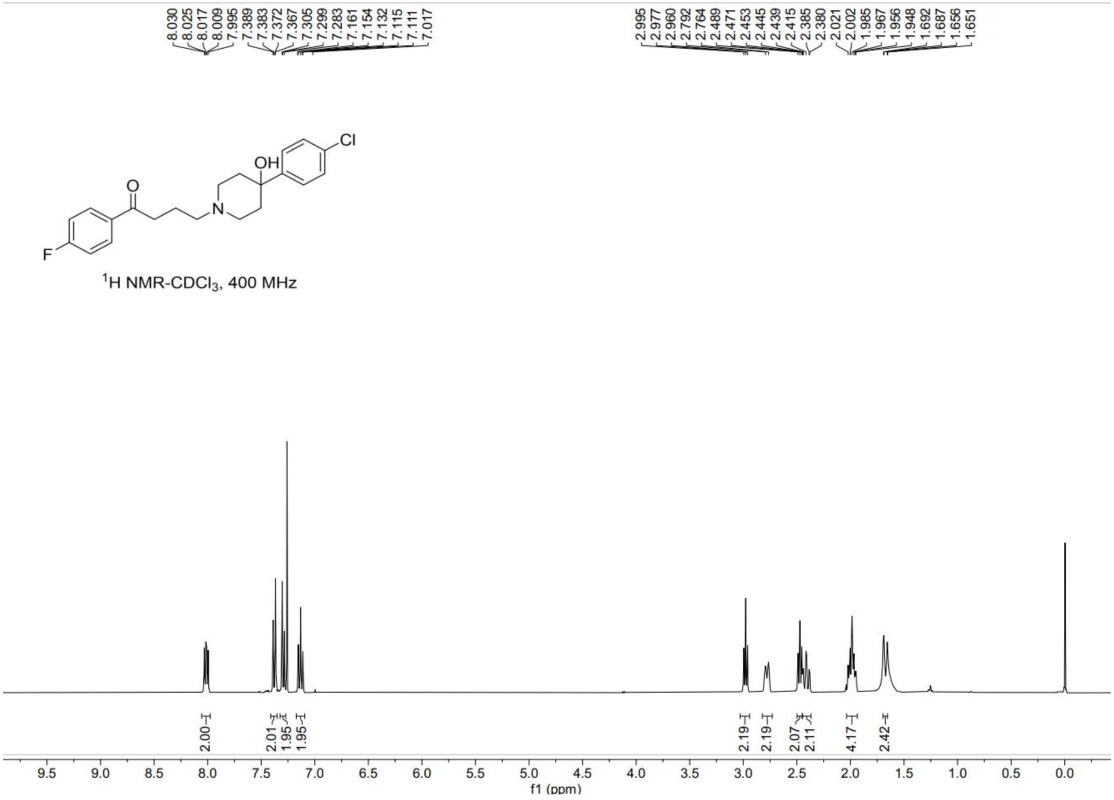

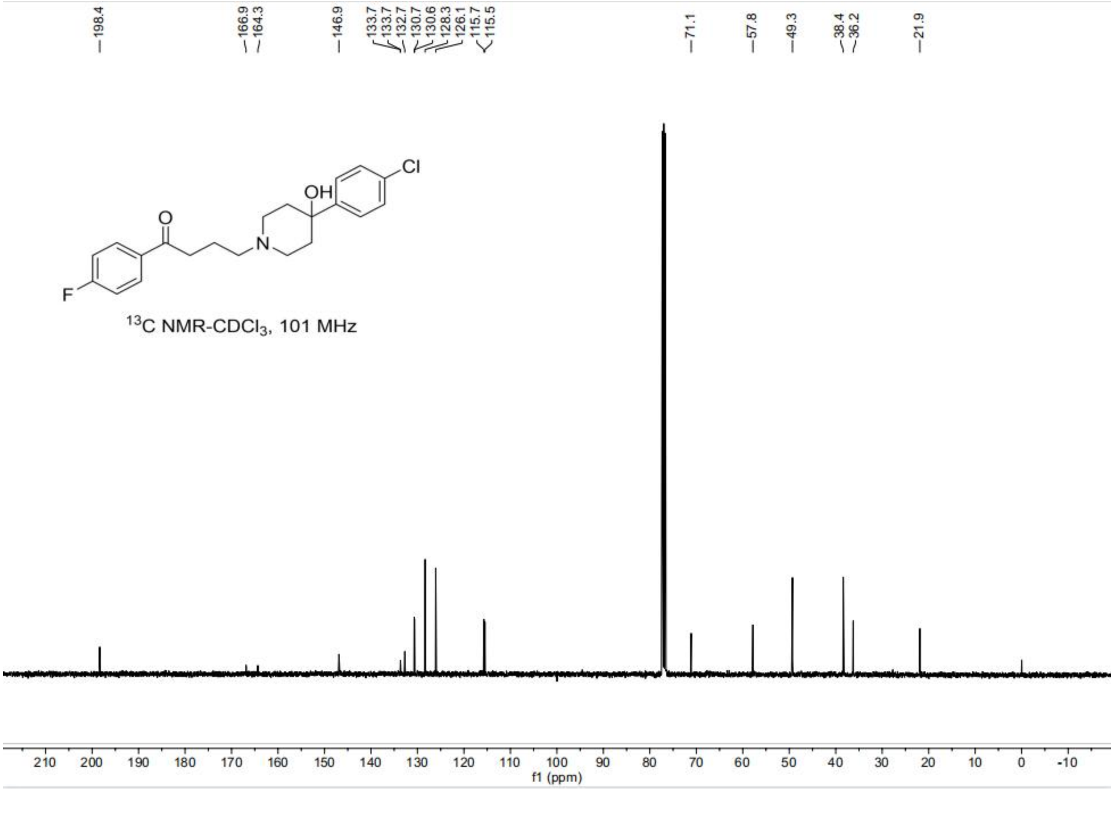

Supplementary Fig. 106. NMR of Haloperidol in $CDCl_3$

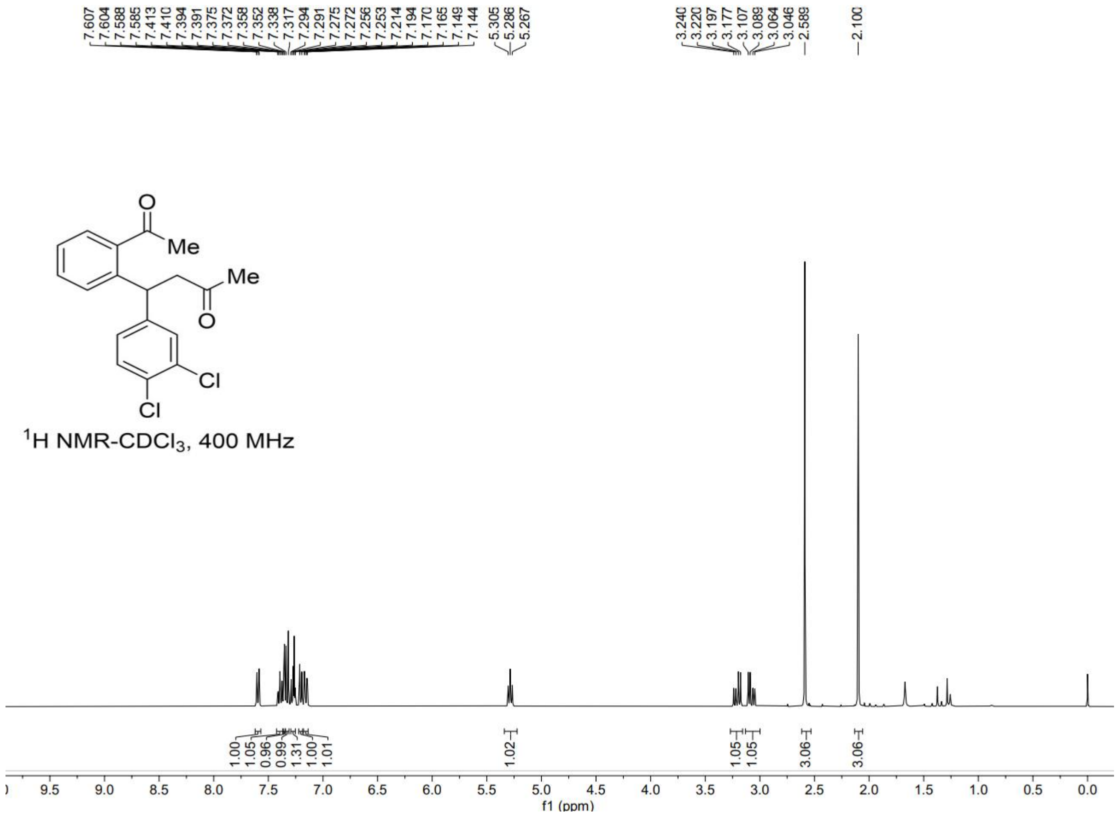

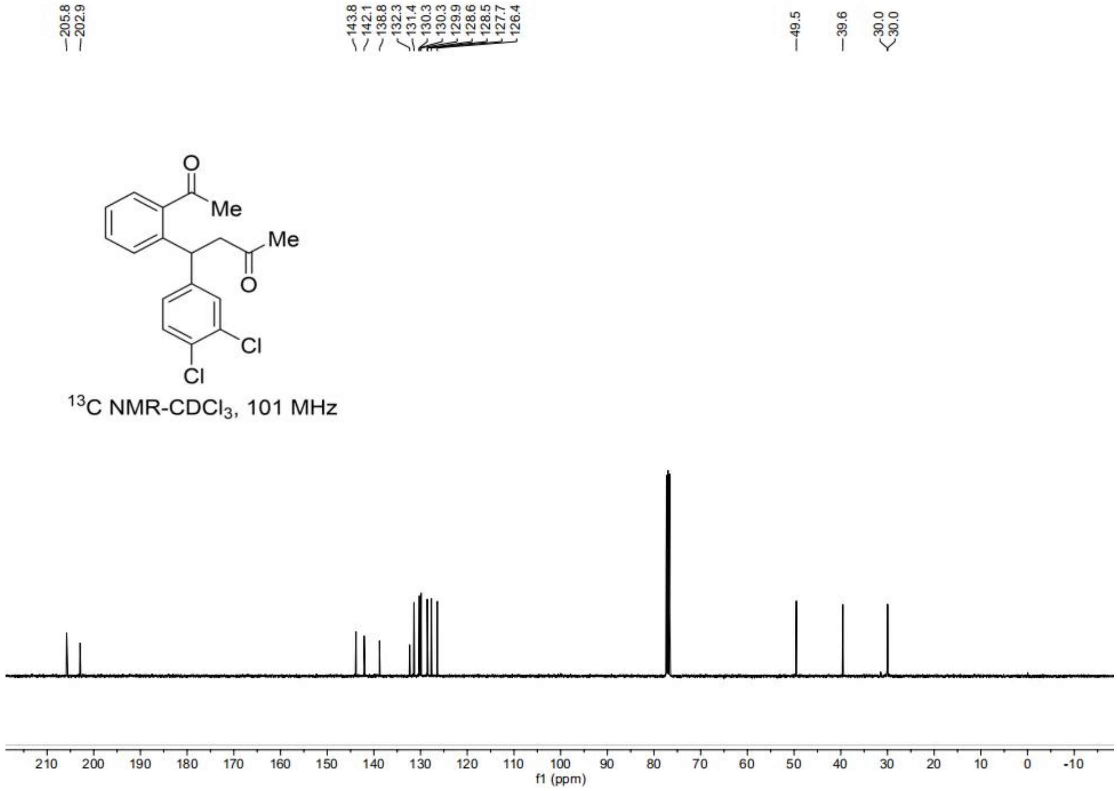

Supplementary Fig. 107. NMR of **54b** in $CDCl_3$

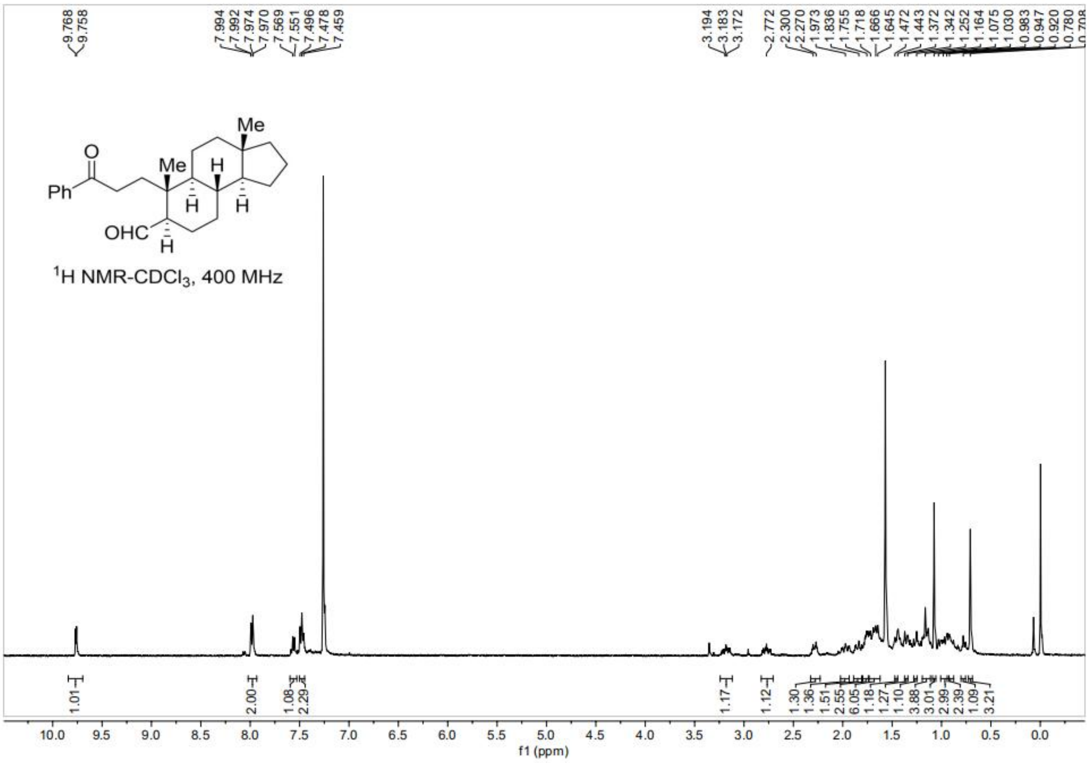

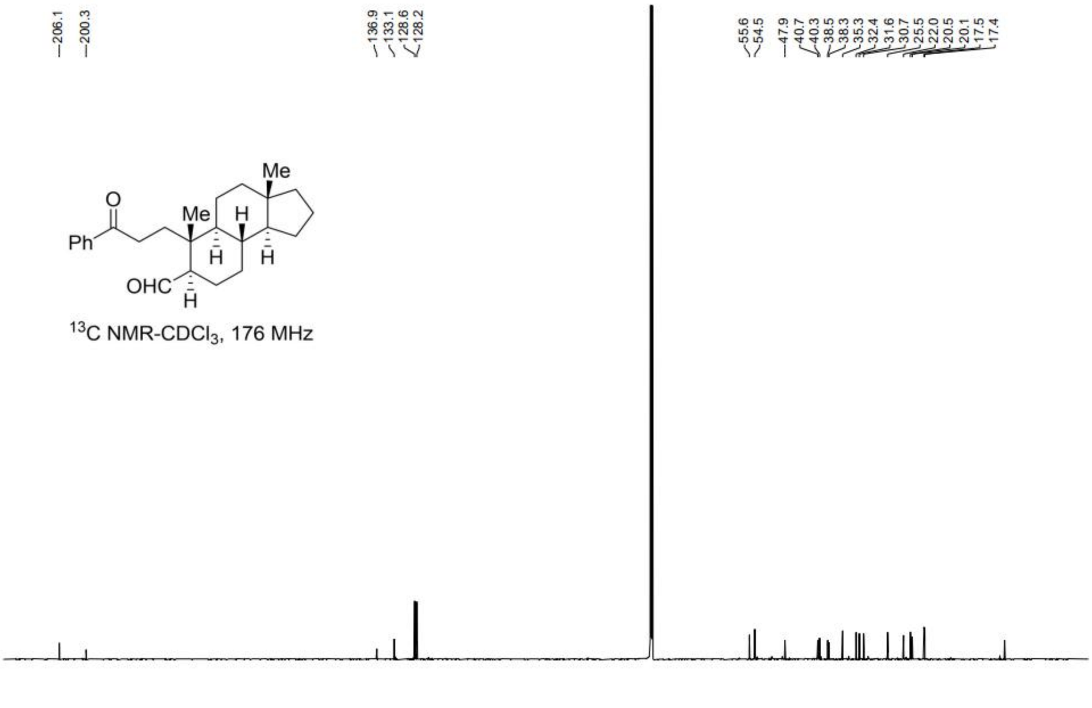

Supplementary Fig. 108. NMR of **55b** in CDCl$_3$

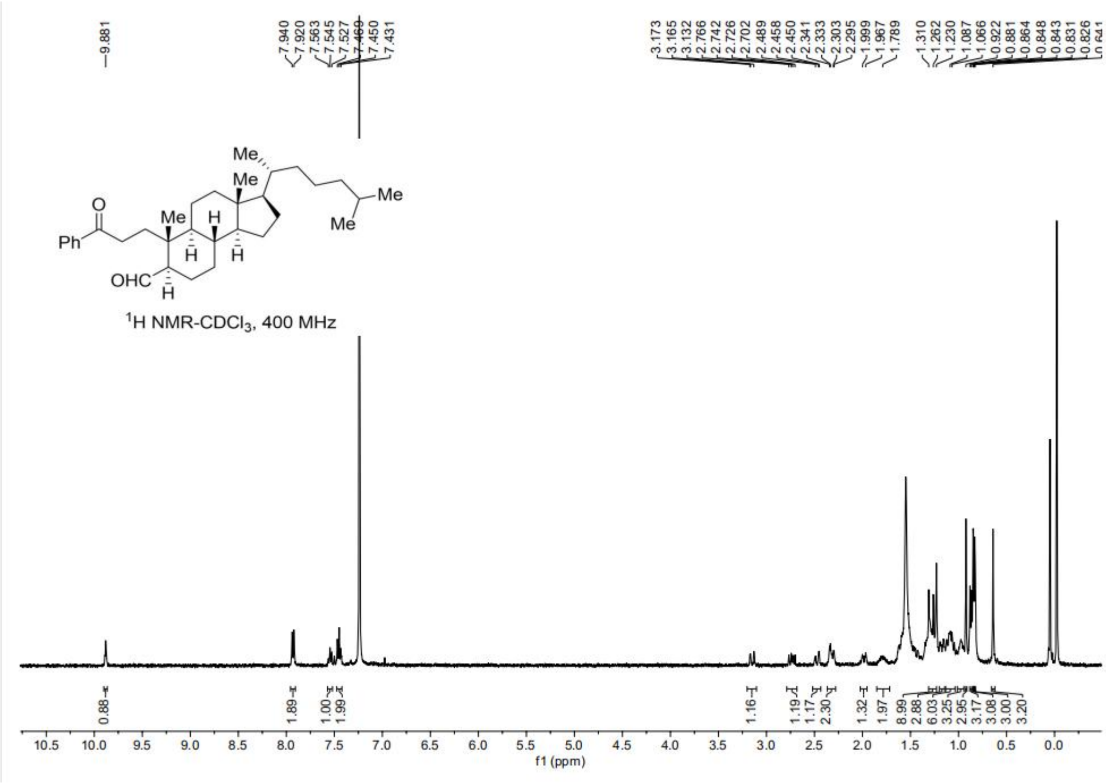

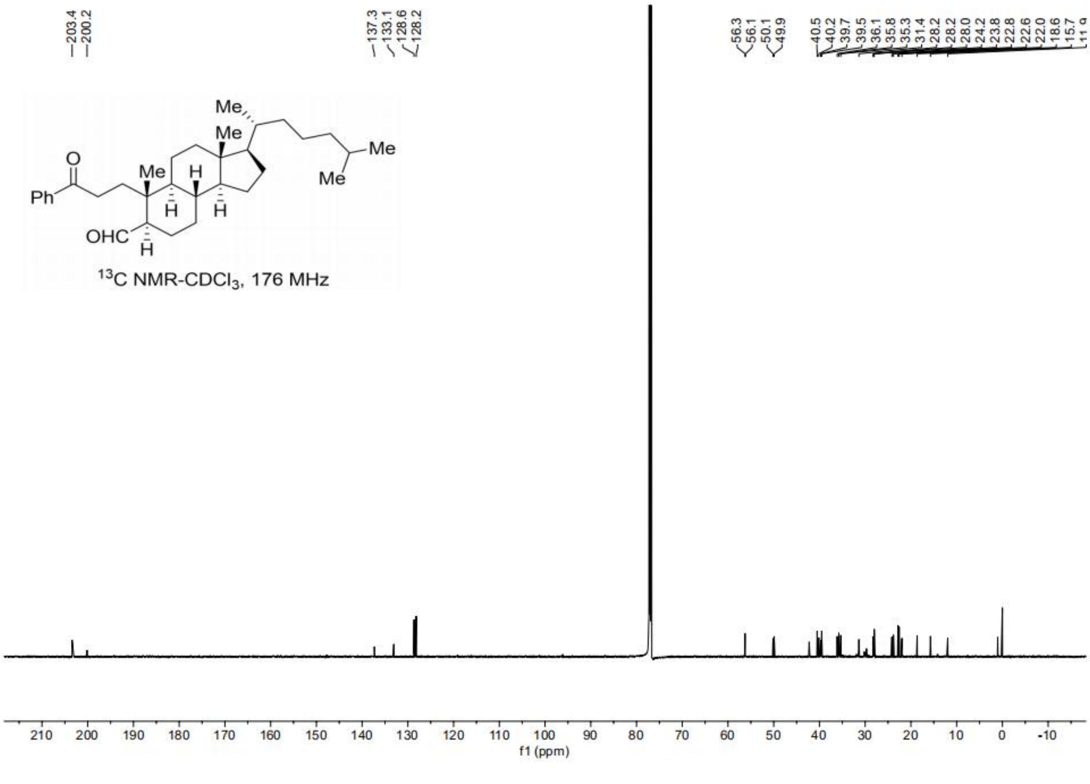

Supplementary Fig. 109. NMR of **56b** in $CDCl_3$

4 Supplementary References

1. Lowry, M. S., Goldsmith, J. I., Slinker, J. D., Rohl, R., Pascal, R. A., Malliaras, G. G. & Bernhard, S. Single-layer electroluminescent devices and photoinduced hydrogen production from an ionic iridium(III) complex. *Chem. Mater.* **17**, 5712-5719 (2005).
2. Liu, Y., Yin, Y., Zhang, Z., Li, C. J., Zhang, H., Zhang, D., Jiang, C., Nomie, K., Zhang, L., Wang, M. L. & Zhao, G. Structural optimization elaborates novel potent Akt inhibitors with promising anticancer activity. *Eur. J. Med. Chem.* **138**, 543-551 (2017).
3. Zhang, D., Tong, D., Yang, D., Sun, J., Zhang, F. & Zhao, G. Design, synthesis and biological evaluation of AKT inhibitors bearing a piperidin-4-yl appendant. *Med. Chem. Commun.* **9**, 1340-1350 (2018).
4. Agekyan, A. A. & Mkryan, G. G. Synthesis of *p*-aminobenzoic acid diamides based on 4-(4-methoxyphenyl)tetrahydro-2*H*-pyran-4-carboxylic acid and [4-(4-methoxyphenyl) tetrahydro-2*H*-pyran-4-yl]methylamine. *Russ. J. Gen. Chem.* **85**, 1057-1062 (2015).
5. Ferreira, A. J., Solano, D. M., Oakdale, J. S. & Kurth M. J. Novel trinitrogen-containing triheterocycles via the intramolecular nitrile oxide cycloaddition reaction. *Synthesis* **20**, 3241-3246 (2011).
6. Alkayal, A., Tabas, V., Montanaro, S., Wright, I. A., Malkov, A. V. & Buckley, B. R. Harnessing applied potential: selective β-hydrocarboxylation of substituted olefins. *J. Am. Chem. Soc.* **142**, 1780-1785 (2020).
7. Minato, D., Arimoto, H., Nagasue, Y., Demizu, Y. & Onomura, O. Asymmetric electrochemical oxidation of 1,2-diols, aminoalcohols, and aminoaldehydes in the presence of chiral copper catalyst. *Tetrahedron* **64**, 6675-6683 (2008).
8. Poirier, M., Pujol-Giménez, J., Manatschal, C., Bühlmann, S., Embaby, A., Javor, S., Hediger, M. A. & Reymond, J. Pyrazolyl-pyrimidones inhibit the function of human solute carrier protein SLC11A2 (hDMT1) by metal chelation. *RSC Med. Chem.* **11**, 1023-1031 (2020).
9. Mir, N. A., Ramaraju, P., Vanaparthi, S., Choudhary, S., Singh, R. P., Sharma, P., Kant, R., Singh, R., Sankaranarayanan, M. & Kumar, I. Sequential multicomponent catalytic synthesis of pyrrole-3-carboxaldehydes: evaluation of antibacterial and antifungal activities along with docking studies. *New J. Chem.* **44**, 16329-16339 (2020).
10. Shimkin, K. W., Gildner, P. G., & Watson, D. A. Copper-catalyzed alkylation of nitroalkanes with α-bromonitriles: synthesis of β-cyanonitroalkanes. *Org. Lett.* **18**. 988-991 (2016).
11. Lee, S., Bae, H. Y. & List, B. Can a ketone be more reactive than an aldehyde? catalytic asymmetric synthesis of substituted tetrahydrofurans. *Angew. Chem. Int. Ed.* **57**, 12162-12166 (2018).
12. Rono, L. J., Yayla, H. G., Wang, D. Y., Armstrong, M. F. & Knowles, R. R. Enantioselective photoredox catalysis enabled by proton-coupled electron transfer: development of an asymmetric aza-pinacol cyclization. *J. Am. Chem. Soc.* **135**, 17735-17738 (2013).
13. Mukhopadhyay, S. & Batra, S. Direct transformation of arylamines to aryl halides via sodium nitrite and N-halosuccinimide. *Chem. Eur. J.* **24**, 14622-14626 (2018).
14. Tachinami, T., Nishimura, T., Ushimaru, R., Noyori, R. & Naka, H. Hydration of terminal alkynes catalyzed by water-soluble cobalt porphyrin complexes. *J. Am. Chem. Soc.* **135**, 50-53 (2013).

15. Kariofillis, S. K., Shields, B. J., Tekle-Smith, M. A., Zacuto, M. J. & Doyle, A. G. Nickel/photoredox-catalyzed methylation of (hetero)aryl chlorides using trimethyl orthoformate as a methyl radical source. *J. Am. Chem. Soc.* **142**, 7683-7689 (2020).
16. Mack, J. B. C., Gipson, J. D., Bois, J. D. & Sigman, M. S. Ruthenium-catalyzed C-H hydroxylation in aqueous acid enables selective functionalization of amine derivatives. *J. Am. Chem. Soc.* **139**, 9503-9506 (2017).
17. Gonzalez-de-Castro, A. & Xiao, J. Green and efficient: iron-catalyzed selective oxidation of olefins to carbonyls with O_2. *J. Am. Chem. Soc.* **137**, 8206-8218 (2015).
18. González, J. R., González, A. Z. & Soderquist, J. A. (E)-2-boryl-1,3-butadiene derivatives of the 10-TMS-9-BBDs: highly selective reagents for the asymmetric synthesis of *anti*-1,2-disubstituted 3,4-pentadien-1-ols. *J. Am. Chem. Soc.* **131**, 9924-9925 (2009).
19. Benischke, A. D., Anthore-Dalion, L., Berionni, G. & Knochel, P. Preparation of functionalized diaryl- and diheteroaryllanthanum reagents by fast halogen-lanthanum exchange. *Angew. Chem. Int. Ed.* **56**, 16390-16394 (2017).
20. Alam, M. I., Alam, M. A., Alam, O., Nargotra, A., Taneja, S. C. & Koul, S. Molecular modeling and snake venom phospholipase A_2 inhibition by phenolic compounds: structure-activity relationship. *Eur. J. Med. Chem.* **114**, 209-219 (2016).
21. Murphy, S. K. & Dong, V. M. Enantioselective ketone hydroacylation using Noyori's transfer hydrogenation catalyst. *J. Am. Chem. Soc.* **135**, 5553-5556 (2013).
22. Xie, J., Guo, L., Yang, X., Wang, L. & Zhou, Q. Enantioselective synthesis of 2,6-cis-disubstituted tetrahydropyrans via a tandem catalytic asymmetric hydrogenation/oxa-Michael cyclization: an efficient approach to (-)-centrolobine. *Org. Lett.* **14**, 4758-4761 (2012).
23. Hosokawa, T., Ohta, T., Kanayama, S. & Murahashi, S. Palladium(II)-catalyzed acetalization of terminal olefins bearing electron-withdrawing substituents with optically active diols. *J. Org. Chem.* **52**, 1758-1764 (1987).
24. Alcaide, B., Almendros, P., Quirós, M. T., López, R., Menéndez, M. I. & Sochacka-Ćwikła, A. Unveiling the reactivity of propargylic hydroperoxides under gold catalysis. *J. Am. Chem. Soc.* **135**, 898-905 (2013).
